# Sterically Hindered Derivatives of Pentacene and Octafluoropentacene

**DOI:** 10.1002/chem.202402651

**Published:** 2024-10-25

**Authors:** Zachary W. Schroeder, Parisa Rezghi Rami, Matthias Adam, Michael J. Ferguson, Frank Hampel, Rik R. Tykwinski

**Affiliations:** ^1^ Department of Chemistry University of Alberta Edmonton, Alberta T6G 2G2 Canada; ^2^ Department of Chemistry and Pharmacy & Interdisciplinary Center for Molecular Materials (ICMM) University of Erlangen-Nuremberg Nikolaus-Fiebiger Str. 10 91058 Erlangen Germany

**Keywords:** Acene stability, Endoperoxide, Organic semiconductor, Pentacene, Stability, Steric hindrance

## Abstract

6,13‐Diethynylpentacene derivatives with sterically bulky substituents (Tr*, tris(3,5‐di‐*tert*‐butylphenyl)methyl groups) appended to the ethynyl moieties at the 6‐ and 13‐positions have been synthesized, as well as derivatives with electron‐withdrawing fluorine groups on the 1,2,3,4,8,9,10,11‐positions. These molecules are designed to investigate relationships between steric and electronic effects on the stability of pentacene toward endoperoxide formation via reaction with photosensitized oxygen in solution under ambient light (i. e., ‘laboratory’ conditions). It is evident from the study that stabilization through changes to the electronic characteristics of pentacene are more effective than the incorporation of sterically bulky groups at the acetylenic termini. Selected pentacene derivatives have been made into binary, amorphous films with the fullerene derivative PCBM to investigate the stability imparted by substituents against cycloaddition reactions. Overall, the introduction of steric protection through the incorporation of Tr* groups is not an efficient strategy for enhancing the persistence of pentacenes. Stabilization through fluorination proves successful for extending the lifetime of the pentacene derivatives by an order of magnitude in solution. Notably, the persistence of pentacene derivatives in solution can also be enhanced through the use of ethereal solvents stabilized with butylated hydroxy toluene (BHT) and/or an increased number of trialkylsilyl groups as substituents.

## Graphical abstract

Pentacene derivatives have been substituted with sterically bulky substituents (Tr*, tris(3,5‐di‐*tert*‐butylphenyl)methyl groups) appended to the ethynyl moieties in 6‐ and 13‐positions and electron withdrawing fluorine groups in the pro‐cata positions. The stability of acene derivatives toward endoperoxide formation has been evaluated in solution under ambient light and trends determined based steric and electronic characteristics of the substituents.

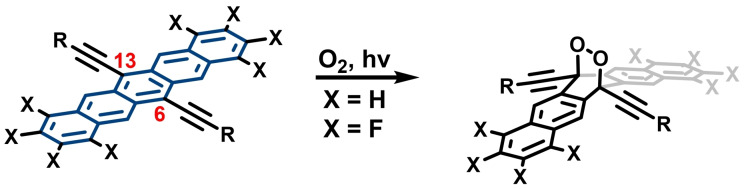

## Introduction

As chemistry and materials science enter the new millennium, the search for small molecule semiconductors has been accelerated by the report of a promising new class of molecules, namely, functionalized acenes. The star of the class has been 6,13‐bis(triisopropylsilylethynyl)pentacene,^[1]^ as well as numerous other derivatives that have been introduced and studied as a function of the structure and constitution of trialkylsilyl groups introduced at the termini of the two ethynyl groups.[^2^, ^3^] In addition to maintaining desirable p‐type semi‐conductor properties, these pentacene derivatives possess two additional advantages over unsubstituted pentacene: 1) they are more soluble, and 2) they are far more persistent (i. e., “stable”).[^4^, ^5^] It is interesting to note, however, that the relative importance of either electronic or steric effects (or a combination of the two‐factors) imparted by pendent substituents toward the stabilization of trialkylsilylethynyl functionalized pentacenes remains a topic of some debate.[^6^, ^7^, ^8^, ^9^, ^10^, ^11^]

Despite enhancements in stability offered by 6,13‐trialkylsilylethynyl groups, substituted pentacenes remain susceptible to a number of processes that lead to degradation,^[10]^ including reactions with oxygen to form endoperoxides,[^9^, ^11^, ^12^, ^13^] cyclodimerizations,[^10^, ^14^, ^15^] and Diels‐Alder (D−A) reactions in the presence of a suitable dienophile.[^11^, ^16^] The D−A reaction of the pentacene with C_60_ or [6,6]‐phenyl‐C_61_‐butyric acid methyl ester (PCBM), in particular, is a concern.[^17^, ^18^, ^19^] Thus, the use of pentacene derivatives as donors in small‐molecule devices that incorporate fullerenes as electron acceptors,[^20^, ^21^] e. g., organic solar cells, is potentially complicated by the reaction between these two semiconductor components.

**Scheme 1:**
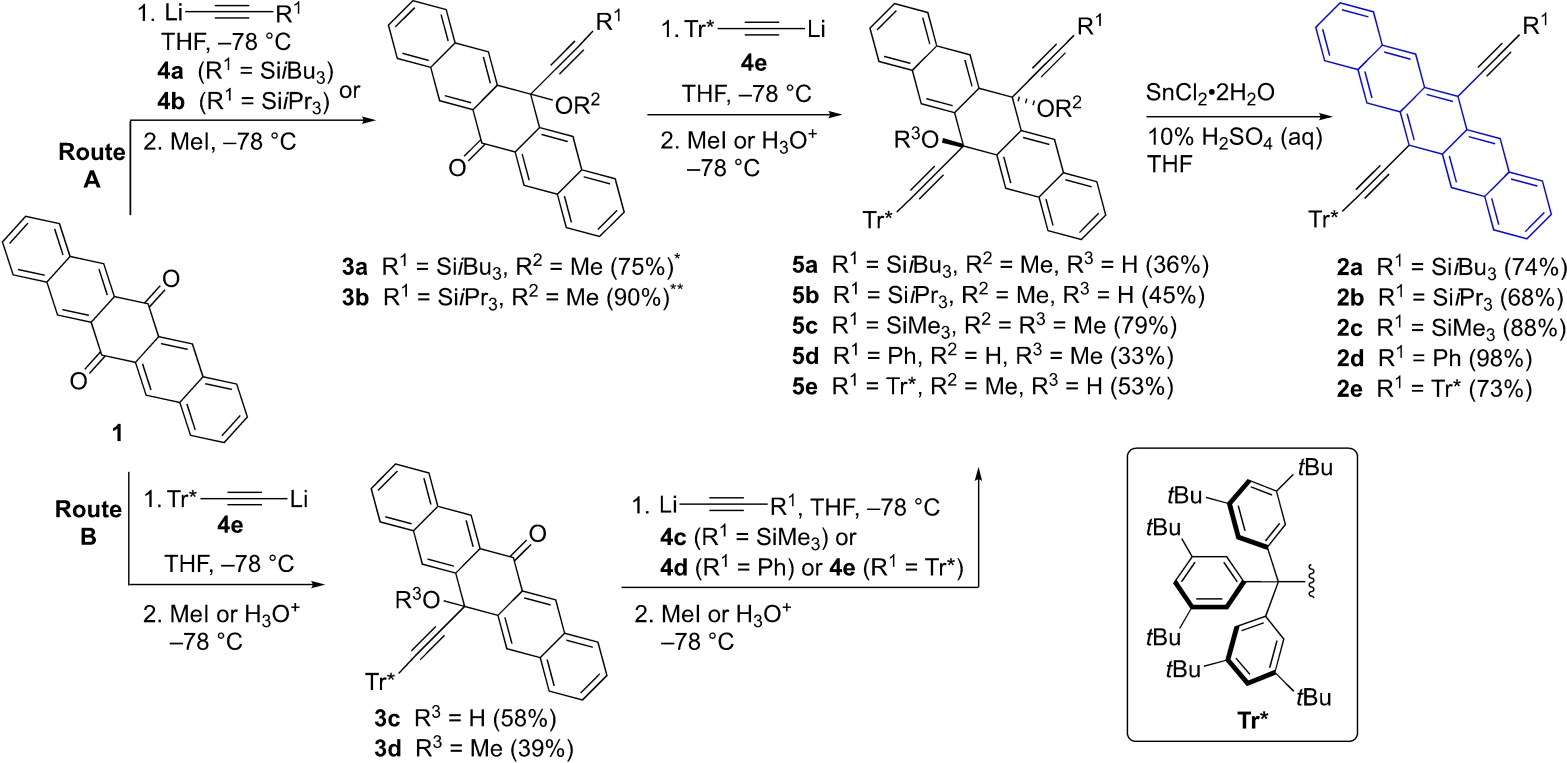
Synthesis of pentacene derivatives **2 a**–**e** (^*^ See reference [27]; ^**^ See reference [28]).

Steric constraints from pendent substituents on the pentacene core can affect both the activation energy in D−A reactions with C_60_ and, more significantly, destabilize the corresponding D−A adducts via steric repulsion, as suggested by Houk and Briseno.^[17]^ To synthesize pentacene derivatives bearing large and sterically encumbering substituent(s), the supertrityl group (Tr*, tris(3,5‐di‐*tert*‐butylphenyl)methyl) is attractive, since it could readily be appended to an ethynyl linker at the 6‐ and/or 13‐position of the pentacene framework (Scheme 1). The Tr* group has been used successfully to stabilize several highly‐conjugated compounds through steric shielding, and the presence of the *tert*‐butyl groups concurrently enhances solubility.[^22^, ^23^] The demonstrated stabilization offered by the presence of trialkylsilylethynyl substituents has been largely preserved in the pentacene targets, which have been assembled from pentacenequinone (**1**). A stepwise synthetic protocol gives access to unsymmetrical substitution at the 6‐ and 13‐positions and allows for the incorporation of trialkylsilylethynyl moieties, e. g., *i*Bu_3_Si−C≡C− (**2 a**), *i*Pr_3_Si−C≡C− (**2 b**) Me_3_Si−C≡C− (**2 c**), and Ph−C≡C− (**2 d**), or to symmetrical substitution with a second Tr*−C≡C− group (**2 e**).^[24]^

**Scheme 2:**
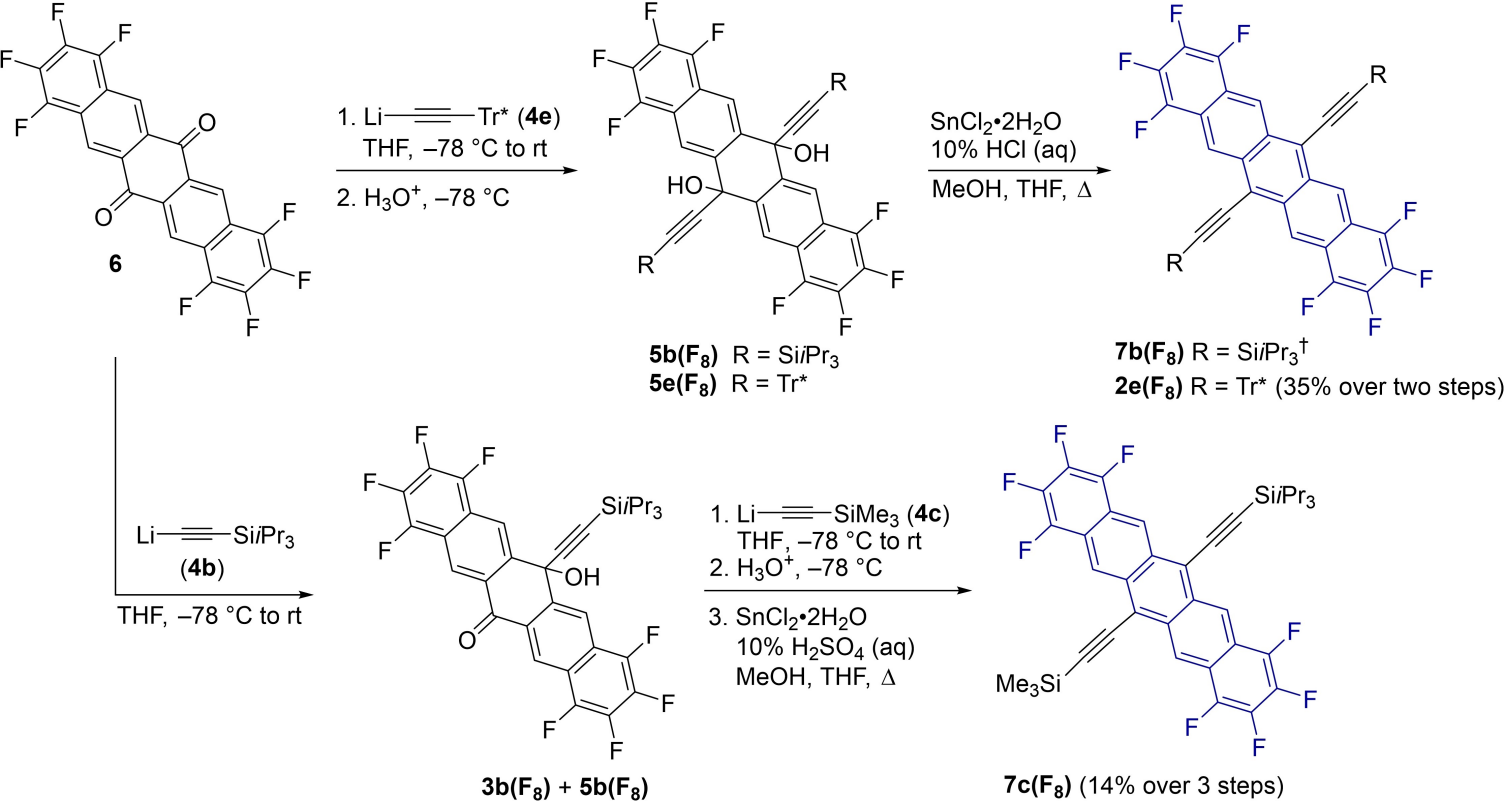
Synthesis of pentacene derivatives **2 e(F_8_)**, **7 b(F_8_**), and **7 c(F_8_)** (^†^See reference [32], no yield recorded).

Similarly, electronic effects influence the persistence of pentacene derivatives, and complementary studies have demonstrated that the incorporation of electron withdrawing functionality at the pro‐cata positions of pentacene derivatives (e. g., fluorine) leads to increased stability.[^25^, ^26^] The relatively isosteric size of H‐ and F‐atoms, and the potential to compare our results with investigations of mono‐ and di‐fluorinated pentacene derivatives by Yeates and coworkers,^[25]^ motivated the synthesis and analysis of the complementary octafluorinated pentacene derivatives (Scheme 2). The persistence of 6,13‐substituted derivatives of 1,2,3,4,8,9,10,11‐octafluoropentacene **2 e(F_8_)**, **7 b(F_8_)**, and **7 c(F_8_)** is compared with the non‐fluorinated counterparts.

In addition to the synthesis and characterization by UV‐vis spectroscopy, the structural analysis by X‐ray crystallography is presented and discussed for seven derivatives (**2 a**–**e**, **2 e(F_8_)**, and **7 c(F_8_)**). Finally, the reactivity of pentacene derivatives toward oxygen (endoperoxide formation), as well as preliminary studies that examine the stability of these sterically demanding pentacene derivatives toward reactions with PCBM, have been investigated using UV‐vis and NMR spectroscopy.

## Results and Discussion

Pentacenequinone (**1**) was used as the common building block for the synthesis of sterically‐hindered pentacene derivatives **2 a**–**e** (Scheme 1) with the supertritylethynyl group incorporated as a common substituent in the 6‐position. The size and constitution of the pendent group in the 13‐position has then been systematically modified. Two different stepwise assembly routes were used. Starting with **1**, ketones **3 a** ^[27]^ and **3 b** ^[28]^ were synthesized via **Route A** according to published procedures using acetylides **4 a** and **4 b**. Ketones **3 a** and **3 b** were then transformed into **5 a** and **5 b**, respectively, by reacting with acetylide **4 e**, which was formed from Tr*‐acetylene^[29]^ via lithiation with *n‐*BuLi. Alternatively, in **Route B** the Tr*‐acetylene group could be introduced in the first step of the synthesis through the reaction of **1** with acetylide **4 e** to give **3 c** or **3 d**, after quenching with either a proton source or MeI, respectively. In the second step, acetylides **4 c**–**4 d** were used in nucleophilic additions to ketone **3 d**, resulting in compounds **5 c**–**d**. Finally, the reaction of acetylide **4 e** with ketone **3 c** gave **5 e**.^[30]^ Reductive aromatization^[31]^ of **5 a**–**e** using SnCl_2_ afforded the desired products **2 a**‐**e** containing the Tr* group, which were purified by column chromatography. The overall yields of **2 a**–**e** from **1** were ca. 20–30 % regardless of the synthetic route utilized (i. e., **Route A** or **B**), and there is no discernible trend in yield of the reductive elimination based on substitution of the precursors **5 a**–**e**.

The synthesis of octafluorinated pentacene derivative **2 e(F_8_)** was accomplished using the method reported by Anthony and coworkers for **7 b(F_8_)** ^[32]^ (Scheme 2). Thus, the reaction of octafluoropentacenequinone (**6**)[^33^, ^34^] with excess acetylide **4 e** provided the intermediate diol **5 e(F_8_)**, which was isolated by column chromatography (the excess Tr* acetylene could also be recovered). Reductive elimination of **4 e** with SnCl_2_ gave **2 e(F_8_)** as a blue crystalline solid in 35 % yield over the two steps. The unsymmetrical derivative **7 c(F_8_)** was synthesized using a stepwise approach. The addition of acetylide **4 b** to **6** gave the mono‐addition product **3 b(F_8_)**, which was isolated by trituration in hexanes and filtration (ca. 85 % pure). The corresponding diol **5 b(F_8_)** was also isolated from the mother liquor and further purified by column chromatography. The subsequent reaction of **3 b(F_8_)** with excess acetylide **4 c**, workup, and reductive aromatization afforded **7 c(F_8_)** in 14 % yield over the three steps. Finally, a sample of compound **7 b(F_8_)** was prepared by reductive aromatization of **5 b(F_8_)**, as reported by Anthony and coworkers.[^32^, ^34^]

**Figure 1:**
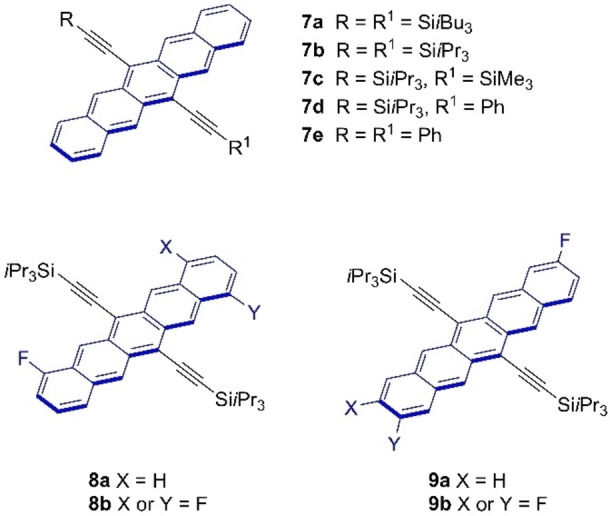
Structures of 6,13‐ethynylated pentacene derivatives used as reference compounds for comparison and meta‐analysis in the present study (**7 a**, reference [35]; **7 b**, **7 c**, and **7 d**, reference [36]; **7 e**, reference [37]; **8 a**, **8 b**, **9 a**, and **9 b**, References [25, 26]). Difluorinated derivatives **8 b** and **9 b** were reported and studied as mixtures of *syn*/*anti* isomers.

**Figure 2:**
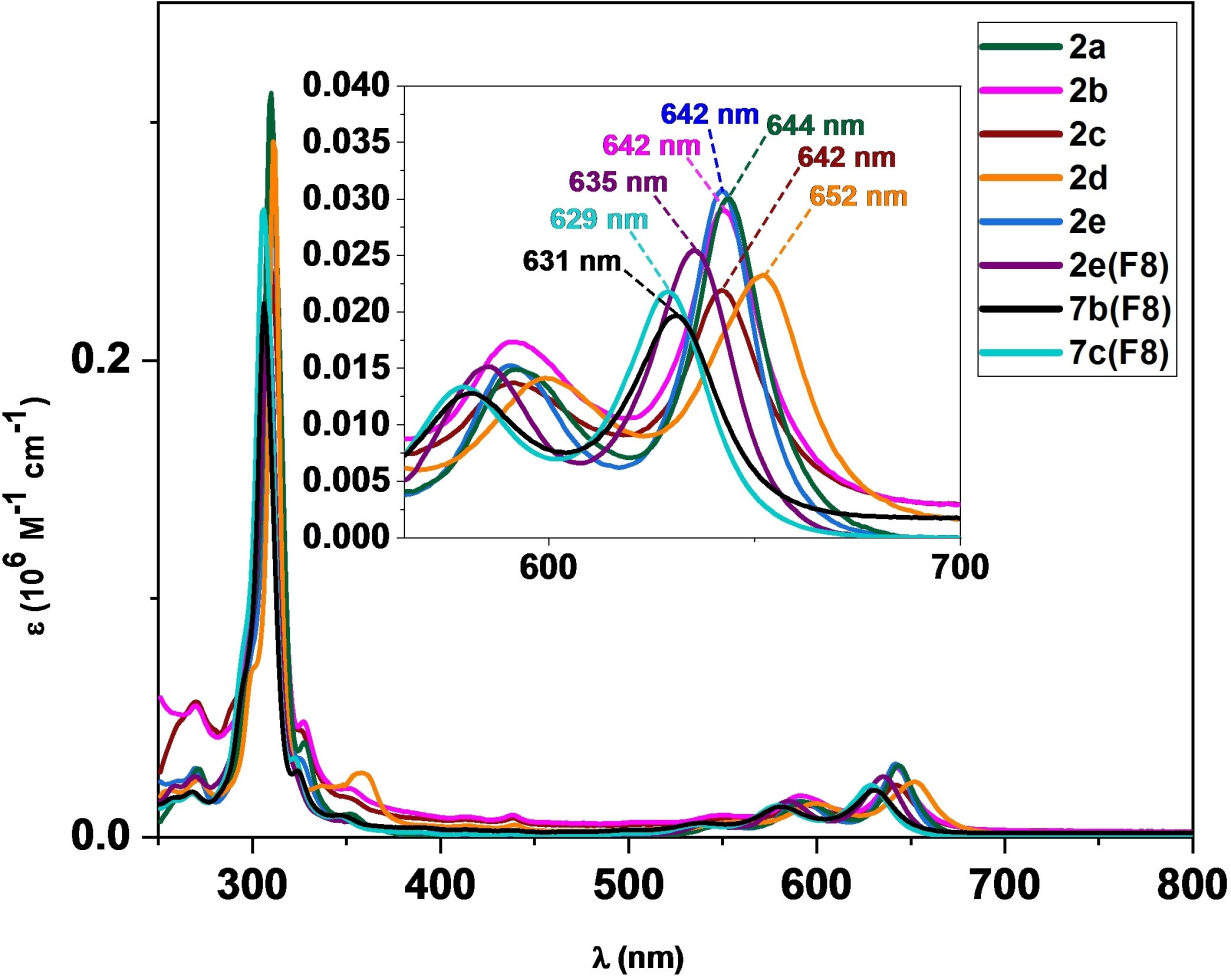
UV‐vis absorption spectra for pentacene derivatives **2 a**–**e**, **2 e(F_8_)**, **7 b(F_8_)**, and **7 c(F_8_)** in CH_2_Cl_2_ (250–800 nm). Inset: Low energy absorption region with low energy *λ*_max_ values provided.

**UV‐vis spectroscopy**. UV‐vis spectra of compounds **2 a**–**e**, **2 e(F_8_)**, **7 b(F_8_)**, and **7 c(F_8_)** have been measured in CH_2_Cl_2_ to examine the electronic effects of Tr*‐substitution on pentacene derivatives in comparison to compounds **7 a** and **7 b** (Figure 1). Compounds **2 a**–**e**, **2 e(F_8_)**, and **7 b**–**c (F_8_)** all absorb in the range of 250–750 nm in CH_2_Cl_2_ with *λ*_max_ values ranging from 631–652 nm (Figure 2). The strongest absorption for non‐fluorinated compounds **2 a**–**e** is observed at *λ*_max_=310–311 nm, with a series of low energy absorptions with *λ*_max_=642–644 nm (**2 a**–**c**, **2 e**), nearly identical to **7 a** (*λ*_max_=644 nm)^[35]^ and **7 b** (*λ*_max_=643 nm).^[36]^ Compound **2 d** shows a bathochromic shift of the low energy absorption to *λ*_max_=652 nm relative to **2 a**–**2 c**. The bathochromic shift is consistent with **7 d** (*λ*_max_=652 nm), which also bears a pendant phenyl substituent.^[36]^ Thus, the bathochromic shift in **2 d** is attributed to the extension of the *π*‐system provided by the pendant phenyl ring. The *λ*_max_ values of compounds **2 a**–**e** are comparable to similarly functionalized derivatives **7 a**–**d** and demonstrate that the electronic effect of the Tr* group on the pentacene core of **2 a**–**e** is comparable to that of the SiR_3_ group in compounds **7 a**–**d**. The absorption spectra of octafluoropentacenes **2 e(F_8_)** and **7 b**–**c(F_8_)** follow similar trends and closely resemble the prototypical absorption profile of the non‐fluorinated counterparts (**2 e**, **7 b**–**7 c**). The key difference is a hypsochromic shift of the low energy absorption of **2 e(F_8_)** (*λ*_max_=635 nm) vs **2 e** (*λ*_max_=642 nm), **7 b(F_8_)** (*λ*_max_=631 nm) vs **7 b** (*λ*_max_=643 nm), and **7 c(F_8_)** λ_max_=629 nm) vs **7 c** (*λ*_max_=643 nm). Due to the structural similarities, the observed hypsochromic shifts of *λ*_max_=7–11 nm is attributed to electron‐deficient fluorine substituents. Refer to Table S4 in the ESI for the low energy *λ*_max_ values for all derivatives.

**Stability in solution**. It is well known that pentacene derivatives degrade faster in solution than in the solid state, most commonly due to photo‐mediated reactions with O_2_.[^7^, ^9^, ^38^] Understanding the persistence of new pentacene derivatives in solution under laboratory conditions is crucial to the development of synthetic methods and purification procedures. Beyond that, persistence is paramount for the implementation of chromophores into functional devices with sufficiently long application lifetimes. To evaluate stability in solution under laboratory conditions, several derivatives have been chosen for UV‐vis spectroscopic studies, including **2 a**–**2 e**, **2 e(F_8_)**, **7 a**, **7 b(F_8_)**, and **7 c(F_8_)** under the rational that a) the *i*Pr_3_Si and *i*Bu_3_Si groups are most commonly found in pentacene derivatives offering an optimal balance of solubility and crystallinity (**2 a**–**b**, **7 a**, **7 b(F_8_)**, and **7 c(F_8_)**), b) the Tr* group offers relatively large steric hindrance to the pentacene core (**2 a**–**b**, **2 e**, and **2 e(F_8_)**) and c) the octafluorination of the core enables the electronic contributions to stability to be directly compared against steric effects imparted by the Tr* group(s) (**2 e(F_8_)**, **7 b(F_8_)**, and **7 c(F_8_)**). To evaluate kinetic stability,^[39]^ the following conditions were used in our study (refer to ESI Figures S1–S50 for full graphical and procedural details):

Solutions with CH_2_Cl_2_ as the solvent, in the dark and either with or without deoxygenation; CH_2_Cl_2_ was selected since it is the most versatile solvent for solubilizing the range of pentacene derivatives under study (Figures S1–S4).

Solutions with stabilizer‐free THF (i. e., the stabilizer BHT, butylated hydroxytoluene, removed) in the presence of ambient laboratory light and either with or without deoxygenation (Figures S5–S11).

Solutions with THF containing the stabilizer BHT, in either the presence or absence of ambient laboratory light and either with or without deoxygenation (Figures S12–S20).

Samples were monitored by UV‐vis spectroscopy, examining the decrease in the intensity of *λ*_max_ over time, and the results are summarized in Table 1 (vide infra).

**Table 1:** Light‐mediated endoperoxidation in solution (half‐lives, *t*_1/2_).^[a]^

	CH_2_Cl_2_^[b]^		Stabilizer free THF^[c]^		BHT stabilized THF^[d]^	
Cmpd	Air (h)	N_2_‐sat (h)	Air (h)	N_2_‐sat (h)	Air (h)	N_2_‐sat (h)
**2 a**	14	22	4.0	5.5	15.2	71.9
**2 b**	7.7	14	3.6	4.8	15.1	50.0
**2 e**	8.6	9.4	2.8	3.3	7.1	30.2
**7 b(F_8_)**	–^[f]^	– ^[f]^	– ^[f]^	– ^[f]^	332^[e]^	–^[f]^
**7 c(F_8_)**	– ^[f]^	– ^[f]^	– ^[f]^	– ^[f]^	271^[e]^	– ^[f]^
**2 e(F_8_)**	– ^[f]^	–^[f]^	– ^[f]^	– ^[f]^	95.7^[e]^	– ^[f]^
**7 a**	– ^[f]^	– ^[f]^	– ^[f]^	– ^[f]^	24.3	59.4

[a] Half‐lives (*t*_1/2_) have an estimates error of <10 % based on trials run in duplicate. [b] CH_2_Cl_2_ was used directly from the solvent purification system. For graphical results, see Figures S1–S4 in the ESI. [c] THF was purchased without stabilizer (butylated hydroxytoluene) and used without further purification. For graphical results, see Figures S5–S11 in the ESI. [d] HPLC grade THF purchased with stabilizer (butylated hydroxytoluene, 250 ppm and was used without further purification). For full graphical results see Figures S12–S20 for **2 a**, **2 b**, **2 e**, and **7 a**; Figures S21–S27 for **2 e(F_8_)**, **7 b(F_8_)**, and **7 c(F_8_)** in the ESI. [e] Average of two trials. [f] Experiments were not conducted.

Stability tests in CH_2_Cl_2_ confirmed that endoperoxidation of compounds **2 a**, **2 b**, and **2 e** was negligible in the absence of light (Figures S1–S4 in the ESI). Thus, subsequent studies of these derivatives omitted dark controls to avoid redundancy.^[39]^ Furthermore, the use of CH_2_Cl_2_ proved problematic due to evaporation in studies that spanned longer time frames, and subsequent photochemical stability experiments were therefore conducted in THF. For THF and CH_2_Cl_2_, the dielectric constants are comparable (ca. 7.5 and 8.9, respectively^[40]^) as is the solubility of oxygen (*x*=8.16×10^−4^ mol^−1[41]^ and *x*=7.09×10^−4^ mol^−1^,^[42]^ respectively, at 298 K and 101.3 kPa partial pressure), but the boiling point of THF (ca. 66 °C) is higher than CH_2_Cl_2_ (ca. 40 °C). Furthermore, the solubility of pentacene derivatives in THF is generally good, making it an effective alternative.

Solutions of THF were used without BHT as a stabilizer under ambient laboratory lighting, and the changes in absorption at *λ*_max_ versus time for **2 a**, **2 b**, and **2 e** were exponential with *t*_1/2_=2.5–6 h at rt (Table 1). The rate of endoperoxide formation under analogous conditions using THF containing BHT was reduced, with *t*_1/2_=7–72 h at rt (Table 1). The stability observed in BHT stabilized THF (250 ppm concentration of BHT) is not unexpected and results from the propensity of BHT to quench reactive oxygen species such as superoxide radicals and singlet oxygen.^[43]^ Based on the observed rates of endoperoxide formation (Table 1), the compound bearing two Tr* groups, **2 e**, is the least stable of the non‐fluorinated pentacene series that have been examined under ambient conditions (Figure S5). Therefore, it is concluded that the presence of Tr* groups does not enhance the stability of 6,13‐diethynylpentacene derivatives against reactive oxygen species. Other stabilizing factors, such as the electronic contributions of substituents, could also play a significant role, and a hypothesis centered on substituent size in the 6,13‐ethynyl groups termini is insufficient. The nature of the terminal group appears more significant, as suggested by a linear relationship between the derivatives half‐lives and the number of silicon atoms present in the termini of the 6‐ and 13‐ethynyl groups of the molecule (Figure S28), namely **2 e** (*t*_1/2_=7.1 h), **2 a** (*t*_1/2_=15.2 h), and **7 a** (*t*_1/2_=24.3 h).^[44]^ This is in agreement trend with the results of Northrop and coworkers, who reported stability enhancements in a 6,13‐bis‐trimethylsilylacetylenepentacene, which contradicted their computationally derived results.^[7]^ While this trend remains semi‐empirical, to our knowledge, this is the first experimentally derived observation relating the stability of 6,13‐diethynylpentacene derivatives to the number of trialkylsilyl substituents.

The lack of kinetic stabilization afforded by the bulky Tr* group motivated investigation into the effects of electron‐withdrawing groups on pentacenes persistence. Using BHT‐stabilized THF under ambient laboratory conditions, the intensity of *λ*_max_ was monitored over time for compounds **2 e(F_8_)**, **7 b(F_8_)**, and **7 c(F_8_)** (Table 1, Figures S21–S27).^[45]^ In general, the half‐lives of the fluorinated pentacene derivatives **2 e(F_8_)** (*t*_1/2_=95.7 h) and **7 c(F_8_)** (*t*_1/2_=271 h) are enhanced by approximately an order of magnitude versus the non‐fluorinated analogs such as for example, **2 e** (*t*_1/2_=7.1 h) and **7 a** (*t*_1/2_=24.3 h). Also noteworthy is the comparison of **2 e(F_8_)** (*t*_1/2_=95.7 h) and **7 b(F_8_)** (*t*_1/2_=332 h), corroborating the stabilizing influence of trialkylsilyl substituents observed in the non‐fluorinated derivatives.

**Figure 3:**
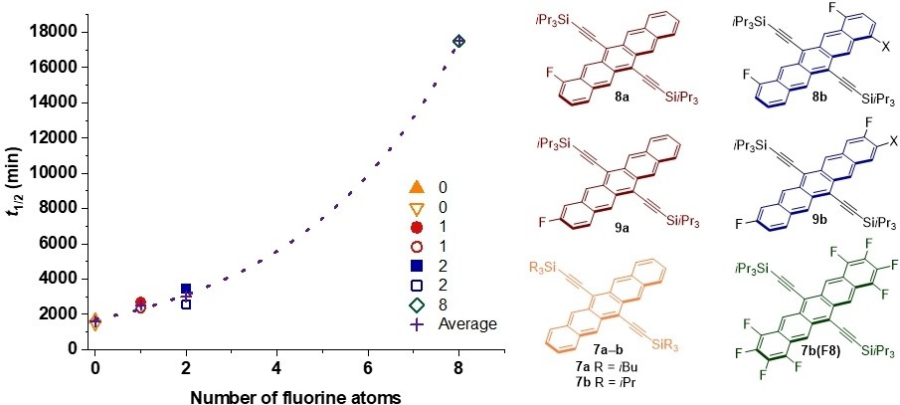
Graphical relationship of endoperoxidation half‐life (*t*_1/2_) and the number of fluorine atoms.^[25]^ The compounds used are as follows: fluorine atoms=none (**7 a**–**b**), one (**8 a**–**9 a**),^[25]^ two (**8 b**–**9 b**),^[25]^ and eight (**7 b(F_8_)**). Data for compounds **8 a**–**9 a** ^[25]^ and **8 b**–**9 b** ^[25]^ was obtained in CHCl_3_ and compound **7 b(F_8_)** was measured in THF.^[46]^ The X substituent represents the syn‐anti mixtures of **8 b** and **9 b** where X=the alternate position of one fluorine substituent.

The influence of fluorine substitution is intriguing, and the results for **7 b(F_8_)** are compared to the lifetimes obtained by Yeates and coworkers for mono‐ and difluorinated pentacene derivatives (Figure 3).[^25^, ^46^] An exponential relationship is observed when the half‐lives for **7 b**, **8 a**–**b**, **9 a**–**b**, **7 a**, and **7 b(F_8_)** are plotted as a function of the number of fluorine atoms in their structure.^[25]^ The fit is further improved when the half‐lives of the two monofluorinated (**8 a** and **9 a**) and two difluorinated pentacene regioisomers (**8 b** and **9 b**) are averaged together (**+** in Figure 3). Since **8 a**/**9 a** and **8 b**/**9 b** are each regioisomeric pairs and **8 b**/**9 b** were synthesized as mixtures of *syn*‐*anti* regioisomers, the averaged half‐lives conveniently represent all the potential mono‐ and difluorinated substitution patterns available at the peripheral rings of pentacene. The early trend, with mono‐ or difluorination, appears more linear in nature, and the exponential relationship only evolves when adding data for the octafluorinated derivative **7 b(F_8_)**.

**Figure 4:**
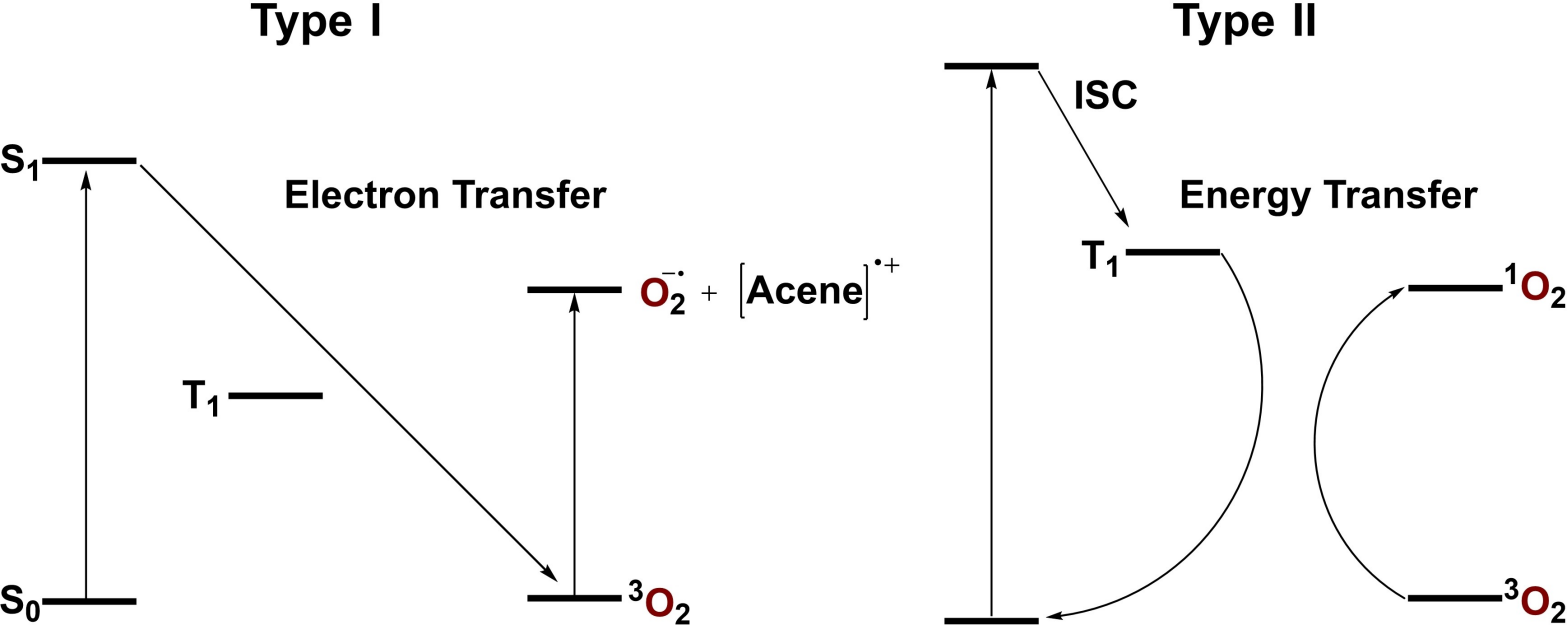
Jablonski diagrams describing *Type I* (left) and *Type II* (right) oxygen sensitization of a photoexcited acene.

There are two common categories of oxygen sensitization by a chromophore that has been photoexcited: *Type I* and *Type II*.[^6^, ^7^, ^11^] *Type I* sensitization occurs when the singlet excited state of a chromophore affects a one‐electron reduction of oxygen to produce a chromophore radical cation and superoxide radical anion (Figure 4). *Type II* sensitization occurs when a high‐lying triplet state of a chromophore allows energy transfer to oxygen to produce singlet oxygen and a ground‐state chromophore (Figure 4). The optical HOMO‐LUMO gap of **7 b(F_8_)** is reported to be nearly analogous with non‐fluorinated TIPS‐pentacene **7 b**.^[34]^ Likewise, the triplet state energy of pentacene is lower than that of singlet O_2_. Thus, the ability for *Type II* oxygen sensitization to occur via energy transfer from the triplet state would not be expected as a major contributor to the rate of endoperoxidation due to the relative endothermicity of the process. To this end, *Type I* sensitization is considered predominantly in the following discussion.

The endoperoxidation process can be stepwise with a diradical intermediate or concerted.^[11]^ Endoperoxidation of 6,13‐diethynylpentacenes favors the central 6‐ and 13‐positions, i. e., those with the most significant orbital coefficients.^[11]^ A small proportion of the 5,14‐endoperoxide is also reportedly formed, as observed by UV‐vis analysis.^[11]^ The 5,14‐endoperoxide has a characteristic UV‐vis absorption of anthracene, and this signature has also been observed during stability studies reported herein (Figures S5–S11).

**Figure 5:**
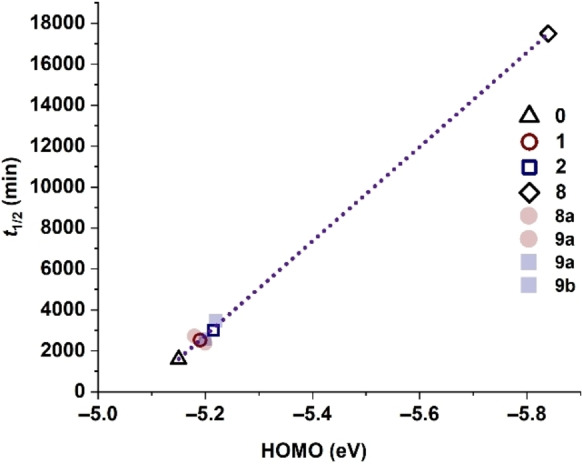
Graphical depiction between half‐life (*t*_1/2_) of the endoperoxide formation versus the energy of the HOMO as a function of the number of fluorine atoms as substituents on the pentacene derivative.[^25^, ^34^] Shaded data points ( 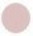 and 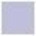) are values for individual mono‐ and difluorinated regioisomers, respectively, whereas hollow points ( 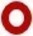 and 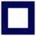) are averages of the two mono‐ and difluorinated regioisomers, respectively.

The linear relationship between pentacene half‐life and HOMO energy of fluorinated pentacenes is consistent with our results, as suggested by Yeates and coworkers (Figure 5). Adding our half‐life data for **7 b(F_8_)**, the linear relationship is maintained between fluorination and HOMO energy.^[47]^ It would be expected that half‐lives plotted as a function of LUMO energies would show an analogous trend as a direct consequence of the synchronous decrease in FMO energies.^[25]^ Note that both the individual and average HOMO energy values of the mono‐ and difluorinated regioisomers are plotted. Yeates and coworkers demonstrate that the fluorine substituents at the 1‐ and 2‐positions of pentacene influence the redox properties differently.^[25]^ Thus, averaging the two regioisomers results in a better fit of the data when compared to **7 b(F_8_)**.

**Figure 6:**
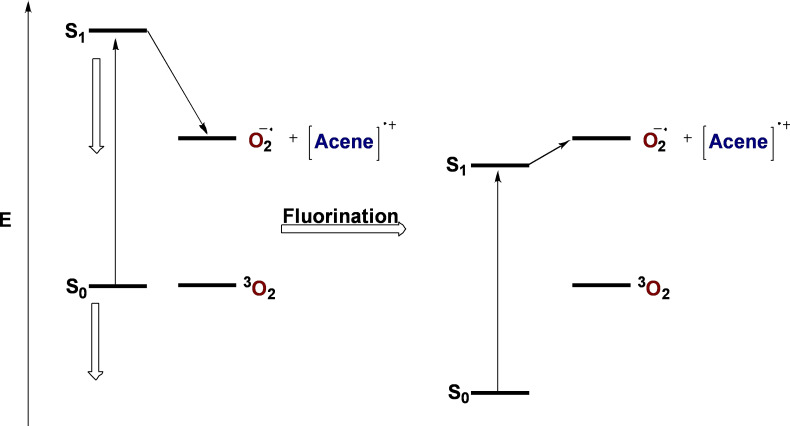
Qualitative depiction of energy states for *Type 1* photoexcited electron transfer of non‐fluorinated (left) and fluorinated (right) pentacene depicted with relative but arbitrary energies.

Since *Type I* electron transfer occurs from the higher‐lying excited state SOMO of pentacene, which is analogous to the LUMO of the ground state molecule, the synchronous decrease in LUMO and HOMO energies can reduce the driving force of electron transfer to O_2_. Thus, as the energy of the LUMO of pentacene decreases upon fluorination, the driving force imparted by photoexcitation becomes less significant if the HOMO‐LUMO gap remains approximately constant. The results obtained for this series support the conclusion that electron transfer to O_2_ can be predictably controlled by increasing the endothermicity of the electron transfer process with O_2_, similar to the findings made by Yeates and coworkers.^[25]^ The absolute and relative energies of the FMOs should be considered to predict stability towards endoperoxidation. The schematic relationship between half‐life and fluorination observed for pentacene is depicted with arbitrary energies in Figure 6.

Furthermore, a fluorinated pentacene in the ground‐state (S_0_) could quench the superoxide radical anion generated from *Type II* sensitization via reverse electron transfer, which would reform triplet oxygen and create a pentacene radical anion. The resultant pentacene radical cation‐anion pair could then react back to the ground state. This quenching mechanism would enhance stability in the solution at a rate that would be under diffusion control. Likewise, back electron transfer could also contribute to the persistence of the fluorinated pentacene derivatives. Due to the reduced energies of the FMOs in the octafluorinated pentacene compared to the unfluorinated counterparts, the rate of back electron transfer from the superoxide radical anion could be enhanced and potentially outcompete endoperoxidation. This route would generate ^1^O_2,_ which has been demonstrated to react significantly slower with pentacene than the superoxide radical anion.^[11]^ As our studies did not probe the formation of singlet oxygen, this mechanism cannot be excluded as a potential stabilization pathway.

**Pentacene stability in the presence of PCBM**. Pentacene and fullerene derivatives are attractive semiconductors for donors and acceptors in solar energy capture, respectively.^[4]^ It has been established that pentacene derivatives undergo Diels‐Alder cycloaddition reactions with C_60_ and its derivatives in the solid state.[^18^, ^48^] To that end, the potential of the steric encumbrance of the Tr* group to provide chemical stability to pentacene derivatives has been explored. The reactivity of derivatives **2 a** and **2 e** was investigated in the presence of PCBM, a common acceptor used in organic solar cells.^[49]^ Binary mixtures of PCBM with either **2 a** or **2 e** were made as solutions in stabilized THF (ca. 5×10^−6^ to 1×10^−5^ mM), and UV‐vis analysis of the solutions showed no significant changes (i. e., no evidence of cycloaddition). The solvent was removed under a stream of dry N_2_ in the dark, and the resulting solid was then dissolved and analyzed by UV‐vis spectroscopy. The spectra of the resulting solutions show a significantly lower intensity of the *λ*_max_ values at 644 nm (**2 a**) and 642 nm (**2 e**),^[50]^ as well as increased high‐energy absorptions. These changes are consistent with disruption of the conjugated backbone of pentacene through a cycloaddition reaction with a fullerene (full experimental details in ESI; Table S2 and Figures S29–S32).^[8]^

In a complementary NMR spectroscopy study, derivatives **2 a** and **2 e** were dissolved in C_6_D_6_ with PCBM (full experimental details in ESI; Table S3 and Figures S33–S36). The solvent was removed under a stream of dry N_2_ in the dark, and the resulting solid was again dissolved in C_6_D_6_. Analysis by NMR spectroscopy showed degradation of the pentacene groups consistent with the UV‐vis analysis, and solutions of pentacene **2 a** and **2 e** display the evolution of a myriad of new signals in their respective spectra as a function of film formation, redissolution, and subsequent analysis.^[51]^ Interestingly, by NMR spectroscopy, **2 e** appeared to show less degradation compared to the study by UV‐vis spectroscopy, perhaps due to solvent effects (i. e., C_6_D_6_ versus THF).

The results of these experiments suggest that the introduction of sterically encumbering Tr* groups at the 6‐ and 13‐positions of **2 e** does not provide significant enhancements of stability towards cycloaddition reactions with PCBM in the solid state compared to **2 a** featuring only a single Tr* group. Notably, however, there appears to be a putative solvent dependence observed when comparing results for the experiments in THF (UV‐vis spectroscopy) versus C_6_D_6_ (NMR spectroscopy). Namely, the pentacene chromophore appears more persistent in the NMR experiments for compound **2 e** compared to the UV‐vis results, although we have not investigated this observation further.

**Thermal behavior**. Traditional melting point analysis (MPA) can be inconvenient for the characterization of pentacene derivatives because the dark blue color of samples renders small changes in color and/or phase challenging to observe. Thus, differential scanning calorimetry (DSC) was used in conjunction with MPA to evaluate the thermal characteristics of compounds **2 a**–**e**, **2 e(F_8_)**, and **7 b**–**c(F_8_)**, in comparison to previously reported **7 a**–**e** as shown in Table S4. The DSC analysis of derivatives **2 a**–**c**, and **2 e** shows an endotherm from melting, followed immediately by exothermic decomposition, which is similar to the behavior of compounds **7 a**–**c** (Figure S88–S97).^[25]^ Due to an unexplained endotherm observed for **2 e**, at 90 °C, a DSC cycling experiment (Figure S93) and MPA under N_2_ (Figure S95) were performed. These experiments confirmed that the low temperature endotherm observed in DSC analysis was not a melt. We posited that said phase transition was in relation to trialkylsilyl group dynamics as reported previously.[^25^, ^52^] Phenylethynyl derivatives **2 d**, **7 d**, and **7 e** all decompose directly without melting. Thus, the thermal stability of **2 e** is not obviously enhanced by introducing sterically bulky Tr* groups to the periphery beyond this derivative showing the highest melting point of the series (321 °C). Fluorination does not impart any notable enhancement of thermal stability of the pentacene derivatives. Compound **2 e(F_8_)** shows an endotherm at 305 °C, but no clear decomposition was evident at higher temperatures. Further, compound **7 b(F_8_)** decomposed immediately upon melting at 313 °C, and **7 c(F_8_)** decomposed at 289 °C prior to melting (refer to Table S4 and Figures S98–S100 in the ESI for details). In general, the data suggests that replacement of trialkylsilyl groups for Tr* groups on the 6,13‐ethynyl moieties and replacing pro‐cata hydrogens with fluorine atoms both have no significant effect on the thermal stability of pentacene based on DSC analysis, suggesting that packing of the chromophores in the solid state is a dominant effect, i. e., packing influences melting point, and decomposition occurs in most derivatives immediately following melting.

**Figure 7:**
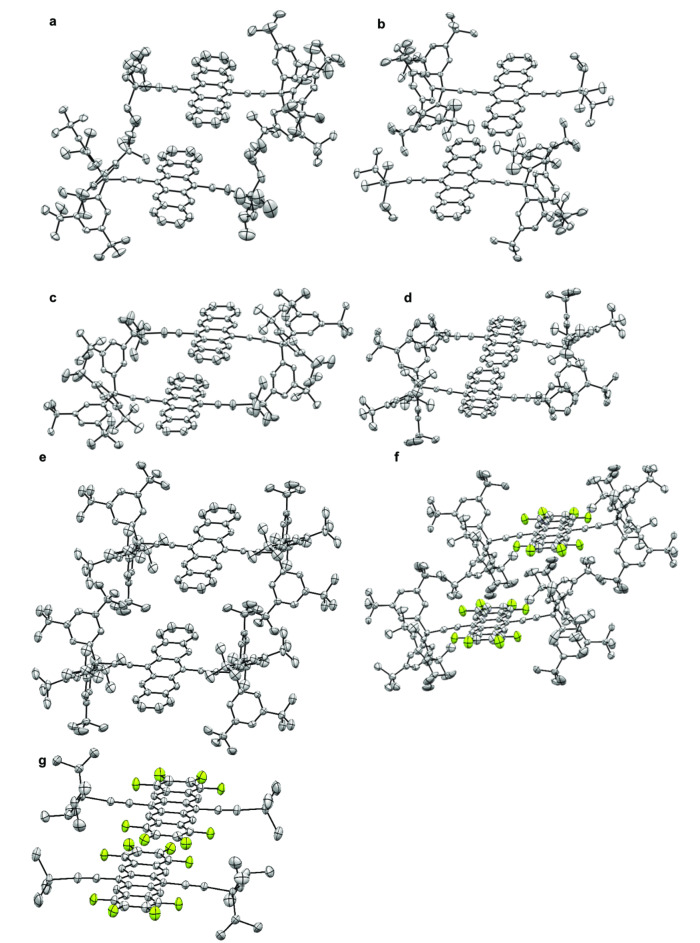
X‐Ray crystallographic structures and packing interactions of pentacene derivatives a) **2 a**, b) **2 b**, c) **2 c**, d) **2 d**, e) **2 e**, f) **2 e(F_8_)**, and g) **7 c(F_8_)**. Co‐crystallized solvent molecules, disordered atoms, and hydrogens were omitted for clarity; all thermal ellipsoids were generated at a 50 % probability level.

**X‐Ray crystallographic analysis**.^[53]^ Solid‐state packing of pentacene derivatives is strongly affected by peripheral substituents, and intermolecular interactions are a central aspect for their use in electronic applications.[^4^, ^5^, ^32^, ^38^] The solid‐state structures has been examined by X‐Ray crystallographic analysis for compounds **2 a**–**2 e**, **6 a**–**6 b**, **2 e(F_8_)**, and **7 c(F_8_)** to examine the influence of the Tr* groups (Figure 7). Crystals are grown by slow evaporation at 4–23 °C from a concentrated solution in CH_2_Cl_2_ or CHCl_3_, which has been layered with either MeOH (**2 a** and **2 b**), CH_3_CN (**2 c**), or hexanes (**2 d** and **2 e**). Structures for **2 a**, **2 b**, **2 e**, and **2 e(F_8_)** show disorder in the *t*‐butyl groups. The *i*Bu_3_Si groups in **2 a** and Me_3_Si groups in **2 c** are also disordered. Several of the structures are solvated, including **2 b** (CH_2_Cl_2_), **2 d** (hexane), **2 e** (CH_2_Cl_2_), and **2 e(F_8_)** (CHCl_3_), likely the result of inefficient packing of the Tr group(s).

Pentacene **2 a** crystallizes in a monoclinic crystal system with eight molecules in the unit cell arranged into centrosymmetric dimeric pairs. The Si‐acetylene moiety is distorted from linearity to 171–172°. Two neighboring molecules of the dimeric pair show *π‐π* interactions between neighboring acene moieties, at ca. 3.4–3.5 Å, but overlap of the dimeric pair is limited to ca. one atom of the terminal aryl rings (Figure 7a). Intermolecular overlap of the *π*‐systems between neighboring dimeric pairs is, however, minimal and long‐range *π‐π* interactions are not present. Formal exchange of triisobutylsilyl groups (**2 a**) with triisopropylsilyl groups (**2 b**) has minimal effects on the packing. Pentacene **2 b** crystallized in a monoclinic crystal system with four molecules in the unit cell arranged into centrosymmetric dimeric pairs. The plane‐to‐plane distance of the dimeric acenes is ca. 3.5 Å. While neighboring molecules of the dimeric pairs appear to adopt a co‐facial arrangement (Figure 7b), there is minimal overlap of the two molecules. There are no *π‐π* interactions between neighboring dimeric pairs, and, thus, long‐range *π*‐stacking is absent. Pentacene **2 c** crystallized in a triclinic crystal system with two molecules in the unit cell arranged as a centrosymmetric dimeric pair (Figure 7c). With the smaller trimethylsilyl group, the two pentacene moieties pack as a dimeric pair with partially overlapped aromatic rings and an interplanar distance of 3.3 Å. Neighboring dimeric pairs are aligned with minimal *π‐π* interactions, likely at the limit of what would constitute a long‐range interaction.

Pentacene **2 d** crystallizes in a triclinic crystal system with two molecules in the unit cell arranged as a centrosymmetric dimeric pair (Figure 7d). With a pendant phenyl group, the two pentacene moieties of the pair overlap by almost three benzene units at an interplanar distance of 3.4 Å. The dimeric pairs are isolated from each other, however, and long‐range orbital overlap is not present.

Bearing two Tr* groups, pentacene **2 e** crystallizes in a triclinic crystal system with two molecules in the unit cell arranged as a centrosymmetric dimeric pair (Figure 7e). The bulky Tr* moieties result in an offset arrangement of the molecules that precludes overlap of the *π*‐systems in the pair.

The octafluorinated analog of **2 e**, pentacene **2 e(F_8_)**, crystallizes in a monoclinic crystal system with two molecules in the unit cell arranged as 1‐D strands (Figure 7f). Neighboring acenes, however, are separated by the Tr* groups. The *π*‐systems have a plane‐to‐plane distance of 8.6 Å, and, thus, there is no *π*‐overlap between acenes.

The unsymmetrically silylated pentacene **7 c(F_8_)** crystallizes in a triclinic crystal system with six molecules in the unit cell, arranged in a one‐dimensional slip‐stack arrangement. Neighboring pentacene groups of the slip‐stacked arrangement overlap by almost three benzene units, with one fluorinated ring overlapping with the central benzene unit at an interplanar distance of 3.3 Å. Long‐range orbital overlap is thus present along the slip‐stacked arrangement in one crystallographic dimension.

Considering the seven solid‐state structures, the key conclusions are:

The Tr*‐group appears to prevent long‐range order of the molecules such that significant *π*‐overlap of the acene is not observed beyond that in a dimeric pair of neighboring molecules.

Fluorination enhances co‐facial overlap between neighboring pentacene groups. Nevertheless, it does not overcome the steric encumbrance imparted by the Tr* end groups that inhibited long‐range overlap of the acenes *π*‐systems.

The Tr* groups do not appear to be sterically bulky enough to inhibit the approach of a small O_2_ molecule (Figure 7). However, these substituents do seem qualitatively large enough to cause at least some steric interference for a Diels‐Alder reaction with PCBM or homo‐dimerization.

## Conclusions

The introduction of the Tr*‐acetylene group(s) onto 6‐ and/or 13‐positions of the pentacene chromophore does not lead to improved kinetic stability toward endoperoxide formation over the benchmark molecules **7 a** or **7 b**. Rather, compound **2 e**, bearing two Tr* groups, is the most reactive compound of the series in solution in the presence of light. While Diels‐Alder cycloaddition reactions are not observed in solutions of the acceptor PCBM with either **2 a** and **2 e**, the Tr* groups are inefficient toward preventing reactions in films formed from these solutions, i. e., in the solid state. Alternatively, electronic effects have been identified as the major contributor to the stability of 6,13‐ethynylated pentacenes through the complementary investigation of octafluorinated derivatives **2 e(F_8_)**, **7 b(F_8_)**, and **7 c(F_8_)**. These studies demonstrate that stability is correlated predominantly to electronic factors (via increased fluorination), and the half‐lives of the fluorinated pentacene derivatives **2 e(F_8_)** (*t*_1/2_=95.7 h) and **7 c(F_8_)** (*t*_1/2_=271 h) are enhanced by approximately an order of magnitude versus the non‐fluorinated analogs **2 e** (*t*_1/2_=7.1 h) and **7 a** (*t*_1/2_=24.3 h).

The exponential relationship between stability (half‐lives) and fluorination suggests that further fluorination might yield pentacene derivatives that are indefinitely photostable in solution in the presence of O_2_. Likewise, these studies highlight a key takeaway, that no one factor dictates stability, such as, for example, HOMO energy levels. Rather, the results from the present series of molecules, in conjunction with a meta‐analysis of complementary investigations by Yeates, Miller, Linker, Anthony, and their respective co‐workers, suggest that consideration for the delicate interplay between all FMO energy levels is essential when attempting to stabilize acenes towards O_2_. Overall, these trends and lifetimes can be utilized to design, synthesize, and manipulate acenes in the laboratory setting.

## Conflict of Interests

The authors declare no conflict of interest.

1

## Supporting information

As a service to our authors and readers, this journal provides supporting information supplied by the authors. Such materials are peer reviewed and may be re‐organized for online delivery, but are not copy‐edited or typeset. Technical support issues arising from supporting information (other than missing files) should be addressed to the authors.

Supporting Information

## Data Availability

The data that support the findings of this study are available in the supplementary material of this article.
