# Supplementary material for: Sterically Hindered Derivatives of Pentacene and Octafluoropentacene
Source: Chemistry. 2024 Oct 25;30(69):e202402651. doi: 10.1002/chem.202402651 (PMC11632412; doi:10.1002/chem.202402651)
Supplement: Supplementary file 1 — Supporting Information [file CHEM-30-e202402651-s001.pdf]

Chemistry–A European Journal

Supporting Information

Sterically Hindered Derivatives of Pentacene and Octafluoropentacene

Zachary W. Schroeder, Parisa Rezghi Rami, Matthias Adam, Michael J. Ferguson, Frank Hampel, and Rik R. Tykwinski*

Table of Content for the Supporting Information

General Information..2

Synthetic procedures and characterization of **2a–e**, **3c–d**, **3b(F8)**, **5a–e, 5b(F8), 5e(F8), 7b–c(F8)** 4

Photochemical Stability Studies..23

Preliminary Photochemical Stabilities in CH_2Cl_2..25

Photochemical Stability Studies of **2a**, **2b**, **2e**, **2e(F8)**, **7a**, **7b(F8)**, and **7c(F8)** in THF..29

Binary Film Stability Studies of **2a** and **2e** Mixed with **PCBM**..53

^{1}H, ^{19}F, and ^{13}C NMR Spectra of Compounds **2a**–**e**, **3c**–**d**, **3b(F8)**, **5a**–**e**, **5b(F8)**, **5e(F8)**, **2e(F8)**, **7b(F8)**, **7c(F8)**, and **7a**..63

Differential Scanning Calorimetry of compounds **2a**–**e**, **2e(F8)**, **7b(F8)**, and **7c(F8)**..115

Electrochemical redox potentials..129

References..130

General Information

All reagents were purchased in reagent grade from commercial suppliers and used without further purification. Compounds **3a**[1] and **3b**[2] were synthesized as described. Unless otherwise stated, all reactions were performed in standard, dry glassware under an inert atmosphere of N_2. THF and CH_2Cl_2 were dried under an atmosphere of N_2 in a commercial solvent purification system (LC Technology Solutions INC.). $MgSO_4$ was used after aq. workup as a drying agent. Saturated aq. NH_4Cl and brine refer to saturated aq. solution of NH_4Cl and NaCl, respectively. Evaporation and concentration in vacuo was performed on a rotary evaporator equipped with a water bath at a maximum temperature of 45 °C. Deoxygenation of solvents or solutions was accomplished by purging N_2 gas through the solvent or solution for at least 30 min. All solvent mixtures are v/v.

Column chromatography was performed using silica gel Si-60 M (Merck, 230–400 mesh). Thin Layer Chromatography (TLC) analysis was carried out on pre-coated plastic sheets covered with 0.20 mm silica gel and visualized with UV-light irradiation (254/364 nm). Melting points (mp) were measured with Thomas-Hoover "uni-melt" apparatus.

^{1}H and ^{13}C NMR spectra were recorded on an Agilent/Varian Mercury 400 (^{1}H: 400 MHz, ^{13}C: 100 MHz), a Agilent/Varian Inova four-channel 500 (^{1}H: 500 MHz, ^{13}C: 125 MHz), an Agilent/Varian VNMRS two-channel 500 MHz (^{1}H: 500 MHz, ^{13}C: 125 MHz), a Agilent VNMRS four-channel 700 (^{1}H: 700 MHz, ^{13}C: 175 MHz), a Jeol GX 400 (^{1}H: 400 MHz, ^{13}C: 100 MHz), a Jeol EX 400 (^{1}H: 400 MHz, ^{13}C: 100 MHz), a Agilent/Varian Inova three-channel 400 (^{1}H: 400 MHz, ^{19}F: 376 MHz, ^{13}C: 100 MHz), a Bruker Avance 300 (^{1}H: 300 MHz, ^{13}C: 75 MHz), and a Bruker Avance 400 (^{1}H: 400 MHz, ^{13}C: 100 MHz) spectrometer. NMR spectra were referenced to the residual solvent signal (^{1}H: $CDCl_3$: 7.26 ppm, THF-d_8: 1.72 or 3.58 ppm, ^{13}C: $CDCl_3$: 77.16 ppm, THF-d_8: 25.31 or 67.21 ppm) and recorded at ambient probe temperature. Coupling constants are reported as observed (±0.5 Hz).

UV-vis measurements were carried out on a Varian Cary 400 in CH_2Cl_2 at rt with quartz cuvettes having 1 cm path length. λ_{max} refers to the lowest energy wavelength of significant absorption (nm), ε refers to molar absorptivity (L mol^{-1} cm^{-1}).

IR spectra were recorded on a Thermo Nicolet 8700 FTIR spectrometer and continuum FTIR microscope as CH_2Cl_2 cast films.

For mass spectral analyses, low-resolution data were provided in cases when M^+ is not the base peak; otherwise, only high-resolution data are provided. MALDI HRMS were recorded on a Bruker 9.4T

Apex-Qe FTICR instrument and using the matrix *trans*-2-[3-(4-*tert*butylphenyl)-2-methyl-2-propenylidene] malononitrile (DCTB). ESI and APPI HRMS were obtained from an Agilent 6220 oaTOF.

Differential scanning calorimetry (DSC) measurements were measured on a Perkin Elmer Pyris 1 DSC or Mettler and Toledo Polymer DSC instruments. All thermal analyses were carried out under a flow of N_2 with a heating rate of 10 °C/min. Melting points from DSC analysis are reported as the endothermic maxima, except in cases when the sample decomposed, in which case the onset temperature of the decomposition exothermic peak is reported, as well as the exothermic maxima corresponding to the decomposition.

X-ray crystallographic data for compounds **2a**, **2c**, **2d**, **3a**, **5b**, **5c, 2e(F$_8$)**, and **7c(F$_8$)** were measured from the X-ray Crystallographic Laboratory, Department of Chemistry, University of Alberta, Edmonton, Alberta, T6G 2G2, Canada (michael.ferguson@ualberta.ca). All analyses were done on a Bruker PLATFORM/SMART 1000 CCD X-ray diffractometer. X-ray crystallographic data for compounds **2b** and **2e** were measured from the X-ray Crystallographic Laboratory, Department of Chemistry, FAU Erlangen-Nürnberg, Erlangen 91054 (contact frank.hampel@fau.de). All analysis was done on an Agilent SuperNova diffractometer with Atlas detector.

Synthetic procedures and characterization of 2a–e, 3c–d, 3b(F8), 5a–e, 5b(F8), 5e(F8), 7b–c(F8)

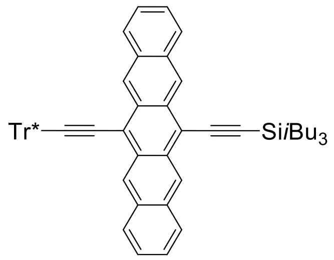

Compound 2a. To a solution of **5a** (350 mg, 0.303 mmol) in dry THF (10 mL) was added $SnCl_2$•$2H_2O$ (684 mg, 3.03 mmol) followed by 10% aq. H_2SO_4 (1.5 mL) under an atmosphere of N_2. The flask was wrapped in aluminum foil to limit light exposure, and the solution was stirred at rt for 20 h. The solution was poured onto a pad of silica gel, eluted with CH_2Cl_2 (20 mL), and the solvent was removed in vacuo. Column chromatography (silica gel, hexanes/CH_2Cl_2 1:1) afforded **2a** (250 mg, 74%) as a deep blue solid. Mp = 250–256 °C. R_f = 0.92 (silica gel, hexanes/CH_2Cl_2 1:1). UV-Vis (CH_2Cl_2) λ_{max} (ε) 270 (43100), 299 (sh, 89900), 310 (315000), 327 (41700), 351 (10300), 415 (2120), 439 (2640), 550 (5250), 591 (15200), 644 (30200) nm. IR (CH_2Cl_2, cast film) 3051 (w), 2953 (s), 2902 (s), 2867 (s), 2128 (m), 1592 (m) cm^{-1}. ^1H NMR (500 MHz, $CDCl_3$) δ 9.28 (s, 2H), 9.16 (s, 2H), 7.97 (d, *J* = 10.0 Hz, 2H), 7.75 (d, *J* = 9.5 Hz, 2H), 7.45 (d, *J* = 1.5 Hz, 6H), 7.43–7.38 (m, 5H), 7.35 (t, *J* = 7.5 Hz, 2H), 2.23 (app nonet, *J* = 6.5 Hz, 3H), 1.27 (s, 54H), 1.23 (d, *J* = 6.5 Hz, 18H), 0.99 (d, *J* = 6.5 Hz, 6H). ^{13}C NMR (125 MHz, $CDCl_3$) δ 150.2, 145.4, 132.4, 132.1, 131.0, 130.8, 128.9, 128.6, 126.6, 126.2, 126.0, 125.6, 124.2, 120.3, 119.7, 117.5, 112.7, 109.3, 105.1, 84.0, 58.5, 35.1, 31.7, 26.7, 25.65, 25.62. APPI HRMS *m/z* calcd for $C_{81}H_{103}Si$ ($[M + H]^+$) 1103.7824, found 1103.7804. DSC: Mp = 263 °C, decomposition, 263 (onset), 265 °C (peak).

A crystal of **2a** suitable for X-ray crystallographic analysis has been grown at 10 °C by slow evaporation of a CH_2Cl_2 solution layered with MeOH. X-ray data for **2a** ($C_{81}H_{102}Si$), F_w = 1103.71; monoclinic crystal system; space group $P2_1/c$; a = 19.0251(7) Å, b = 16.9209(6) Å, c = 44.1018(17) Å; α = 92.021(2)°, β = 90°, γ = 90°; V = 14188.5(9) Å^3; Z = 8; $\rho_{(calcd)}$ = 1.033 g/cm^3; $2\theta_{max}$ = 140.45°; μ =

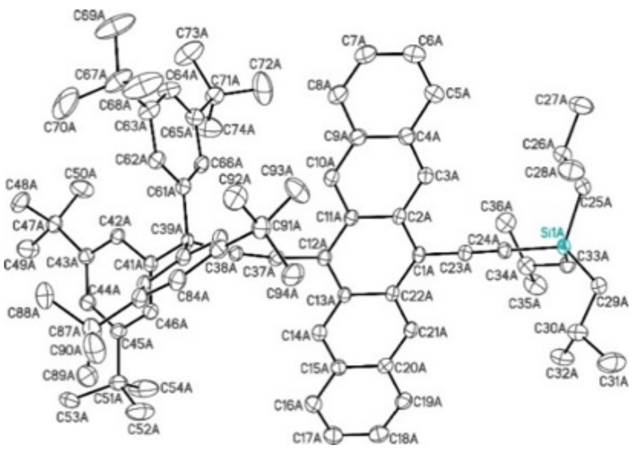

0.582 mm^{-1}; T = 173.15 K; total data collected = 89687; R_1 = 0.1073 [21275 observed reflections with $I \geq 2\sigma(I)$]; ωR_2 = 0.2828 for 26987 data, 1607 variables, and 162 restraints; largest difference, peak and hole = 1.000 and –0.479 e Å^{-3}.

The C–C distances within one of the disordered 2-methylpropyl groups (C25B to C28B; C25C to C28C) were restrained to be approximately the same by use of the SHELXL SADI instruction. The C–C distances within the minor orientation of one of the disordered tert-butyl groups (C91A, C92C to C94C) were similarly restrained. Finally, the rigid-bond restraint (RIGU) was applied to one of the tert-butyl groups (C71A to C74C) and to all the disordered 2-methylpropyl groups (C25B to C36C). CCDC 2240556.

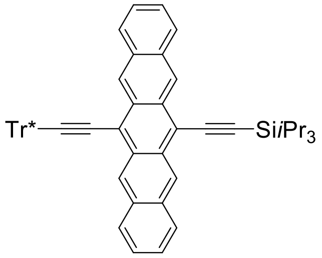

Compound 2b. To the solution of **5b** (230 mg, 0.207 mmol) in dry THF (12 mL) was added $SnCl_2$•$2H_2O$ (135 mg, 0.598 mmol) followed by 10% aq. H_2SO_4 (0.1 mL) under an atmosphere of N_2. The flask was wrapped in aluminum foil to limit light exposure, and the solution was stirred at rt for 17 h. The solution was poured onto a pad of silica gel, eluted with hexanes/CH_2Cl_2 5:1, and the solvent was removed in vacuo. Column chromatography (silica gel, hexanes/CH_2Cl_2 9:1) afforded **2b** (150 mg, 68%) as a deep blue solid. Mp = 294–296 °C (decomposition, dark blue to dark green color change). R_f = 0.37 (silica gel, hexanes/CH_2Cl_2 9:1). UV-Vis (CH_2Cl_2) λ_{max} (ε) 270 (47600), 298 (sh, 85400), 310 (278000), 327 (sh, 44400), 351 (21200), 414 (7320), 439 (8220), 547 (8310), 592 (16300), 642 (27700) nm. IR (CH_2Cl_2, cast film) 3050 (w), 2963 (s), 2865 (s), 2130 (m), 1592 (m) cm^{-1}. ^{1}H NMR (700 MHz, $CDCl_3$) δ 9.29 (s, 2H), 9.15 (s, 2H), 7.95 (d, J = 8.4 Hz, 2H), 7.72 (d, J = 8.4 Hz, 2H), 7.42 (d, J = 2.1 Hz, 6H), 7.38–7.36 (m, 5H), 7.33–7.31 (t, J = 8.4 Hz, 2H), 1.43–1.33 (m, 21H), 1.25 (s, 54H). ^{13}C NMR (175 MHz, $CDCl_3$) δ 150.2, 145.4, 132.4, 132.2, 131.0, 130.8, 128.9, 128.8, 126.6, 126.3, 126.0, 125.6, 124.2, 120.3, 119.7, 117.5, 112.8, 106.6, 105.1, 84.0, 58.5, 35.1, 31.7, 19.2, 11.9. MALDI HRMS (DCTB) *m/z* calcd for $C_{78}H_{96}Si$ (M^+) 1060.7276, found 1060.7258. DSC: Mp = 317 °C, decomposition, 317 (onset), 319 °C (peak).

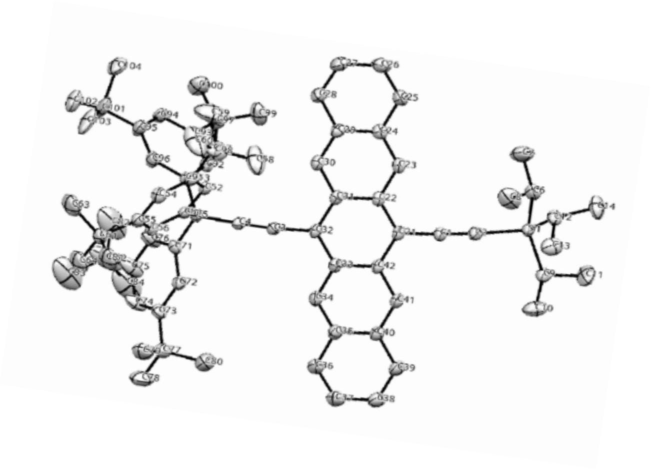

A crystal of **2b** suitable for X-ray crystallographic analysis has been grown at 4 °C by slow evaporation of a CH_2Cl_2 solution layered with hexanes. X-ray data for **2b** ($C_{78}H_{96}Si{\bullet}CH_2Cl_2$), F_w = 1146.56; monoclinic crystal system; space group $P2_1/n$; a = 18.0377(4) Å, b = 14.1188(2) Å, c = 28.1271(5) Å; α = 90.00°, β = 100.7166(19)°, γ = 90.00°; V = 7038.2(2) Å^3; Z = 4; $\rho_{(calcd)}$ = 1.082 g/cm^3; 2θ$_{max}$ = 123.16°; μ = 1.285 mm^{-1}; T = 172.95 K; total data collected = 14108; R_1 = 0.0698 [7622 observed reflections with $I \geq 2\sigma(I)$]; ωR_2 = 0.1909 for 8837 data, 796 variables, and 45 restraints; largest difference, peak and hole = 0.70 and –0.78 e Å^{-3}.

The three disordered *tert*-butyl groups were refined with the following occupancies: C62/63/64:C65/66/67 = 54:46, C82/83/84:C85/86/87 = 52:48, C102/103/104:C105/106/107 = 51:49. CCDC 2268220.

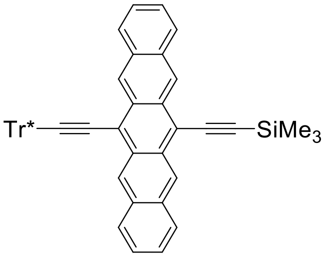

Compound 2c. To a solution of **5c** (145 mg, 0.139 mmol) in dry THF (10 mL) was added $SnCl_2{\bullet}2H_2O$ (110 mg, 0.487 mmol) under an atmosphere of N_2. The flask was wrapped in aluminum foil to limit light exposure, and the solution was stirred at rt for 6 h before being poured into H_2O (40 mL). The mixture was extracted with CH_2Cl_2 (3 x 30 mL), and the organic phase was washed with brine (50 mL), dried ($MgSO_4$), and the solvent removed in vacuo. Column chromatography (silica gel, CH_2Cl_2/hexanes 1:9) and removal of solvent in vacuo yielded **2c** (120 mg, 88%) as a dark blue solid. Mp = 190–196 °C. R_f = 0.34 (silica gel, hexanes/CH_2Cl_2 9:1). UV-Vis (CH_2Cl_2) λ_{max} (ε) 271 (57900), 298 (sh, 83300), 310 (237000), 326 (sh, 40700), 351 (13200), 413 (4860), 439 (5690), 545 (5890), 591 (12200), 642 (20600) nm. IR (CH_2Cl_2, cast film) 3051 (vw), 2963 (s), 2904 (m), 2867 (m), 2131 (w), 1592 (m) cm^{-1}. ^{1}H NMR (400 MHz, $CDCl_3$) δ 9.19 (s, 2H), 9.14 (s, 2H), 8.01 (d, J = 8.5 Hz, 2H), 7.72 (d, J = 8.5 Hz, 2H), 7.43 (d, J = 1.8 Hz, 6H), 7.40–7.38 (m, 2H), 7.37 (t, J = 1.8 Hz, 3H), 7.34–7.31 (m, 2H),

1.25 (s, 54H), 0.53 (s, 9H). ^{1}H NMR (400 MHz, THF-d_8) δ 9.22 (s, 2H), 9.17 (s, 2H), 8.02 (d, J = 8.5 Hz, 2H), 7.75 (d, J = 8.5 Hz, 2H), 7.50 (d, J = 1.8 Hz, 6H), 7.44 (t, J = 1.8 Hz, 3H), 7.41–7.33 (m, 4H), 1.26 (s, 54H), 0.53 (s, 9H). ^{13}C NMR (100 MHz, $CDCl_3$) δ 150.2, 145.4, 132.4, 132.2, 130.8, 130.7, 128.9, 128.8, 126.6, 126.1, 126.0, 125.6, 124.2, 124.1, 120.3, 84.1, 58.6, 35.1, 31.7, 0.5 (four signals coincident or not observed). ^{13}C NMR (100 MHz, THF-d_8) δ 150.7, 146.2, 133.2, 133.0, 131.3, 131.2, 129.2, 126.8, 126.6, 126.5, 124.7, 120.8, 59.3, 35.5, 31.8, 0.1 (eight signals coincident or not observed). MALDI HRMS (DCTB) m/z calcd for $C_{72}H_{84}Si$ (M^+) 976.6337, found 976.6334. DSC: Mp = 198 °C, decomposition, 204 °C (onset) 209 °C (peak).

A crystal of **2c** suitable for X-ray crystallographic analysis has been grown at 10 °C by slow evaporation of a CH_2Cl_2 solution layered with CH_3CN. X-ray data for **2c** ($C_{72}H_{84}Si$), F_w = 977.48; triclinic crystal system; space group $P\bar{1}$ (No. 2); a = 10.9538(9) Å, b = 15.9701(13) Å, c = 18.0909(15) Å; α = 95.5891(15)°, β = 90.3445(15)°, γ = 96.1645(15)°; V = 3131.0(4) Å^3; Z = 2; $\rho_{(calcd)}$ = 1.037 g/cm^3; $2\theta_{max}$ = 53.55°; μ = 0.076 mm^{-1}; T = 173.15 K; total data collected = 53593; R_1 = 0.0479 [9549 observed reflections with $I \geq 2\sigma(I)$]; ωR_2 = 0.1347 for 13320 data, 671 variables, and 52 restraints; largest difference, peak and hole = 0.363 and –0.272 e Å^{-3}.

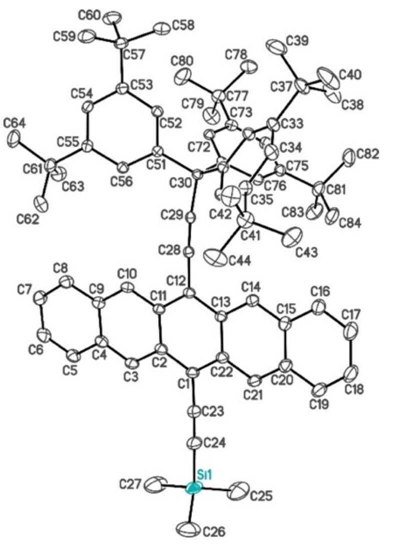

The rigid-bond restraint (**RIGU**) was applied to the atoms of the disordered trimethylsilyl group. CCDC 2240559.

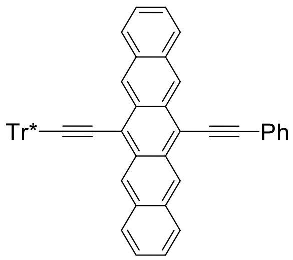

Compound 2d. To a solution of **5d** (80 mg, 0.078 mmol) in dry THF (12 mL) was added $SnCl_2$•$2H_2O$ (176 mg, 0.780 mmol) followed by 10% aq. H_2SO_4 (1 mL) under an atmosphere of N_2. The flask was wrapped in aluminum foil to limit light exposure, and the solution was stirred at rt for 24 h. The solution was poured onto a pad of silica gel, eluted with CH_2Cl_2 (20 mL), and the solvent was removed in vacuo. Column chromatography (silica gel, hexanes/CH_2Cl_2 1:3) afforded **2d** (75 mg, 98%) as a deep blue solid. Mp = 115—120 °C (decomp, observed dark blue to dark green color change). R_f = 0.78

(silica gel, hexanes/CH_2Cl_2 1:1). UV-Vis (CH_2Cl_2) λ_{max} (ε) 272 (16600), 300 (sh, 65700), 311 (291000), 337 (16500), 357 (22000), 440 (1540), 555 (3660), 599 (11600), 652 (21200) nm. IR (CH_2Cl_2, cast film) 3050 (w), 2963 (s), 2904 (m), 2867 (m), 2191 (vw), 1592 (m) cm^{-1}. ^1H NMR (400 MHz, $CDCl_3$) δ 9.28 (s, 2H), 9.16 (s, 2H), 8.04 (d, J = 8.5 Hz, 2H), 7.92–7.90 (m, 2H), 7.74 (d, J = 8.5 Hz, 2H), 7.55–7.47 (m, 3H), 7.44 (d, J = 1.7 Hz, 6H), 7.41–7.38 (m, 2H), 7.37 (t, J = 1.7 Hz, 3H), 7.35–7.31 (m, 2H) 1.25 (s, 54H). APPI HRMS m/z calcd for $C_{75}H_{81}$ ($[M + H]^+$) 981.6333, found 981.6321. DSC: decomposition, 109 (onset), 135 °C (peak).

A crystal of **2d** suitable for X-ray crystallographic analysis has been grown at 10 °C by slow evaporation of a CH_2Cl_2 solution layered with hexanes. X-ray data for **2d** ($C_{75}H_{80}$•C_6H_{14}), F_w = 1067.56; triclinic crystal system; space group P–1 (No. 2); a = 13.1218(12) Å, b = 13.6776(13) Å, c = 20.3552(19) Å; α = 97.6201(17)°, β = 91.3622(17)°, γ = 113.4363(16)°; V = 3310.5(5) Å^3; Z = 2; $\rho_{(calcd)}$ = 1.071 g/cm^3; 2θ$_{max}$ = 51.51°; μ = 0.060 mm^{-1}; T = 193.15 K; total data collected = 25092; R_1 = 0.0505 [7917 observed reflections with $I \geq 2\sigma(I)$]; ωR_2 = 0.1486 for 12585 data, 692 variables, and 0 restraints; largest difference, peak and hole = 0.274 and –0.211 e Å^{-3}. CCDC 2240553.

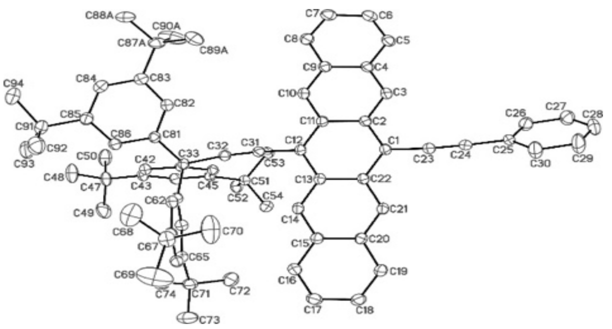

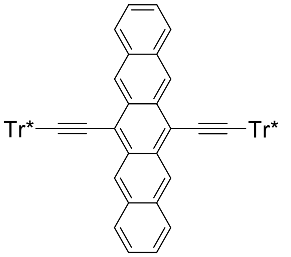

Compound 2e. To the solution of **5e** (200 mg, 0.130 mmol) in dry THF (8 mL) that had been purged with N_2 for 10 min was added $SnCl_2$•$2H_2O$ (350 mg, 1.55 mmol) followed by 10% aq. H_2SO_4 (1.75 mL). The flask was wrapped in aluminum foil to limit light exposure, and the solution was stirred at rt for 16 h. The solution was poured onto a pad of silica gel, eluted with hexanes/CH_2Cl_2 (5:1), and the solvent was removed in vacuo. Column chromatography (silica gel, hexanes/CH_2Cl_2 1:9) afforded **2e** (140 mg, 73%) as a deep blue solid. Mp = 300—304 °C (Partial decomposition apparent by TLC analysis after cooling). R_f = 0.45 (silica gel, hexanes/CH_2Cl_2 9:1). UV-vis (CH_2Cl_2) λ_{max} (ε) 269 (29200), 300 (sh, 80200), 310 (268000), 326 (sh, 33100), 353 (8770), 415 (1410), 438 (1840), 548 (5160),

591 (15400), 642 (30800) nm. IR (CH_2Cl_2, cast film) 3054 (w), 2962 (s), 2904 (m), 2867 (m), 1591 (m) cm^{-1}. ^1H NMR (700 MHz, THF-d_8) δ 9.16 (s, 4H), 7.74–7.72 (m, 4H), 7.52 (d, J = 1.8 Hz, 12H), 7.44 (t, J = 1.8 Hz, 6H), 7.32–7.30 (m, 4H), 1.26 (s, 108H). ^{13}C NMR (126 MHz, $CDCl_3$) δ 150.1, 145.5, 132.1, 131.0, 128.9, 126.4, 125.4, 124.1, 120.2, 118.6, 111.9, 84.4, 58.5, 35.1, 31.7. ^{13}C NMR (175 MHz, THF-d_8) δ 150.8, 146.3, 133.0, 131.5, 129.3, 126.9, 126.6, 126.3, 124.7, 120.8, 35.5, 31.7 (three signals coincident or not observed). MALDI HRMS (DCTB) *m/z* calcd for $C_{112}H_{138}$ (M^+) 1483.0793, found 1483.0779. DSC: Mp = 321 °C, decomposition, 319 °C (onset), 321 °C (peak).

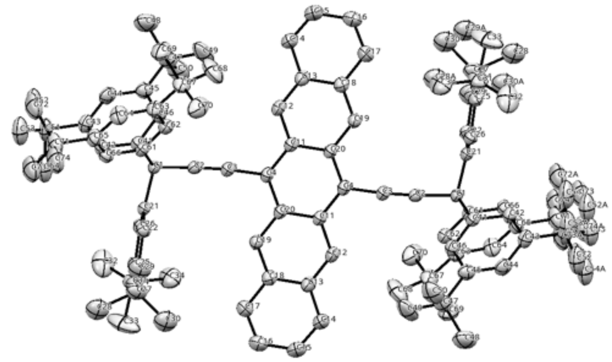

A crystal of **2e** suitable for X-ray crystallographic analysis has been grown at 4 °C by slow evaporation of a CH_2Cl_2 solution layered with hexanes. X-ray data for **2e** ($C_{112}H_{138}$•$2CH_2Cl_2$), F_w = 1654.07; triclinic crystal system; space group P–1; a = 13.7545(5) Å, b = 20.7623(4) Å, c = 22.0601(7) Å; α = 80.869(2)°, β = 81.684(3)°, γ = 74.585(2)°; V = 5961.4(3) $Å^3$; Z = 2; $\rho_{(calcd)}$ = 0.921 g/cm^3; $2\theta_{max}$ = 123.62°; μ = 1.184 mm^{-1}; T = 172.9 K; total data collected = 29992; R_1 = 0.0803 [13990 observed reflections with $I \geq 2\sigma(I)$]; ωR_2 = 0.2351 for 17957 data, 1109 variables, and 55 restraints; largest difference, peak and hole = 0.832 and –0.681 e $Å^{-3}$.

The four disordered *tert*-butyl groups were refined with the following occupancies: C28/29/30:C28a/29a/30a = 74:26, C52/53/54:C52a/53a/54a = 73:27, C72/73/74:C72a/73a/74a = 63:37, C52b/53b/54b:C52c/53c/54c = 41:59. CCDC 2268221.

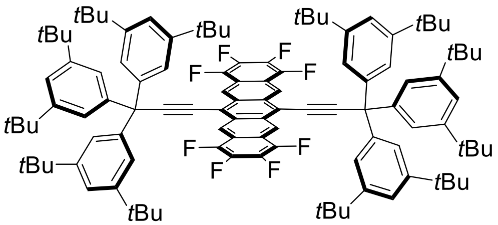

Compound 2e(F₈). The diol (61.0 mg, 0.038 mmol) was dissolved in N_2 saturated THF/MeOH (2, 5 mL) and nitrogen gas was bubbled through the solution continuously throughout the setup (10 min). $SnCl_2$•$2H_2O$ (140 mg, 0.62 mmol) was added at once followed by 10% aq. HCl (0.3 mL) and the reaction was stirred at reflux. After 21 h the reaction was cooled and poured over cold MeOH (50 mL). The blue precipitate was collected via suction filtration and washed with H_2O/MeOH (1:1, 5 x

10 mL). The crude blue solid was purified by column chromatography (silica gel, hexanes) and a subsequent recrystallization from hot acetone cooled slowly to –30 °C to afford **2e(F$_8$)** (56 mg, 25% over two steps) as a crystalline blue solid after suction filtration. Mp = no visible phase change ≤ 300 °C. R_f = 0.72 (silica gel, CH_2Cl_2/hexanes 1:9). UV-vis (CH_2Cl_2) λ_{max} (ε) 307 (205000), 541 (5550), 586 (15100), 635 (25400). IR (CH_2Cl_2 cast film) 3073 (w), 2966 (s), 2905 (m), 2869 (m), 2217 (w), 1590 (s) cm^{-1}. ^{1}H NMR (400 MHz, $CDCl_3$) δ 9.41 (s, 4H), 7.34 (s, 18H), 1.20 (s, 108H). ^{13}C {^{1}H, ^{19}F} NMR (100 MHz, $CDCl_3$) δ 150.3, 145.0, 141.5, 137.4, 131.0, 124.0, 120.8, 120.5, 119.7, 115.1, 82.8, 58.6, 35.0, 31.5 (one signal coincident or not observed). ^{19}F NMR (376 MHz, $CDCl_3$) δ –149.0 to –149.2 (m, 4F), –158.8 to –158.9 (m, 4F). MALDI HRMS (DCTB) m/z calcd for $C_{112}H_{130}F_8$ (M$^+$) 1627.0039, found 1627.0050. TGA: Td ≈ 405 °C. DSC: Mp = 305 °C, decomposition, 301 °C (onset), 305 °C (peak).

A crystal of **2e(F$_8$)** suitable for X-ray crystallographic analysis was grown at 23 °C by slow evaporation from $CHCl_3$/MeOH. X-ray data for **2e(F$_8$)** ($C_{112}H_{130}F_8$•2$CHCl_3$), F_w = 1866.89; monoclinic crystal system; crystal dimensions 0.29 x 0.13 x 0.05 mm; space group P2$_1$/n (an alternate setting of $P2_1/c$ [No. 14]); a = 18.799(2) Å, b = 11.0530(14) Å, c = 26.032(3) Å; α = 90°, β = 103.8514(19)°, γ = 90°; V = 5251.7(11) Å^3; Z = 2; $\rho_{(calcd)}$ = 1.181 g/cm^3; 2θ_{max} = 51.47°; μ = 0.224 mm^{-1}; T = 173 K; total data collected = 78274; R_1 = 0.0575 [5811 observed reflections with $I \geq 2\sigma(I)$]; wR_2 = 0.1593 for 10028 data, 19 restraints, and 632 variables; largest difference, peak and hole = 0.450 and –0.560 e Å^{-3}.

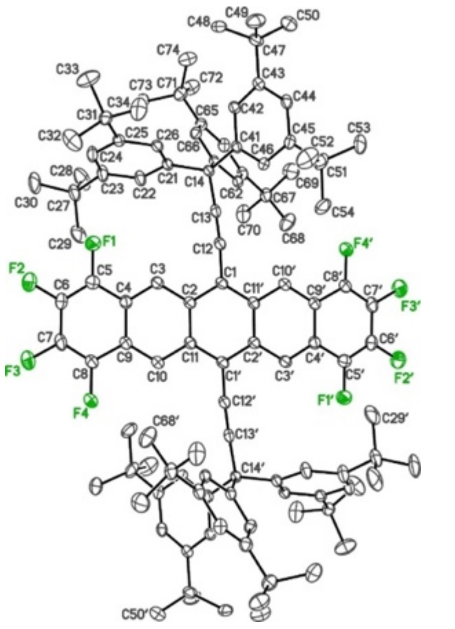

The C–C distances within the minor orientation of the disordered tert-butyl groups were restrained to be approximately the same by use of the *SHELXL* **SADI** instruction. Additionally, the C23–C27 and C23–C27A distances were similarly restrained. Finally, the C···C distances within the tert-butyl group defined by atoms (C27A–C30A) were similarly restrained. CCDC 2240558.

Compound 3a was synthesized as reported.[21] A crystal of **3a** suitable for X-ray crystallographic analysis have been grown at 4 °C by slow evaporation of a CH_2Cl_2 solution layered with MeOH. X-ray data for **3a** ($C_{37}H_{42}O_2Si$), F_w = 546.79; monoclinic crystal system; space group $P2_1/n$ (No. 14); a = 9.8249(3) Å, b = 20.4468(6) Å, c = 15.5149(5) Å; α = 90°, β = 98.1901(14)°, γ = 90°; V = 3084.97(16) Å^3; Z = 4; $\rho_{(calcd)}$ = 1.177 g/cm^3; 2θ$_{max}$ = 146.61°; μ = 0.899 mm^{-1}; T = 173.15 K; total data collected = 20600; R_1 = 0.0353 [5567 observed reflections with $I \geq 2\sigma(I)$]; ωR_2 = 0.0995 for 6165 data, 418 variables, and 0 restraints; largest difference, peak and hole = 0.232 and −0.229 e Å^{-3}. CCDC 2240557.

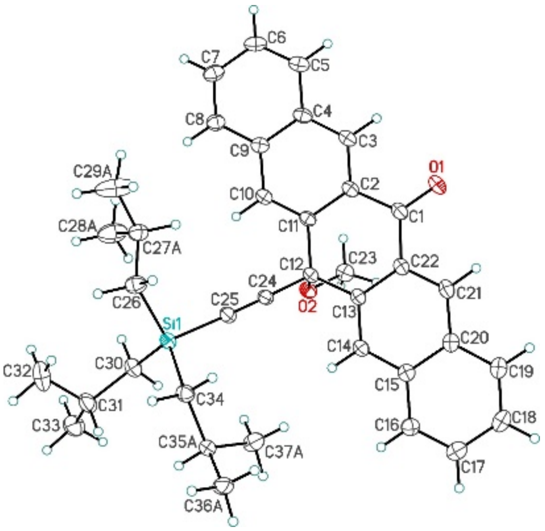

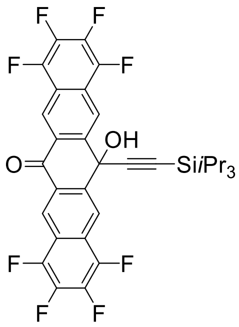

Compound 3b(F$_8$). To a solution of *i*Pr$_3$Si–C≡C–H (1.45 g, 1.78 mL, 7.96 mmol) in dry THF (10 mL) at −78 °C was added *n*-HexLi (2.88 mL, 2.3 M in hexanes, 6.63 mmol). The resulting solution was stirred for 30 min generating acetylide **4b**. The acetylide **4b** was then transferred slowly dropwise over ca. 20 minutes to a secondary flask containing a slurry of **6** (4.42 mmol, 2.00 g) in THF (40 mL) at −10 °C. Once the addition was complete the reaction was slowly warmed to rt overnight. After 12 h the reaction mixture was cooled to −78 °C and satd. aqueous NH_4Cl (40 mL) was added via syringe dropwise. The reaction mixture was warmed to rt and filtered to remove excess quinone. The insoluble material was extracted by washing with CH_2Cl_2 (4 x 15 mL) before the organic layer of the filtrate was separated. The aqueous phase was then extracted with CH_2Cl_2 (4 x 50 mL), and the combined organic extracts were washed with H_2O (60 mL), and brine (50 mL). The combined organic extract was dried over $MgSO_4$, gravity filtered, and concentrated *in vacuo*. The crude solid was triturated in hot hexanes (60 mL), cooled to −30 °C, and the remaining solid was isolated via suction filtration to yield crude ketone **3b(F$_8$)** (1.64 g, 59%; ca. 85% pure by ^{1}H NMR) as a pale-yellow solid.

The mother liquor contains **5b(F$_8$)** that can be isolated, see below. Mp = 261–262 °C (decomposes on melt, pale-yellow → black). R_f = 0.54 (silica gel, CH_2Cl_2). IR (CH_2Cl_2 cast film) 3400 (br m), 2960 (w), 2947 (w), 2866 (w), 1658 (s), 1456 (s) cm^{-1}. ^{1}H NMR (400 MHz, $CDCl_3$) δ 9.05 (s, 2H), 8.99 (s, 2H), 3.06 (s, 1H), 1.25–1.10 (m, 21H). ^{13}C {^{1}H, ^{19}F} NMR (100 MHz, $CDCl_3$) δ 182.6, 143.6, 142.6, 140.3, 140.1, 139.1, 129.8, 122.8, 121.8, 119.9, 119.6, 105.9, 93.4, 68.7, 18.7, 11.3. ^{19}F NMR (376 MHz, $CDCl_3$) δ –146.0 (t, J = 17.3 Hz, 2F), –148.2 (t, J = 17.7 Hz, 2F), –152.5 (t, J = 16.2 Hz, 2F), –155.2 (t, J = 18.4 Hz, 2F). ESI HRMS m/z calcd for $C_{33}H_{25}F_8O_2Si$ ([M–H]$^-$) 633.1503, found 633.1507. APPI HRMS m/z calcd for $C_{33}H_{25}F_8OSi$ ([M–OH]$^+$) 617.1541, found 617.1538.

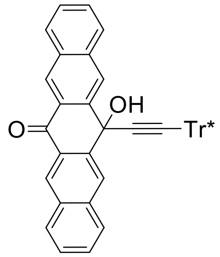

Compound 3c. To a solution of Tr*–C≡C–H (2.0 g, 3.3 mmol) in dry THF (35 mL) cooled to –78 °C was added *n*-BuLi (1.3 mL, 2.5 M in hexanes, 3.3 mmol) slowly under an atmosphere of N_2. The resulting solution was stirred for 1 h providing acetylide **4e**. The acetylide **4e** was transferred via cannula dropwise into a suspension of **PQ** (3.1 g, 10 mmol) in dry THF (35 mL) at –78 °C. After stirring the reaction mixture at rt for 12 h, it was quenched via the addition of satd. aq. NH_4Cl (50 mL). The unreacted **PQ** (900 mg, 2.92 mmol) was filtered and recovered. The filtrate was extracted with CH_2Cl_2 (3 x 50 mL). The combined organic phases were washed with brine (50 mL), dried over $MgSO_4$, filtered, and the solvent was removed in vacuo. Column chromatography (silica gel, CH_2Cl_2/hexanes 4:1) and removal of solvent in vacuo yielded **3c** (1.75 g, 58%) as a pale-orange solid. Mp = 108–110 °C (decomp, pale-orange → green color change). R_f = 0.28 (silica gel, hexanes/CH_2Cl_2 1:4). IR (CH_2Cl_2, cast film) 3415 (br, w), 3057 (w), 2963 (s), 2904 (m), 2868 (m), 2232 (vw), 1663 (m), 1628 (m), 1622 (m), 1592 (m) cm^{-1}. ^{1}H NMR (700 MHz, $CDCl_3$) δ 8.86 (s, 2H), 8.59 (s, 2H), 8.04 (d, J = 8.5 Hz, 2H), 7.78 (d, J = 8.5 Hz, 2H), 7.60–7.54 (m, 4H), 7.21 (t, J = 1.8 Hz, 3H), 6.93 (d, J = 1.8 Hz, 6H), 3.13 (s, 1H), 1.09 (s, 54H). ^{13}C NMR (175 MHz, $CDCl_3$) δ 184.2, 150.0, 144.7, 140.2, 136.1, 132.9, 129.9, 129.8, 128.8, 128.5, 128.4, 127.3, 127.0, 123.8, 120.0, 95.5, 86.6, 67.9, 56.7, 34.9, 31.5. MALDI MS (DCTB) m/z 935.6 ([M + Na]$^+$, 60), 912.6 (M$^+$, 20), 895.6 ([M – OH]$^+$, 100). MALDI HRMS (DCTB) m/z calcd for $C_{67}H_{76}O_2$ (M$^+$) 912.5840, found 912.5836. MALDI HRMS (DCTB) m/z calcd for $C_{67}H_{76}NaO_2$ ([M + Na]$^+$) 935.5738, found 935.5734.

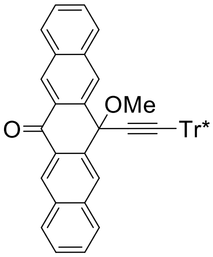

Compound 3d. To a solution of Tr*–C≡C–H (3.1 g, 5.1 mmol) in dry THF (50 mL) cooled to −78 °C was added *n*-BuLi (2.5 mL, 2.5 M in hexanes, 6.1 mmol) slowly (over 5 min) under an atmosphere of N_2. The resulting solution was stirred for 1 h providing acetylide **4e**. The acetylide **4e** was transferred via cannula dropwise into a suspension of **PQ** (2.2 g, 7.1 mmol) in dry THF (50 mL) at –78 °C. The reaction mixture was stirred at rt for 24 h. The solution was cooled to −78 °C, and MeI (1.30 mL, 20.9 mmol) was added slowly, and the reaction flask was wrapped in aluminum foil to limit light exposure. After stirring the reaction mixture at rt for 4 h, it was quenched via the addition of satd. aq. NH_4Cl (20 mL). The unreacted **PQ** (1.0 g, 3.2 mmol) was filtered and recovered. The filtrate was extracted with CH_2Cl_2 (3 x 50 mL). The combined organic phases were washed with brine (50 mL), dried over $MgSO_4$, filtered, and the solvent was removed in vacuo. Column chromatography (silica gel, CH_2Cl_2/hexanes 2:1 → 3:1) and removal of solvent in vacuo yielded **3d** (1.87 g, 39%) as a pale-orange solid and recovered Tr*–C≡C–H (1.20 g, 1.98 mmol), R_f = 0.81 (silica gel, CH_2Cl_2/hexanes 2:1). Mp = 204−206 °C. R_f = 0.40 (silica gel, hexanes/CH_2Cl_2 1:2). IR (CH_2Cl_2, cast film) 3057 (w), 2963 (s), 2904 (m), 2868 (m), 1677 (m), 1593 (m), 1273 (m) cm^{-1}. ^1H NMR (700 MHz, $CDCl_3$) δ 8.82 (s, 2H), 8.46 (s, 2H), 8.04–8.02 (m, 2H), 7.68–7.67 (m, 2H), 7.58–7.54 (m, 4H), 7.30 (t, *J* = 1.8 Hz, 3H), 7.21 (d, *J* = 1.8 Hz, 6H), 3.12 (s, 3H), 1.17 (s, 54H). ^{13}C NMR (175 MHz, $CDCl_3$) δ 185.2, 150.1, 144.9, 137.0, 135.3, 133.0, 129.9, 129.8, 129.6, 128.7, 128.6, 128.3, 127.4, 123.9, 120.2, 98.5, 83.3, 75.0, 57.2, 52.4, 35.0, 31.6. MALDI MS (DCTB) *m/z* 926.6 (M^+, 10), 895.6 ($[M-OCH_3]^+$, 100). MALDI HRMS (DCTB) *m/z* calcd for $C_{68}H_{78}O_2$ (M^+) 926.5996, found 926.5995.

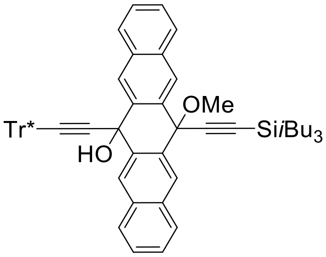

Compound 5a. To solution of Tr*–C≡C–H (1.66 g, 2.74 mmol) in dry THF (10 mL) at −78 °C under an atmosphere of N_2 was added dropwise *n*-BuLi (1.2 mL, 2.5 M in hexanes, 3.0 mmol). The resulting

solution was stirred for 5 min at –78 °C and 15 min at rt providing acetylide **4e**. The acetylide **4e** was added dropwise over 5 min to a solution of **3a** (500 mg, 0.914 mmol) in dry THF (10 mL) at –78 °C. The ice bath was removed, and the reaction mixture warmed to rt and stirred for 24 h. After quenching the reaction via the addition of satd. aq. NH_4Cl solution (15 mL), the resulting solution was extracted with CH_2Cl_2 (3 × 30 mL). The combined organic phases were washed with brine (30 mL), dried over $MgSO_4$, filtered, and the solvents removed in vacuo. Column chromatography (silica gel, CH_2Cl_2/hexanes 1:1) and removal of solvent in vacuo yielded **5a** (380 mg, 36%) as an off-white solid. Mp = 108–110 °C. R_f = 0.37 (silica gel, hexanes/CH_2Cl_2 1:1). IR (CH_2Cl_2, cast film) 3057 (w), 2954 (s), 2903 (m), 2868 (m), 2167 (vw), 1593 (m), 1249 (m) cm^{-1}. ^1H NMR (700 MHz, $CDCl_3$) δ 8.57 (s, 2H), 8.50 (s, 2H), 7.90–7.89 (m, 2H), 7.85–7.84 (m, 2H), 7.51–7.48 (m, 4H), 7.08 (t, *J* = 1.8 Hz, 3H), 6.76 (d, *J* = 1.8 Hz, 6H), 3.29 (s, 1H), 2.35 (s, 3H), 2.09 (app nonet, *J* = 6.7 Hz, 3H), 1.12 (d, *J* = 6.6 Hz, 18H), 0.98 (s, 54H), 0.84 (d, *J* = 6.9 Hz, 6H). ^{13}C NMR (175 MHz, $CDCl_3$) δ 149.6, 145.2, 139.2, 133.7, 132.9, 132.7, 128.23, 128.19, 128.0, 126.6, 126.3, 124.5, 124.1, 119.4, 105.2, 94.9, 91.6, 86.9, 67.8, 56.2, 50.8, 34.8, 31.3, 26.6, 25.4 (two signals coincident or not observed). MALDI MS (DCTB) *m/z* 1189.8 ([M + K]$^+$, 8), 1173.8 ([M + Na]$^+$, 36), 1150.8 (M^+, 61), 1133.8 ([M – OH]$^+$, 53), 1119.8 ([M – OCH_3]$^+$, 100). MALDI HRMS (DCTB) *m/z* calcd for $C_{82}H_{106}O_2Si$ (M^+) 1150.7957, found 1150.7954.

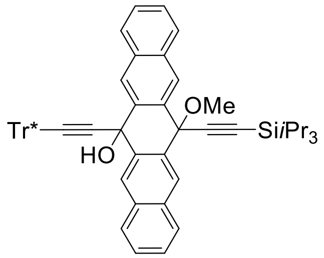

Compound 5b. To a solution of Tr*–C≡C–H (600 mg, 0.992 mmol) in dry THF (15 mL) at –78 °C under an atmosphere of N_2 was added *n*-BuLi (0.40 mL, 2.5 M in hexanes, 0.99 mmol). The resulting solution was stirred for 5 min at –78 °C and 50 min at rt providing acetylide **4e**. The acetylide **4e** added dropwise over 5 min to a solution of **3b** (200 mg, 0.792 mmol) in dry THF (7 mL) at –78 °C under an atmosphere of N_2. The reaction mixture was warmed to rt and stirred for 48 h. After quenching the reaction via the addition of H_2O (5 mL) and satd. aq. NH_4Cl solution (5 mL), the resulting solution was extracted with CH_2Cl_2 (3 × 30 mL). The combined organic phases were washed with brine (40 mL), dried over $MgSO_4$, filtered, and the solvents removed in vacuo. Column chromatography (silica gel, CH_2Cl_2/hexanes 1:3) and removal of solvent in vacuo yielded **5b** (200 mg, 45%) as a pure pale-green solid and recovered Tr*–C≡C–H (100 mg, 0.165 mmol, R_f = 0.95 (CH_2Cl_2 /hexanes 1:1)). Mp = 165–167 °C (decomp, pale green → dark green color change). R_f = 0.68 (silica

gel, CH_2Cl_2 /hexanes 1:1). IR (CH_2Cl_2, cast film) 3056 (w), 2962 (s), 2904 (m), 2866 (m), 2169 (vw), 1593 (m), 1248 (m) cm^{-1}. ^1H NMR (700 MHz, $CDCl_3$) δ 8.63 (s, 2H), 8.50 (s, 2H), 7.90−7.89 (m, 2H), 7.85−7.83 (m, 2H), 7.51−7.48 (m, 4H), 7.08 (t, J = 1.8 Hz, 3H), 6.76 (d, J = 1.8 Hz, 6H), 3.30 (s, 1H), 2.38 (s, 3H), 1.27−1.26 (m, 21 H), 0.98 (s, 54H). ^{13}C NMR (175 MHz, $CDCl_3$) δ 149.6, 145.1, 139.3, 133.7, 132.9, 132.6, 128.3, 128.2, 128.1, 126.6, 126.3, 124.4, 124.1, 119.4, 105.2, 101.3, 91.5, 86.9, 67.8, 66.5, 56.2, 50.7, 34.8, 31.3, 19.0, 11.6. MALDI MS (DCTB) m/z 1148.7 ($[M + K]^+$, 6), 1131.7 ($[M + Na]^+$, 58), 1108.7 (M^+, 61), 1091.7 ($[M - OH]^+$, 76), 1077.7 ($[M - OCH_3]^+$, 100), 1060.7 ($[M - OCH_3 - OH]^+$, 43). MALDI HRMS (DCTB) m/z calcd for $C_{79}H_{100}O_2Si$ (M^+) 1108.7487, found 1108.7478.

A crystal of **5b** suitable for X-ray crystallographic analysis has been grown at 10 °C by slow evaporation of a CH_2Cl_2 solution layered with MeOH. X-ray data for **5b** ($C_{79}H_{100}O_2Si \bullet 0.5CH_2Cl_2 \bullet 0.5CH_3OH$), F_w = 1168.16; triclinic crystal system; space group P–1 (No. 2); a = 10.7080(18) Å, b = 15.305(3) Å, c = 23.625(4) Å; α = 97.811(3)°, β = 102.562(3)°, γ = 97.301(3)°; V = 3694.6(11) $Å^3$; Z = 2; $\rho_{(calcd)}$ = 1.050 g/cm^3; $2\theta_{max}$ = 51.45°; μ = 0.111 mm^{-1}; T = 193.15 K; total data collected = 28003; R_1 = 0.0561 [8622 observed reflections with $I \geq 2\sigma(I)$]; ωR_2 = 0.1755 for 14050 data, 860 variables, and 209 restraints; largest difference, peak and hole = 0.395 and –0.249 e $Å^{-3}$.

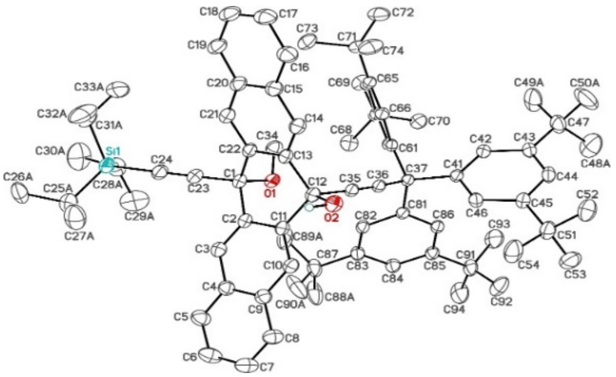

The C–C distances within the disordered *tert*-butyl groups were restrained to be approximately the same using *SHELEX* **SADI** instruction. Likewise, the Si–C, C–C, Si···C, and C···C distances were also restrained. Finally, the rigid-bond restraint (**RIGU**) was applied to the anisotropic displacement parameters of the atoms of the disordered groups (including Si1). CCDC 2240554.

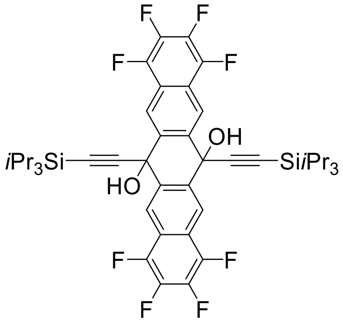

Compound 5b(F$_8$). The mother liquor (vide supra) was subsequently concentrated in vacuo and the residue was purified by column chromatography (silica gel, Et_2O/hexanes, 1:9) to afford a sample of the known diol **5b(F$_8$)**[3] Mp = 183—185 °C. R_f = 0.50 (silica gel, Et_2O/hexanes 1:10). IR (CH_2Cl_2 cast film) 3439 (br m), 2944 (m), 2891 (w), 2866 (m), 1508 (s), 1468 (s) cm^{-1}. ^1H NMR (500 MHz, $CDCl_3$) δ 8.90 (s, 4H), 3.52 (s, 2H), 1.20–1.10 (m, 42H). ^{13}C {^1H, ^{19}F} NMR (100 MHz, $CDCl_3$) δ 142.6, 138.8, 137.9, 119.7, 118.9, 107.8, 91.9, 69.5, 18.7, 11.3. ^{19}F NMR (376 MHz, $CDCl_3$) δ –148.8 to –149.1 (m, 4F), –156.8 to –157.2 (m, 4F). MALDI HRMS (DCTB) *m/z* calcd for $C_{44}H_{48}F_8NaO_2Si_2$ ($[M+Na]^+$) 839.2957, found 839.2958.

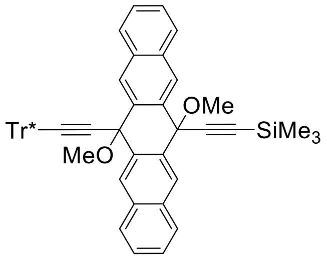

Compound 5c. To a solution of $Me_3Si–C≡C–H$ (2.80 mL, 19.7 mmol) in dry THF (15 mL) cooled to −78 °C was added *n*-BuLi (7.5 mL, 2.5 M in hexanes, 19 mmol) slowly under an atmosphere of N_2. The resulting solution was stirred for 5 min at −78 °C and 10 min rt providing acetylide **4c**. The acetylide **4c** was transferred via cannula dropwise into a solution of **3d** (4.5 g, 4.8 mmol) in dry THF (30 mL) at −78 °C under an atmosphere of N_2. The cooling bath was removed after stirring for 10 min. The solution was warmed to rt and stirred for 2 h under an atmosphere of N_2. The solution was cooled to −78 °C and MeI (3.0 mL, 48 mmol) was added slowly, and the reaction flask was wrapped in aluminum foil to limit light exposure. The solution was allowed to warm to rt and stir for 48 h. It was cooled to 0 °C and quenched via the addition of satd. aq. NH_4Cl (100 mL). H_2O (100 mL) was added, and the reaction mixture was extracted with CH_2Cl_2 (3 x 100 mL). The combined organic phases were washed with brine (100 mL), dried over $MgSO_4$, filtered, and the solvent was removed in vacuo. Column chromatography (silica gel, CH_2Cl_2/hexanes 1:1) and removal of solvent in vacuo yielded **5c** (4.0 g, 79%) as a bright green solid. Mp = 125–128 °C. R_f = 0.64 (silica gel, hexanes/CH_2Cl_2 1:1). IR (CH_2Cl_2, cast film) 3058 (w), 2962 (s), 2904 (m), 2868 (m), 2170 (vw), 1593 (m), 1250 (m) cm^{-1}. ^1H NMR (700 MHz, $CDCl_3$) δ 8.47 (s, 2H), 8.45 (s, 2H), 7.94 (d, *J* = 9.4 Hz, 2H), 7.70 (d, *J* = 9.4 Hz, 2H), 7.52–7.50 (m, 2H), 7.47–7.46 (m, 2H), 7.24 (t, *J* = 1.8 Hz, 3H), 7.10 (d, *J* = 1.8 Hz, 6H), 3.16 (s, 3H), 2.73 (s, 3H), 1.12 (s, 54H), 0.14 (s, 9H). ^{13}C NMR (175 MHz, $CDCl_3$) δ 149.9, 145.2, 134.9, 133.9, 133.5, 133.2, 128.5, 128.2, 127.9, 127.5, 126.6, 126.4, 124.0, 119.9, 106.8, 96.8, 91.4, 84.9, 75.2, 74.2, 57.0,

52.6, 51.4, 34.9, 31.5, 0.1. MALDI MS (DCTB) m/z 1038.7 (M^+, 5), 1007.6 ($[M - OCH_3]^+$, 100). MALDI HRMS (DCTB) m/z calcd for $C_{74}H_{90}O_2Si$ (M^+) 1038.6705, found 1038.6701.

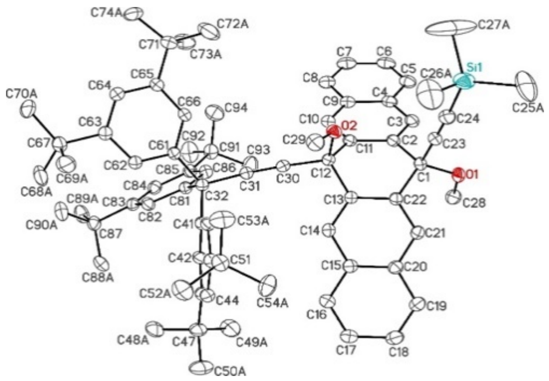

A crystal of **5c** suitable for X-ray crystallographic analysis has been grown at 10 °C by slow evaporation of a CH_2Cl_2 solution layered with MeOH. X-ray data for **5c** ($C_{74}H_{90}O_2Si$), F_w = 1039.62; monoclinic crystal system; space group $P2_1/n$; a = 18.9794(7) Å, b = 16.3771(6) Å, c = 20.6675(6) Å; β = 92.153(3)°; V = 6419.5(4) Å^3; Z = 4; $\rho_{(calcd)}$ = 1.076 g/cm^3; 2θ$_{max}$ = 140.37°; μ = 0.641 mm^{-1}; T = 173.15 K; total data collected = 35973; R_1 = 0.0659 [7638 observed reflections with $I \geq 2\sigma(I)$]; ωR_2 = 0.1873 for 12040 data, 904 variables, and 120 restraints; largest difference, peak and hole = 0.280 and –0.484 e Å^{-3}.

The Si–C distances within the disordered $SiMe_3$ group were restrained to be approximately the same by use of the *SHELXL* **SADI** instruction and the anisotropic displacement parameters of the group had the rigid bond restraint (**RIGU**) applied. The C–C distances within the disordered *tert*-butyl groups were also restrained by **SADI**. CCDC 2240555.

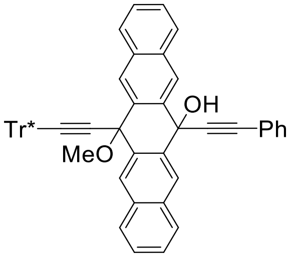

Compound 5d. To a solution of Ph–C≡C–H (0.31 mL, 2.8 mmol) in dry THF (10 mL) at –78 °C under an atmosphere of N_2 was added dropwise n-BuLi (1.1 mL, 2.5 M in hexanes, 2.7 mmol). The resulting solution was stirred for 5 min at −78 °C and 30 min at rt t providing acetylide **4d**. The acetylide **4d** was added dropwise over 5 min to a solution of **3d** (500 mg, 0.539 mmol) in dry THF (10 mL) at −78 °C. The reaction mixture was warmed to rt and stirred for 20 h. After quenching the reaction via the addition of satd. aq. NH_4Cl solution (30 mL), the resulting solution was extracted with CH_2Cl_2 (3 × 30 mL). The combined organic phases were washed with brine (30 mL), dried over $MgSO_4$, filtered, and the solvents removed in vacuo. Column chromatography (silica gel, hexanes/EtOAc 10:1) and removal of solvent in vacuo yielded **5d** (185 mg, 33%) as a pale-yellow solid. Mp = 125−130 °C (decomp., pale-yellow → pale-green color change). R_f = 0.55 (silica gel, hexanes/EtOAc 10:1). IR

(CH_2Cl_2, cast film) 3410 (br w), 3057 (w), 2963 (s), 2904 (m), 2867 (m), 2236 (vw), 1593 (m), 1248 (m) cm^{-1}. ^{1}H NMR (400 MHz, $CDCl_3$) δ 8.76 (s, 2H), 8.45 (s, 2H), 7.93 (d, *J* = 8.1 Hz, 2H), 7.80–7.78 (m, 2H), 7.56 (d, *J* = 8.1 Hz, 2H), 7.52–7.49 (m, 2H), 7.47–7.42 (m, 5H), 7.36 (t, *J* = 1.7 Hz, 3H), 7.33 (d, *J* = 1.7 Hz, 6H), 5.77 (s, 1H), 3.33 (s, 3H), 1.22 (s, 54H). ^{13}C NMR (100 MHz, $CDCl_3$) δ 154.5, 150.3, 144.8, 137.4, 133.9, 133.6, 132.6, 128.9, 128.63, 128.59, 128.5, 127.5, 127.1, 126.7, 123.9, 120.3, 78.9, 74.4, 72.3, 60.5, 57.5, 56.9, 52.4, 35.1, 31.6 (three signals coincident or not observed). ESI MS (CH_2Cl_2/CH_3OH) *m/z* 1051.6 ([M + Na]$^+$, 100), 1011.6 ([M − OH]$^+$, 8), 997.6 ([M − OCH_3]$^+$, 18). ESI HRMS (CH_2Cl_2/CH_3OH) *m/z* calcd for $C_{76}H_{84}NaO_2$ ([M + Na]$^+$) 1051.6364, found 1051.6346.

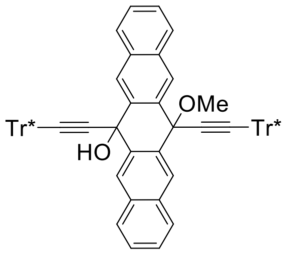

Compound 5e. To a solution of Tr*–C≡C–H (890 mg, 1.47 mmol) in dry THF (10 mL) at −78 °C under an atmosphere of N_2 was added dropwise *n*-BuLi (0.60 mL, 2.5 M in hexanes, 1.5 mmol). The resulting solution was stirred for 5 min at −78 °C and 1 h at rt providing acetylide **4e**. The acetylide **4e** was added dropwise over 5 min to a solution of **3d** (340 mg, 0.367 mmol) in dry THF (10 mL) at −78 °C under N_2. The reaction mixture was warmed to rt and stirred for 19 h. After quenching the reaction via the addition of satd. aq. NH_4Cl solution (10 mL), the resulting solution was extracted with CH_2Cl_2 (3 × 20 mL). The combined organic phases were washed with satd. aq. NH_4Cl solution (10 mL), brine (20 mL), dried over $MgSO_4$, filtered, and the solvents removed in vacuo. Column chromatography (silica gel, CH_2Cl_2/hexanes 1:1) and removal of solvent in vacuo recovered Tr*–C≡C–H (300 mg, 0.496 mmol, R_f = 0.95 (silica gel, CH_2Cl_2 /hexanes 1:1) and yielded **5e** as a white solid (300 mg, 53%) containing approximately 10% unknown/unidentified impurity. Mp = 130–132 °C. R_f = 0.40 (silica gel, hexanes/CH_2Cl_2 1:1). IR (CH_2Cl_2, cast film) 3052 (w), 2963 (s), 2905 (m), 2868 (m), 1593 (m), 1248 (m) cm^{-1}. ^{1}H NMR (700 MHz, $CDCl_3$) δ 9.00 (s, 2H), 8.89 (s, 2H), 8.35 (bs, 2H), 8.02 (bs, 2H), 7.92–7.43 (m, 22H), 3.76 (s, 1H), 2.88 (s, 3H), 1.68 (s, 54H), 1.46 (s, 54H). ^{1}H NMR (700 MHz, THF-d_8) δ 8.62 (s, 2H), 8.45 (s, 2H), 7.90 (d, *J* = 8.1 Hz, 2H), 7.54 (d, *J* = 8.1 Hz, 2H), 7.43–7.41 (m, 2H), 7.38 (t, *J* = 1.8 Hz, 3H), 7.37–7.35 (m, 8H), 7.08 (t, *J* = 1.8 Hz, 3H), 6.80 (d, *J* = 1.8 Hz, 6H), 5.73 (s, 1H), 2.39 (s, 3H), 1.19 (s, 54H), 0.95 (s, 54H). ^{13}C NMR (175 MHz, $CDCl_3$) δ 150.0, 149.8, 149.6, 145.3, 139.3, 133.9, 133.7, 132.7, 128.5, 128.2, 128.1, 126.4, 125.9, 124.6, 124.1, 124.0, 120.1, 119.4, 98.0, 91.6, 87.1, 84.2, 67.9, 57.4, 56.2, 51.1, 35.2, 34.8, 31.7, 31.3 (one signal coincident or not observed). ^{13}C NMR (175

MHz, THF-d_8) δ 150.6, 149.9, 146.5, 146.1, 141.9, 134.6, 134.5, 133.2, 128.9, 128.5, 128.3, 126.8, 126.1, 125.7, 124.8, 124.6, 120.6, 119.6, 84.8, 77.8, 58.0, 56.8, 51.4, 35.4, 35.1, 31.7, 31.4 (four signals coincident or not observed). MALDI MS (DCTB) *m/z* 1531.1 (M^+, 94), 1514.1 ($[M - OH]^+$, 71), 1500.1 ($[M - OCH_3]^+$, 100). MALDI HRMS (DCTB) *m/z* calcd for $C_{113}H_{142}O_2$ (M^+) 1531.1004, found 1531.0994.

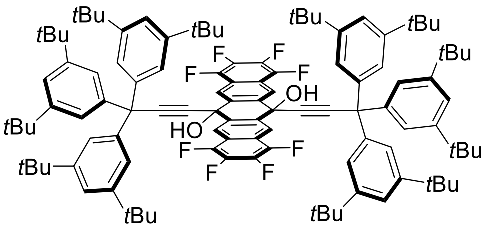

Compound 5e(F₈). To a solution of Tr*–C≡C–H (320 mg, 0.719 mmol) in dry THF (8 mL) at –78 °C was added, *n*-HexLi (0.380 mL, 1.40 M in hexanes, 0.531 mmol) and the solution was stirred for 30 minutes, warmed to rt and stirred for 15 min, and then warmed to 50 °C for 10 min to dissolve the white suspension. The acetylide **4e** solution was cooled to –78 °C and solid **6** (40 mg, 0.088 mmol) was added batch-wise. The reaction mixture was warmed slowly to rt and stirred for 18 h. The reaction mixture was then cooled to –78 °C and quenched with the dropwise addition of satd. aq. NH_4Cl (5 mL). The biphasic solution was warmed to rt and stirred vigorously until the red color dissipated to yellow and EtOAc (10 mL) was added. The organic layer was separated, and the aqueous phase was extracted with EtOAc (2 x 10 mL). The combined organic phases were washed with H_2O (10 mL), brine (10 mL), dried over Na_2SO_4, filtered, and concentrated in vacuo. The crude material was purified by column chromatography (silica gel, CH_2Cl_2/hexanes 1:10 → 1:3) and excess Tr*–C≡C–H (210 mg) was recovered. Compound **5e(F₈)** was collected as the second major band R_f = 0.17 (silica gel, CH_2Cl_2/hexanes 1:3) dried in vacuo to afford the intermediate diol (55 mg, 38%; 95% pure) as a foamy yellow solid. R_f = 0.67 (silica gel, CH_2Cl_2/hexanes 1:1). IR (solid) 3520 (br w), 2964 (s), 2905 (m), 2869 (m), 2231 (w), 1508 (m) cm^{-1}. ^{1}H NMR (400 MHz, $CDCl_3$) δ 8.81 (s, 4H), 7.21 (t, *J* = 1.6 Hz, 6H), 6.91 (d, *J* = 1.6 Hz, 12H), 3.32 (s, 2H), 1.08 (s, 108H). ^{19}F NMR (376 MHz, $CDCl_3$) δ –148.5 to –148.7 (m, 4F), –157.7 to –157.9 (m, 4F). MALDI HRMS (DCTB) *m/z* calcd for $C_{112}H_{132}F_8NaO_2$ ($[M+Na]^+$) 1683.9992, found 1683.9991.

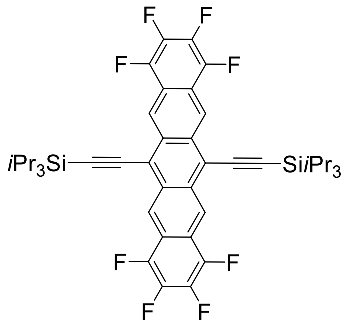

Compound 7b(F8). Synthesized as previously reported by Anthony and coworkers.[3]

Mp = no visible phase change 308–311 °C (partial thermal decomposition evident from TLC analysis of sample). R_f = 0.53 (silica gel, hexanes). UV-vis (CH_2Cl_2) λ_{max} (ε) 306 (224000), 324 (28000), 346 (9170) 540 (6010), 581 (12800) 631 (19700). IR (solid) 3072 (w), 2944 (s), 2892 (w), 2868 (s), 2131 (w), 1587 (s), 1475 (s) cm^{-1}. ^{1}H NMR (400 MHz, $CDCl_3$) δ 9.55 (s, 4H), 1.44–1.25 (m, 42H). ^{13}C {^{1}H, ^{19}F} NMR (100 MHz, $CDCl_3$) δ 141.8, 137.6, 130.8, 120.8, 120.1, 120.09, 110.5, 103.0, 19.0, 11.7. ^{19}F NMR (376 MHz, $CDCl_3$) δ –149.3 to –149.9 (m, 4F), –156.7 to –157.1 (m, 4F). MALDI HRMS (DCTB) *m/z* calcd for $C_{44}H_{46}F_8Si_2$ (M^+) 782.3005, found 782.3003. TGA: Td ≈ 390 °C. DSC: Mp = 313 °C, decomposition, 313 °C (onset), 315 °C (peak).

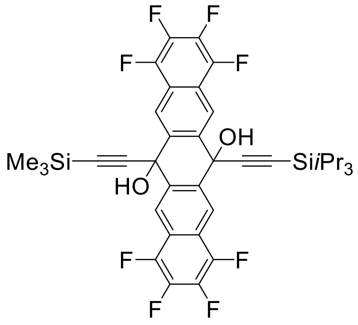

Compound 5c(F$_8$). To a solution of Me_3Si–C≡C–H (2.65 mmol, 260 mg, 0.377 mL) in dry THF (10 mL) at –78 °C was added, *n*-HexLi (1.0 mL, 2.3 M in hexanes, 2.3 mmol). The resulting solution was stirred for 30 min generating acetylide **4c**. The crude **3b(F$_8$)** (0.662 mmol, 420 mg) was added at once to the stirring solution of Me_3Si–C≡C–Li **4c** and the reaction was left to warm to rt slowly overnight. After 14 h the reaction mixture was cooled to –78 °C and satd. aqueous NH_4Cl (5 mL) was added via syringe dropwise. The mixture was warmed to rt, and the organic phase collected. The aqueous phase was extracted with CH_2Cl_2 (3 x 30 mL), and the combined organic extract was washed with H_2O (20 mL), and brine (30 mL), dried over $MgSO_4$, gravity filtered, and concentrated *in vacuo*. The crude solid was dissolved in cold hexanes (30 mL) and suction filtered to remove insoluble impurities, and residual

starting material. The filtrate was concentrated in vacuo to give a crude product as a foamy yellow/orange solid.

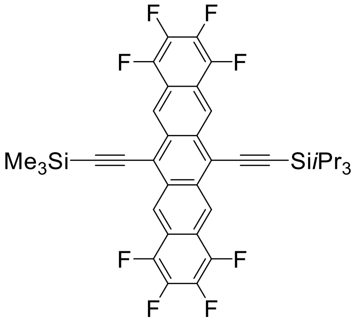

Compound 7c(F$_8$). The crude solid was dissolved in N_2 saturated THF/MeOH (1:4, 25 mL) and nitrogen gas was bubbled through the solution continuously throughout the setup (10 min). $SnCl_2$•$2H_2O$ (4.43 mmol, 1.00 g) was added at once followed by 10% aqueous H_2SO_4 (0.2 mL) and the reaction was stirred for 3 h at reflux (the reaction precipitated some solid quickly, extended reaction times resulted in increased loss of TMS-substituent from the product). The reaction mixture was washed with water (3 x 20 mL), dried over Na_2SO_4, filtered, and concentrated in vacuo. Column chromatography (silica gel, hexanes) and recrystallization from hot acetone after cooling slowly to –30 °C to afforded **7c(F$_8$)** (110 mg, 24% over two steps) as a crystalline blue solid after suction filtration. Mp = no visible phase change ≤300 °C (partial thermal decomposition evident from TLC analysis of sample). R_f = 0.24 (silica gel, hexanes). UV-vis (CH_2Cl_2) λ_{max} (ε) 306 (263000), 322 (sh, 32300), 345 (8800) 538 (5140), 579 (13400) 629 (21800). IR (CH_2Cl_2 cast film) 3071 (w), 2949 (m), 2898 (w), 2868 (m), 2132 (m), 1480 (s), 1445 (s) cm^{-1}. ^1H NMR (400 MHz, $CDCl_3$) δ 9.53 (s, 2H), 9.42 (s, 2H), 1.46–1.31 (m, 21H), 0.53 (s, 9H). ^{13}C {^1H, ^{19}F} NMR (100 MHz, $CDCl_3$) δ 141.8, 137.7, 137.6, 130.6, 130.5, 120.7, 120.6, 120.3, 120.0, 119.99, 119.6, 113.6, 110.6, 102.9, 101.2, 19.0, 11.7, 0.15 (One signal coincident or not observed). ^{19}F NMR (376 MHz, $CDCl_3$) δ –149.6 to –149.9 (m, 4F), –156.7 to –157.0 (m, 4F). MALDI HRMS (DCTB) m/z calcd for $C_{38}H_{34}F_8Si_2$ (M^+) 698.2066, found 698.2057. TGA: Td ≈ 420 °C. DSC: decomposition, 231 °C (onset), 248 °C (peak).

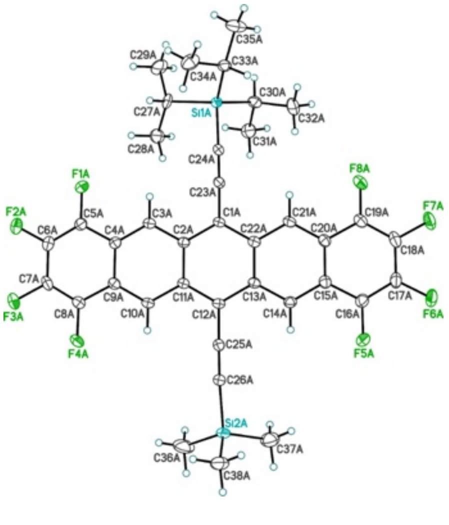

A crystal of **7c(F$_8$)** suitable for X-ray crystallographic analysis was grown at –30 °C by slow evaporation from CH_2Cl_2/MeOH. X-ray data for **7c(F$_8$)** ($C_{38}H_{34}F_8Si_2$), F_w = 698.83; triclinic crystal system; crystal dimensions 0.37 x 0.14 x 0.05 mm; space group P-1 (No. 2); a = 13.7328(8) Å, b = 17.9179(10) Å, c = 22.8336(13) Å; α = 108.5404(9)°, β = 102.1161(10)°, γ = 90.3227(10)°; V = 5192.3(5) Å^3; Z = 6; $\rho_{(calcd)}$ = 1.341 g/cm^3; $2\theta_{max}$ = 51.64°; μ = 0.172 mm^{-1}; T = 173 K; total data collected = 117604; R_1 = 0.0484

[12537 observed reflections with $I \geq 2\sigma(I)$]; wR_2 = 0.1373 for 19795 data, 0 restraints, and 1315 variables; largest difference, peak and hole = 0.340 and –0.312 e Å^{-3}. CCDC 2240560.

Photochemical Stability Studies

For compounds **2a**, **2b**, **2e**, and **7a**: Two solutions were prepared. A volumetric flask (200 mL) was charged with pentacene and solvent (200 mL) to give a solution of ca. 10^{-5} M. From this flask, 100 mL was transferred to a secondary volumetric flask, and the remaining 100 mL was reserved. The solution in the 100 mL volumetric was sparged for ca. 30 min with N_2, and nitrogen saturated solvent was then added to return the volume to 100 mL. Two samples were prepared in cuvettes, the reserved solution solvent was openly exposed to the atmosphere during preparation and the nitrogen saturated solution was not. Each cuvette was then capped and wrapped with parafilm to minimize solvent loss. Aluminum foil was used to exclude light during the experimental setup, and all trials in each set of experiments were prepared and analyzed on the same day. HPLC grade THF was used in the first set of experiments (THF does not contain BHT as stabilizer. THF without stabilizer (BHT), obtained from non-stabilized UV-vis grade THF, was used for the second set of experiments in THF. The temperature remained between 23–26 °C for the entire length of all experiments. Time points were taken on hour or half hour intervals, and data was collected until all samples decomposed (transformed) to at least ca. half the initial concentration.

For compounds **2e(F$_8$)**, **7b(F$_8$)**, and **7c(F$_8$)**: A volumetric flask (200 mL) was charged with a pentacene derivative and THF containing BHT stabilizer (200 mL) to give a solution of ca. 10^{-5} M. Two stock solutions were prepared using individually weighed samples, and each was transferred into a cuvette that was capped and wrapped with parafilm to minimize solvent losses. The stock solution was stored in the dark throughout the analyses and used as a dark control. The temperature during the investigation remained between 23–26 °C for the entire length of all experiments. Time points were taken on hour or half hour intervals and data was collected until all samples have decomposed (transformed) to less than ca. half the initial concentration.

Table S1. Quantities for UV-vis endoperoxidation sample preparation in THF

	Conc. (M)	Mass (mg)	mmol	Vol (mL)
Non-stabilized **2e**	1.10×10^{-5}	3.27	2.20×10^{-3}	200
**2b**	1.62×10^{-5}	3.46	3.26×10^{-3}	200
**2a**	1.11×10^{-5}	2.45	2.22×10^{-3}	200
BHT stabilized **2e**	1.34×10^{-5}	3.98	2.68×10^{-3}	200
**2b**	1.24×10^{-5}	2.64	2.49×10^{-3}	200
**2a**	1.21×10^{-5}	2.67	2.42×10^{-3}	200
**7a**	1.31×10^{-5}	1.89	2.61×10^{-3}	200
BHT stabilized **7c(F$_8$)**	1.22×10^{-5}	1.70	2.43×10^{-3}	200
	1.15×10^{-5}	1.80	2.30×10^{-3}	200
**2e(F$_8$)**	1.29×10^{-5}	4.20	2.58×10^{-3}	200
	1.16×10^{-5}	3.78	2.32×10^{-3}	200
**7b(F$_8$)**	1.41×10^{-5}	2.20	2.81×10^{-3}	200
	1.44×10^{-5}	2.26	2.89×10^{-3}	200

Photochemical Stabilities in CH_2Cl_2

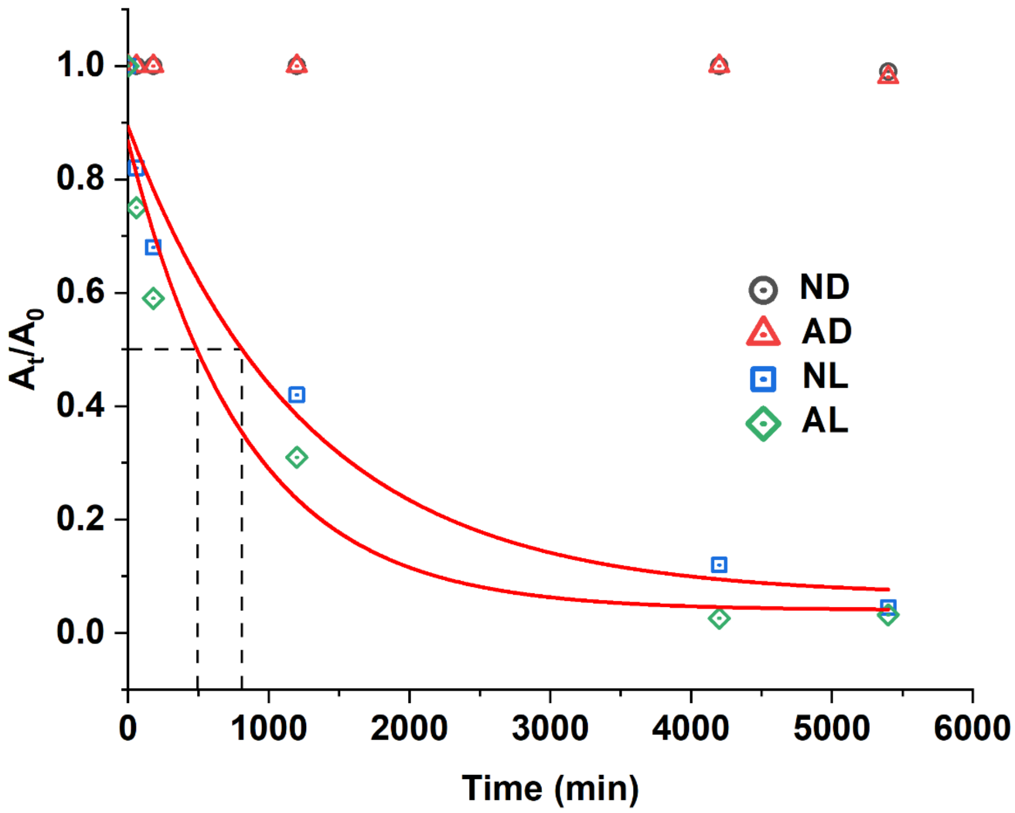

Figure S1. Absorbance-time profiles of compound **2a** in CH_2Cl_2; half-life of samples which were in the presence of (N_2-light (NL) and Air-light (AL)) and absence of (N_2-dark (ND), Air-dark (AD)) light. Hashed cross section indicates the half-life ($t_{1/2}$). A_t = Absorption at time interval. Time, A_0 = initial absorption.

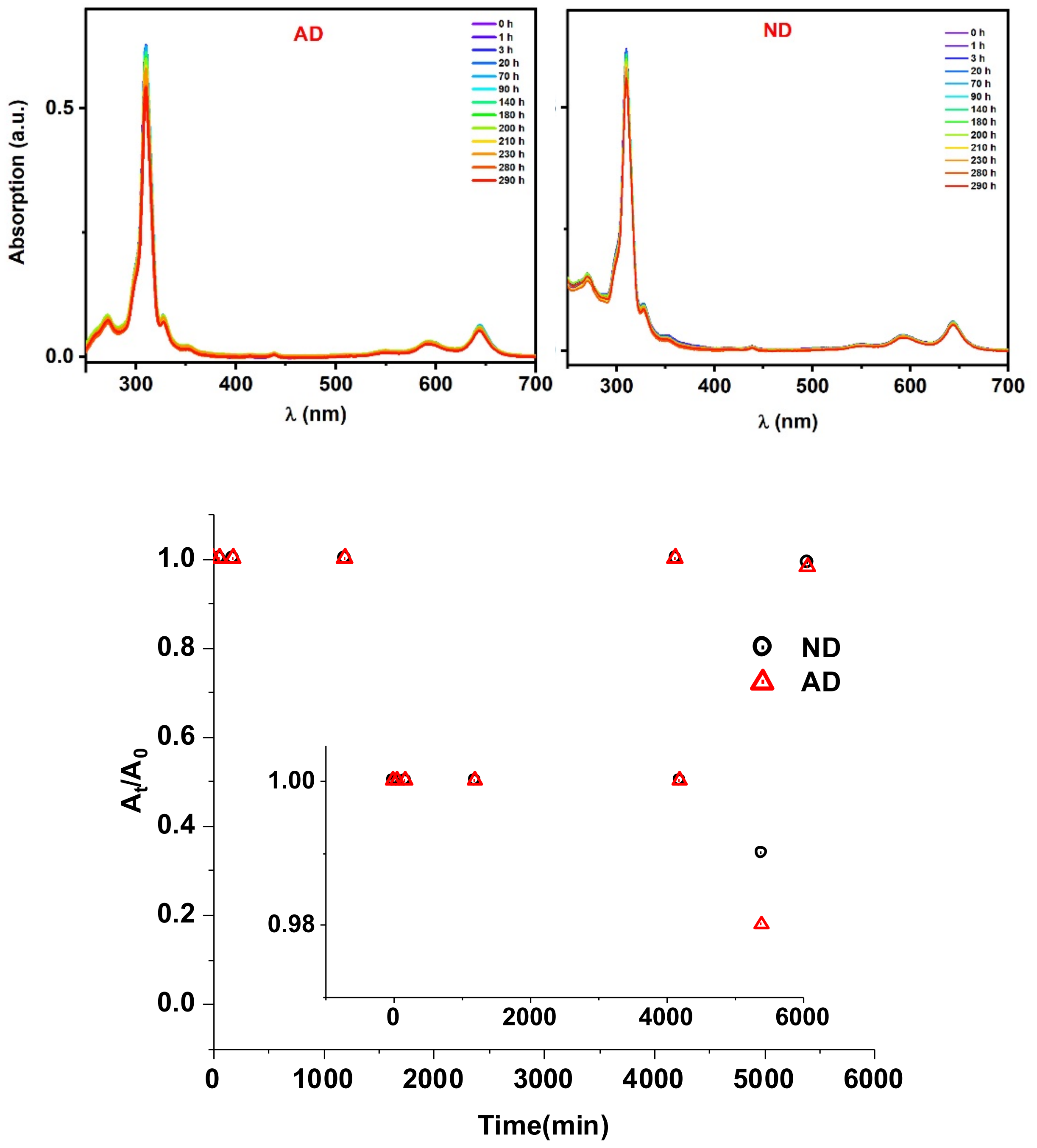

Figure S2. UV-vis spectra of air/dark (AD), and N_2 saturated/dark (ND) solutions of compound **2a** in CH_2Cl_2. Absorbance-time profiles of compound **2a** in CH_2Cl_2; half-life of samples which were in the presence of light (ND and AD) indicated as $t_{1/2}$. Inset shows expansion along y-axis (A_t/A_0). A_t = Absorption at time interval. Time, A_0 = initial absorption.

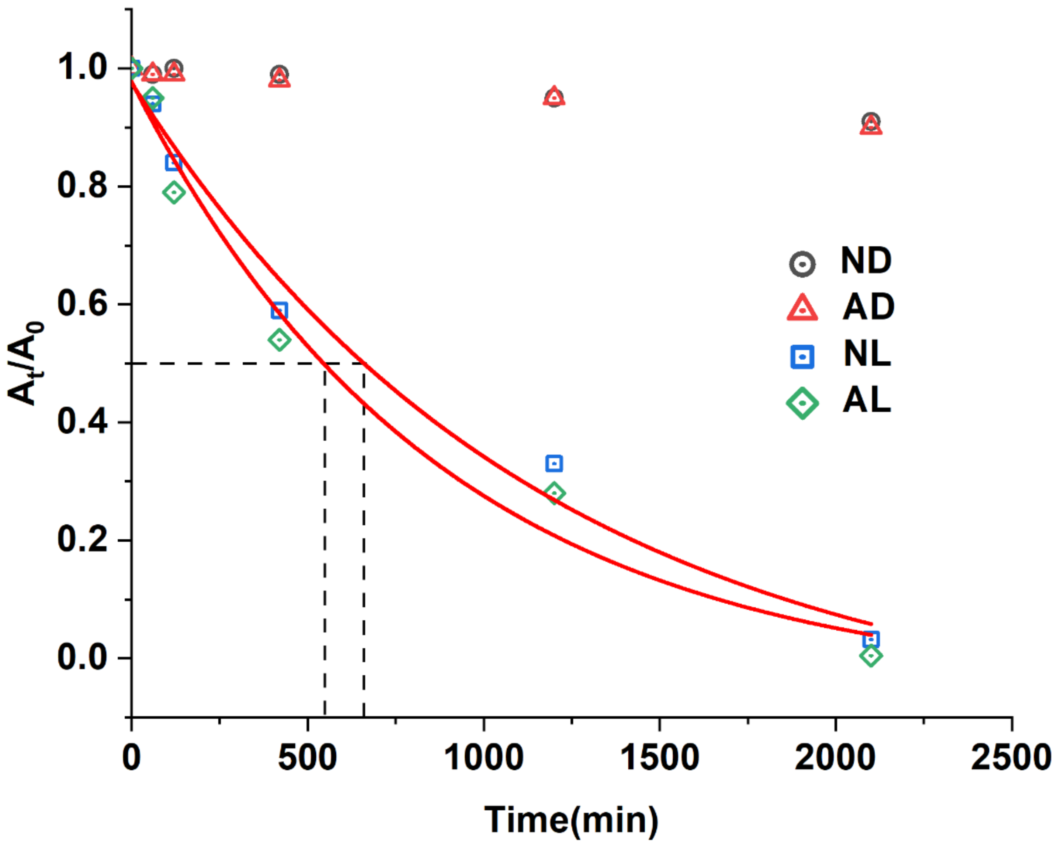

Figure S3. Absorbance-time profiles of compound **2e** in CH_2Cl_2; half-life of samples which were in the presence of (N_2-light (NL) and Air-light (AL)) and absence of (N_2-dark (ND), Air-dark (AD)) light. Hashed cross section indicates the half-life ($t_{1/2}$). A_t = Absorption at time interval. Time, A_0 = initial absorption.

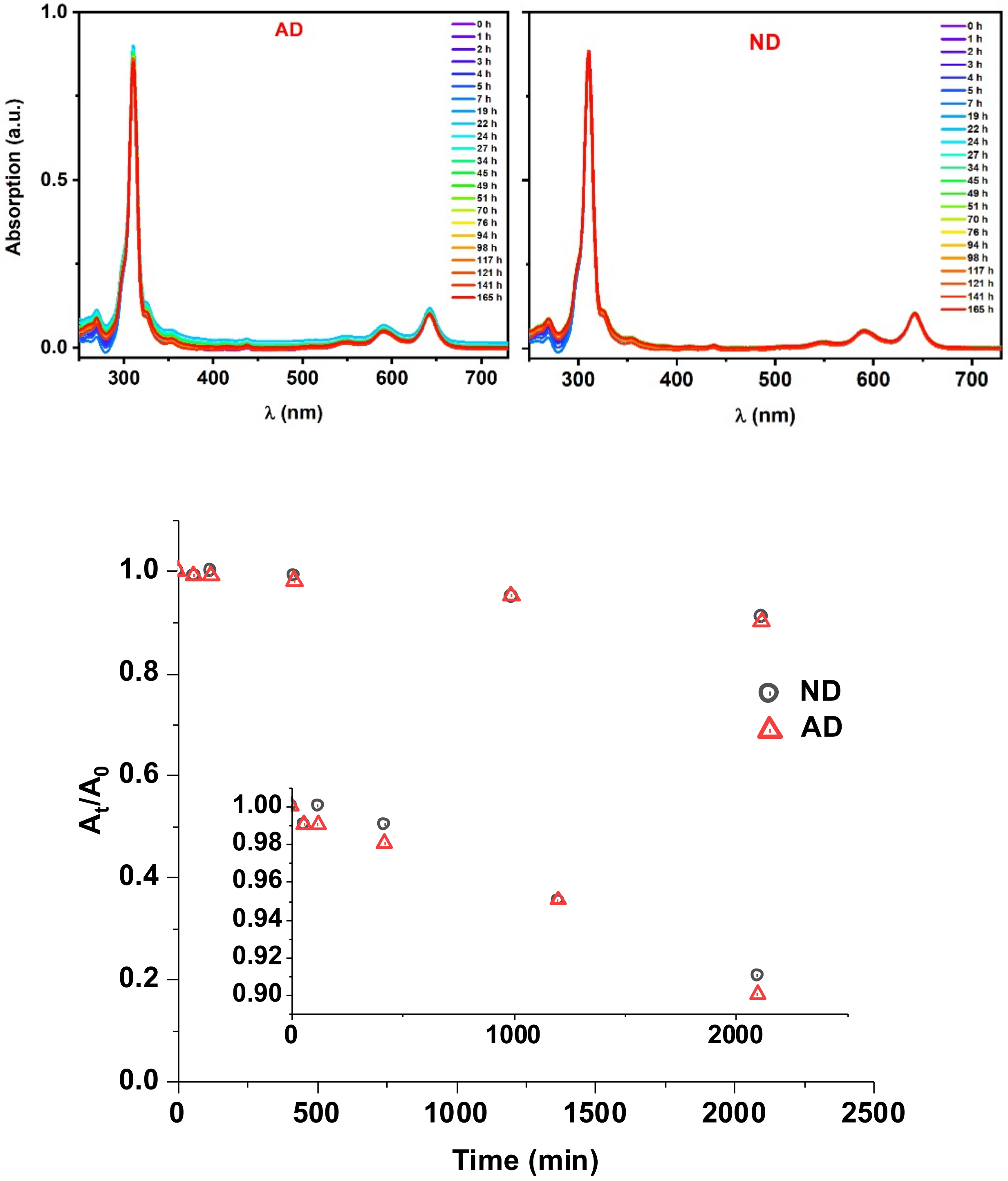

Figure S4. Absorbance-time profiles of compound **2e** in CH_2Cl_2; half-life of samples which were in the presence of light (ND and AD) indicated as $t_{1/2}$. Inset shows expansion along y-axis (A_t/A_0).

Photochemical Stability Studies of 2a, 2b, 2e, 2e(F8), 7a, 7b(F8), and 7c(F8) in THF

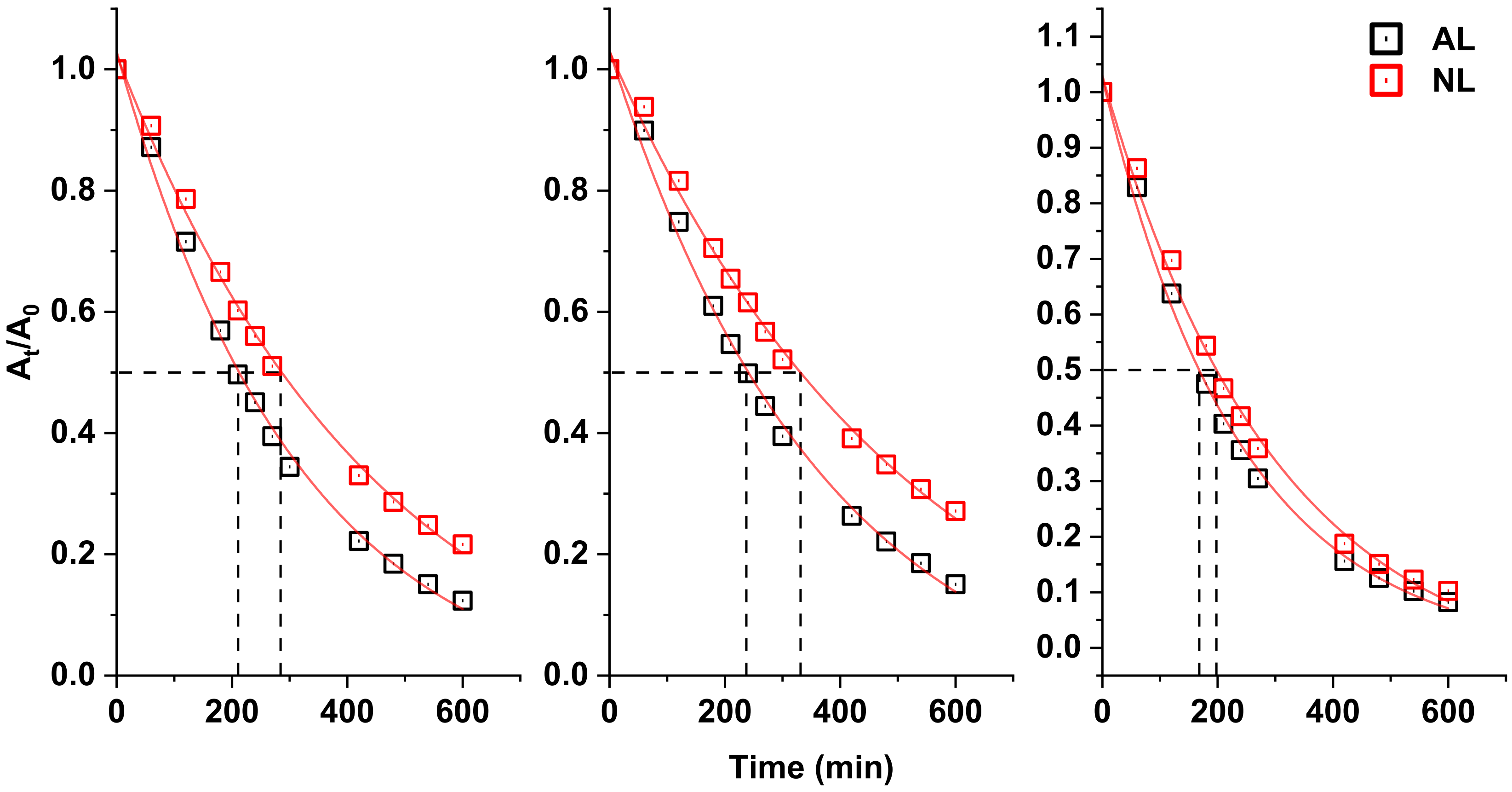

Figure S5. Absorbance-time profiles of compounds **2a** (left), **2b** (middle), and **2e** (right) as solutions in stabilizer (BHT) free THF over a period of 600 minutes. Half-life of sample solutions which were in ambient THF exposed to air and light (AL), and nitrogen saturated THF exposed to light (NL) are denoted $t_{1/2}$. Hashed cross section indicates the half-life ($t_{1/2}$).

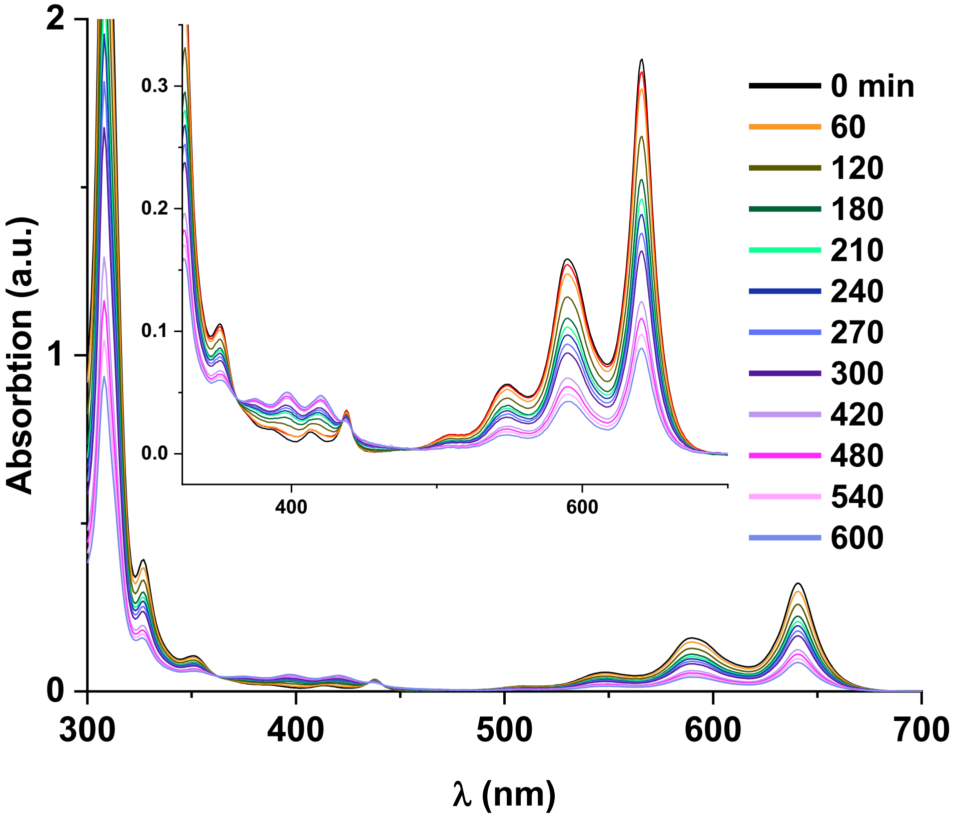

Figure S6. UV-vis absorbance-time profile of **2a** (NL), in stabilizer free THF over a period of 600 min. Inset shows expansion along the x- and y-axis (A_t/A_0).

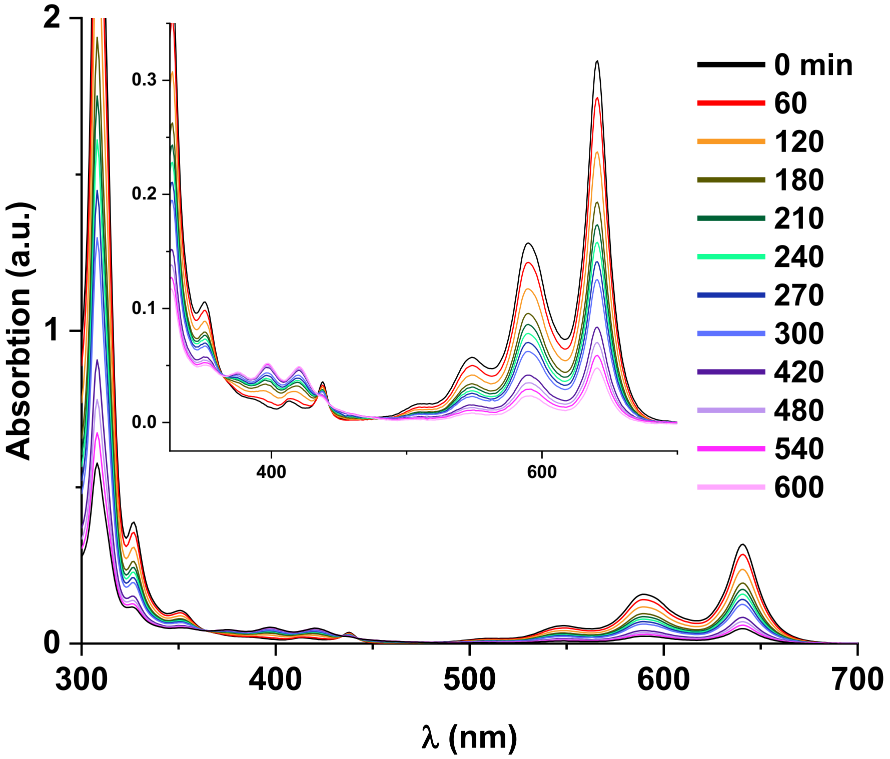

Figure S7. UV-vis absorbance-time profile of **2a** (AL), in stabilizer free THF over a period of 600 min. Inset shows expansion along the x- and y-axis (A_t/A_0).

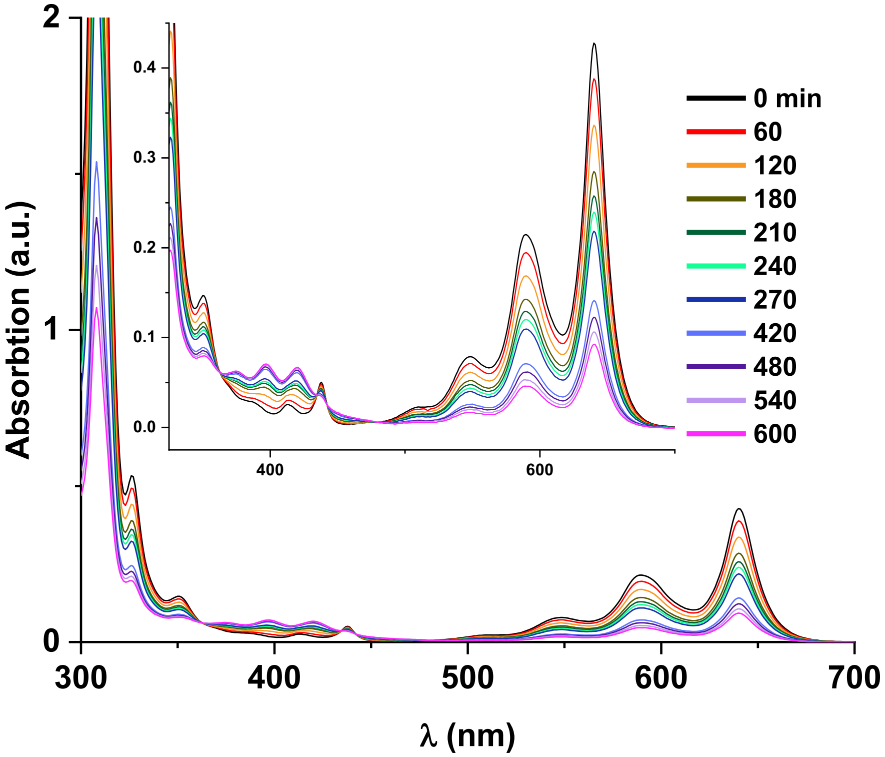

Figure S8. UV-vis absorbance-time profile of **2b** (NL), in stabilizer free THF over a period of 600 min. Inset shows expansion along the x- and y-axis (A_t/A_0).

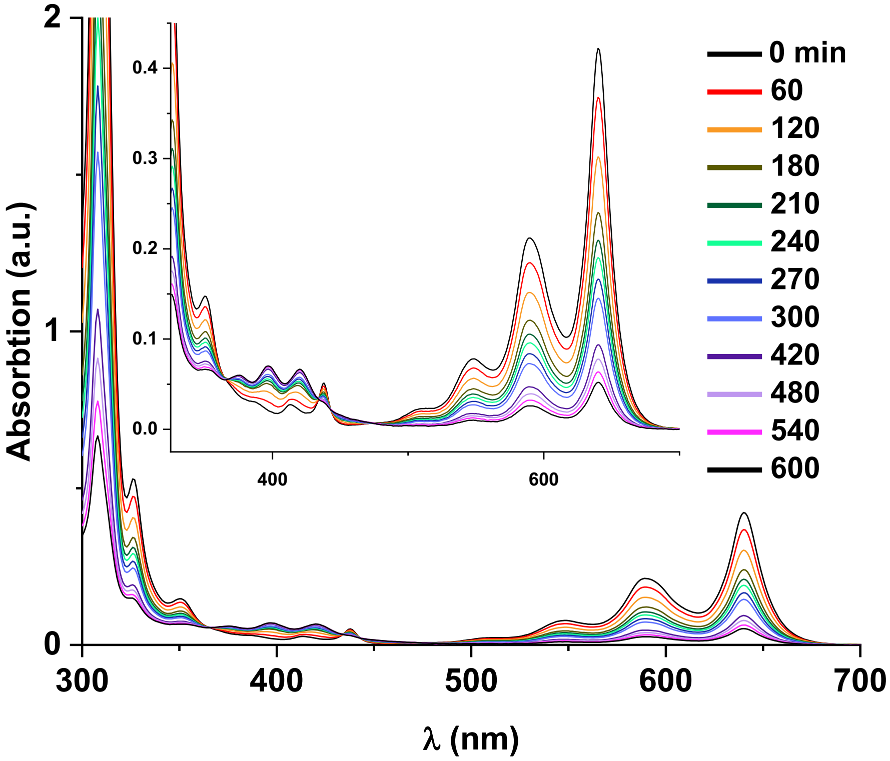

Figure S9. UV-vis absorbance-time profile of **2b** (AL), in stabilizer free THF over a period of 600 min. Inset shows expansion along the x- and y-axis (A_t/A_0).

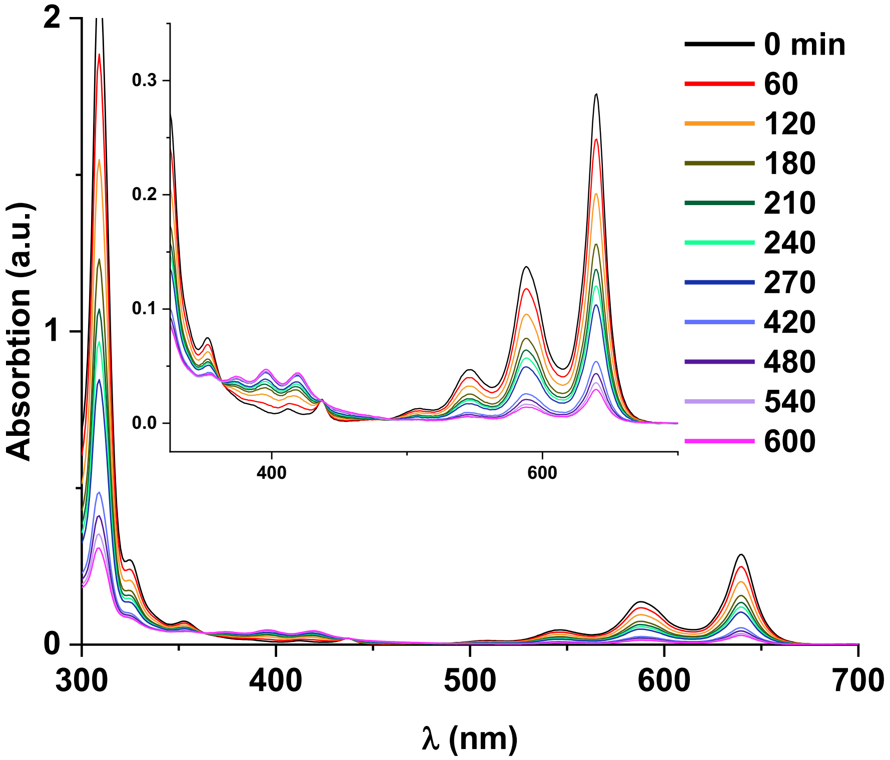

Figure S10. UV-vis absorbance-time profile of **2e** (NL), in stabilizer free THF over a period of 600 min. Inset shows expansion along the x- and y-axis (A_t/A_0).

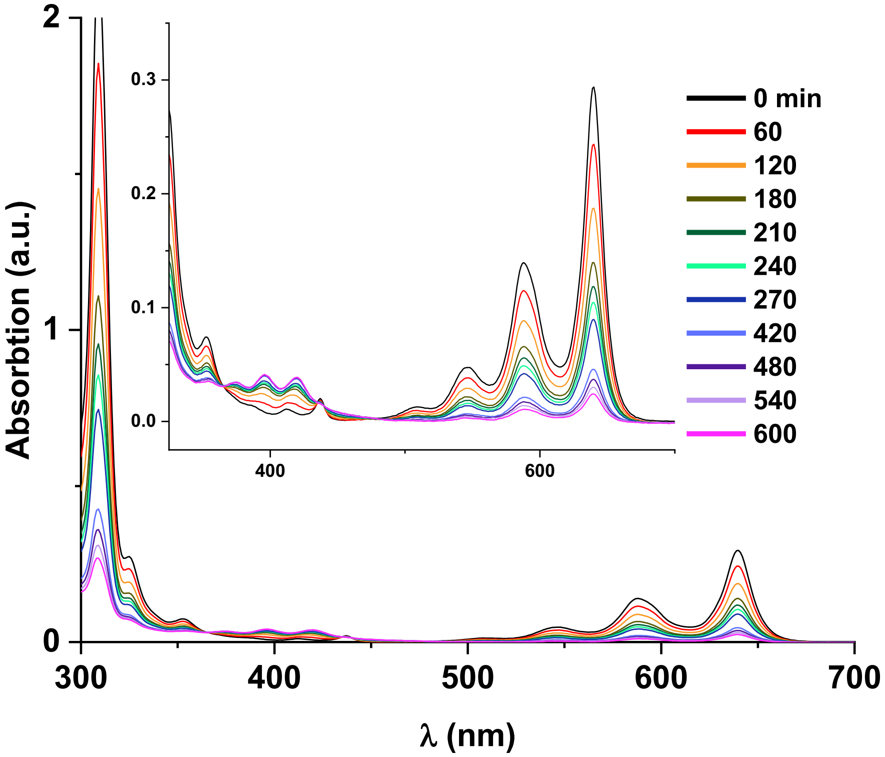

Figure S11. UV-vis absorbance-time profile of **2e** (AL), in stabilizer free THF over a period of 600 min. Inset shows expansion along the x- and y-axis (A_t/A_0).

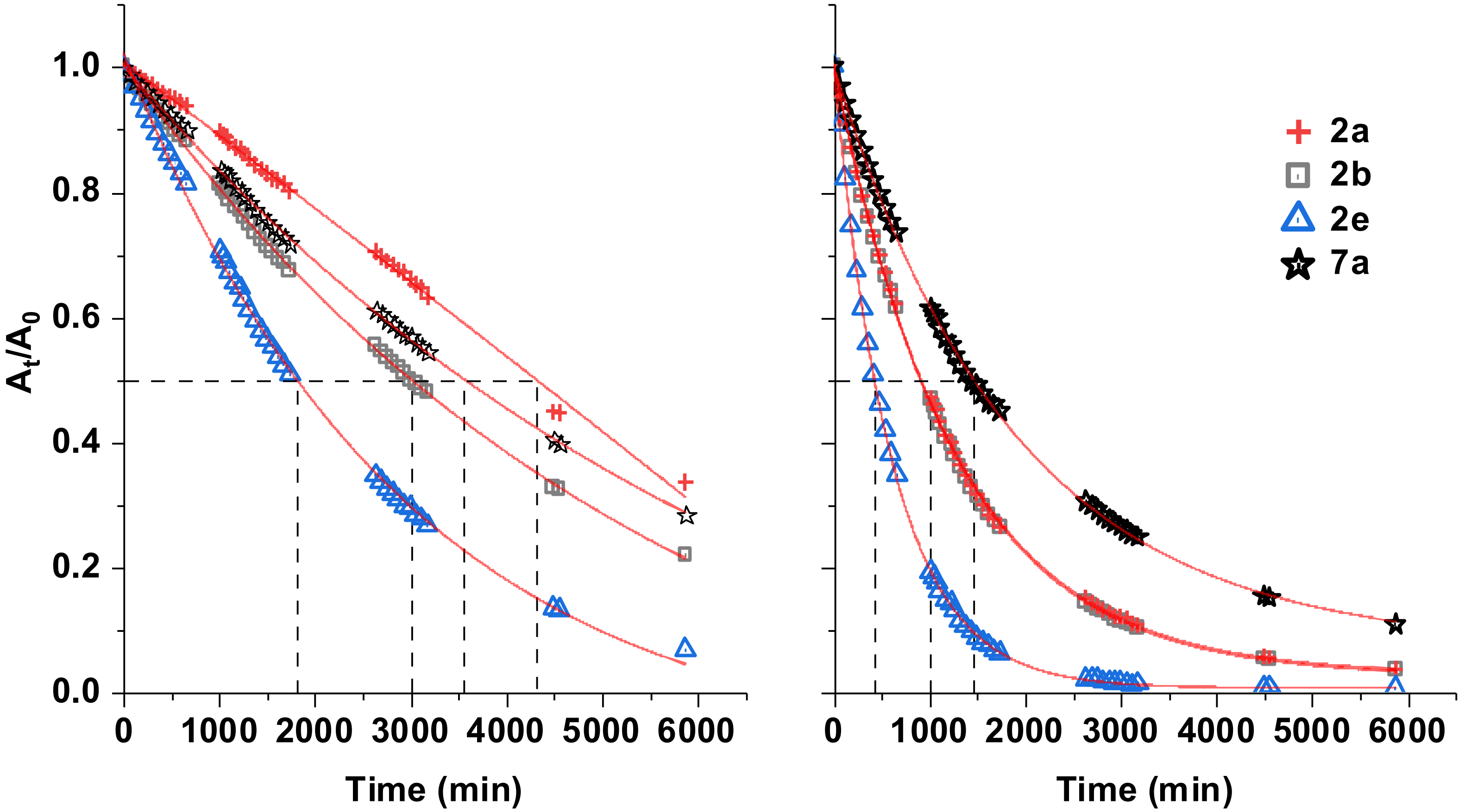

Figure S12. Absorbance-time profiles of compounds **2a** (red **+**), **2b** (grey ■), **2e** (blue ▲), and **7a** (black ☆) as solutions in BHT stabilized THF over a period of 6000 minutes. Half-life of samples in the presence of light in nitrogen saturated (NL, left), or ambient (AL, right) THF. Hashed cross section indicates the half-lives ($t_{1/2}$).

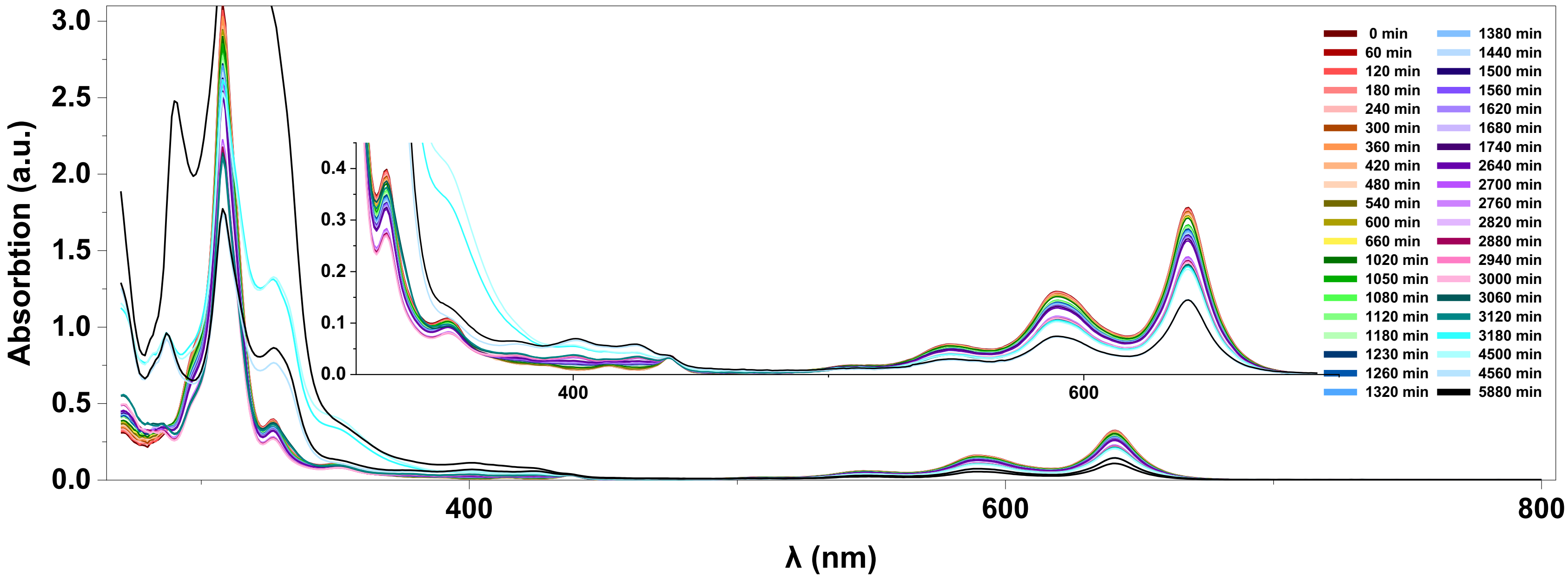

Figure S13. UV-vis absorbance-time profile of **2a** (NL), in BHT stabilized THF over a period of 5880 min. Inset shows expansion along the x- and y-axis (A_t/A_0).

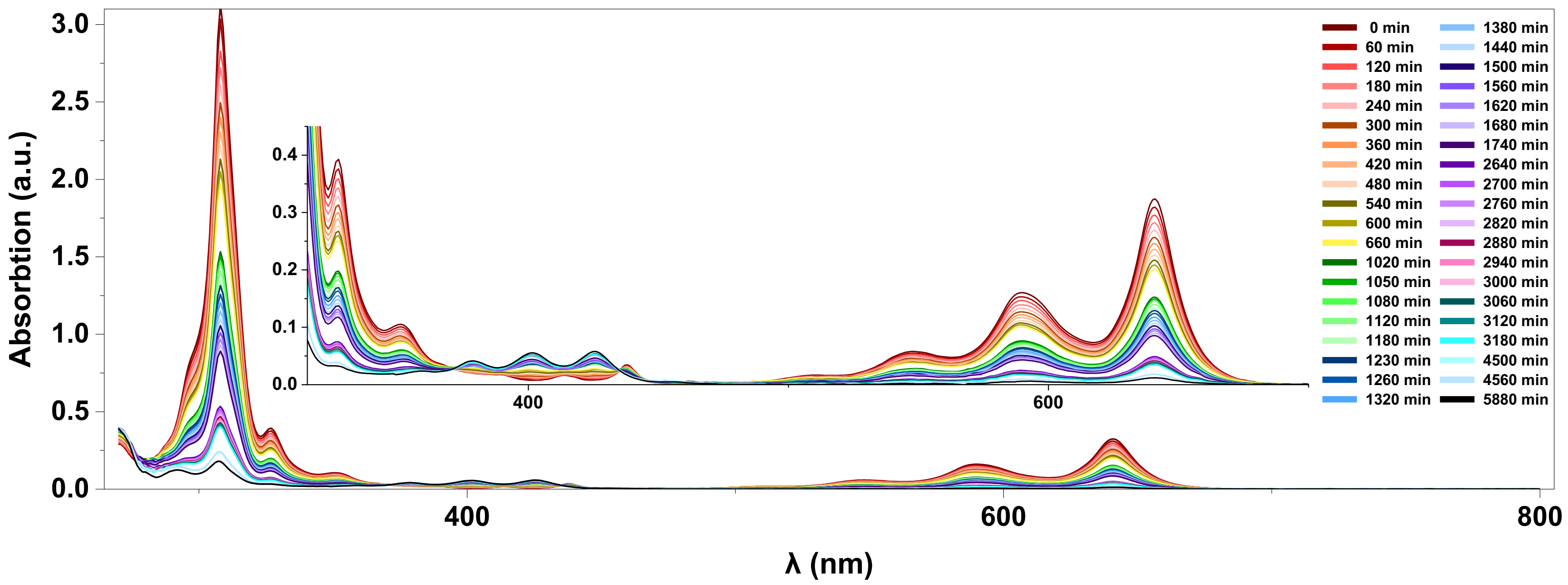

Figure S14. UV-vis absorbance-time profile of **2a** (AL), in BHT stabilized THF over a period of 5880 min. Inset shows expansion along the x- and y-axis (A_t/A_0).

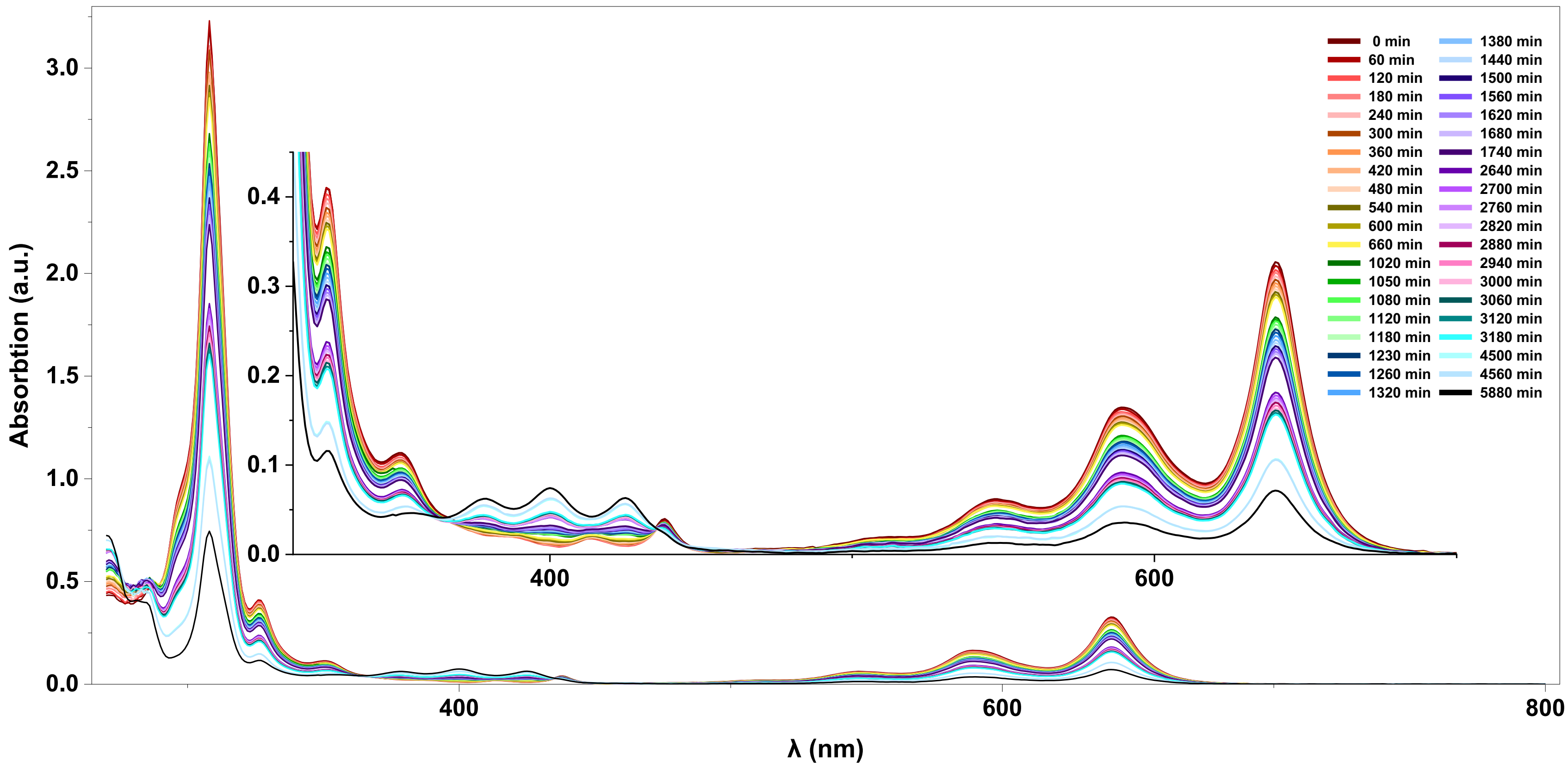

Figure S15. UV-vis absorbance-time profile of **2b** (NL), in BHT stabilized THF over a period of 5880 min. Inset shows expansion along the x- and y-axis (A_t/A_0).

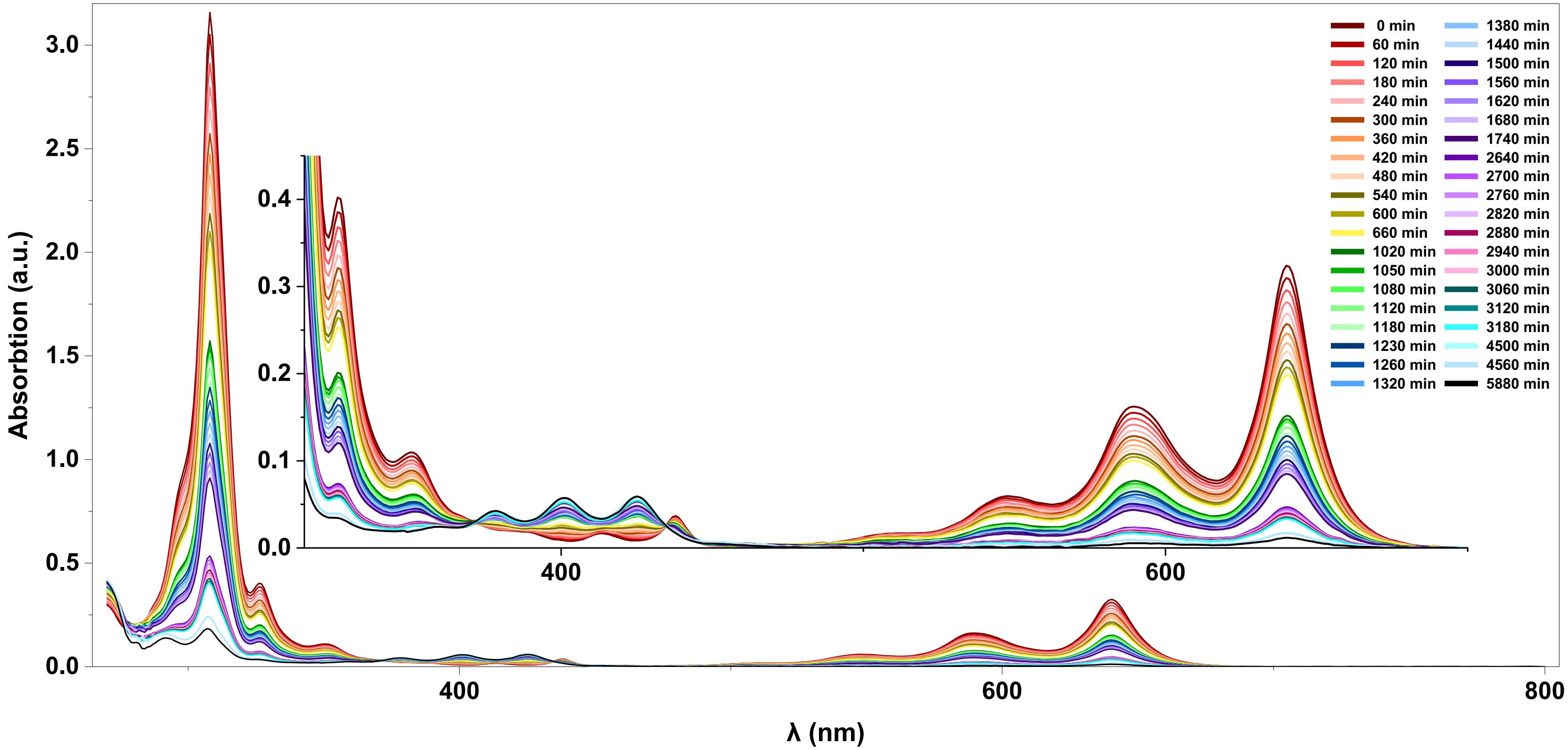

Figure S16. UV-vis absorbance-time profile of **2b** (AL), in BHT stabilized THF over a period of 5880 min. Inset shows expansion along the x- and y-axis (A_t/A_0).

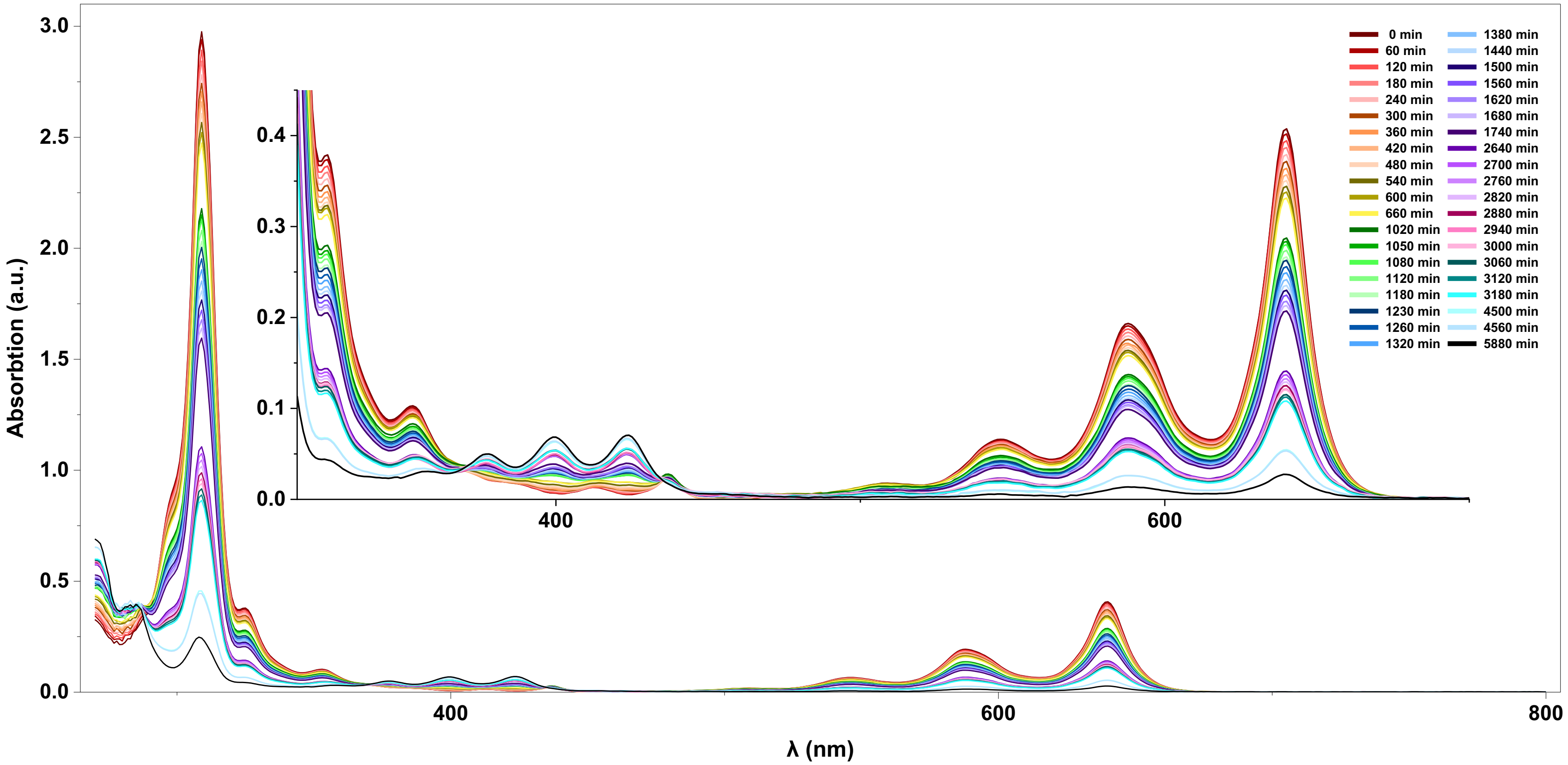

Figure S17. UV-vis absorbance-time profile of **2e** (NL), in BHT stabilized THF over a period of 5880 min. Inset shows expansion along the x- and y-axis (A_t/A_0).

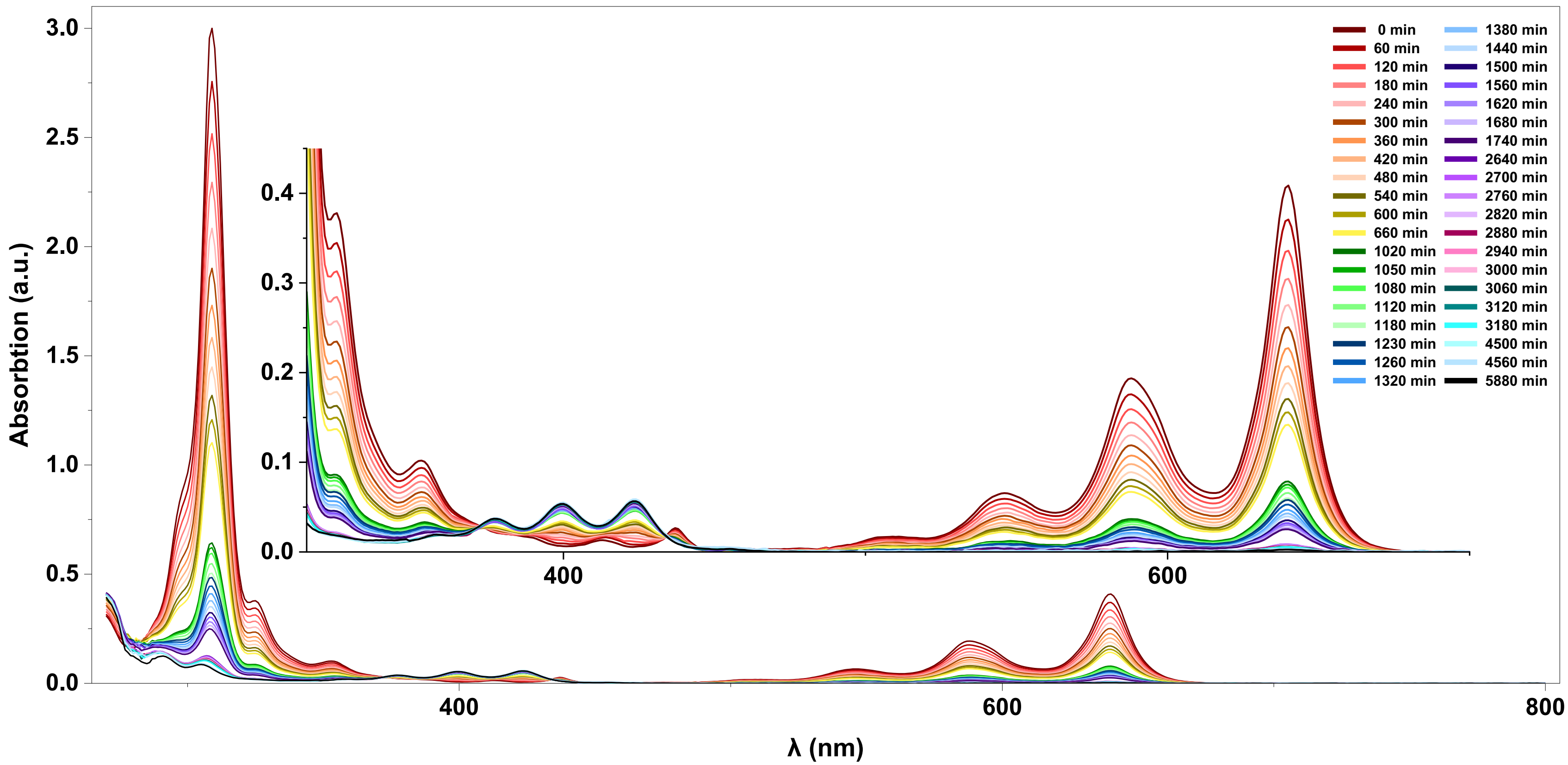

Figure S18. UV-vis absorbance-time profile of **2e** (AL), in BHT stabilized THF over a period of 5880 min. Inset shows expansion along the x- and y-axis (A_t/A_0).

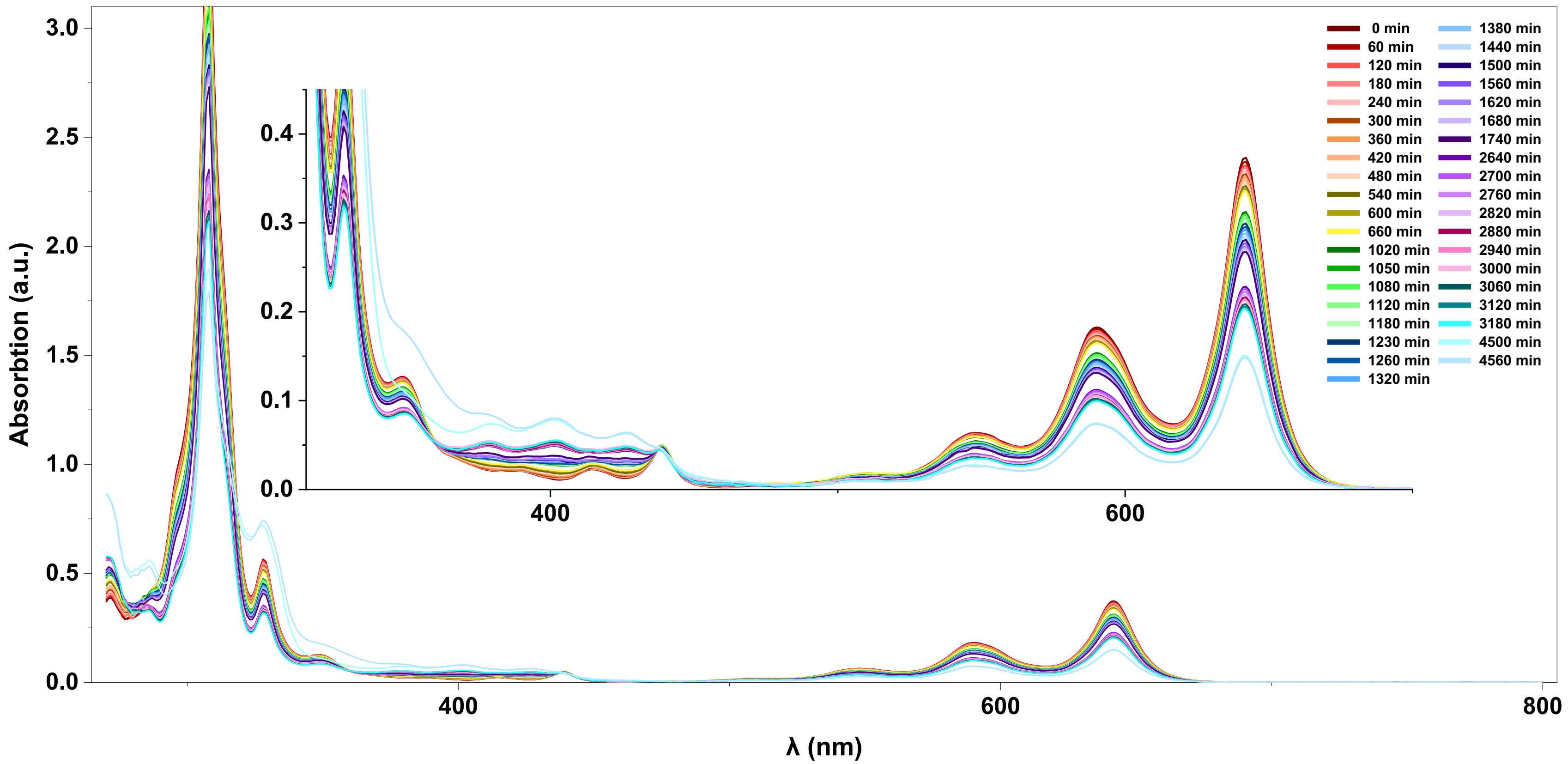

Figure S19. UV-vis absorbance-time profile of **7a** (NL), in BHT stabilized THF over a period of 4560 min. Inset shows expansion along the x- and y-axis (A_t/A_0).

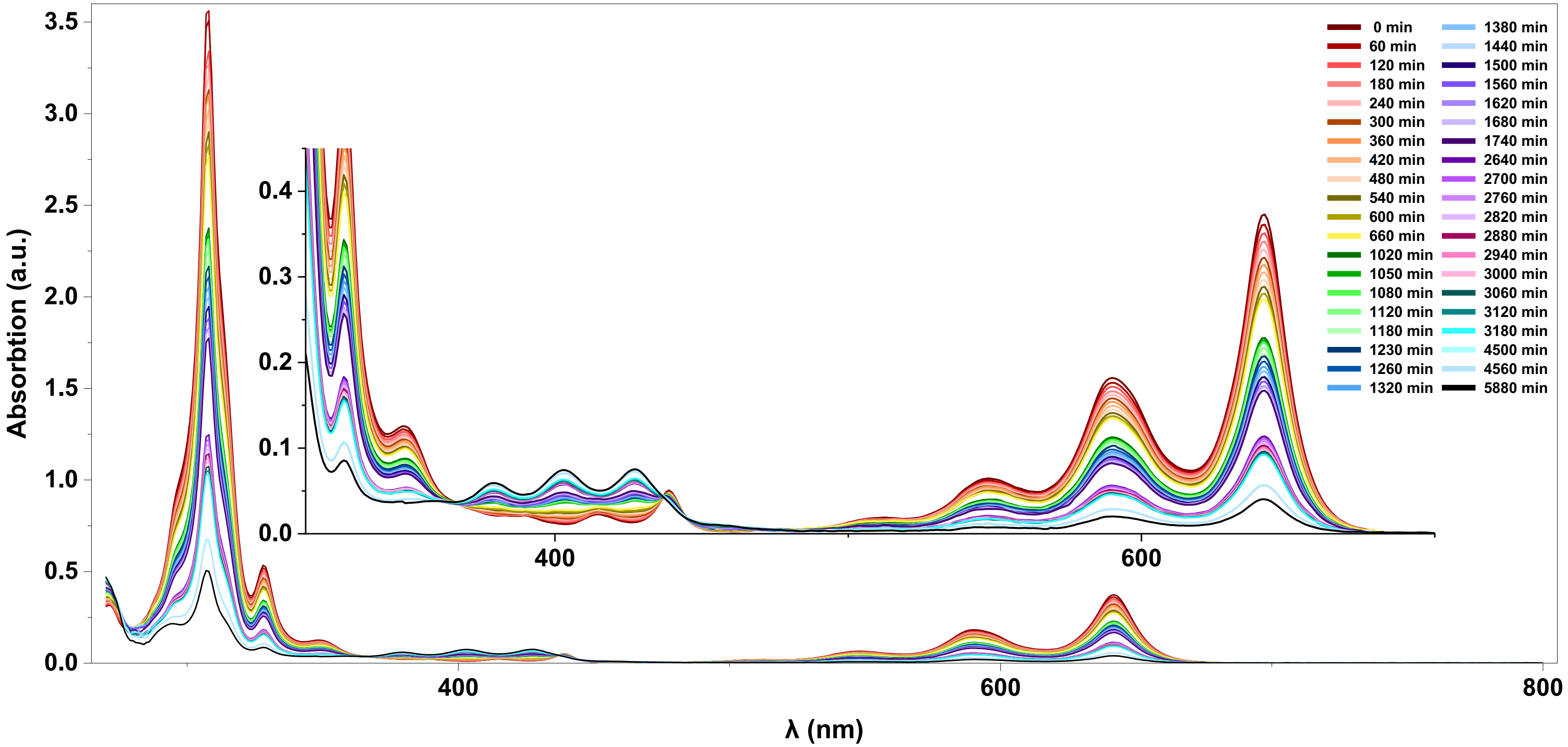

Figure S20. UV-vis absorbance-time profile of **7a** (AL), in BHT stabilized THF over a period of 5880 min. Inset shows expansion along the x- and y-axis (A_t/A_0).

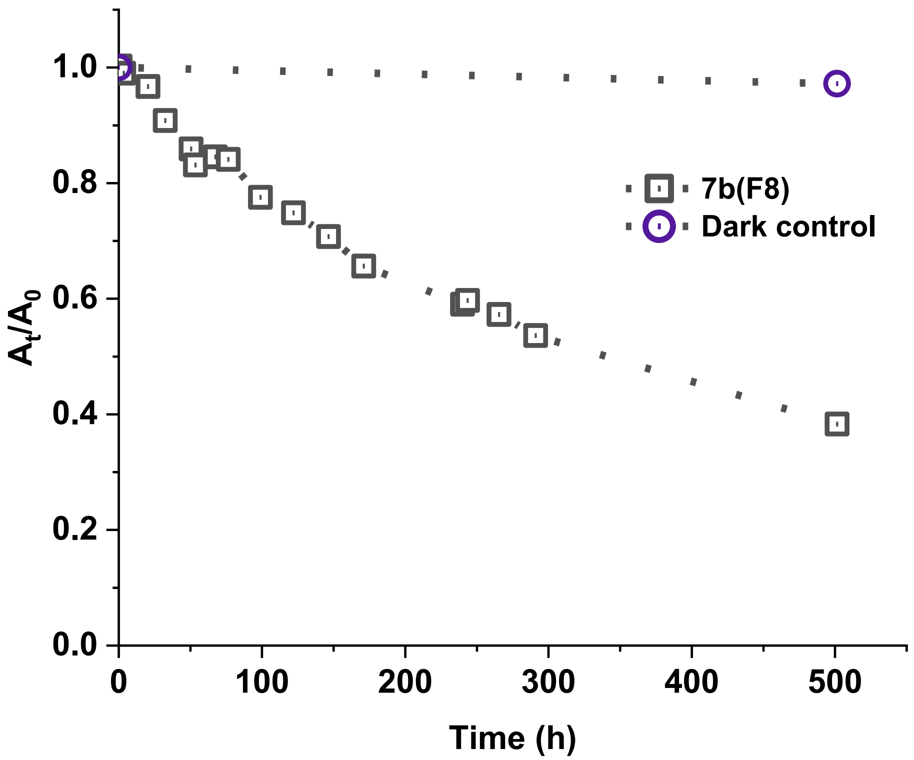

Figure S21. UV-vis absorbance-time profile of **7b(F8)**, under ambient laboratory conditions in BHT stabilized THF over a period of 502 hours. Averages of two trials were used, and the difference between the two measurements was less than ±10% in all cases.

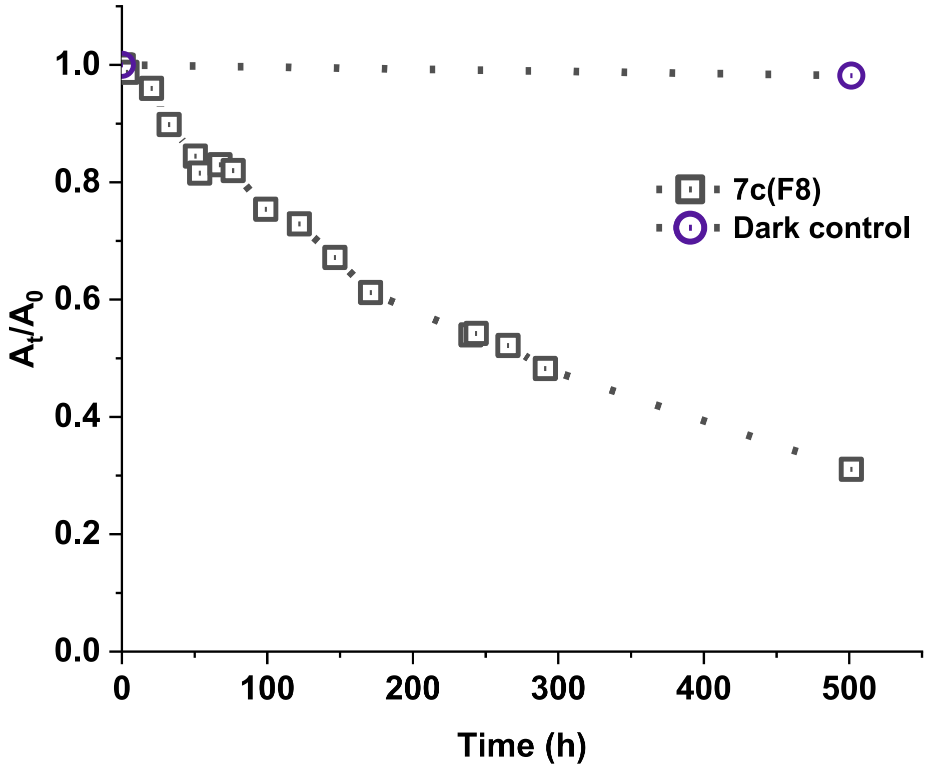

Figure S22. UV-vis absorbance-time profile of **7c(F8)**, under ambient laboratory conditions in BHT stabilized THF over a period of 502 hours. Averages of two trials were used, and the difference between the two measurements was less than ±10% in all cases.

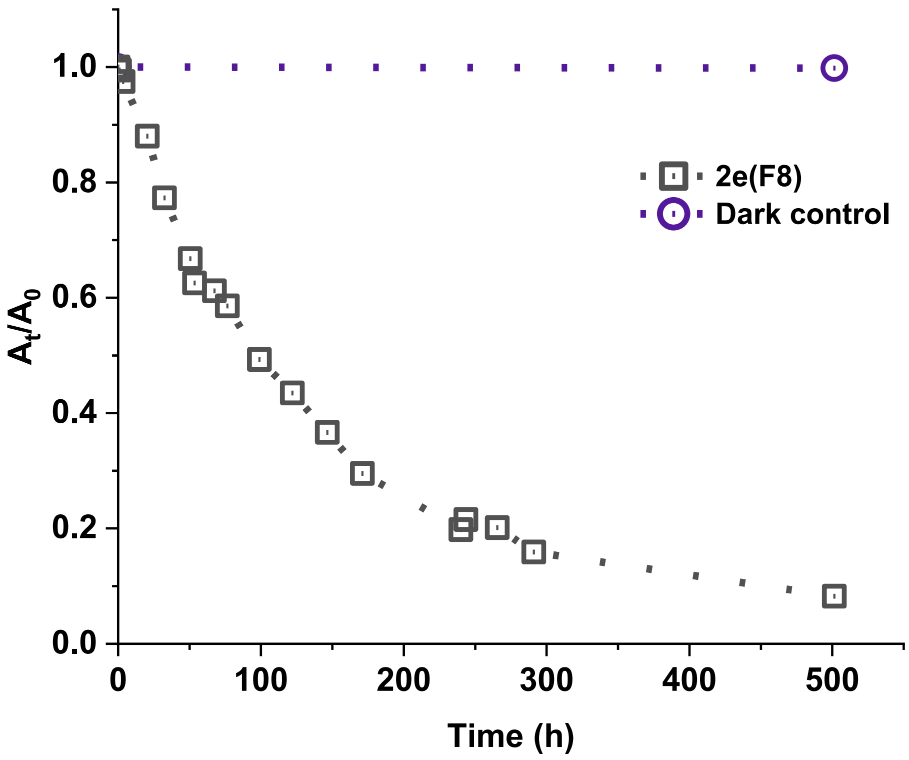

Figure S23. UV-vis absorbance-time profile of **2e(F8)**, under ambient laboratory conditions in BHT stabilized THF over a period of 502 hours. Averages of two trials were used, and the difference between the two measurements was less than ±10% in all cases.

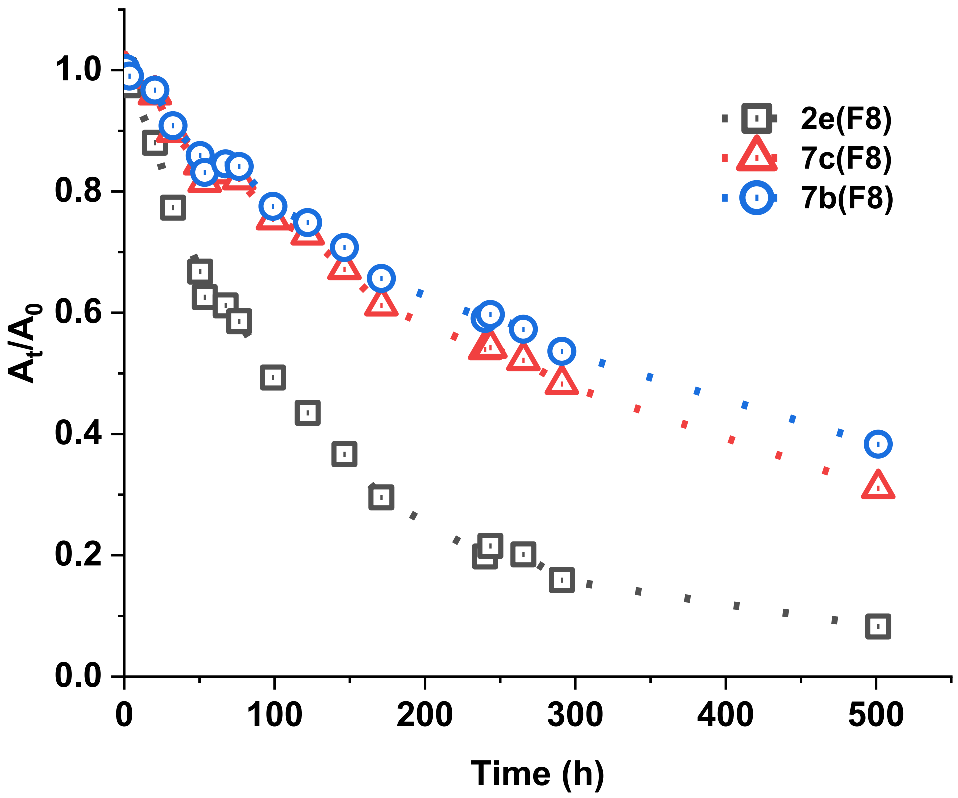

Figure S24. UV-vis absorbance-time profile of **2e(F8)**, **7c(F8)**, and **7b(F8)** under ambient laboratory conditions in BHT stabilized THF over a period of 502 hours. Averages of two trials were used, and the difference between the two measurements was less than ±10% in all cases.

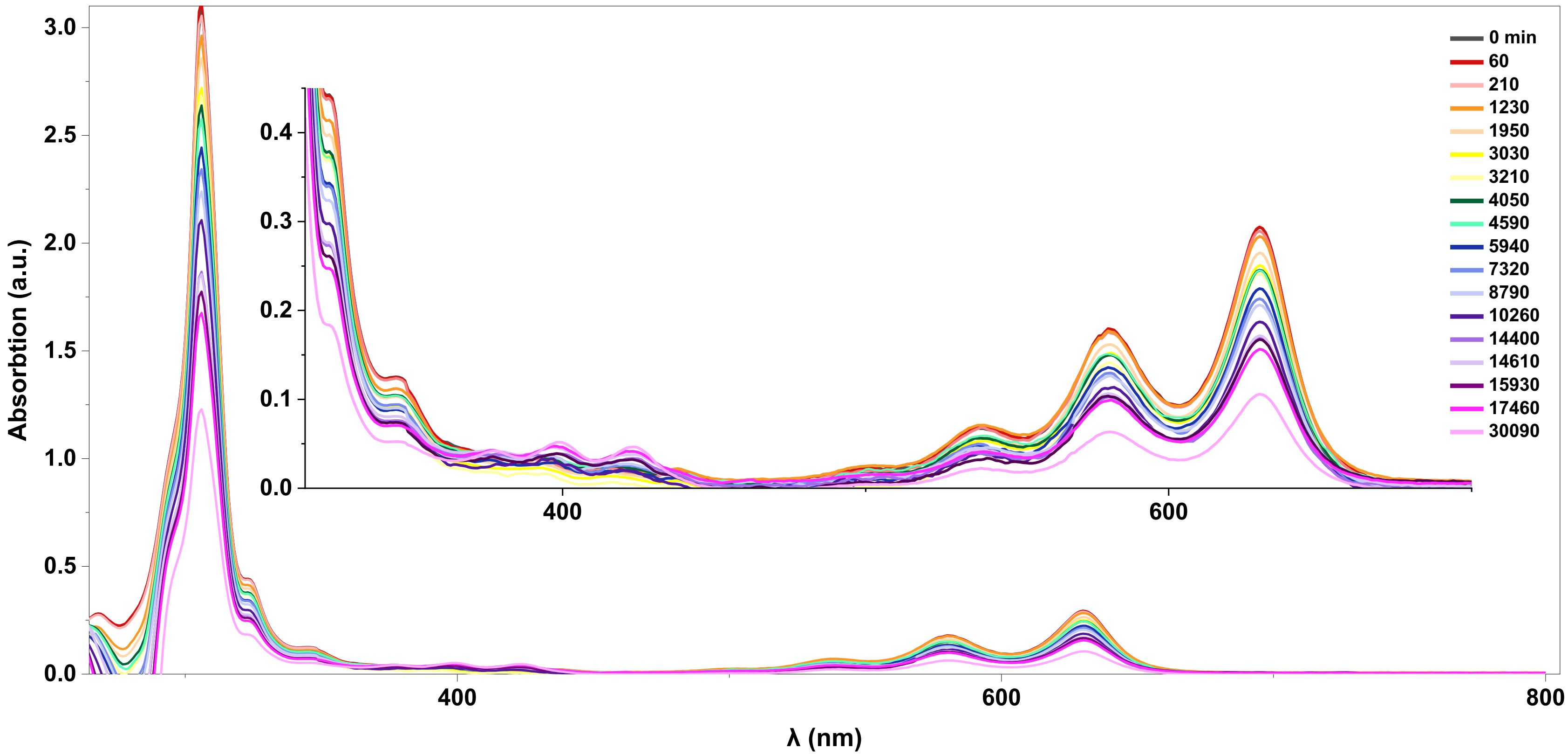

Figure S25. UV-vis absorbance-time profile of **7b(F8)**, in BHT stabilized THF over a period of 502 hours. Inset shows expansion along the x- and y-axis (A_t/A_0). Averages of two trials were used, and the difference between the two measurements was less than ±10% in all cases.

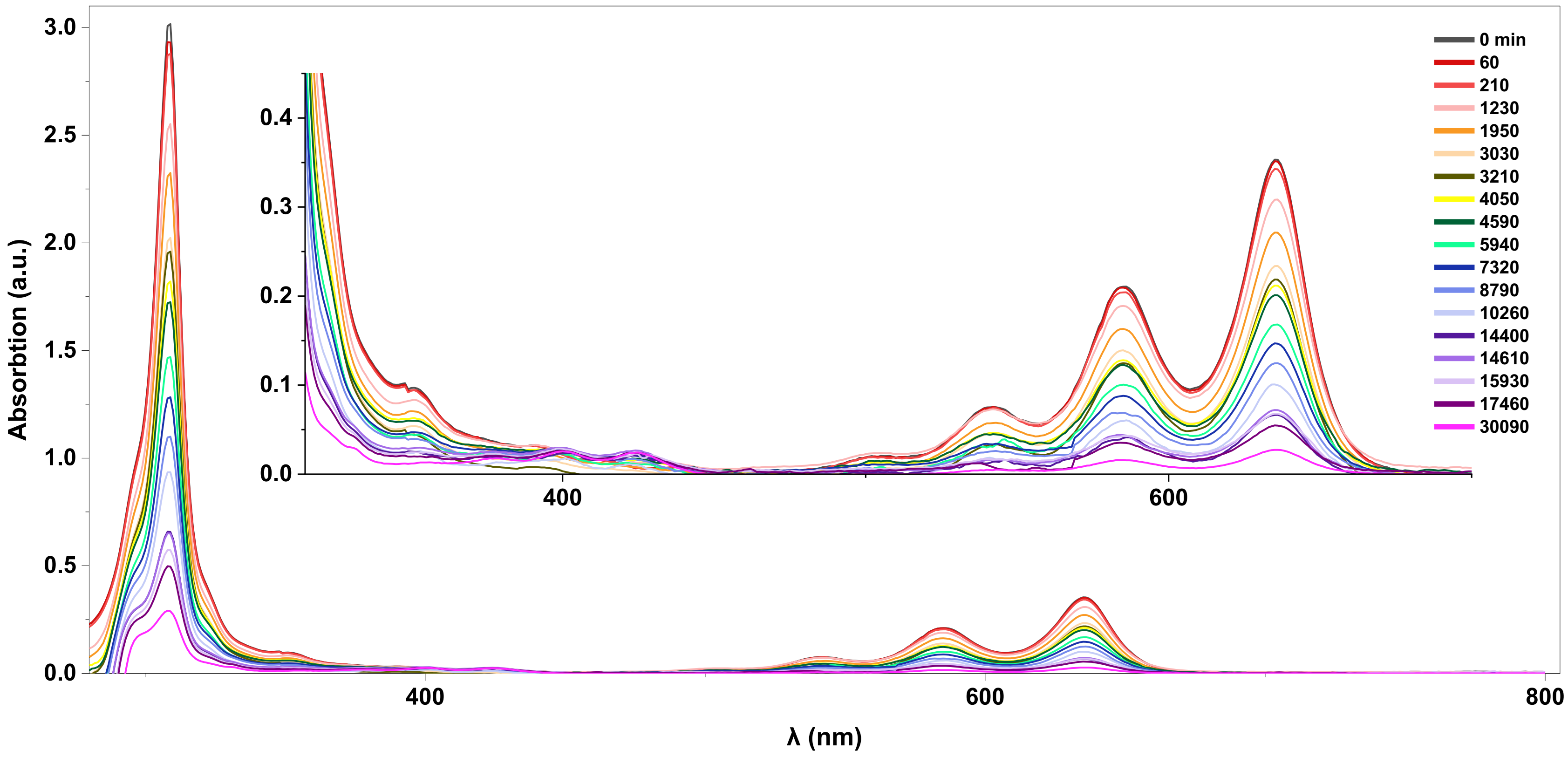

Figure S26. UV-vis absorbance-time profile of **2e(F8)**, in BHT stabilized THF over a period of 502 hours. Inset shows expansion along the x- and y-axis (A_t/A_0). Averages of two trials were used, and the difference between the two measurements was less than ±10% in all cases.

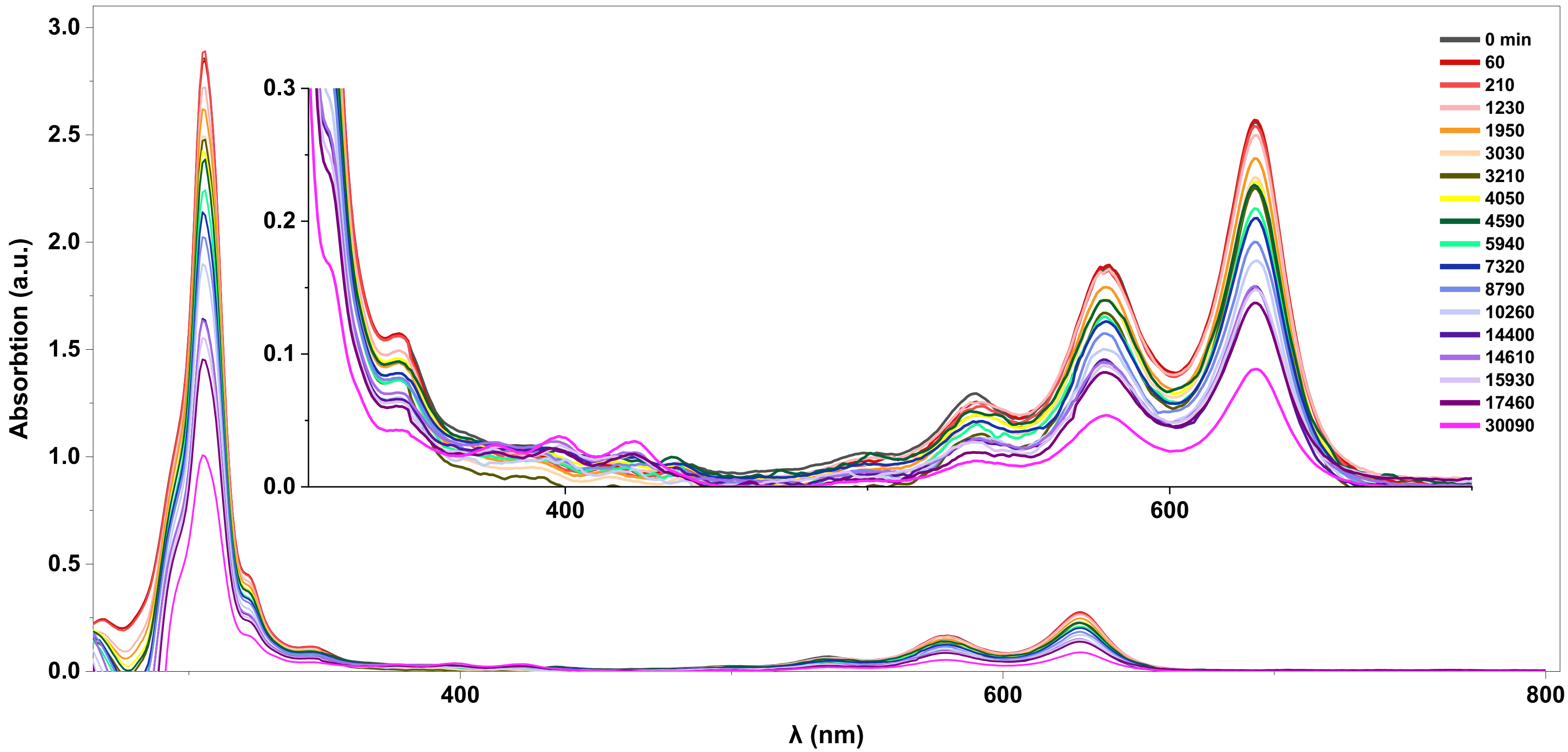

Figure S27. UV-vis absorbance-time profile of **7c(F8)**, in BHT stabilized THF over a period of 502 hours. Inset shows expansion along the x- and y-axis (A_t/A_0). Averages of two trials were used, and the difference between the two measurements was less than ±10% in all cases.

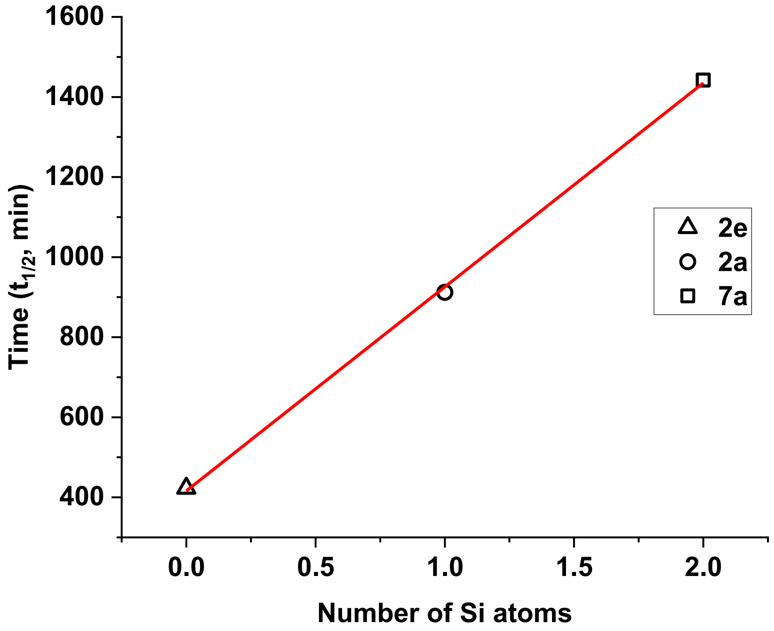

Figure S28. Relationship between the number of silicon atoms present in a molecule and the associated half-life ($t_{1/2}$).

Studies of Binary-Film Stability of 2a and 2e Mixed with PCBM

UV-vis Spectroscopic Analysis: A volumetric flask was charged with BHT stabilized THF (200 mL, freeze-pump-thaw degassed, three cycles, N_2), a sample of pentacene derivative (either **2a** or **2e**, Table S2) was added, and the solutions placed in the dark. A UV-vis sample was taken from each volumetric flask. PCBM was added to the remaining solution of **2a** or **2e** in each of the volumetric flasks, and the resulting solution was mixed thoroughly. Two aliquots from each volumetric flask were then taken. One aliquot was placed in a cuvette, capped, and used as a solution-state reference. The second aliquot was placed in a round-bottomed flask and the solvent evaporated to dryness under an N_2 stream. The resulting film was then placed under high vacuum to ensure effective desolvation. The solution-state reference sample was analyzed by UV-vis spectroscopy immediately (Sample **PCBM & Pentacene**, Figures S29–32) and then wrapped fully in aluminum foil to exclude light. The reference was analyzed again just before analyzing (Sample **Pentacene**, Figure S29). The solution of pentacene and PCBM was analyzed prior to evaporation to verify short term stability in solution (Sample **PCBM & Pentacene-40min**, Figures S29–32). The film was redissolved with an approximately equivalent volume of degassed THF and UV-vis spectra were obtained (Sample **Redissolved Film**, Figures S29–32).

Table S2. Masses of pentacene derivatives **2a** and **2e** utilized for UV-vis binary film study with PCBM.

	Mass of acene (mg)	***Mass of PCBM (mg)***
2a	2.41	4.39
2a	1.58	1.07
2e	4.12	5.25
2e	1.57	1.37

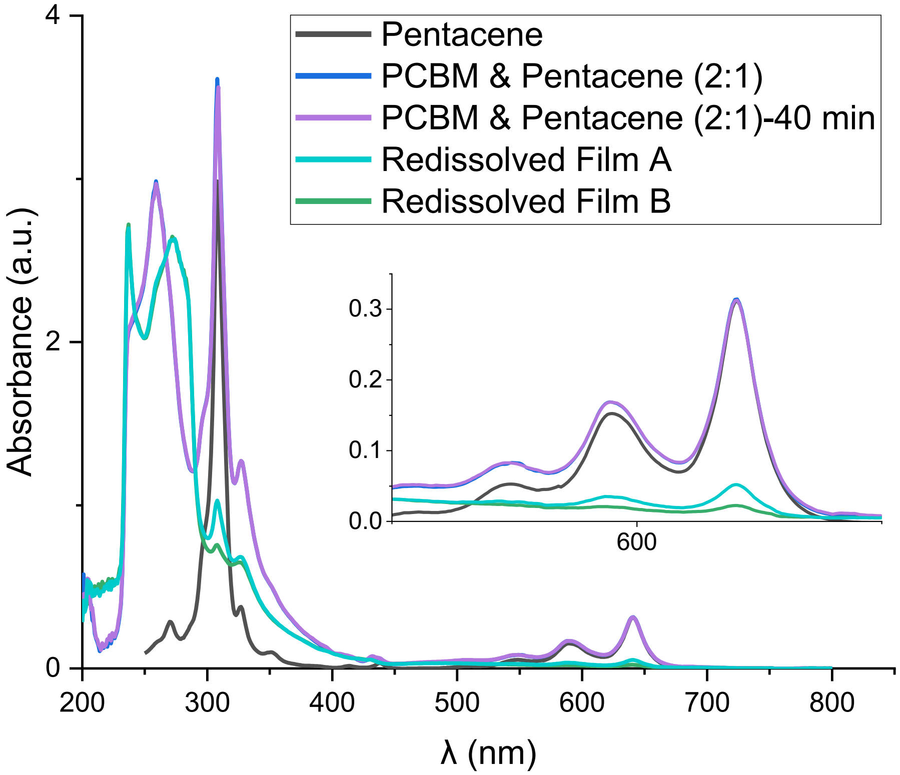

Figure S29. UV-vis analysis of donor-acceptor binary films; Compound **2a** 1:2 with PCBM in THF: solution-state reference sample (blue), solution-state reference at time of film analysis (purple), and solution of redissolved film samples (teal/green). The spectrum of **2a** is shown for comparison (black).

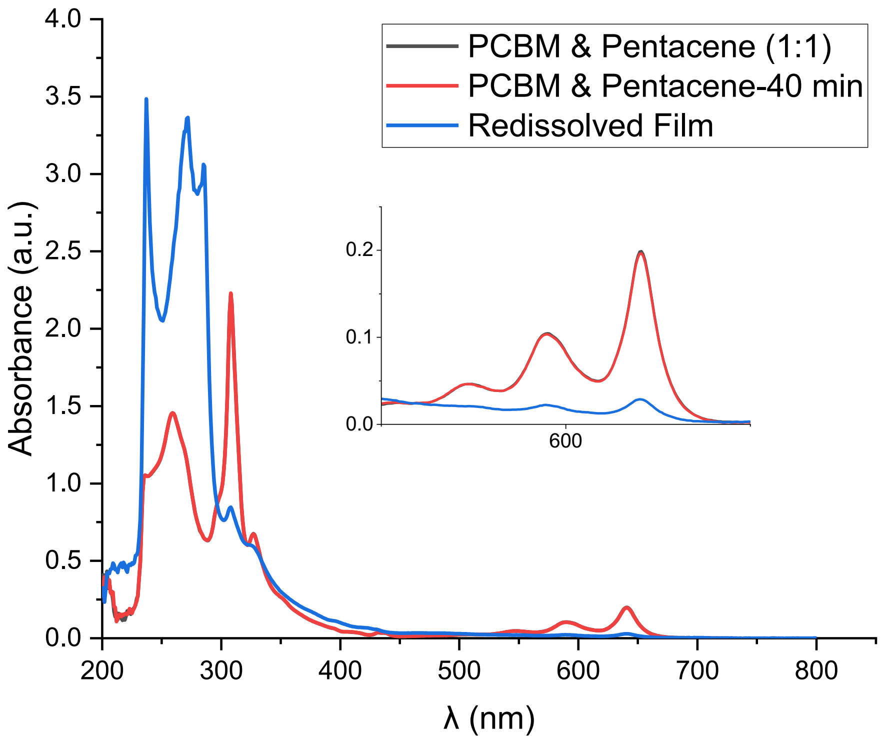

Figure S30. UV-vis analysis of donor-acceptor binary films; Compound **2a** 1:1 with PCBM in THF: solution-state reference sample (black), solution-state reference at time of film analysis (red), and solution of redissolved film samples (blue). The spectrum of **2a** is shown for comparison (black).

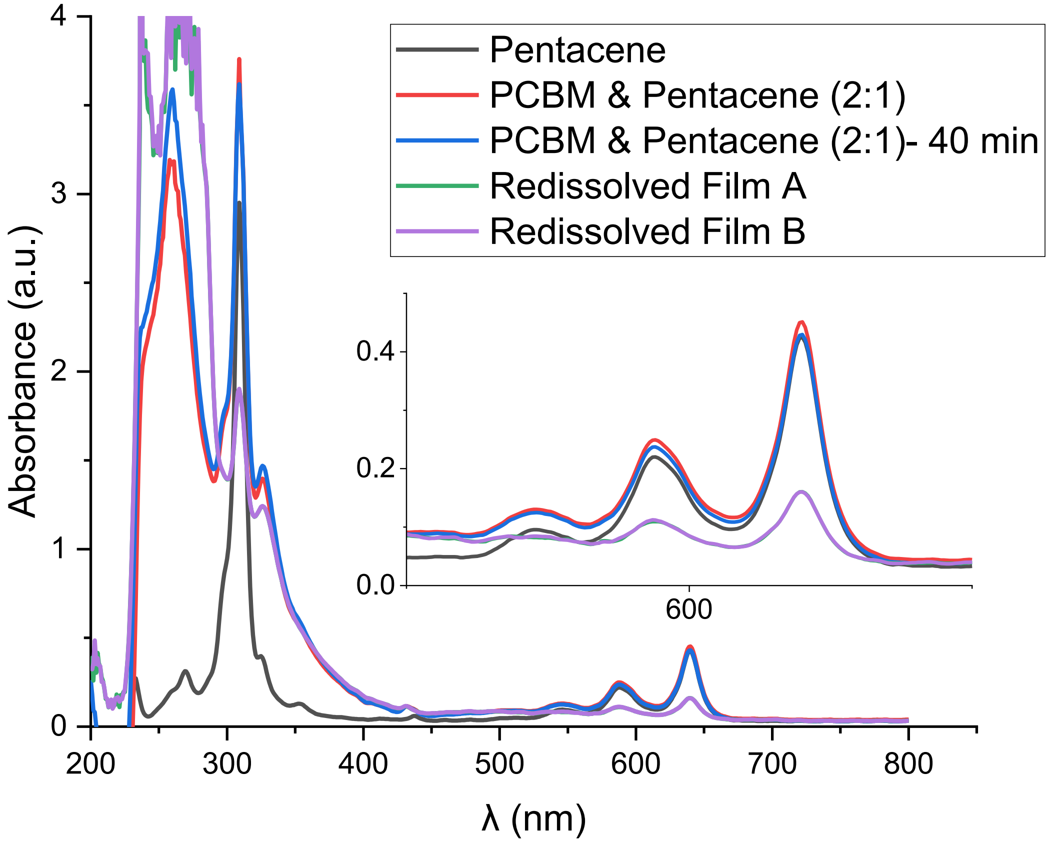

Figure S31. UV-vis analysis of donor-acceptor binary films; Compound **2e** 1:2 with PCBM.

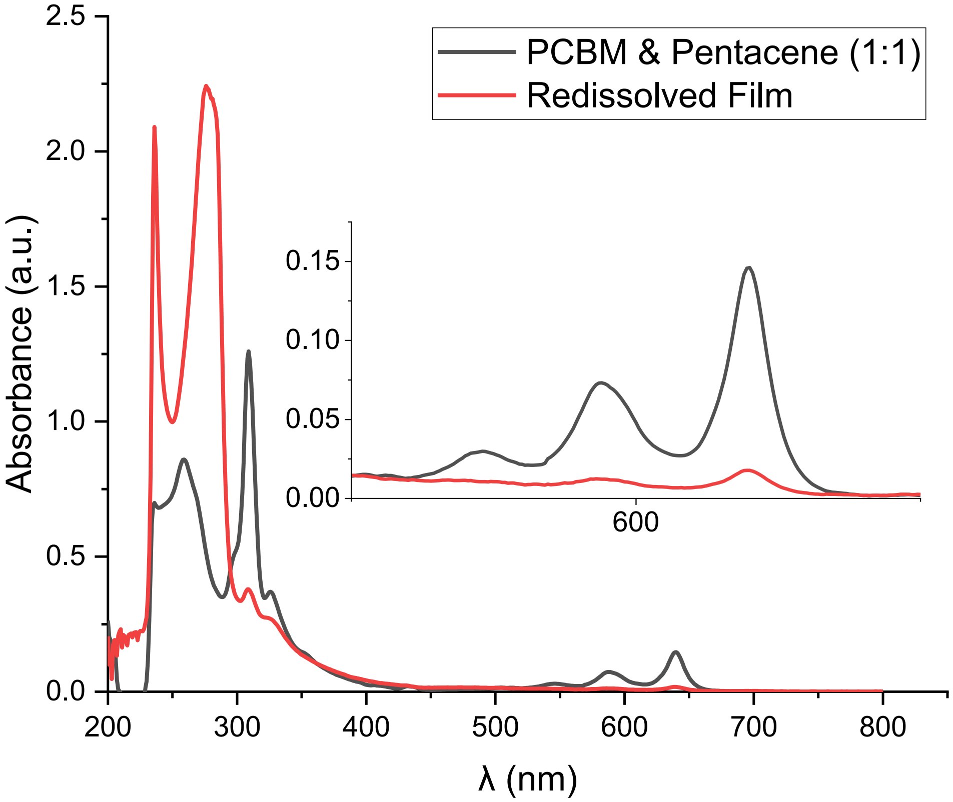

Figure S32. UV-vis analysis of donor-acceptor binary films; Compound **2e** 1:1 with PCBM in THF (black), film redissolved (red).

NMR Spectroscopic Analysis: The solvent (C_6D_6) was freeze-pump-thaw degassed over three cycles. Samples of PCBM and pentacene (**2a**, **2e**) were weighted in ca. 1:1 stoichiometry and added to a dry flask under N_2. The solvent C_6D_6 was added (1.5 mL). Once the solution was homogeneous, half of the original volume was transferred to an NMR tube and analyzed via ^{1}H NMR spectroscopy. The remaining volume of solvent was removed under a constant stream of N_2 until a film was formed. Once dry to ensure residual solvent was removed the film was placed under high vacuum. The film was then redissolved in ca. 0.75 mL of C_6D_6 and the solution was analyzed via ^{1}H NMR spectroscopy.

Control Reactions: To demonstrate that the decomposition was not a result of PCBM or pentacene components reacting with itself upon film formation controls were conducted where PCBM and pentacene were separately dissolved in C_6D_6 and films were generated as described above. The redissolved samples were then analyzed by ^{1}H NMR spectroscopy **Figure S33**–**S34**.

Table S3. Masses of pentacene derivatives **2a**, **2e**, and PCBM were utilized for the NMR spectroscopy binary film study.

	Mass of acene (mg)	***Mass of PCBM (mg)***
2a	1.15	0.97
2e	0.80	0.65

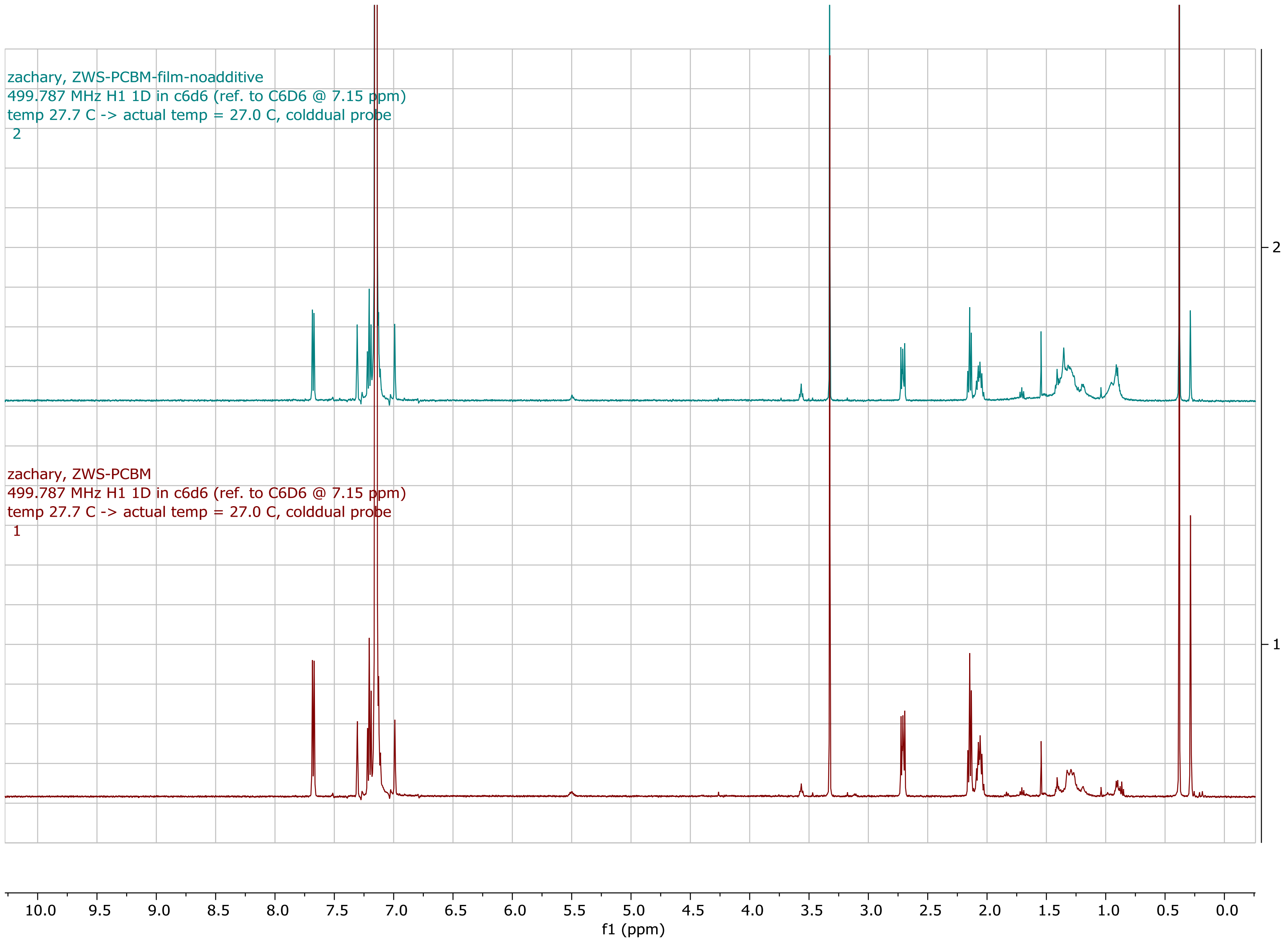

Figure S33. Control experiment analyzing a solvent cast film of PCBM independent of the donor pentacene. Top (film redissolved), bottom (initial solution).

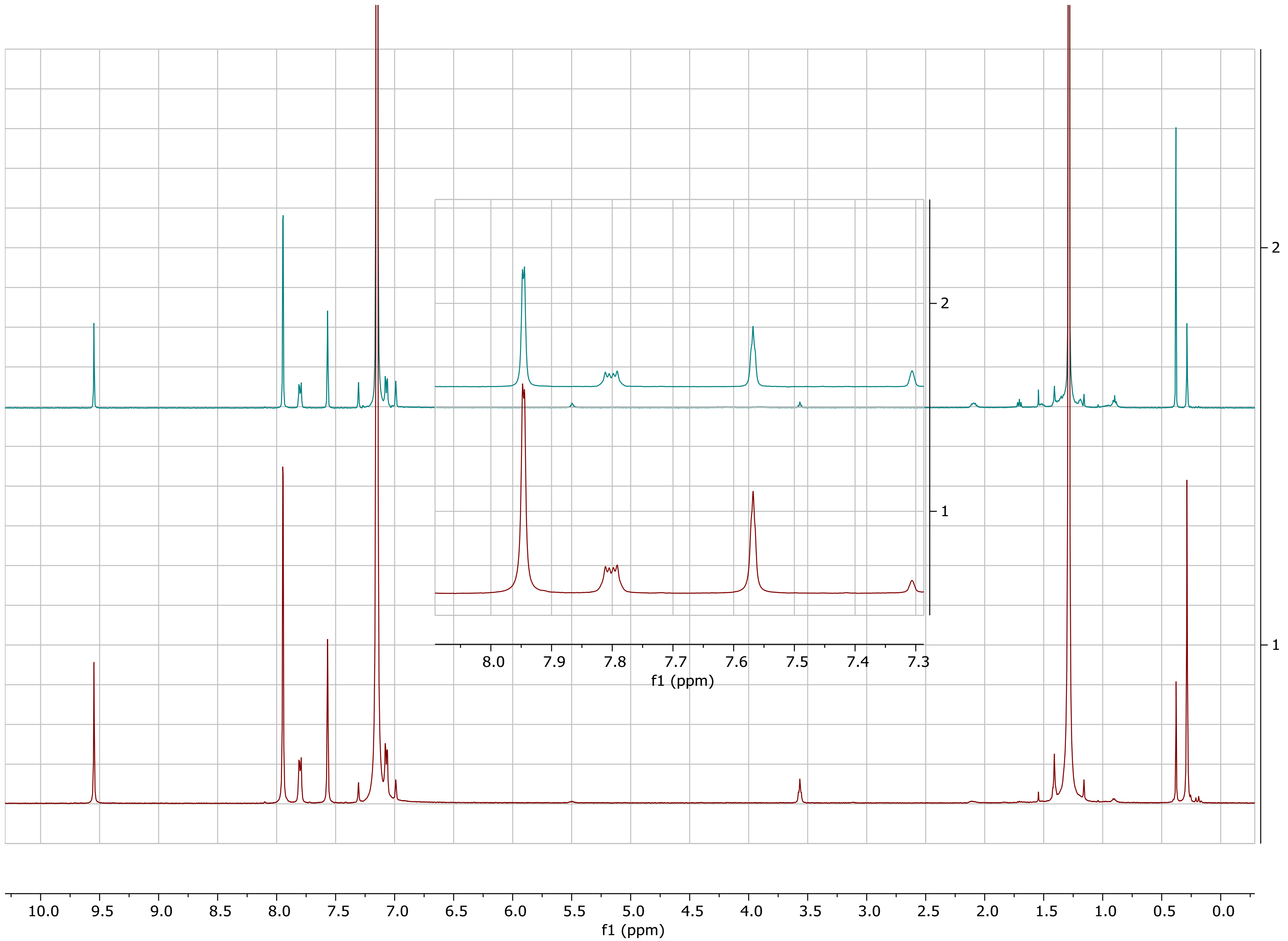

Figure S34. Control experiment analyzing a solvent cast film of **2e** independent of acceptor PCBM. Top (film redissolved), bottom (initial solution). Inset shows expansion of the aromatic region of the NMR spectra.

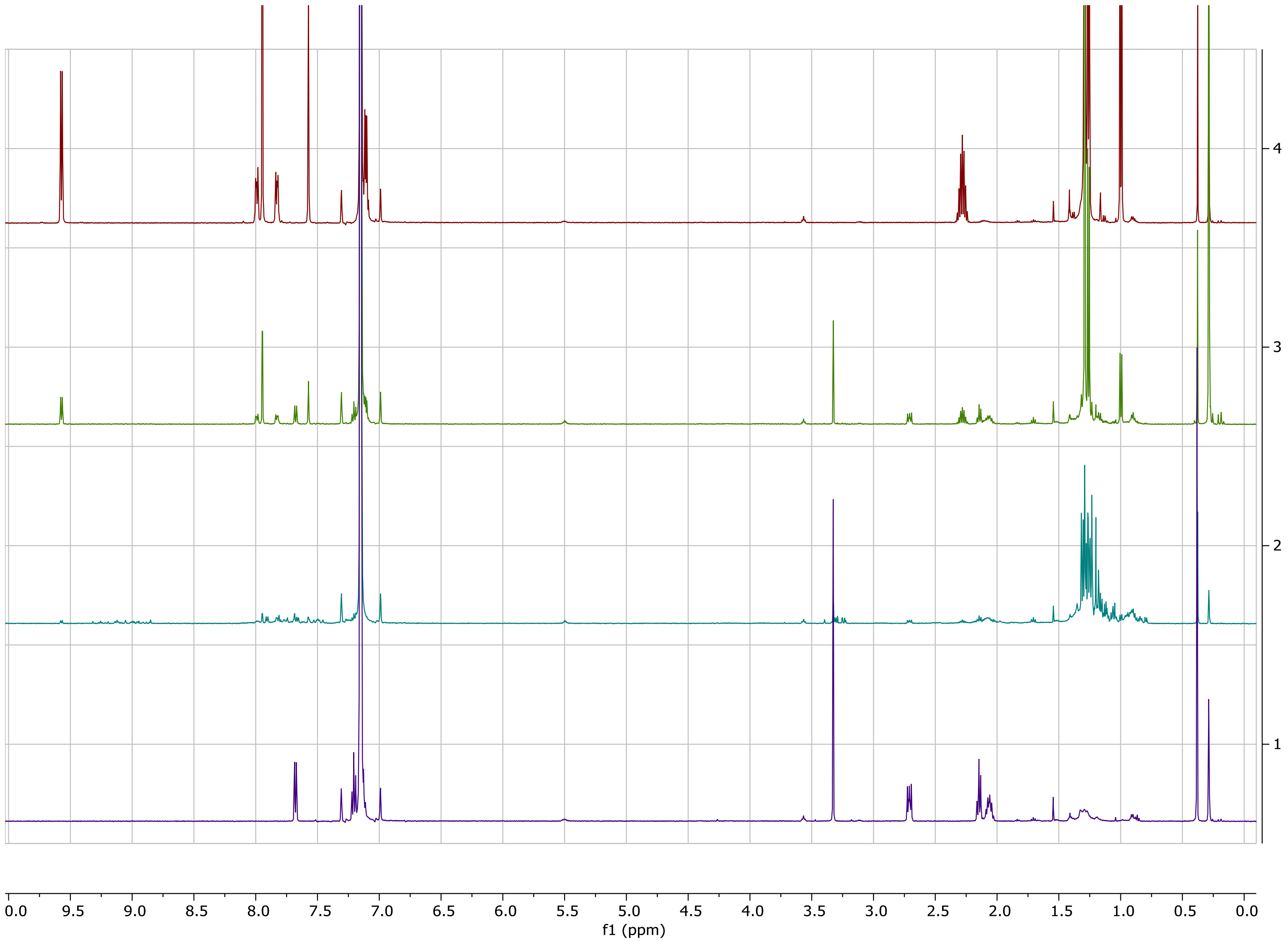

Figure S35. Compound **2a** (red); PCBM solution with **2a** (green); Redissolved film of **PCBM/2a** (teal); PCBM (purple) in C_6D_6.

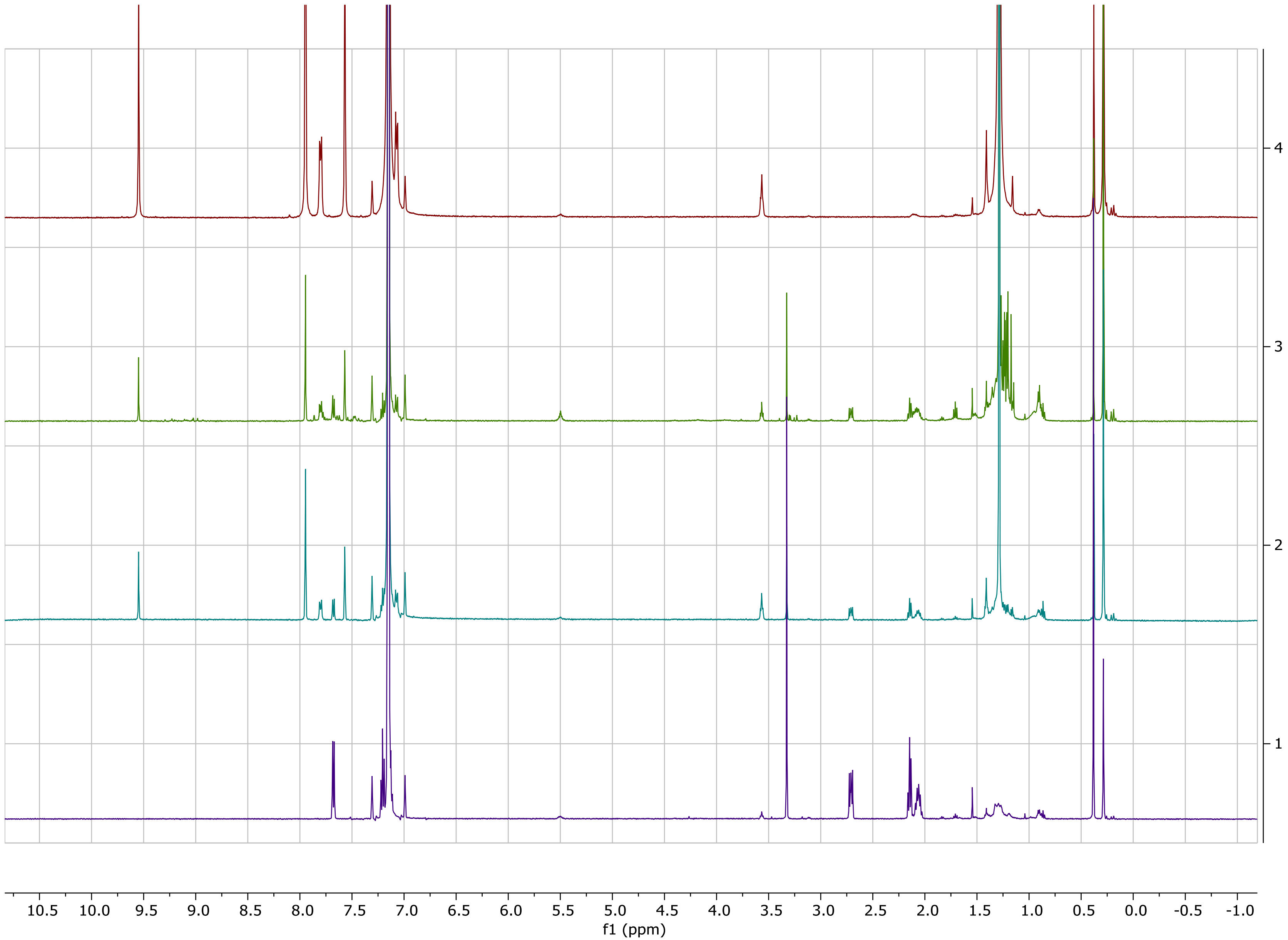

Figure S36. Compound **2e** (red); PCBM solution with **2e** (teal); Redissolved film of **PCBM/2a** (green); PCBM (purple) in C_6D_6.

^{1}H, ^{19}F, and ^{13}C NMR Spectra of Compounds 2a–e, 3c–d, 3b(F8), 5a–e, 5b(F8), 5e(F8), 2e(F8), 7b(F8), 7c(F8), and 7a

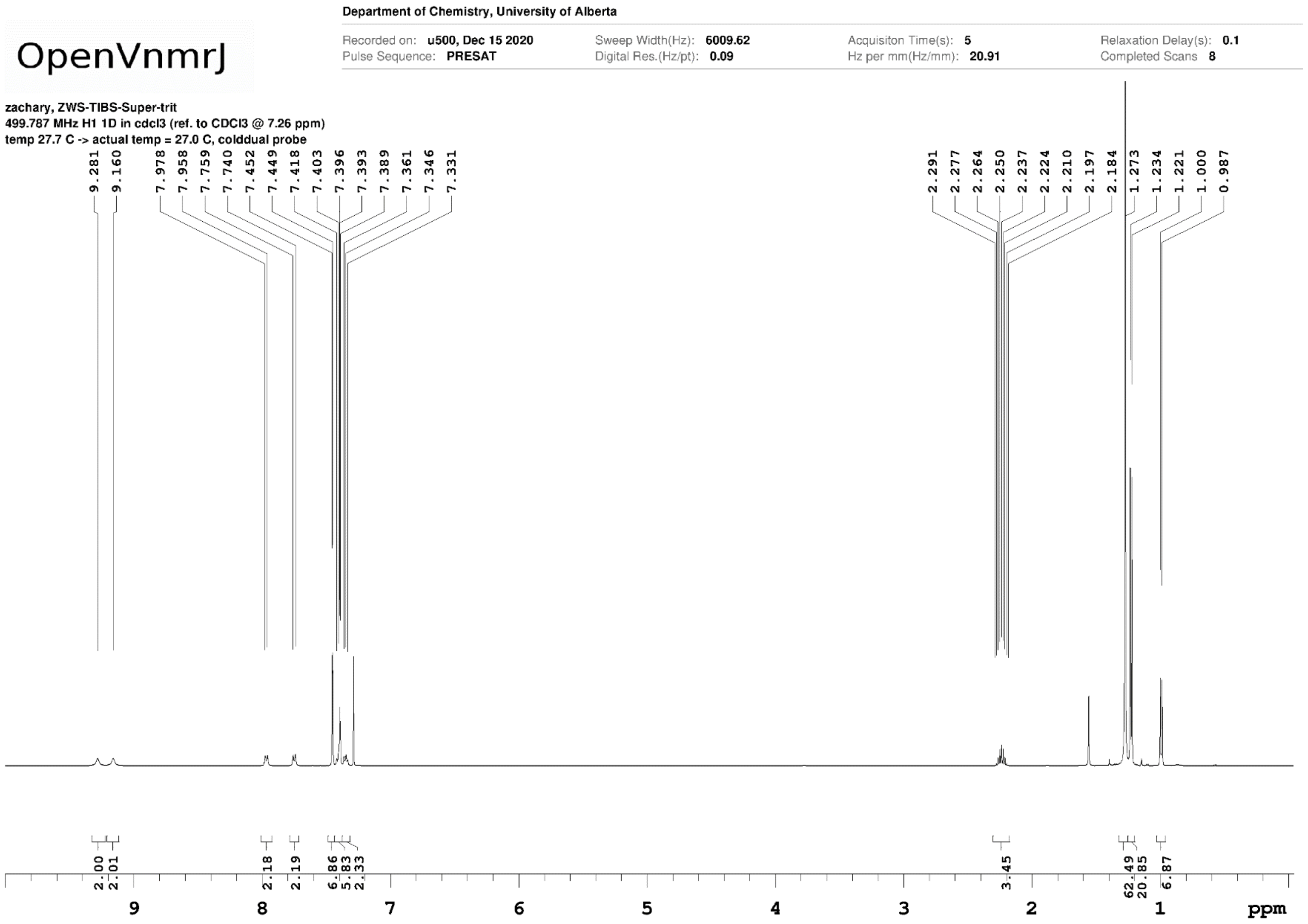

Figure S37. ^{1}H NMR spectrum of compound **2a**, 500 MHz, $CDCl_3$.

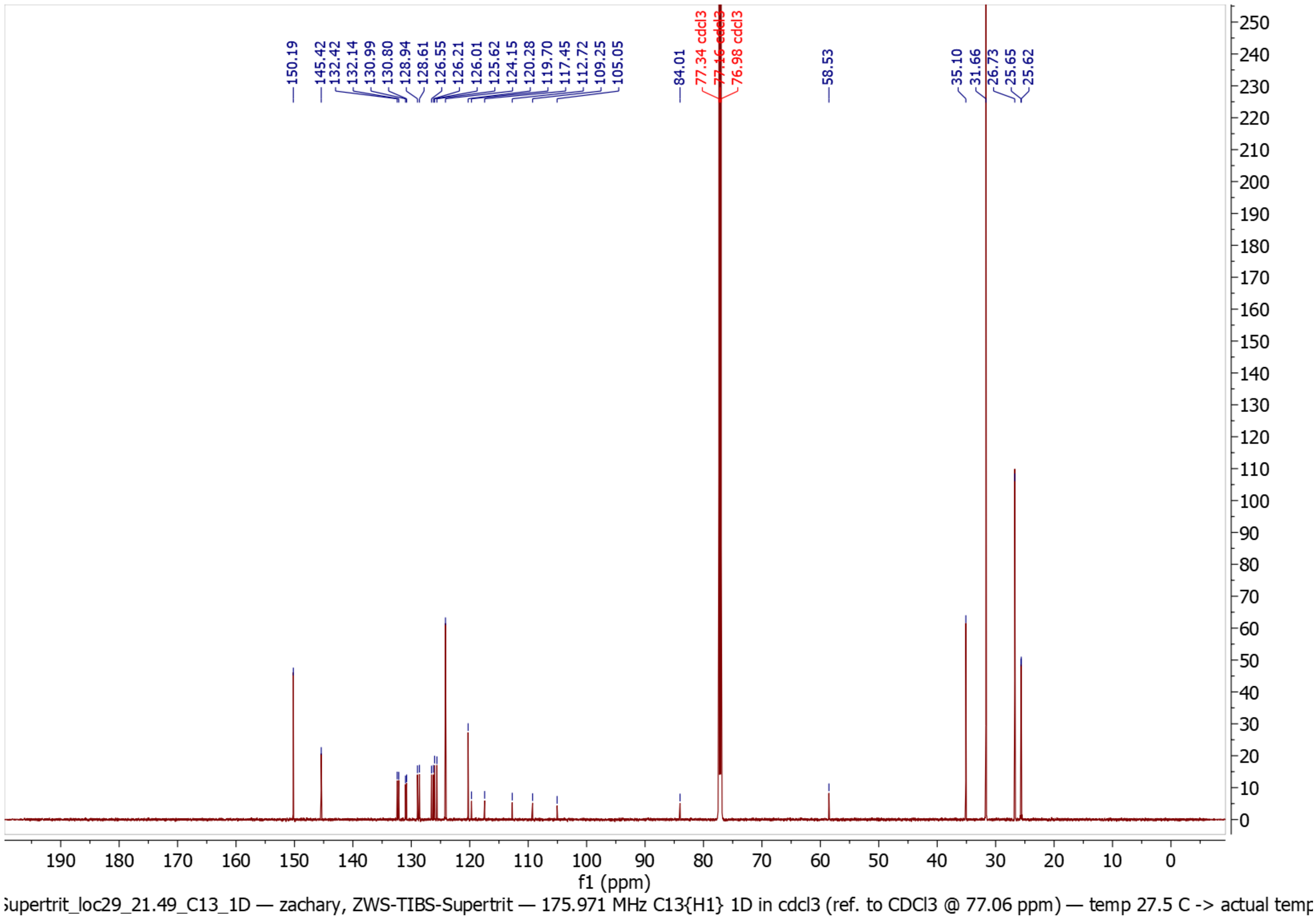

Figure S38. ^{13}C NMR spectrum of compound **2a**, 176 MHz, $CDCl_3$.

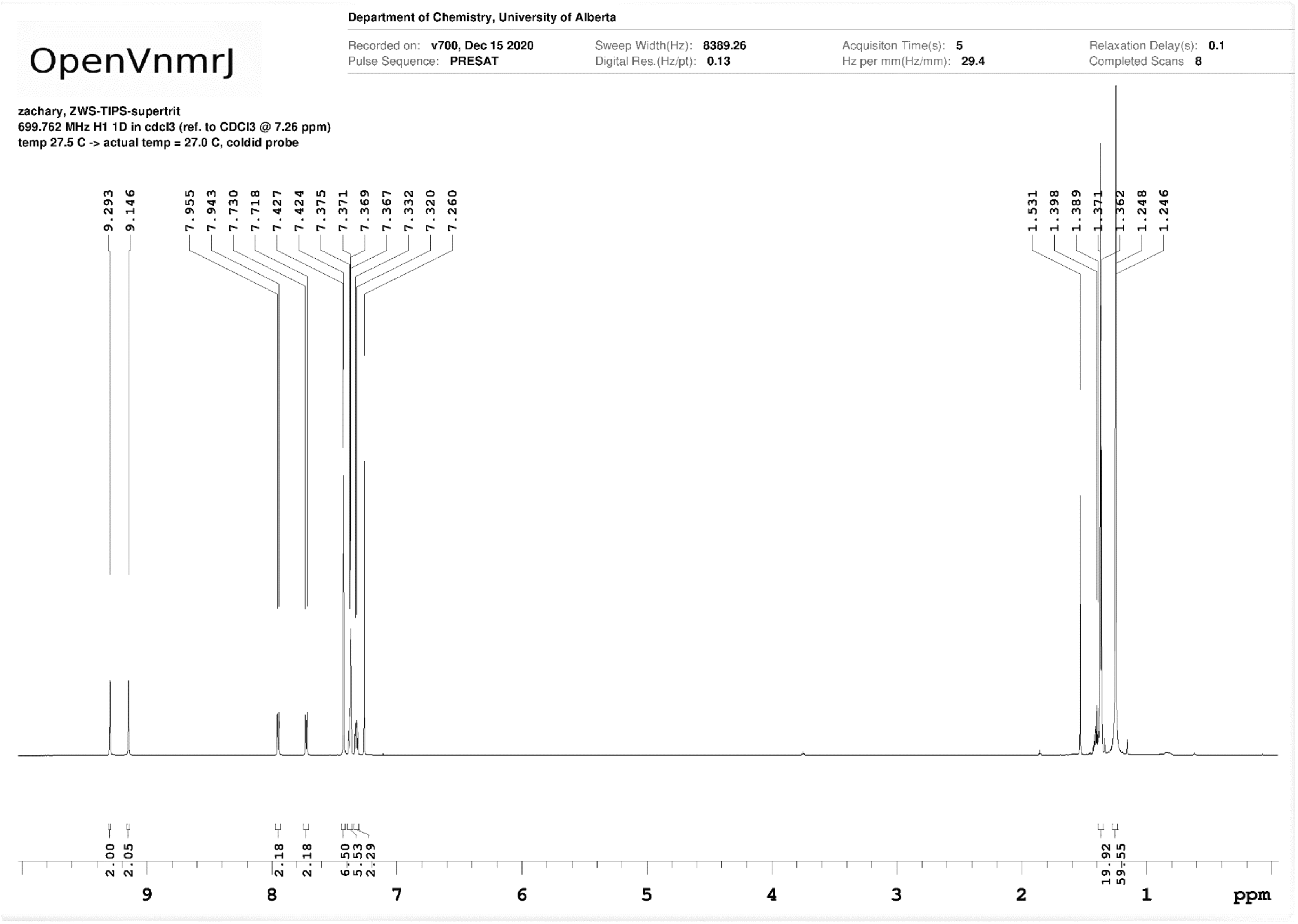

Figure S39. ^{1}H NMR spectrum of compound **2b**, 700 MHz, $CDCl_3$.

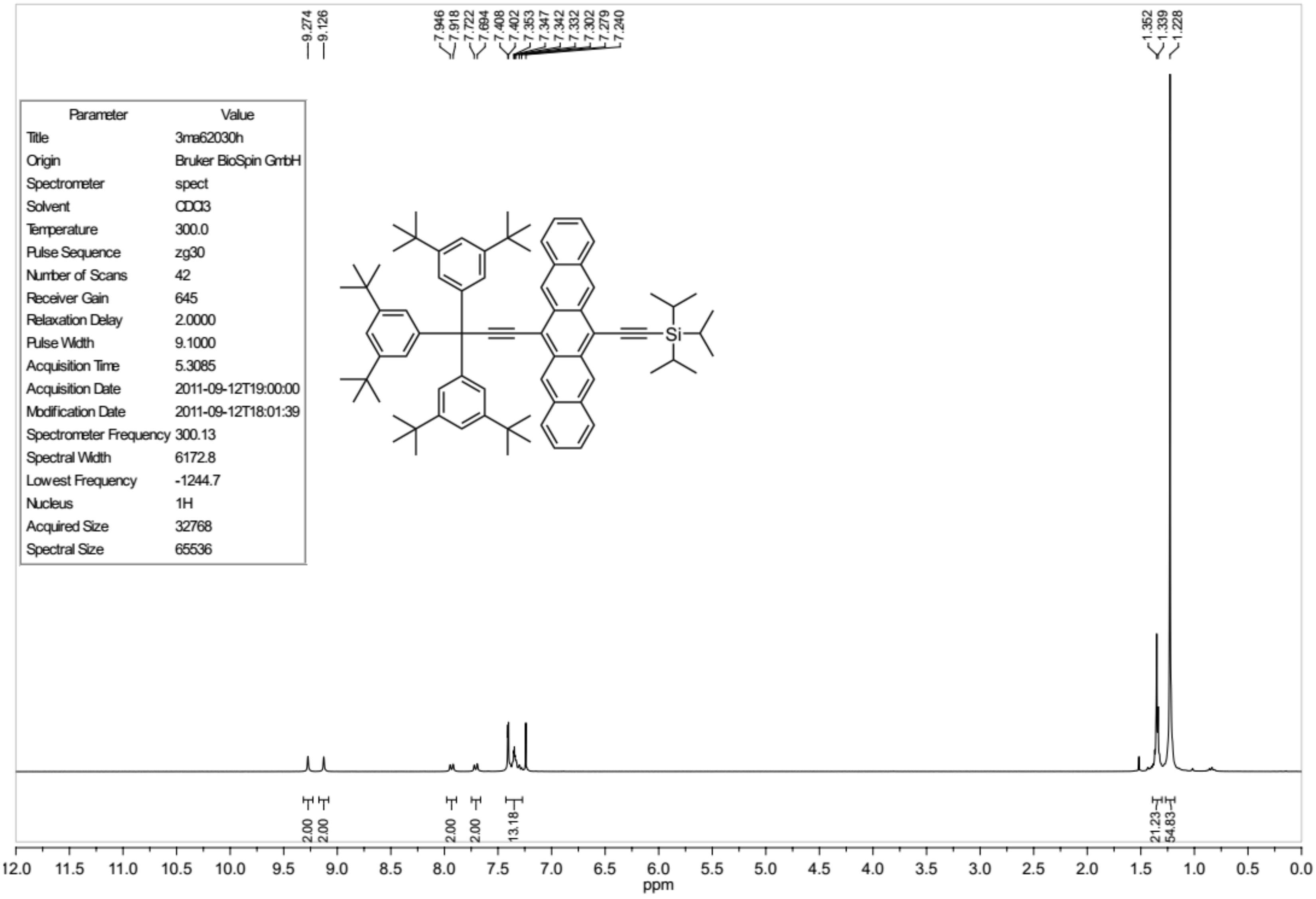

Figure S40. ^{1}H NMR spectrum of compound **2b**, 300 MHz, $CDCl_3$.

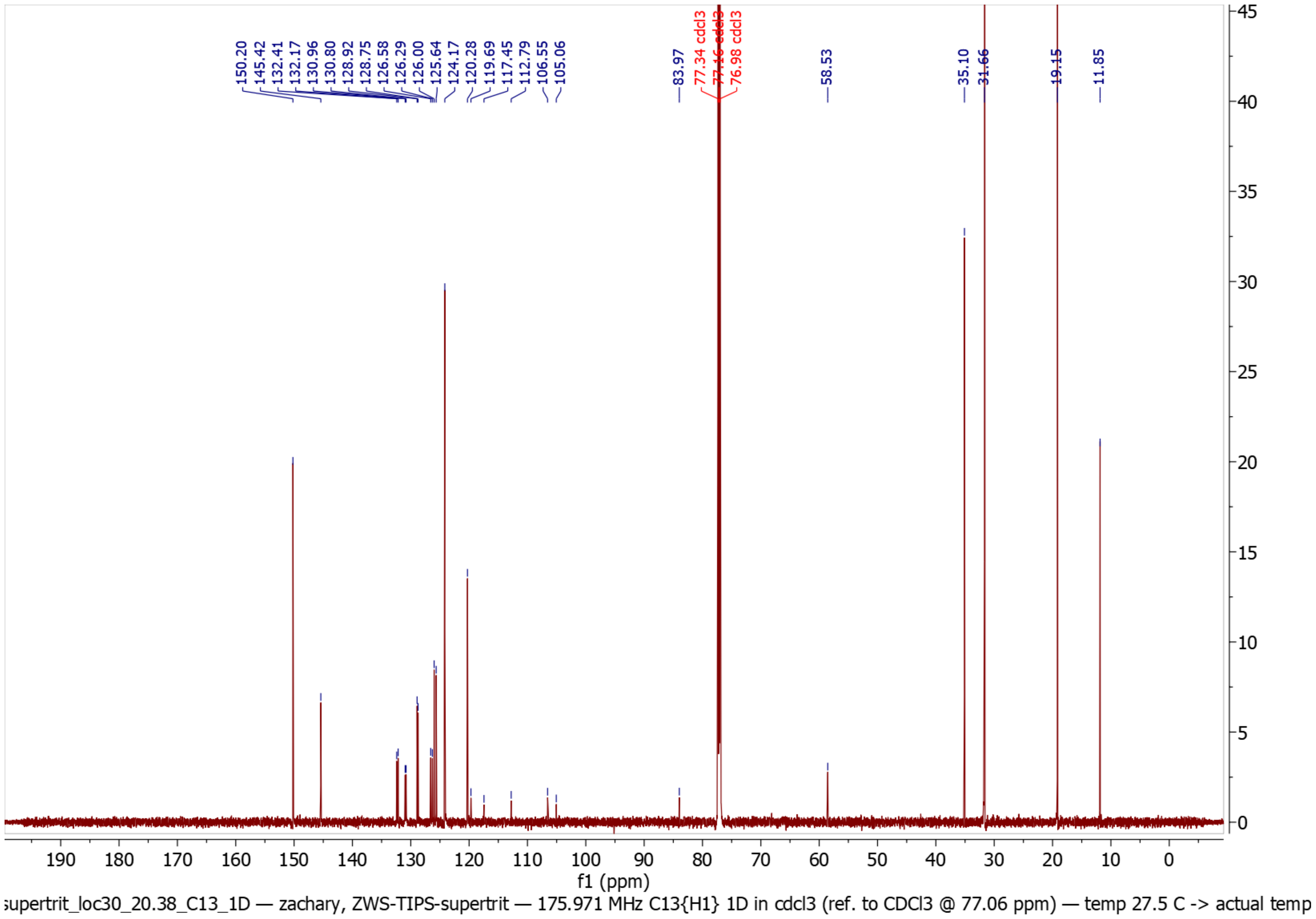

Figure S41. ^{13}C NMR spectrum of compound **2b**, 175 MHz, $CDCl_3$.

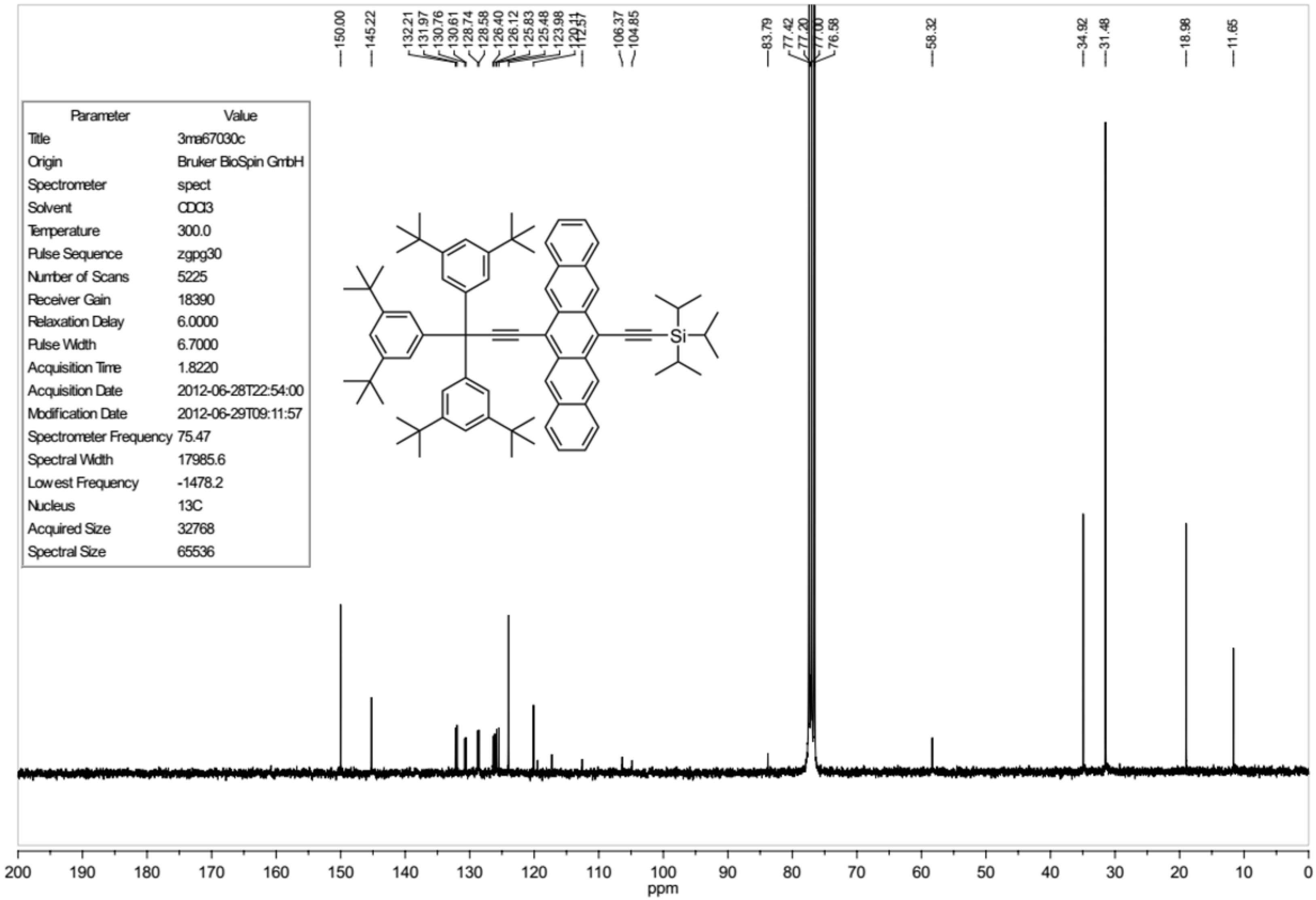

Figure S42. ^{13}C NMR spectrum of compound **2b**, 75 MHz, $CDCl_3$.

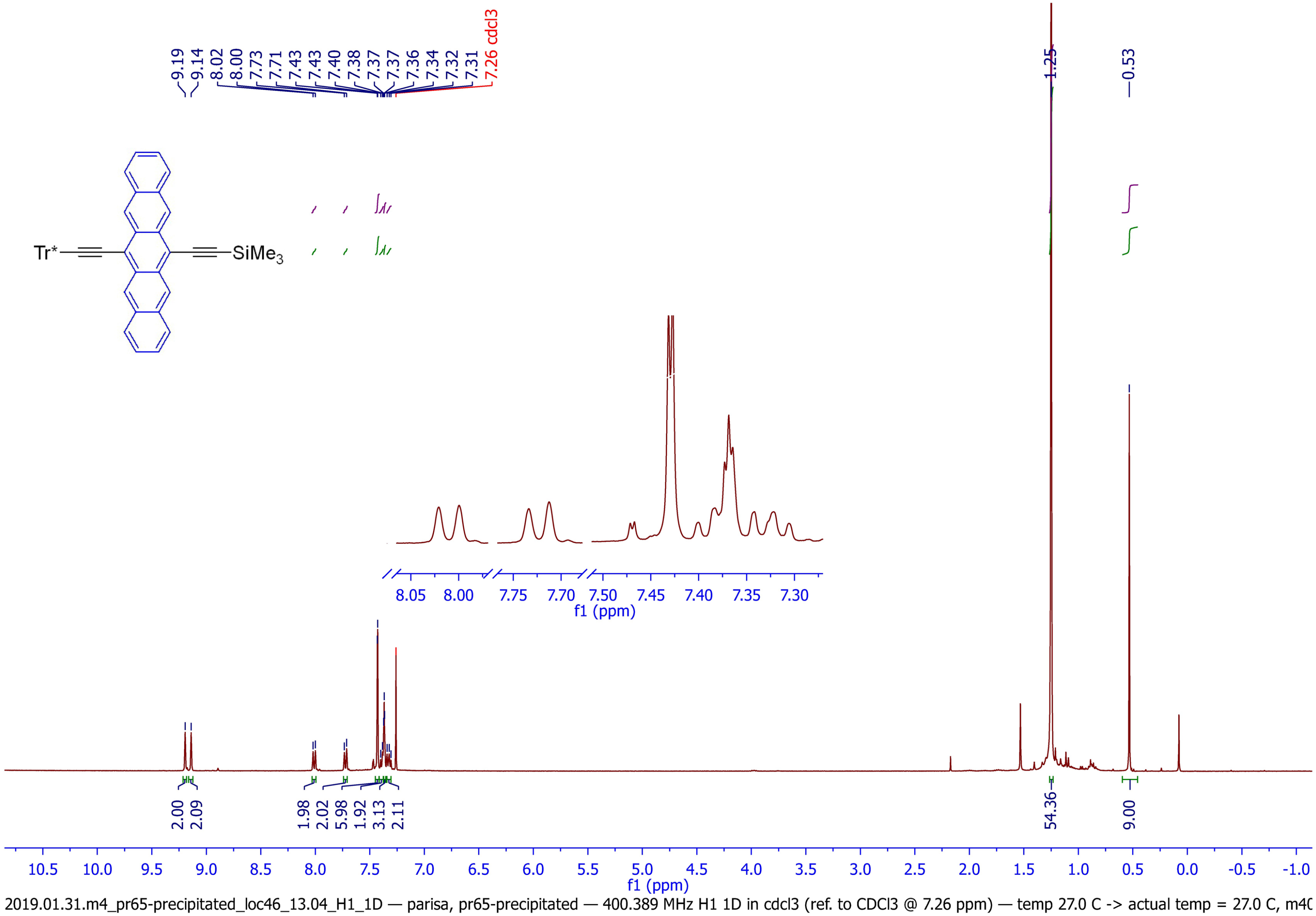

Figure S43. ^{1}H NMR spectrum of compound **2c**, 400 MHz, $CDCl_3$.

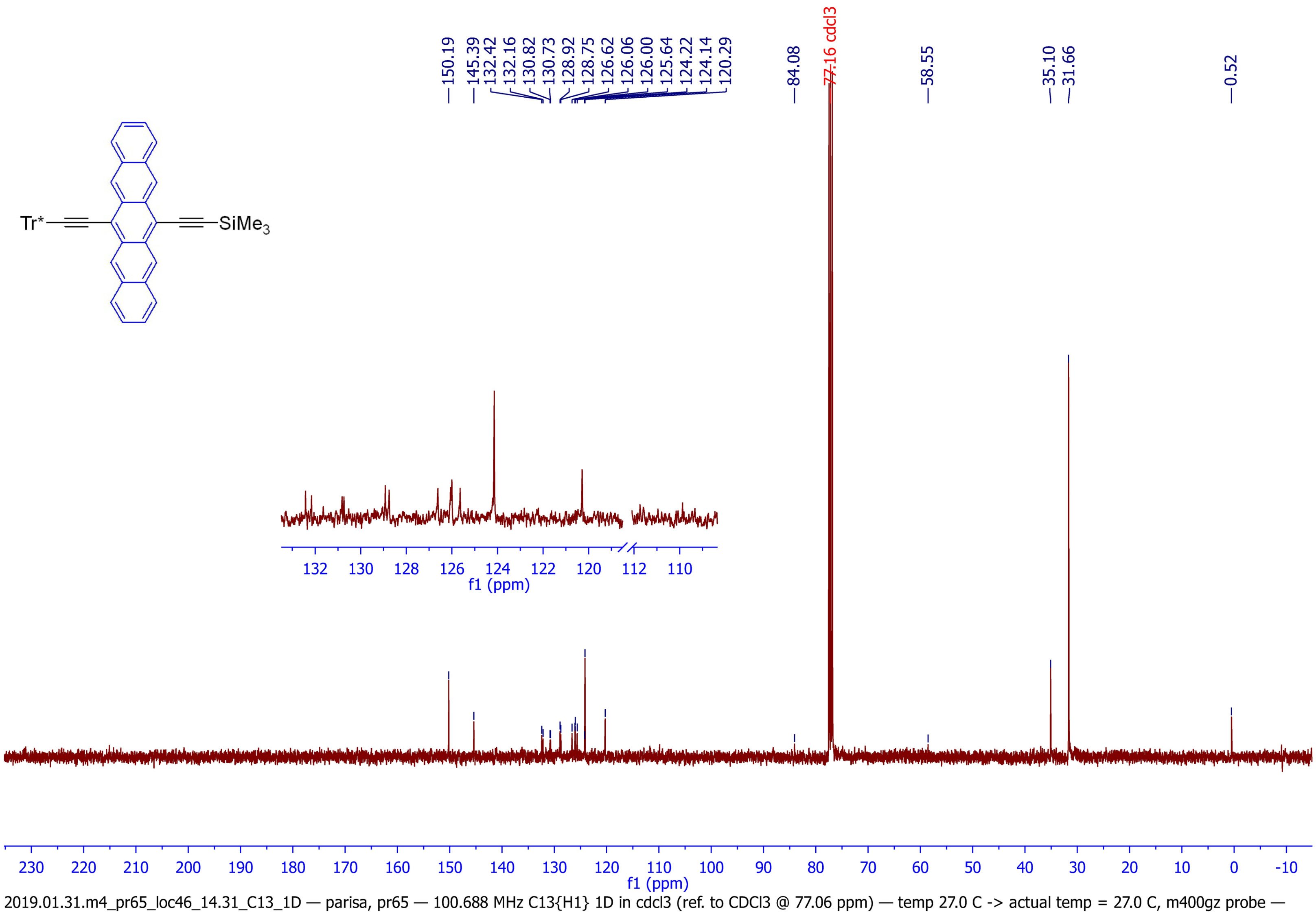

Figure S44. ^{13}C NMR spectrum of compound **2c**, 100 MHz, $CDCl_3$.

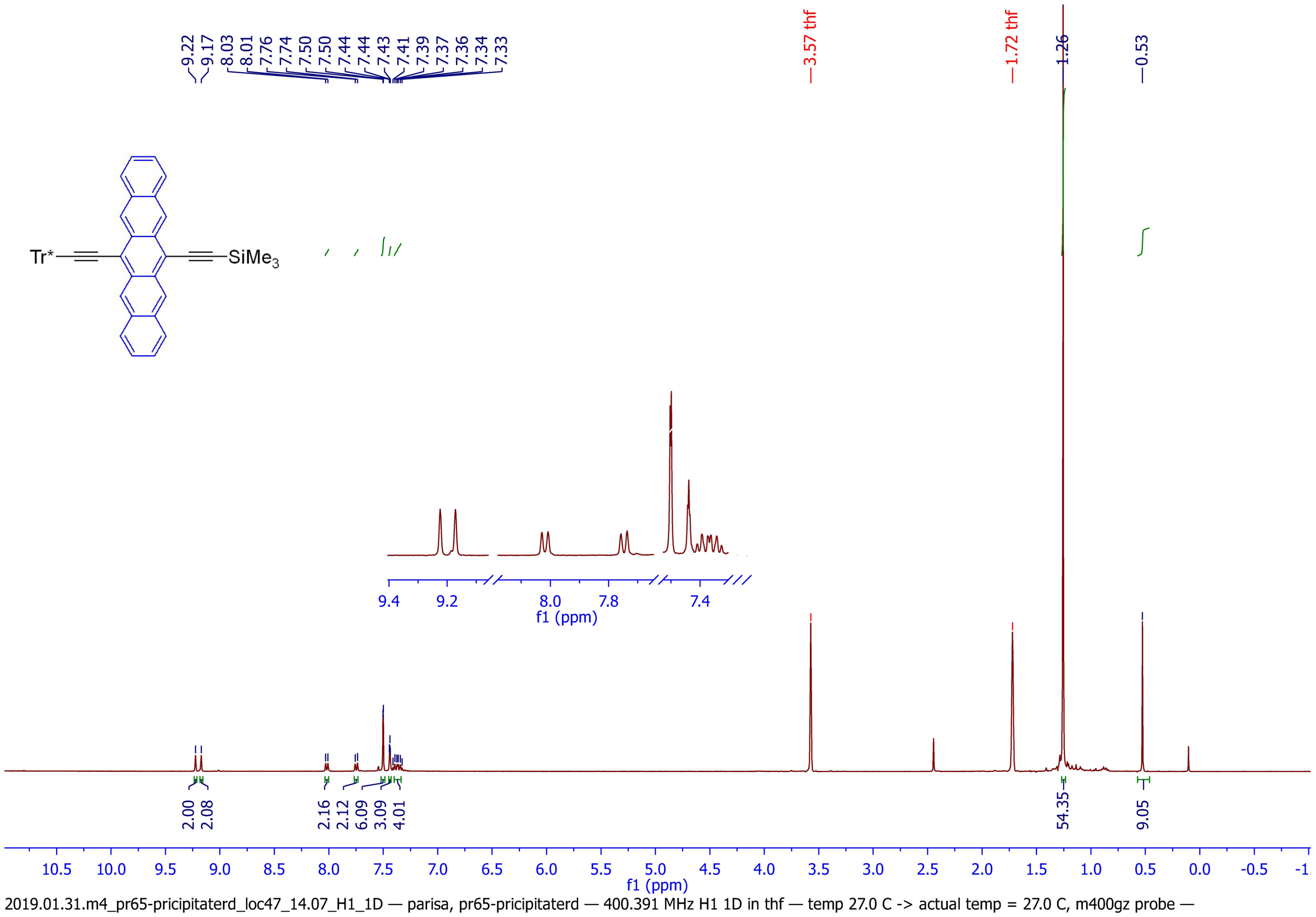

Figure S45. ^{1}H NMR spectrum of compound **2c**, 400 MHz, THF-d_8.

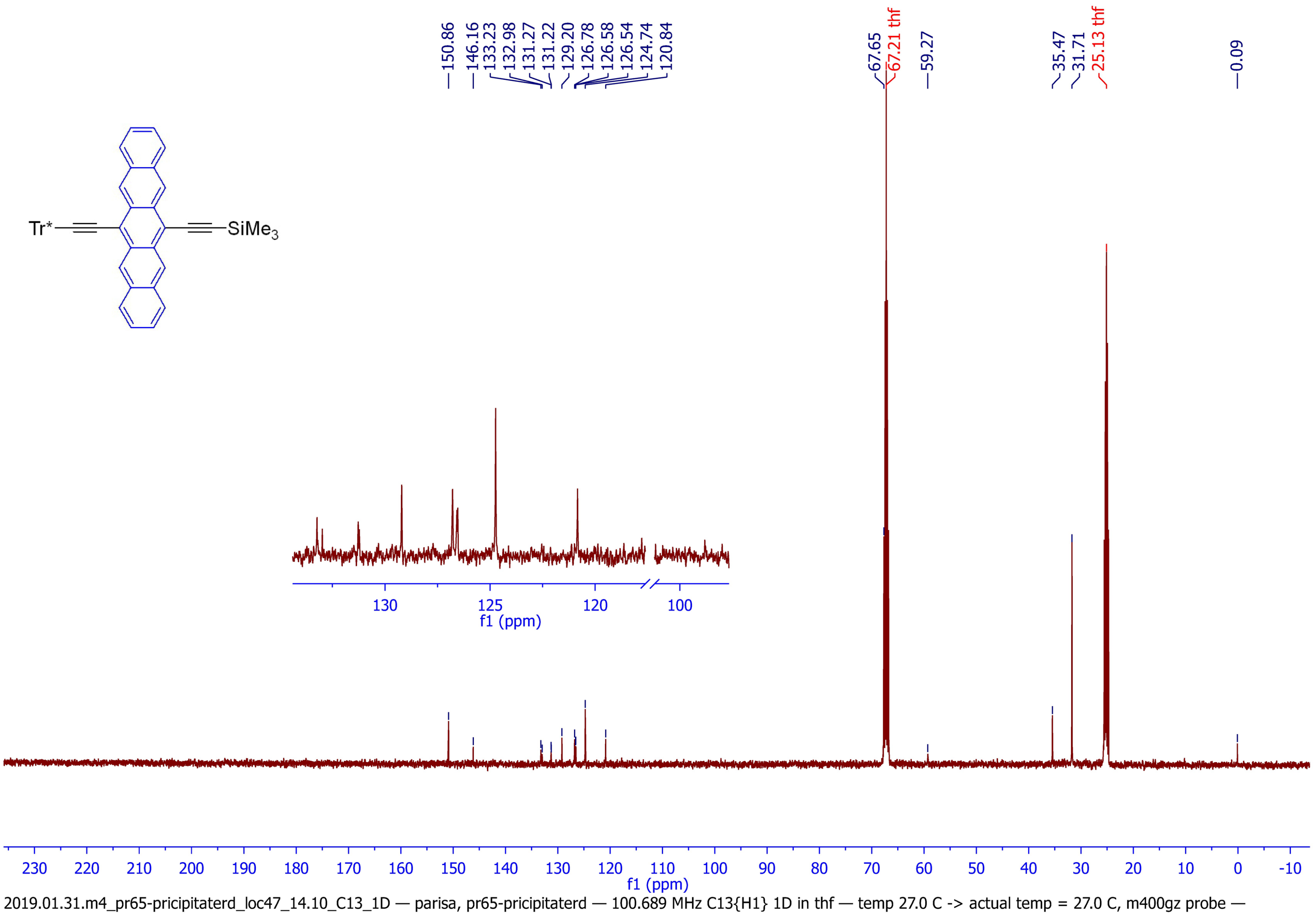

Figure S46. ^{13}C NMR spectrum of compound **2c**, 100 MHz, THF-d_8.

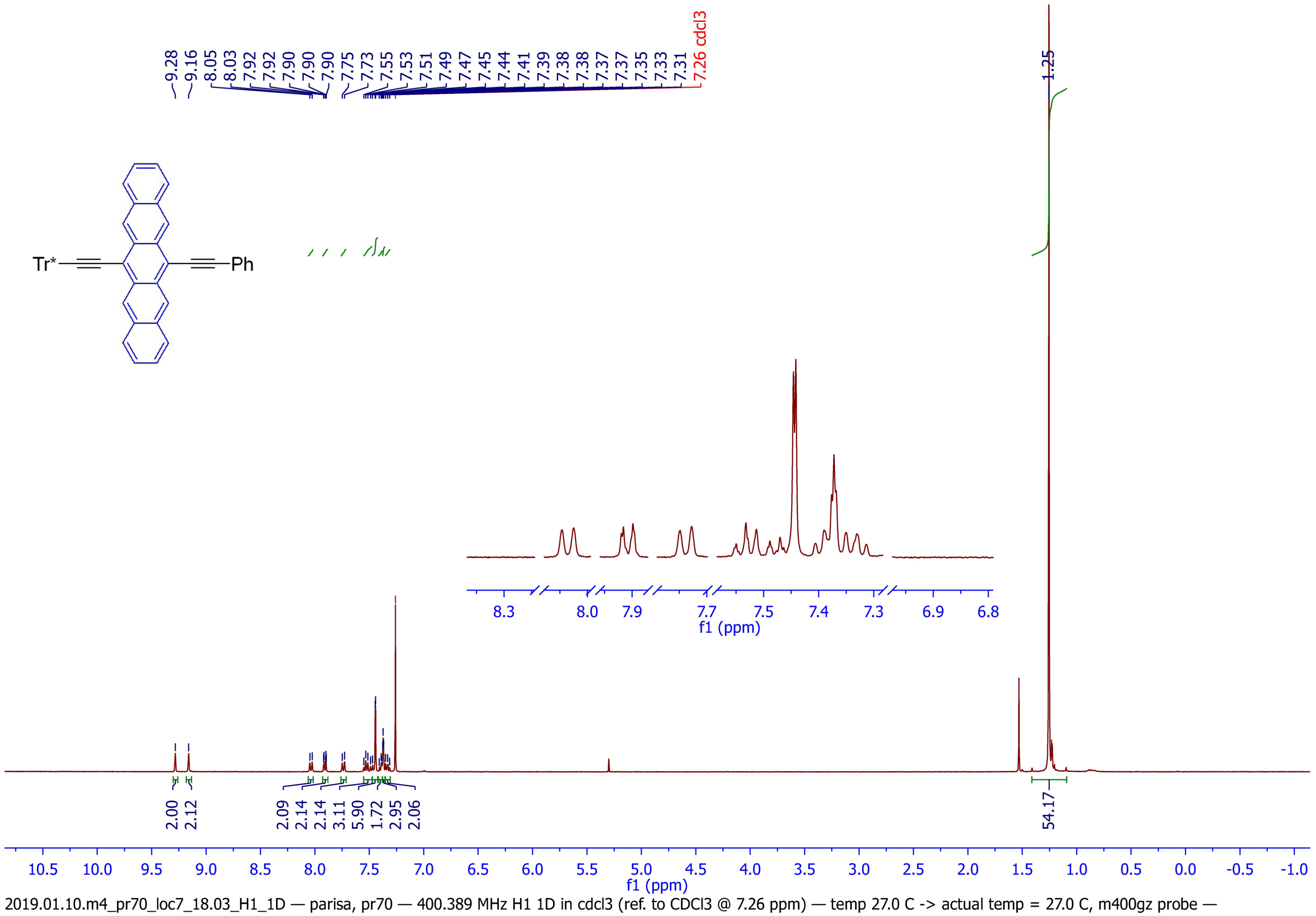

Figure S47. ^{1}H NMR spectrum of compound **2d**, 400 MHz, $CDCl_3$.

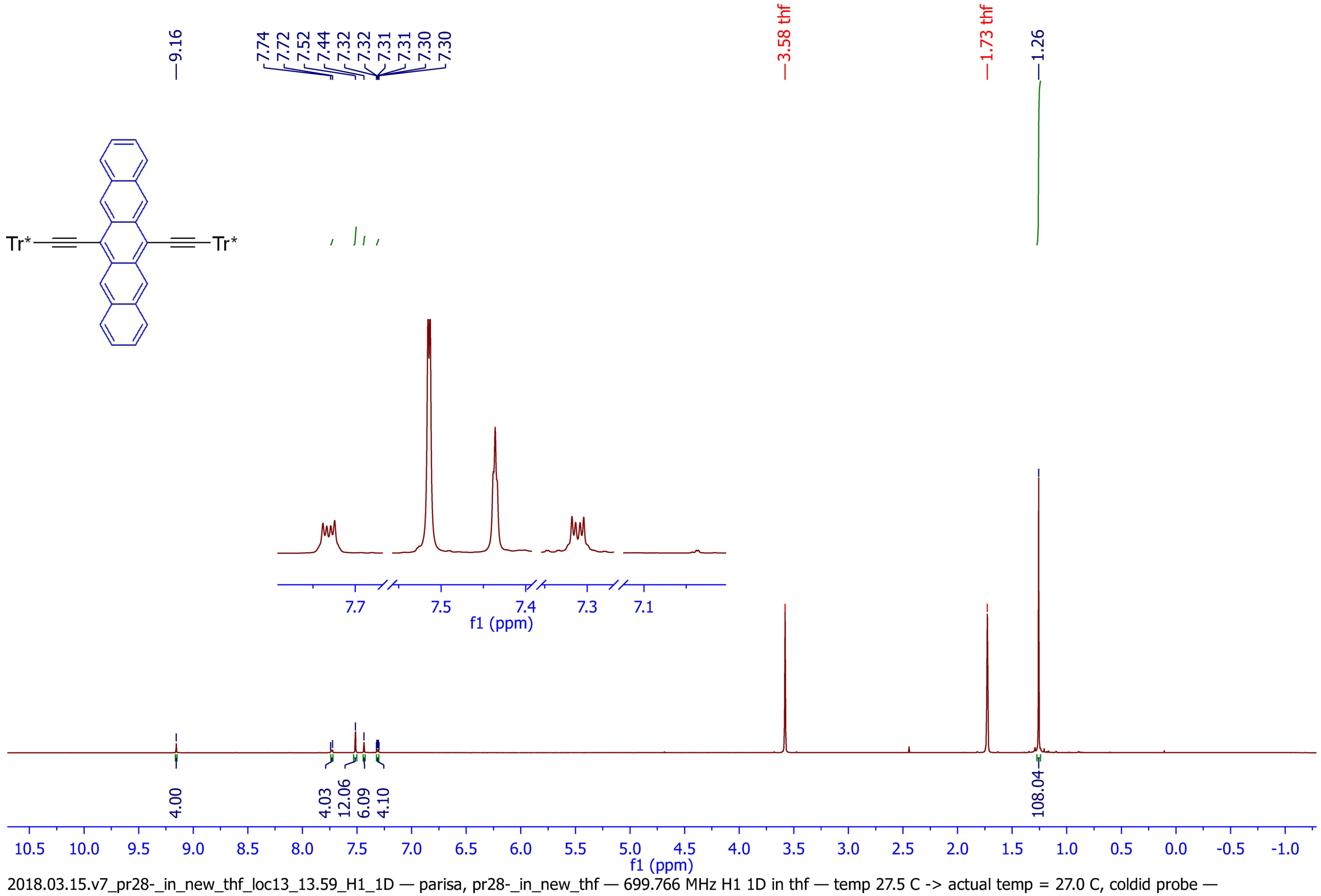

Figure S48. ^{1}H NMR spectrum of compound **2e**, 700 MHz, THF-d_8.

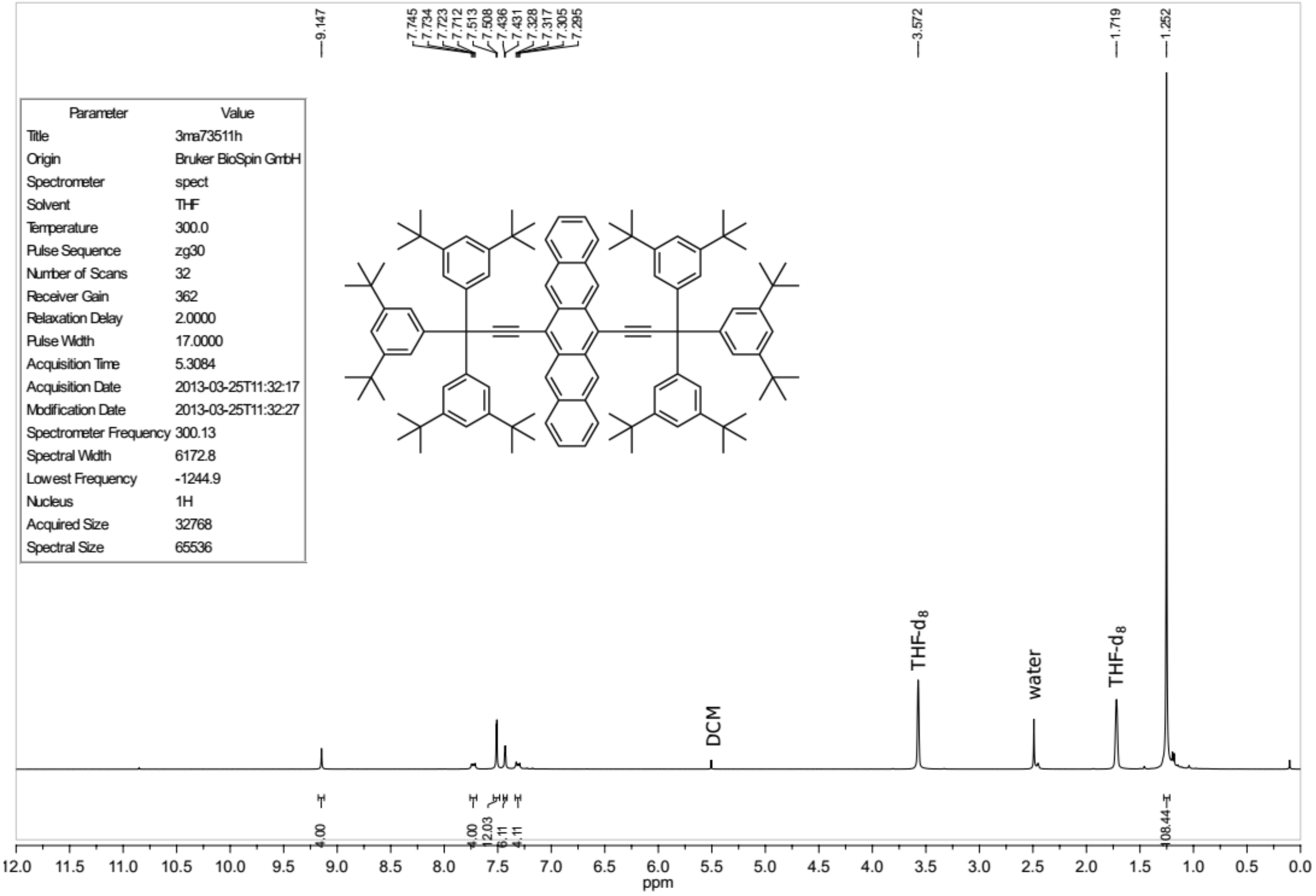

Figure S49. ^{1}H NMR spectrum of compound **2e**, 300 MHz, THF-d_8.

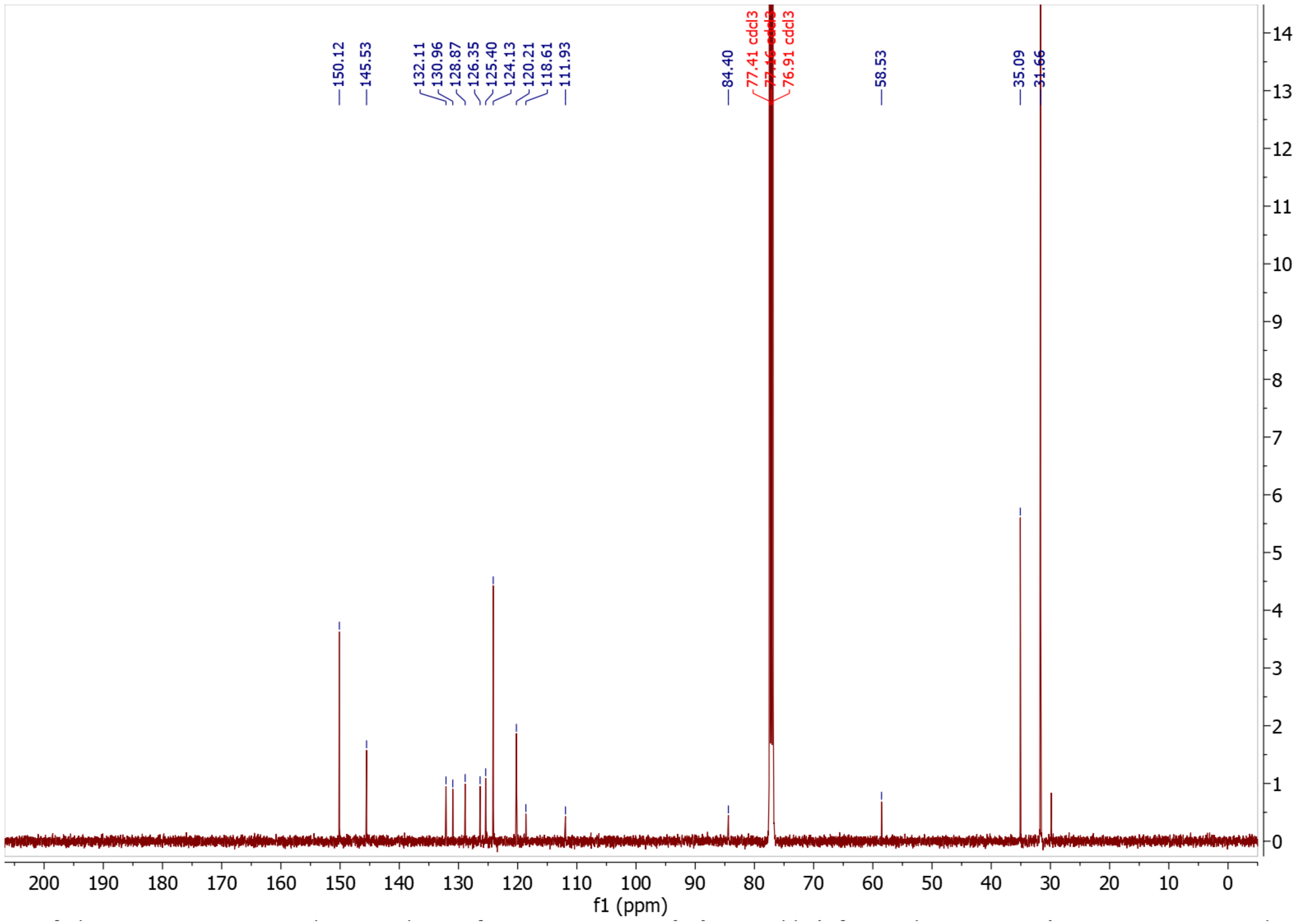

Figure S50. ^{13}C NMR spectrum of compound **2e**, 175 MHz, $CDCl_3$.

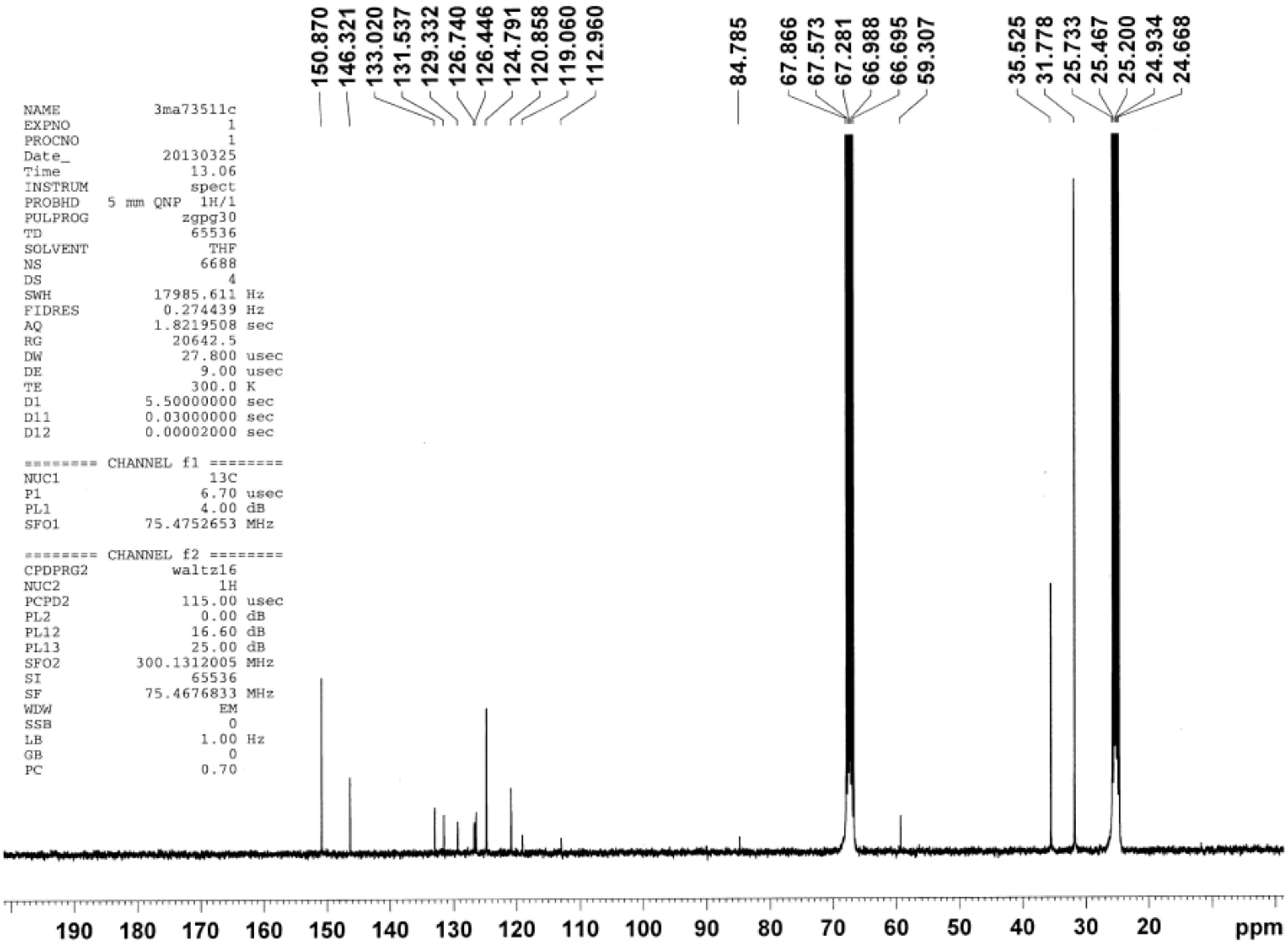

Figure S51. ^{13}C NMR spectrum of compound **2e**, 75 MHz, THF-d_8.

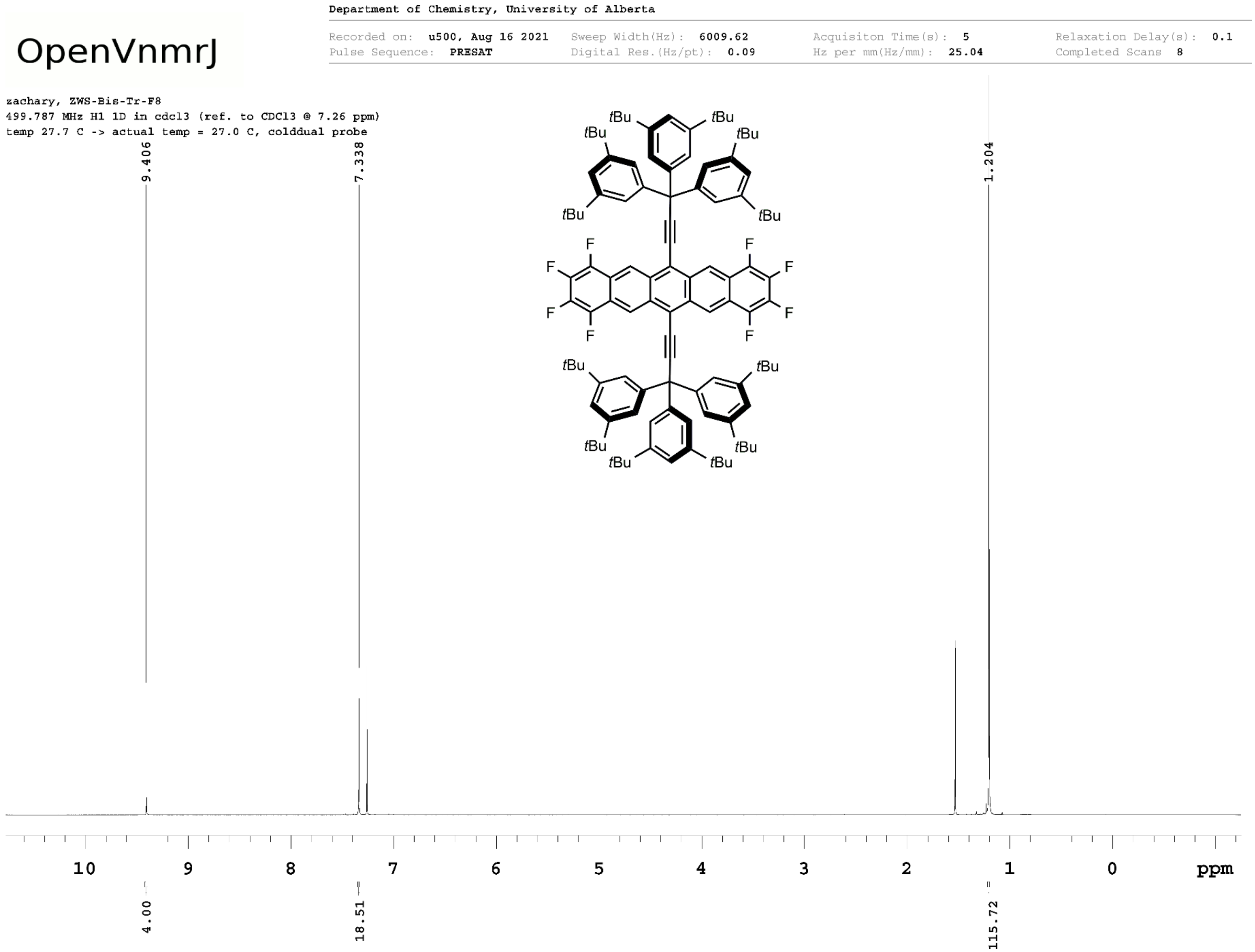

Figure S52. ^{1}H NMR spectrum of compound **2e(F$_8$)**, 500 MHz, $CDCl_3$.

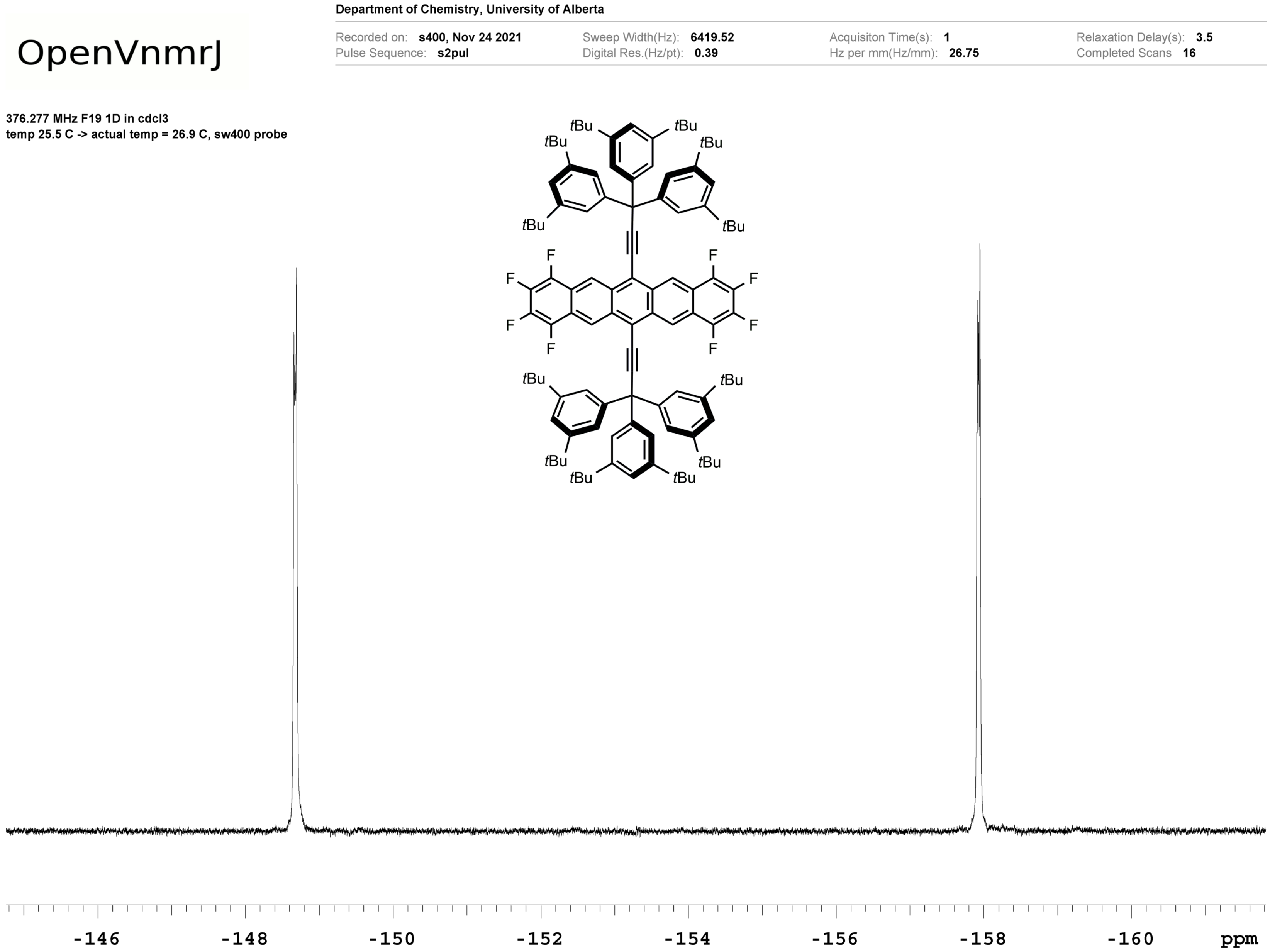

Figure S53. ^{19}F NMR spectrum of compound **2e(F$_8$)**, 376 MHz, $CDCl_3$.

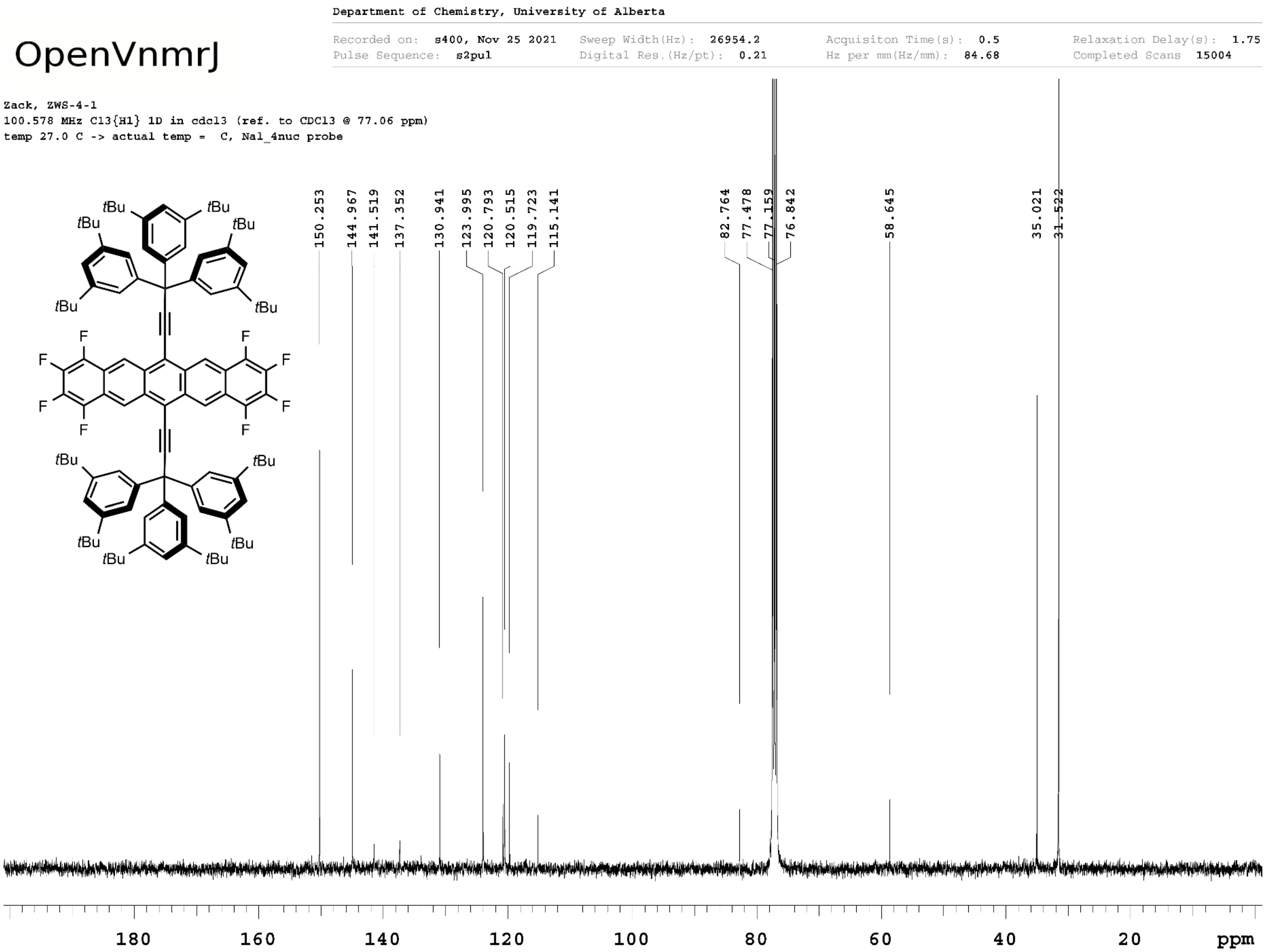

Figure S54. $^{13}C\{^{1}H, ^{19}F\}$ NMR spectrum of compound **2e(F$_8$)**, 100 MHz, $CDCl_3$.

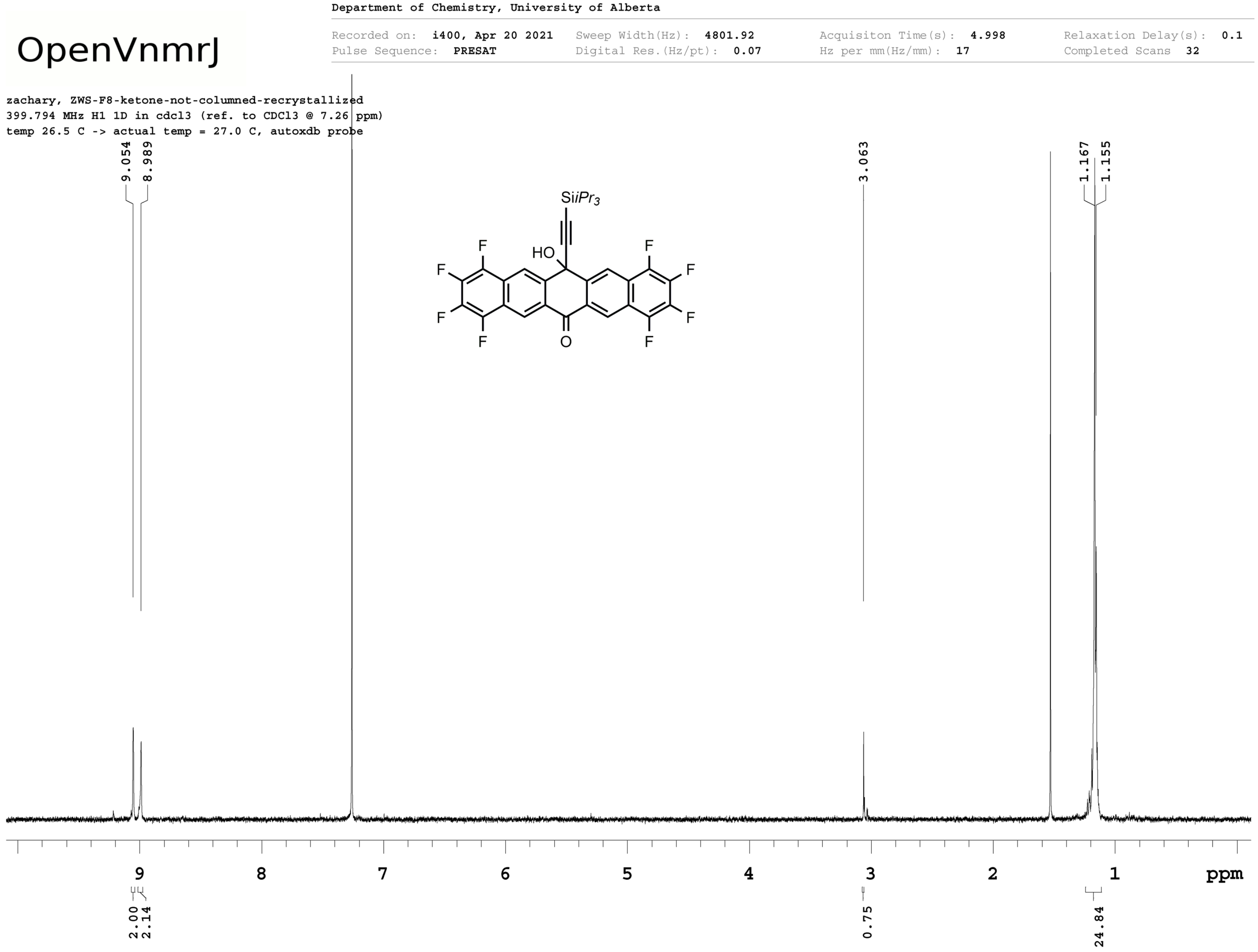

Figure S55. ^{1}H NMR spectrum of compound **3b(F$_8$)**, 400 MHz, $CDCl_3$.

OpenVnmrJ

Department of Chemistry, University of Alberta

Recorded on: i400, Apr 20 2021 Sweep Width(Hz): 78817.7 Acquisiton Time(s): 0.5 Relaxation Delay(s): 3.5
Pulse Sequence: s2pul Digital Res.(Hz/pt): 0.6 Hz per mm(Hz/mm): 328.4 Completed Scans 32

zachary, ZWS-F8-ketone-not-columned-recrystallized
376.134 MHz F19 1D in cdcl3
temp 26.5 C -> actual temp = 27.0 C, autoxdb probe

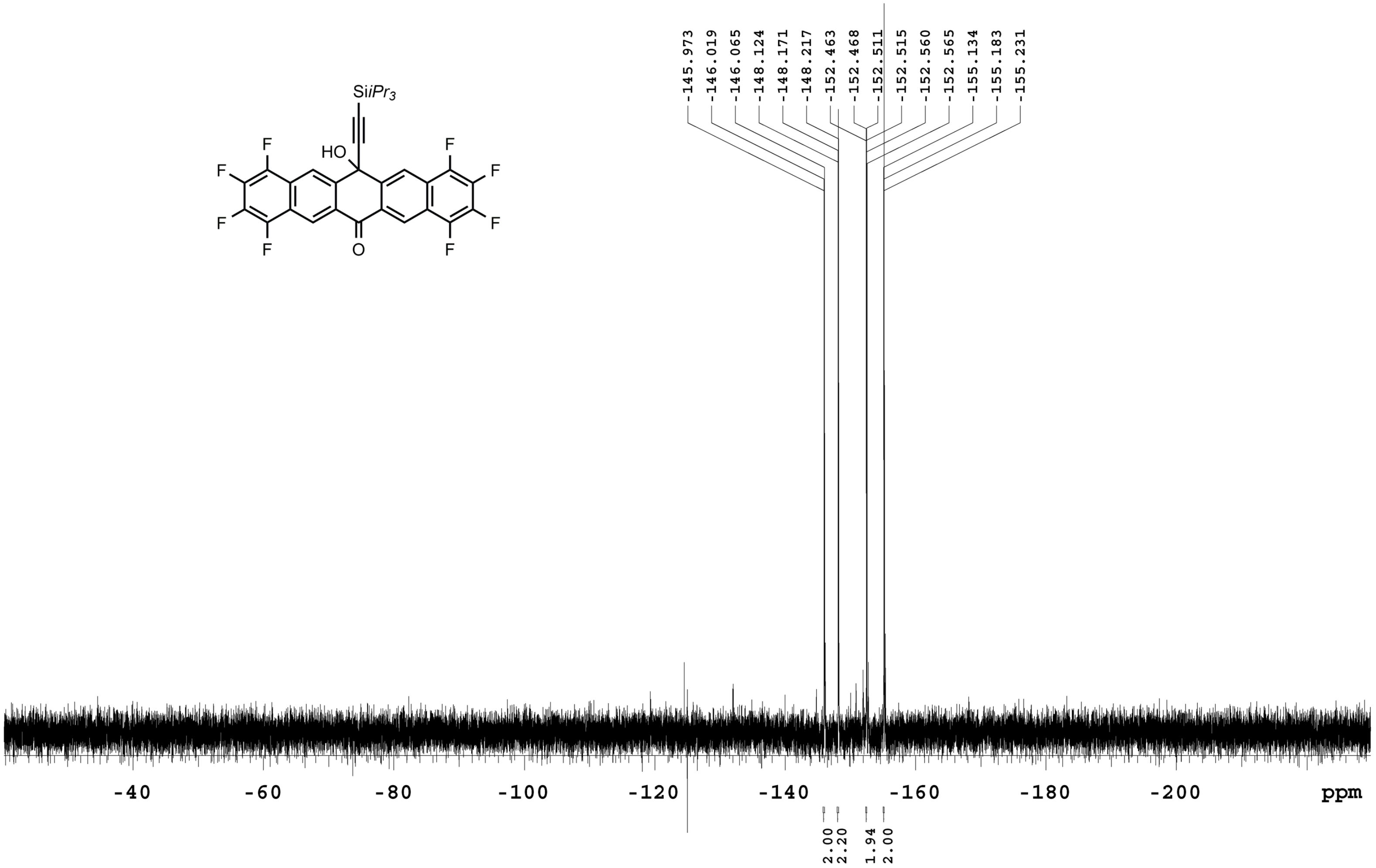

Figure S56. ^{19}F NMR spectrum of compound **3b(F$_8$)**, 376 MHz, $CDCl_3$.

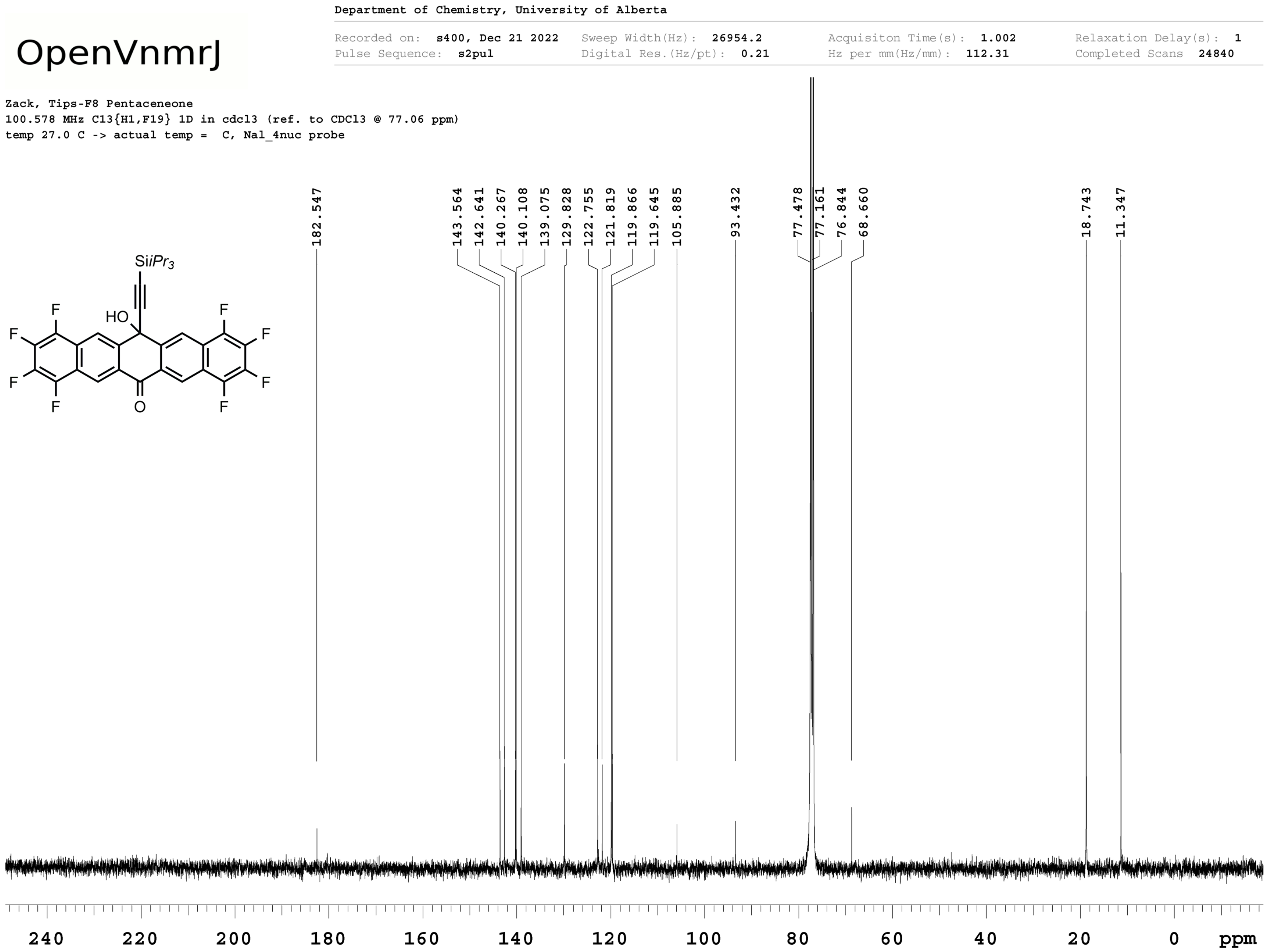

Figure S57. $^{13}C\{^1H, ^{19}F\}$ NMR spectrum of compound **3b(F$_8$)**, 100 MHz, $CDCl_3$.

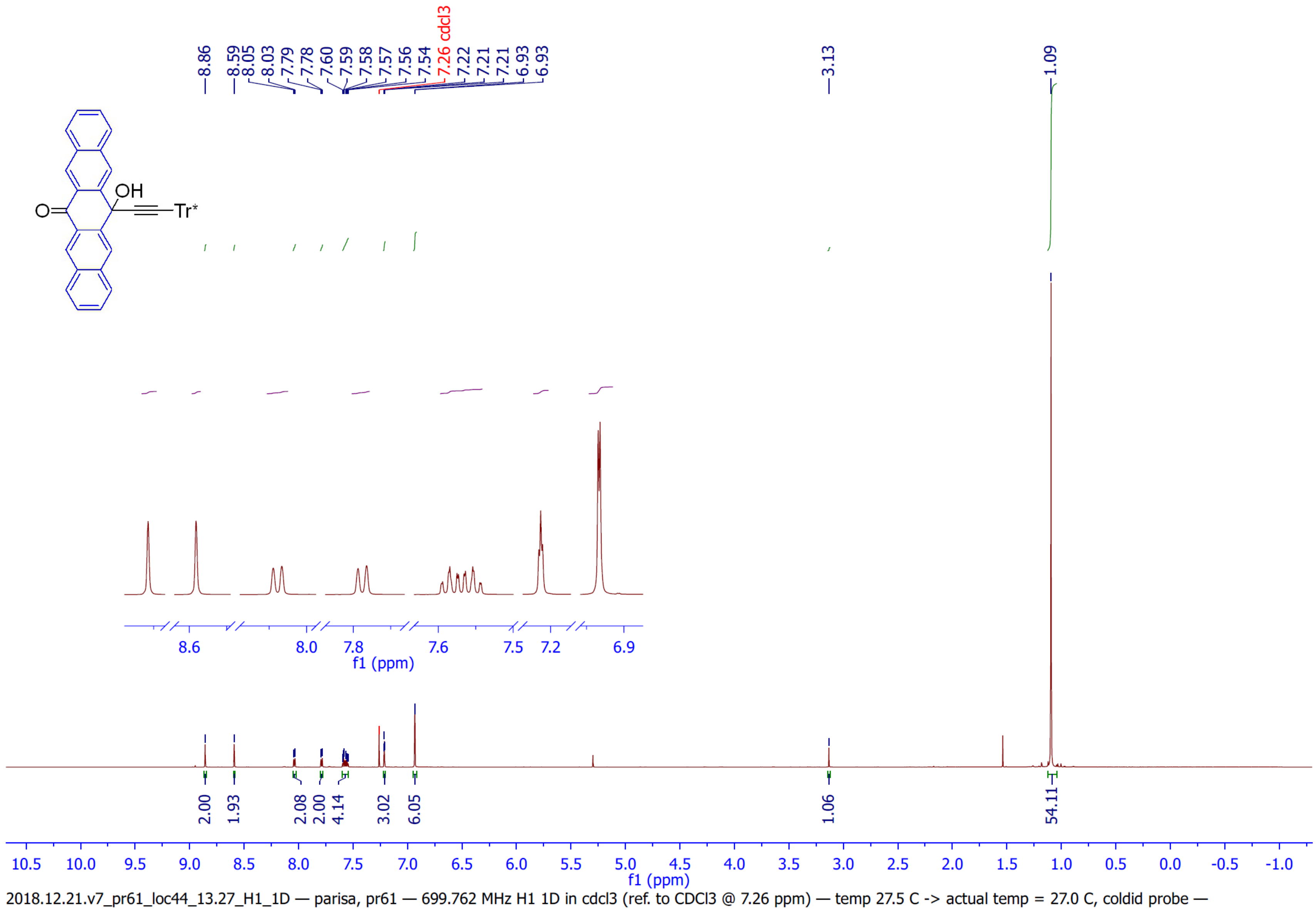

Figure S58. ^{1}H NMR spectrum of compound **3c**, 700 MHz, $CDCl_3$.

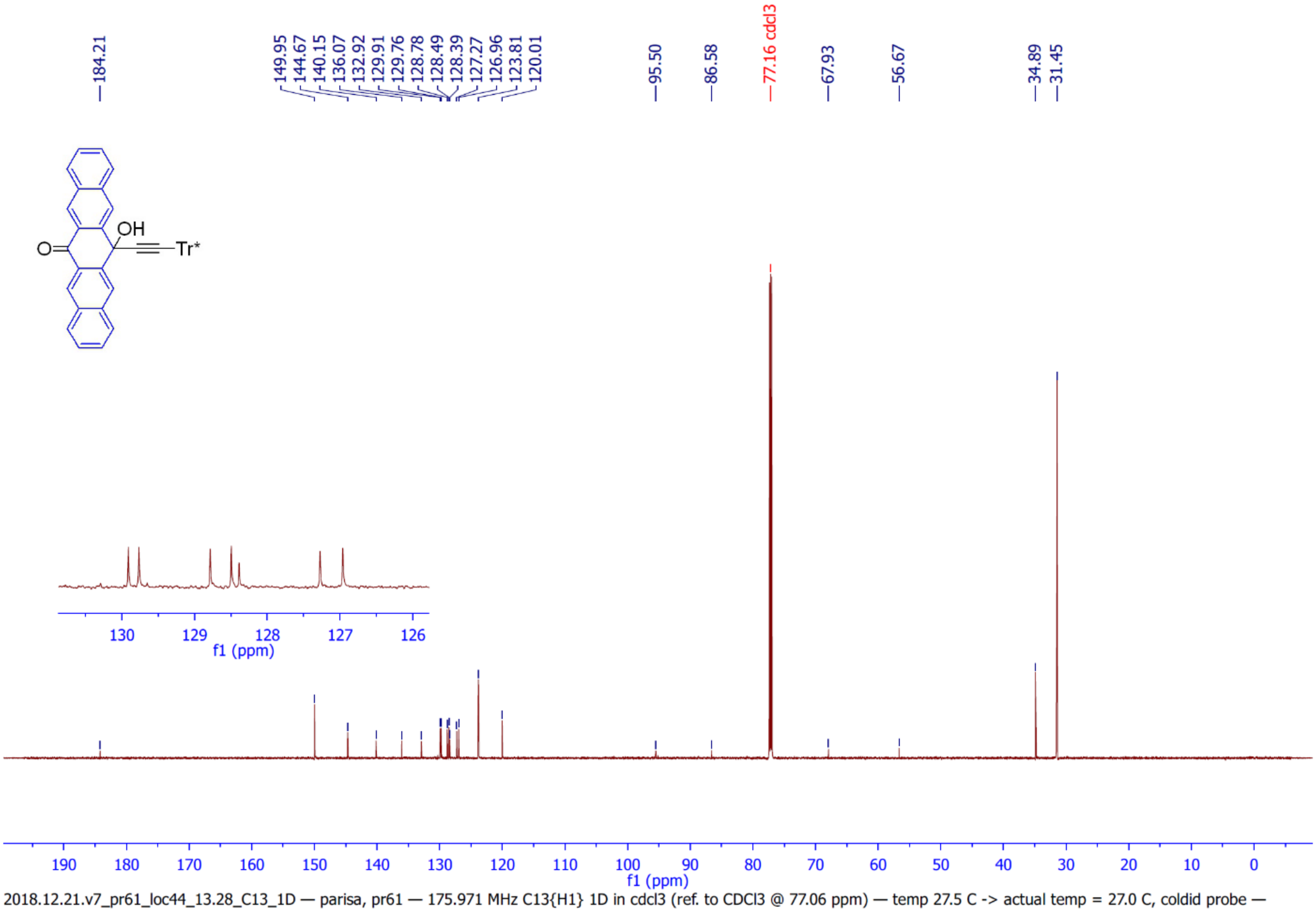

Figure S59. ^{13}C NMR spectrum of compound **3c**, 176 MHz, $CDCl_3$.

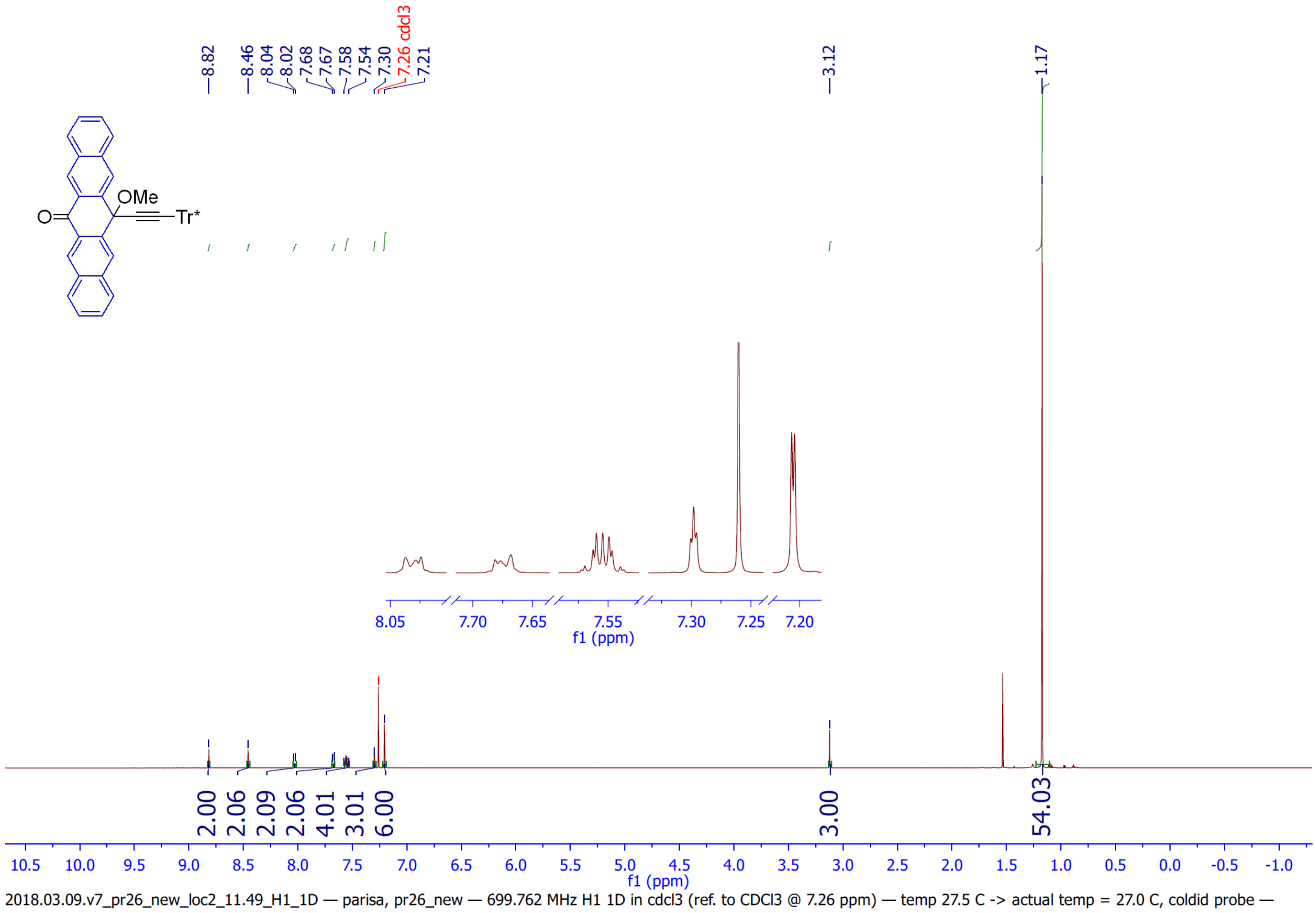

Figure S60. ^{1}H NMR spectrum of compound **3d**, 700 MHz, $CDCl_3$.

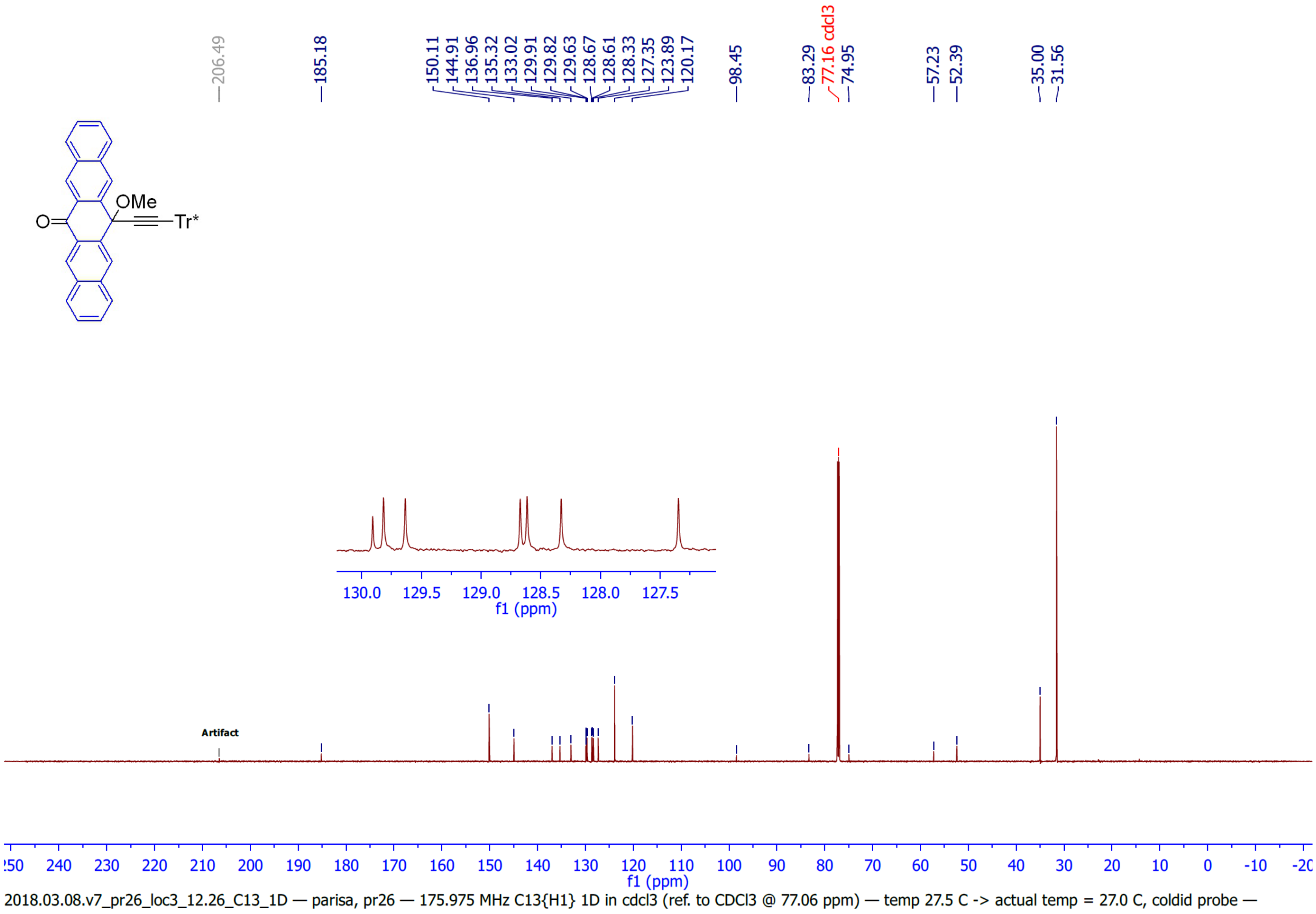

Figure S61. ^{13}C NMR spectrum of compound **3d**, 176 MHz, $CDCl_3$.

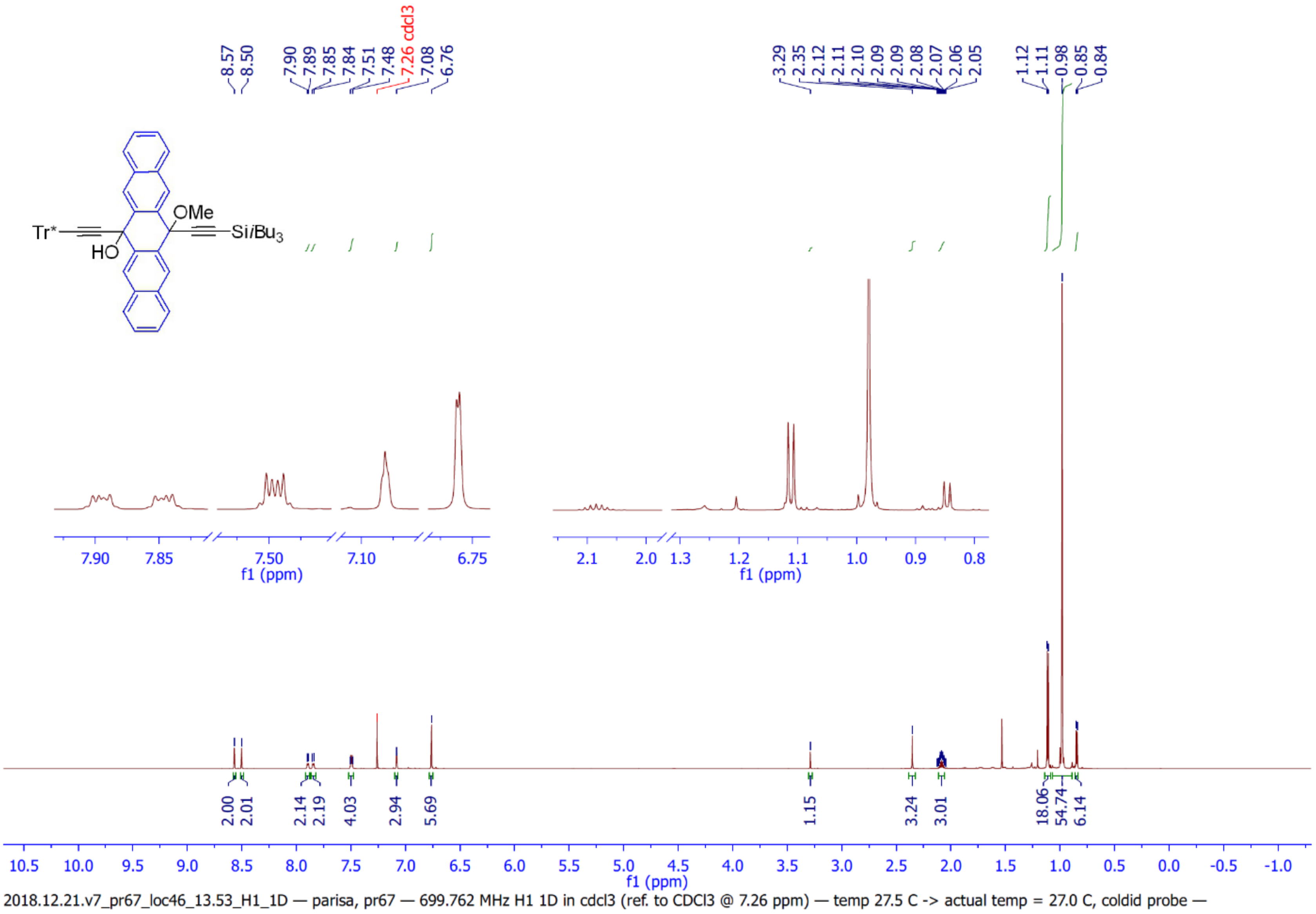

Figure S62. ^{1}H NMR spectrum of compound **5a**, 700 MHz, $CDCl_3$.

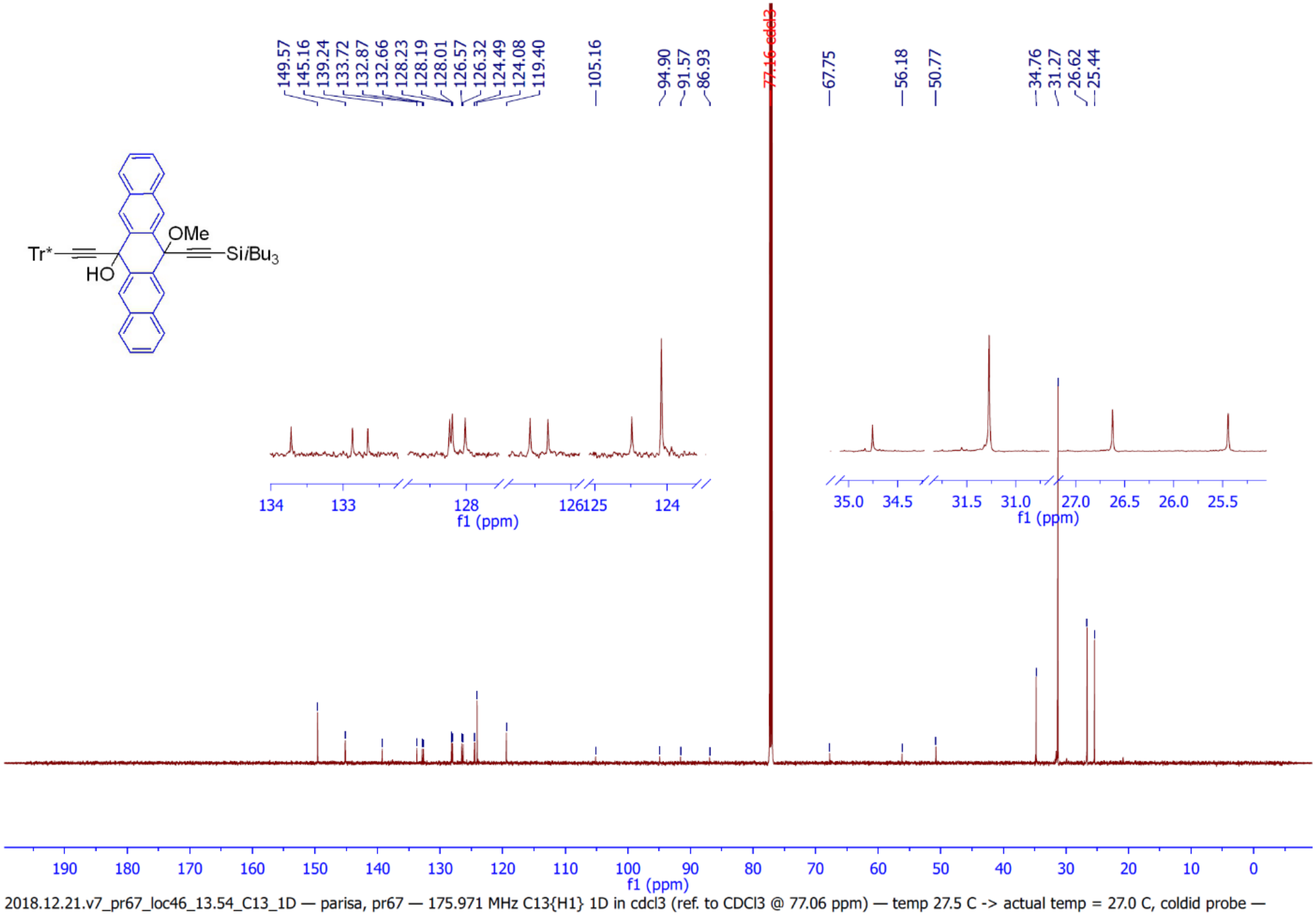

Figure S63. ^{13}C NMR spectrum of compound **5a**, 176 MHz, $CDCl_3$.

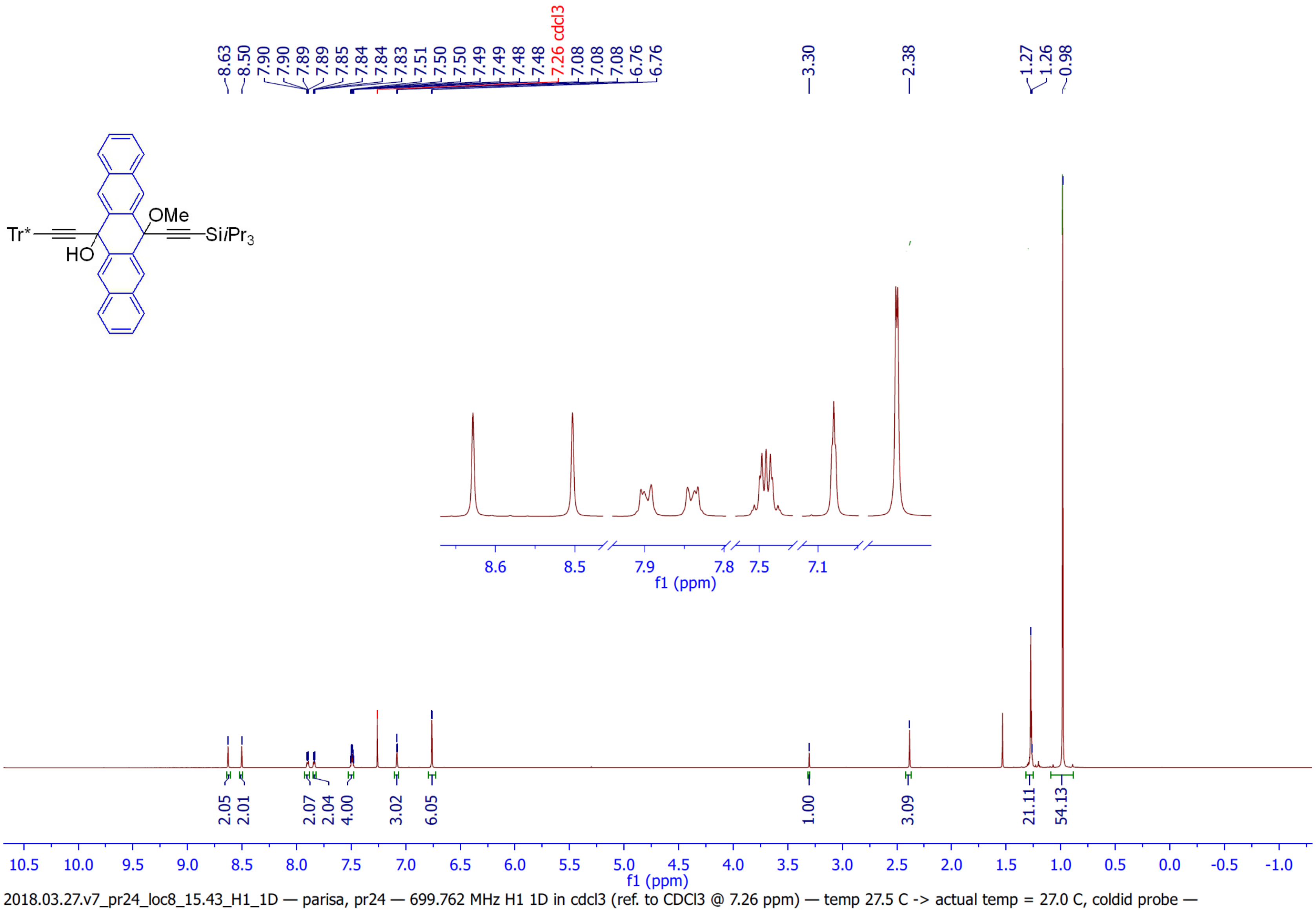

Figure S64. ^{1}H NMR spectrum of compound **5b**, 700 MHz, $CDCl_3$.

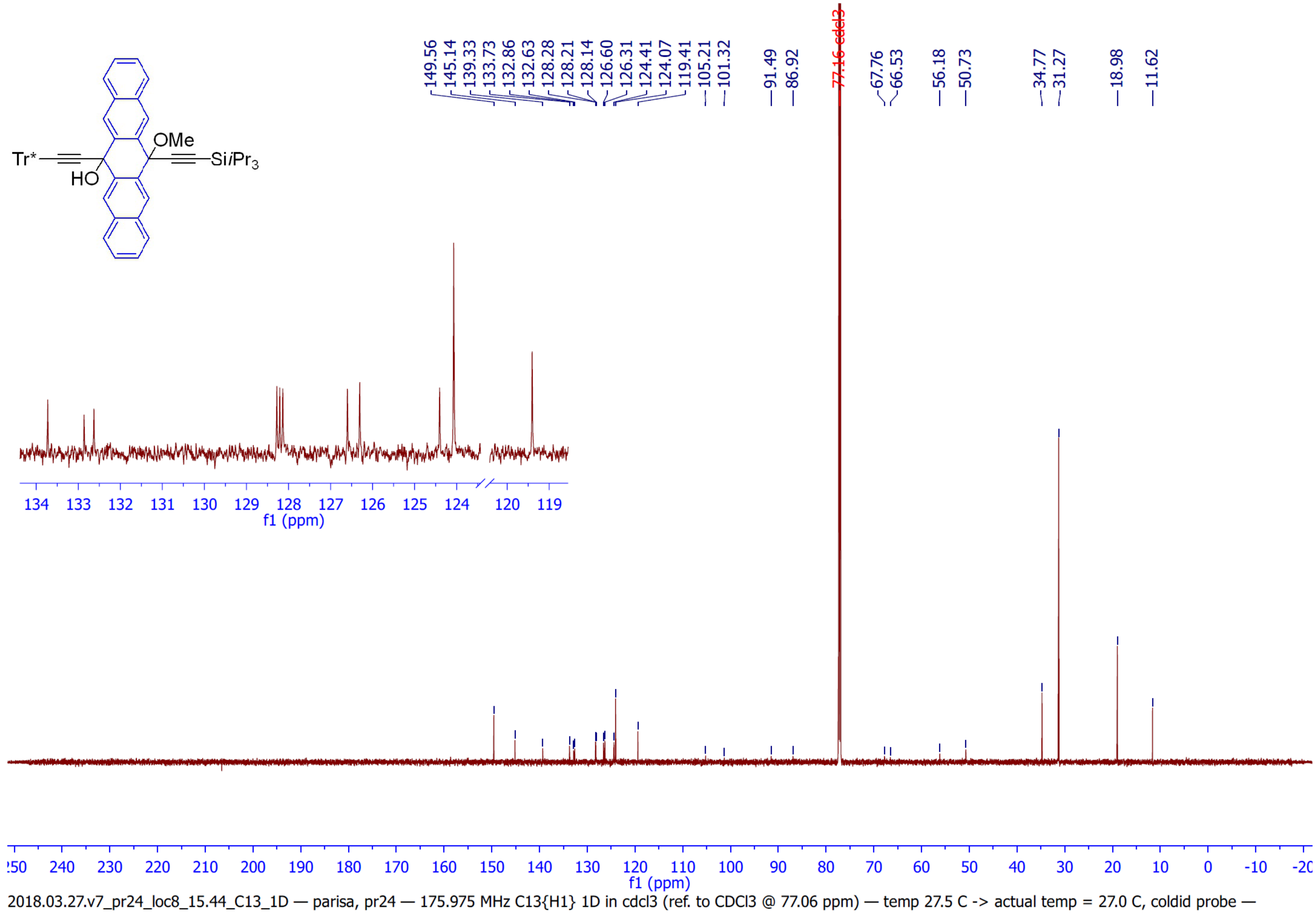

Figure S65. ^{13}C NMR spectrum of compound **5b**, 176 MHz, $CDCl_3$.

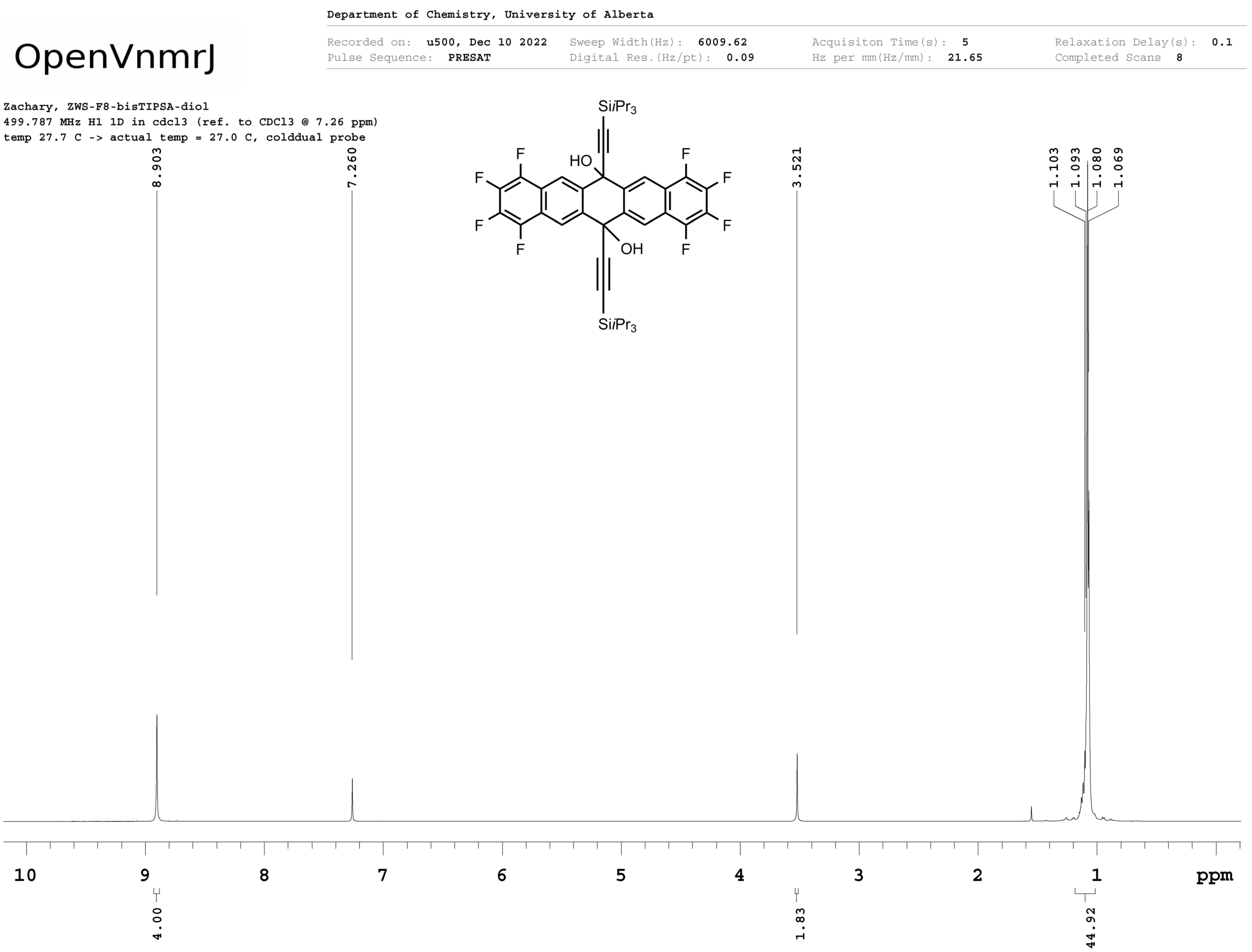

Figure S66. ^{1}H NMR spectrum of compound **5b(F_8)**, 500 MHz, $CDCl_3$.

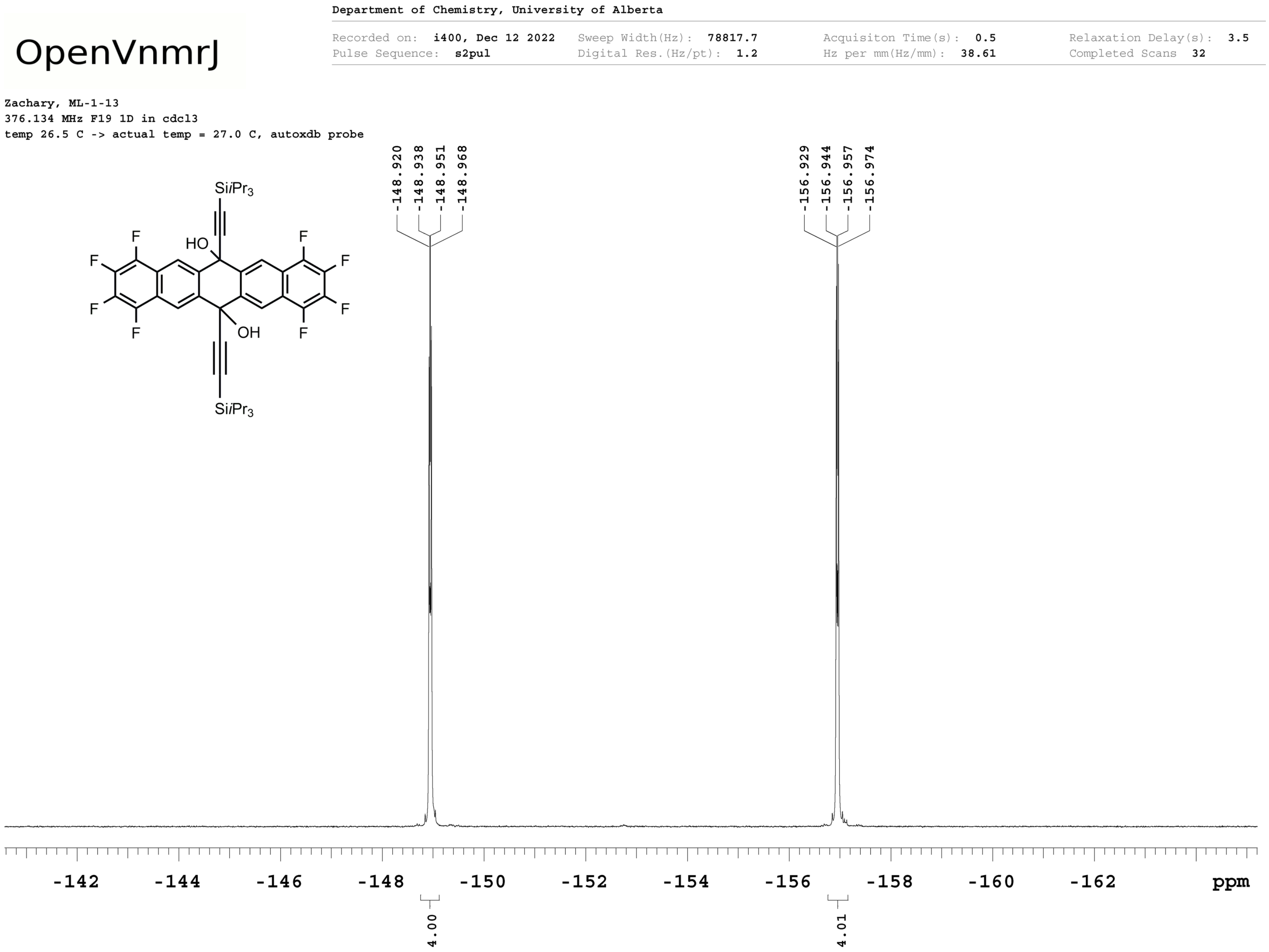

Figure S67. ^{19}F NMR spectrum of compound **5b(F$_8$)**, 376 MHz, $CDCl_3$.

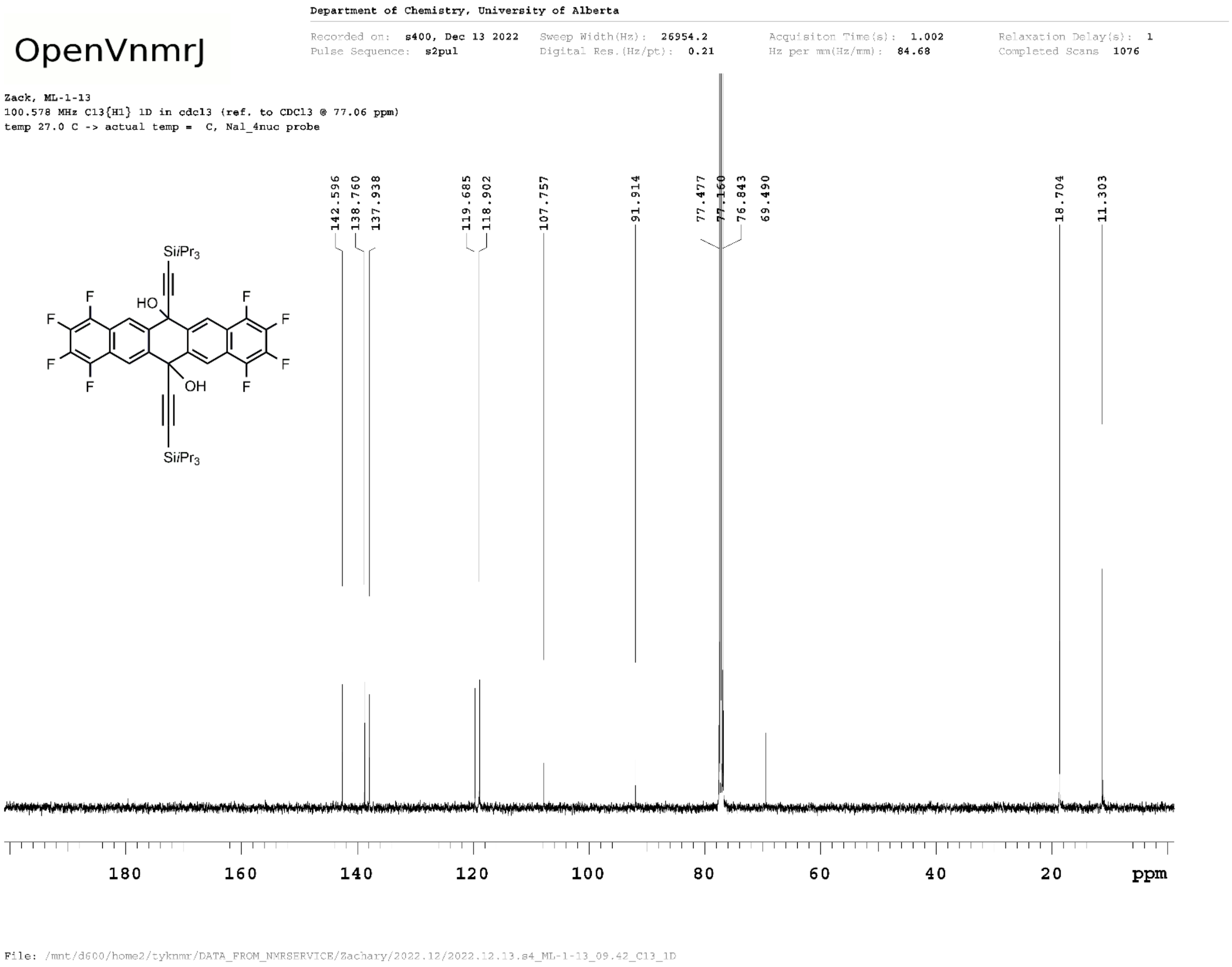

Figure S68. $^{13}C\{^{1}H, ^{19}F\}$ NMR spectrum of compound **5b(F$_8$)**, 100 MHz, $CDCl_3$.

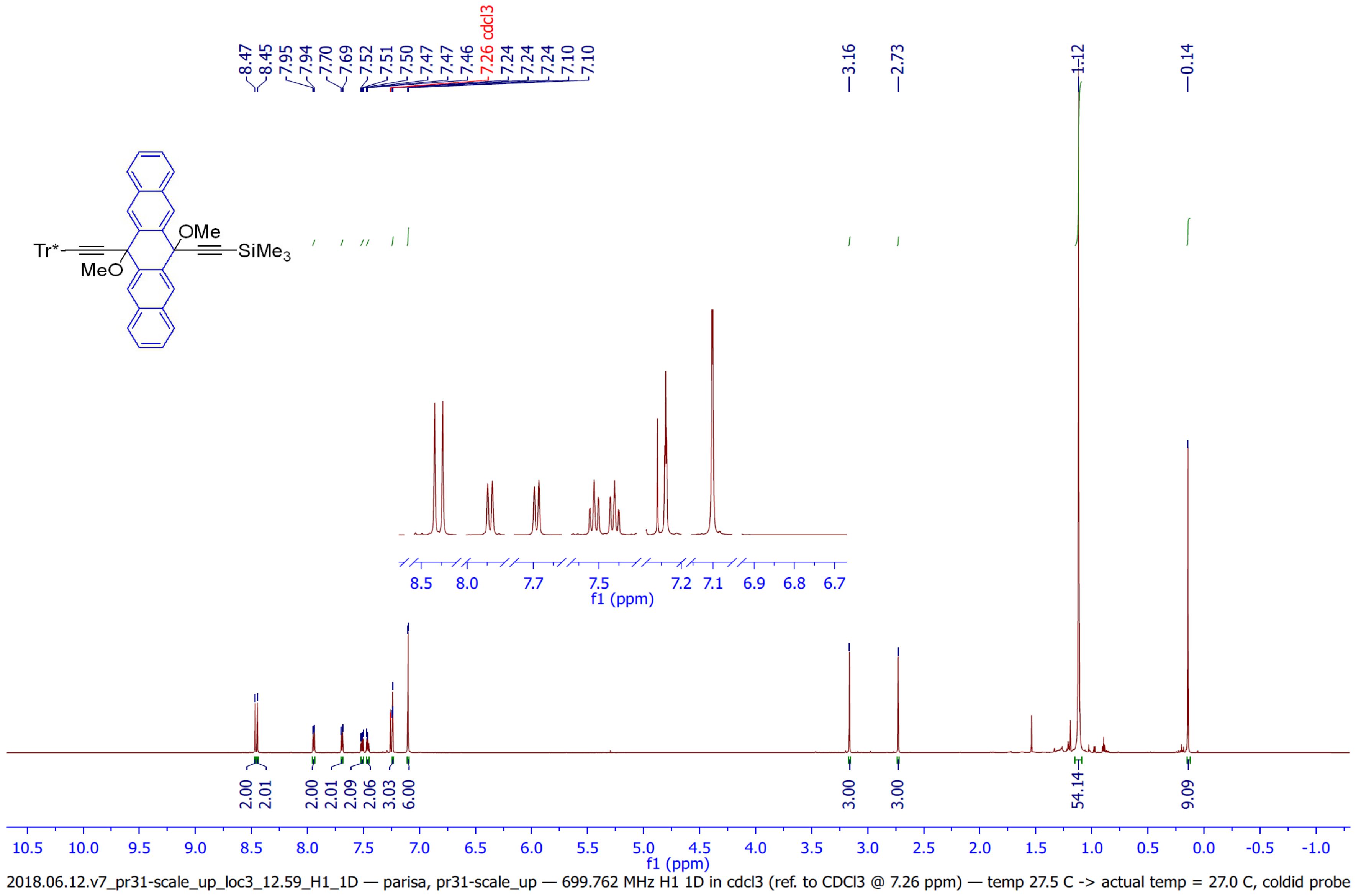

Figure S69. ^{1}H NMR spectrum of compound **5c**, 700 MHz, $CDCl_3$.

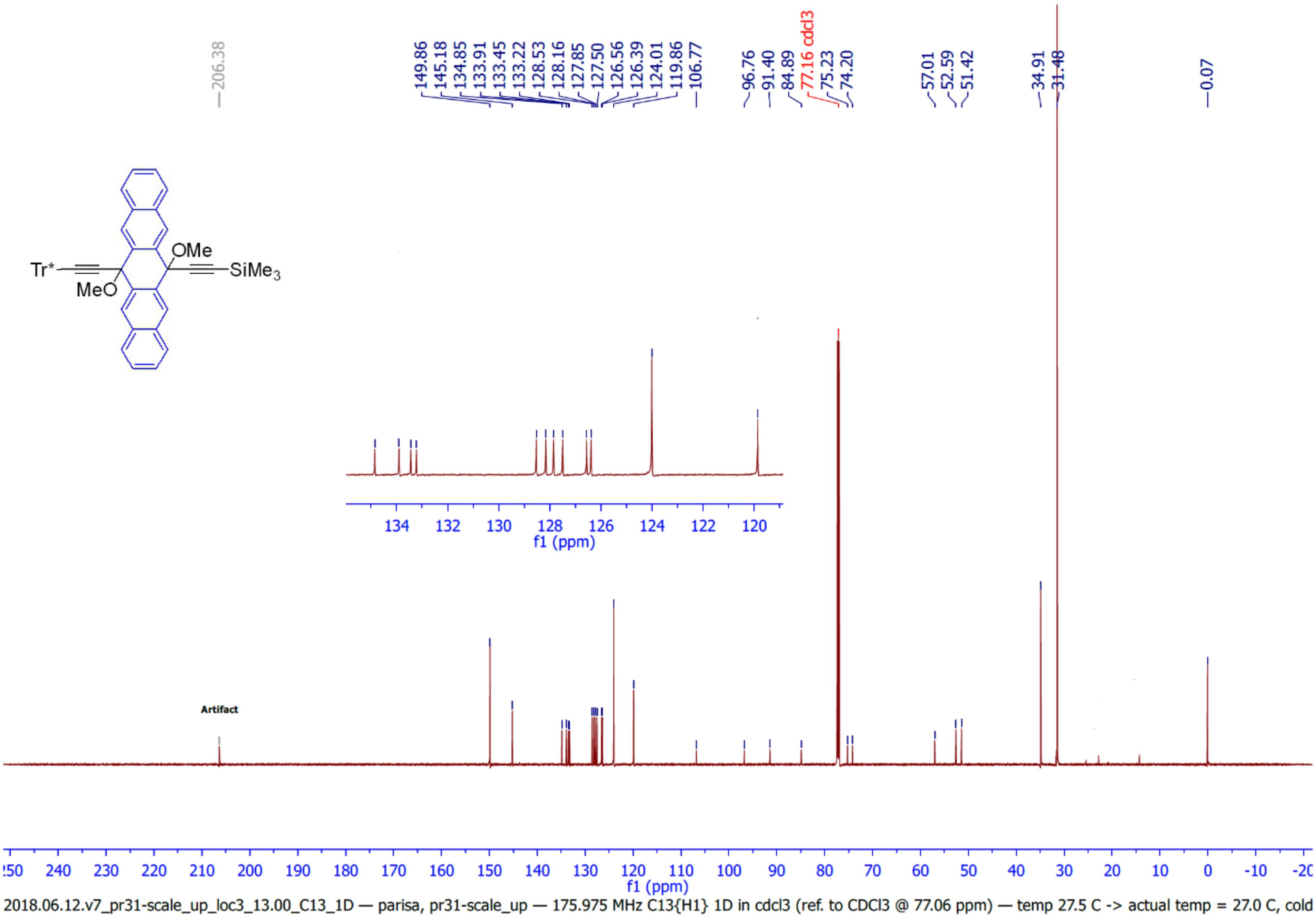

Figure S70. ^{13}C NMR spectrum of compound **5c**, 176 MHz, $CDCl_3$.

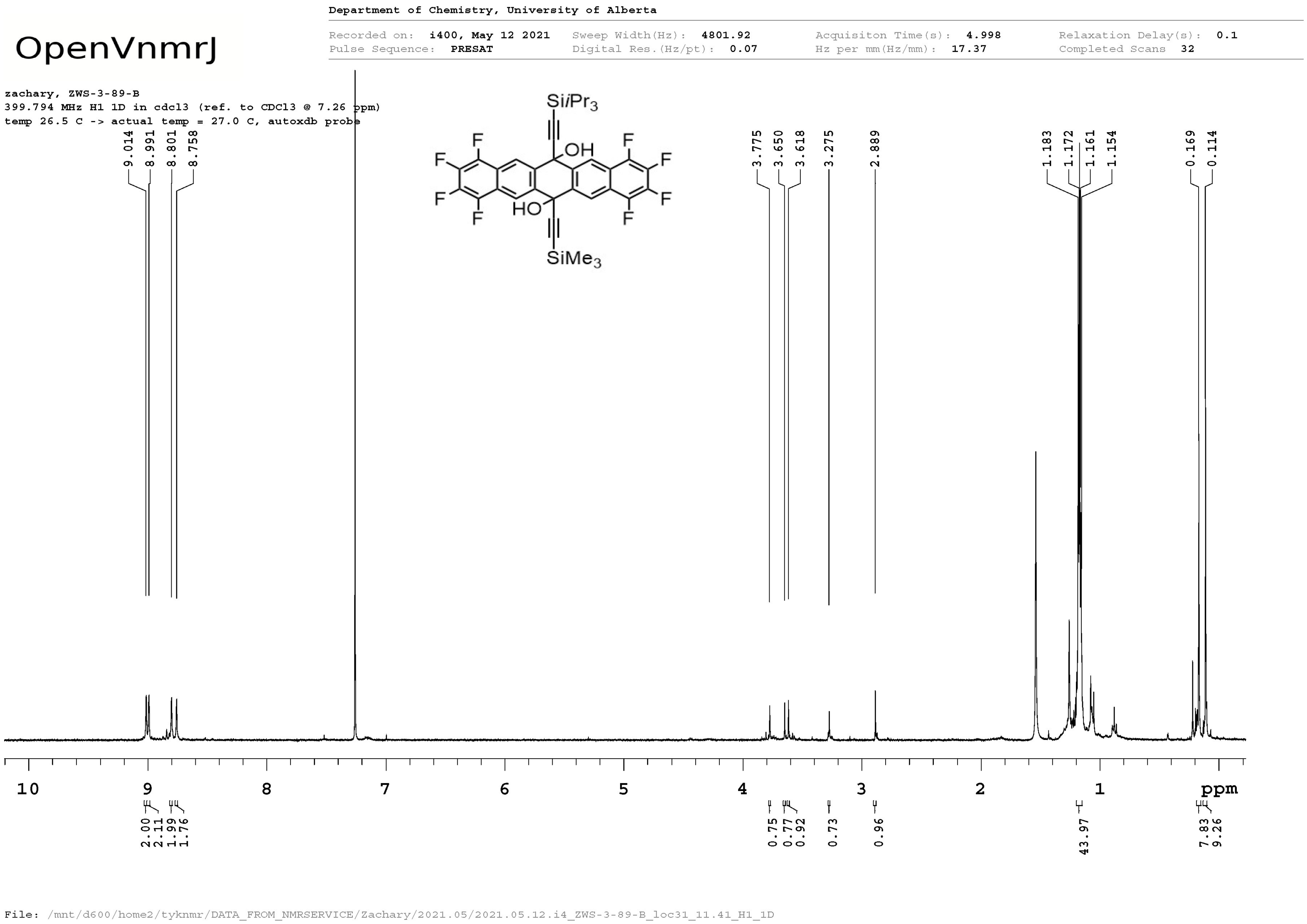

Figure S71. ^{1}H NMR spectrum of compound **5c(F$_8$)**, 400 MHz, $CDCl_3$.

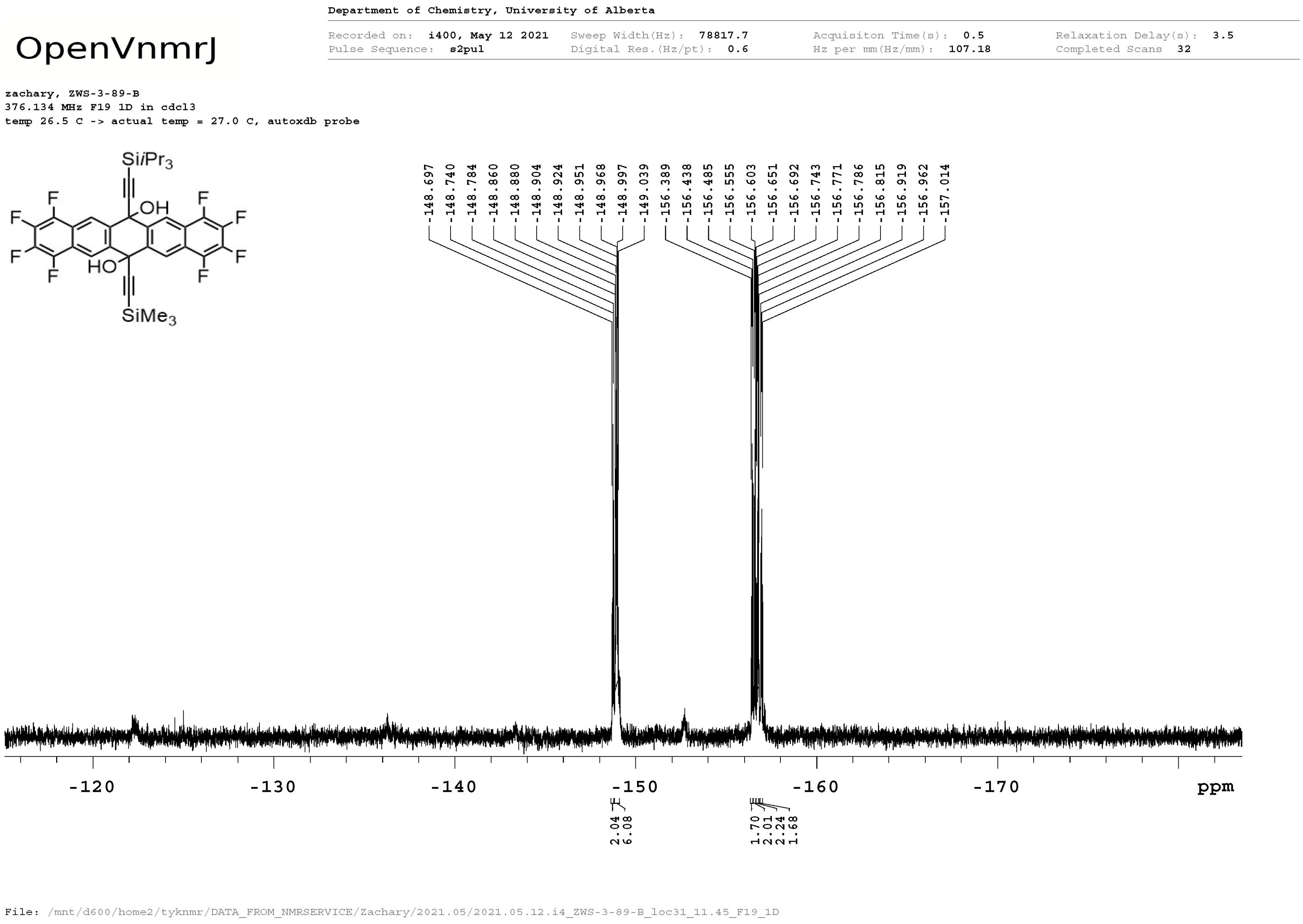

Figure S72. ^{19}F NMR spectrum of compound **5c(F$_8$)**, 376 MHz, $CDCl_3$.

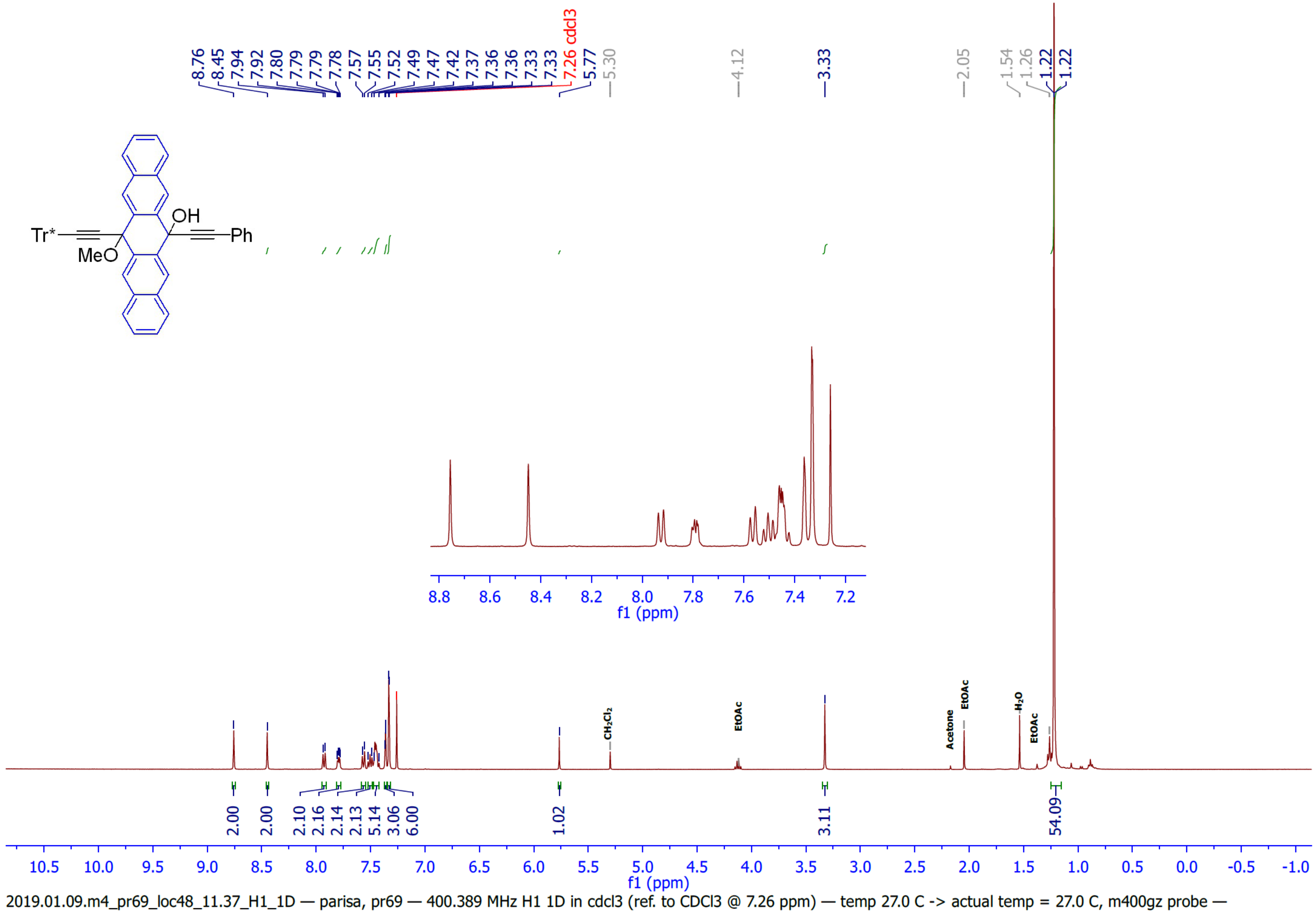

Figure S73. ^{1}H NMR spectrum of compound **5d**, 400 MHz, $CDCl_3$.

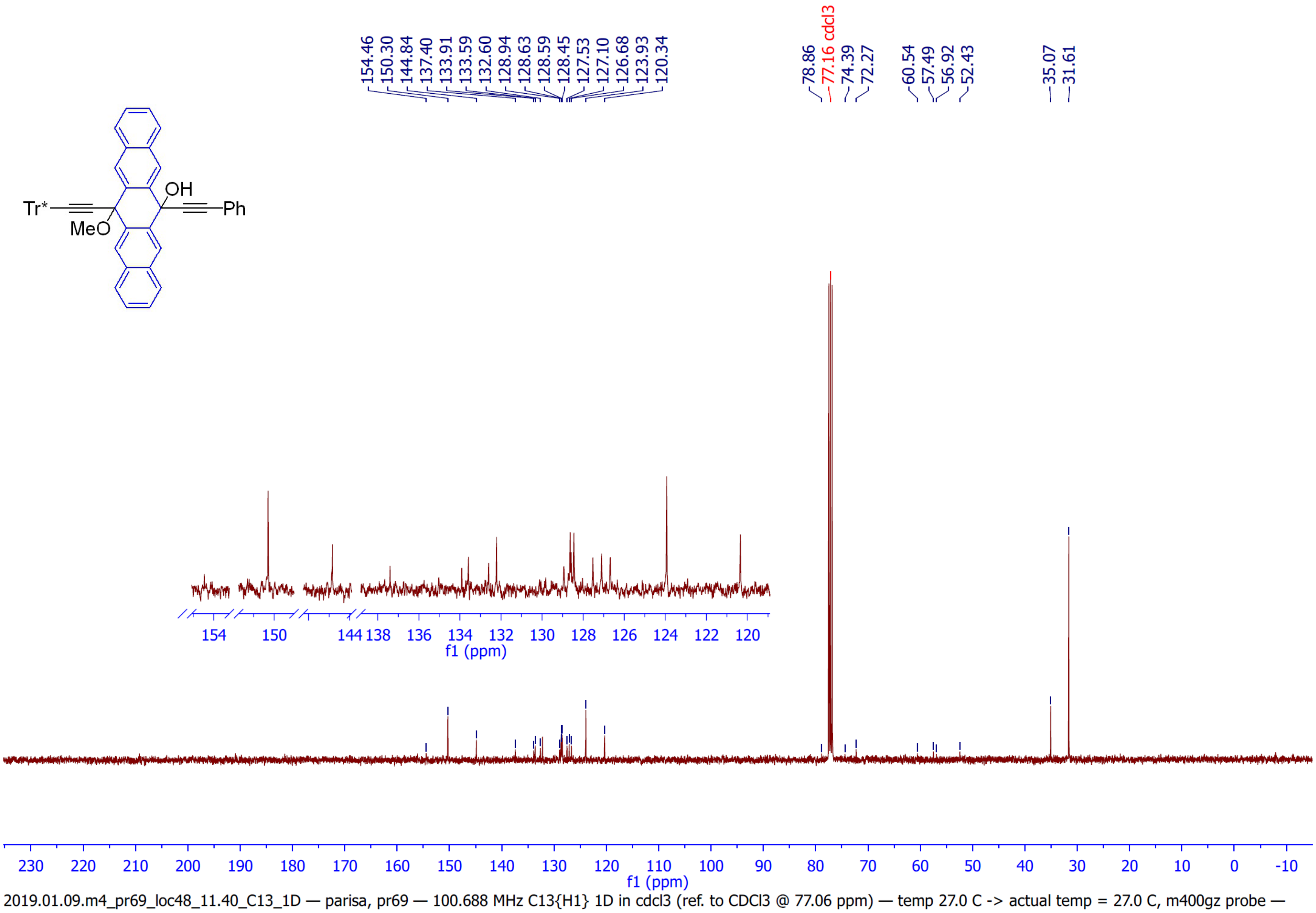

Figure S74. ^{13}C NMR spectrum of compound **5d**, 100 MHz, $CDCl_3$.

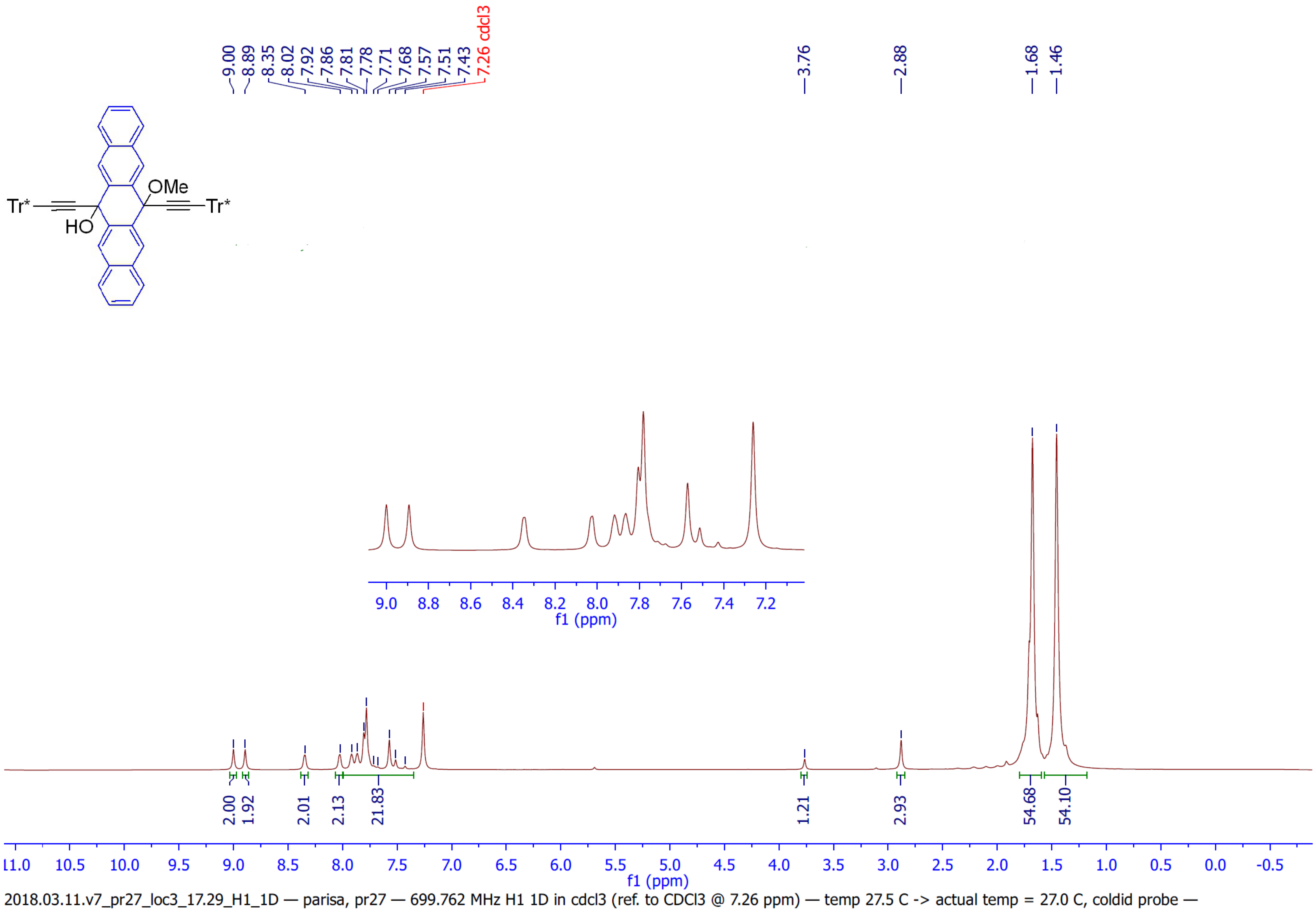

Figure S75. ^{1}H NMR spectrum of compound **5e**, 700 MHz, $CDCl_3$.

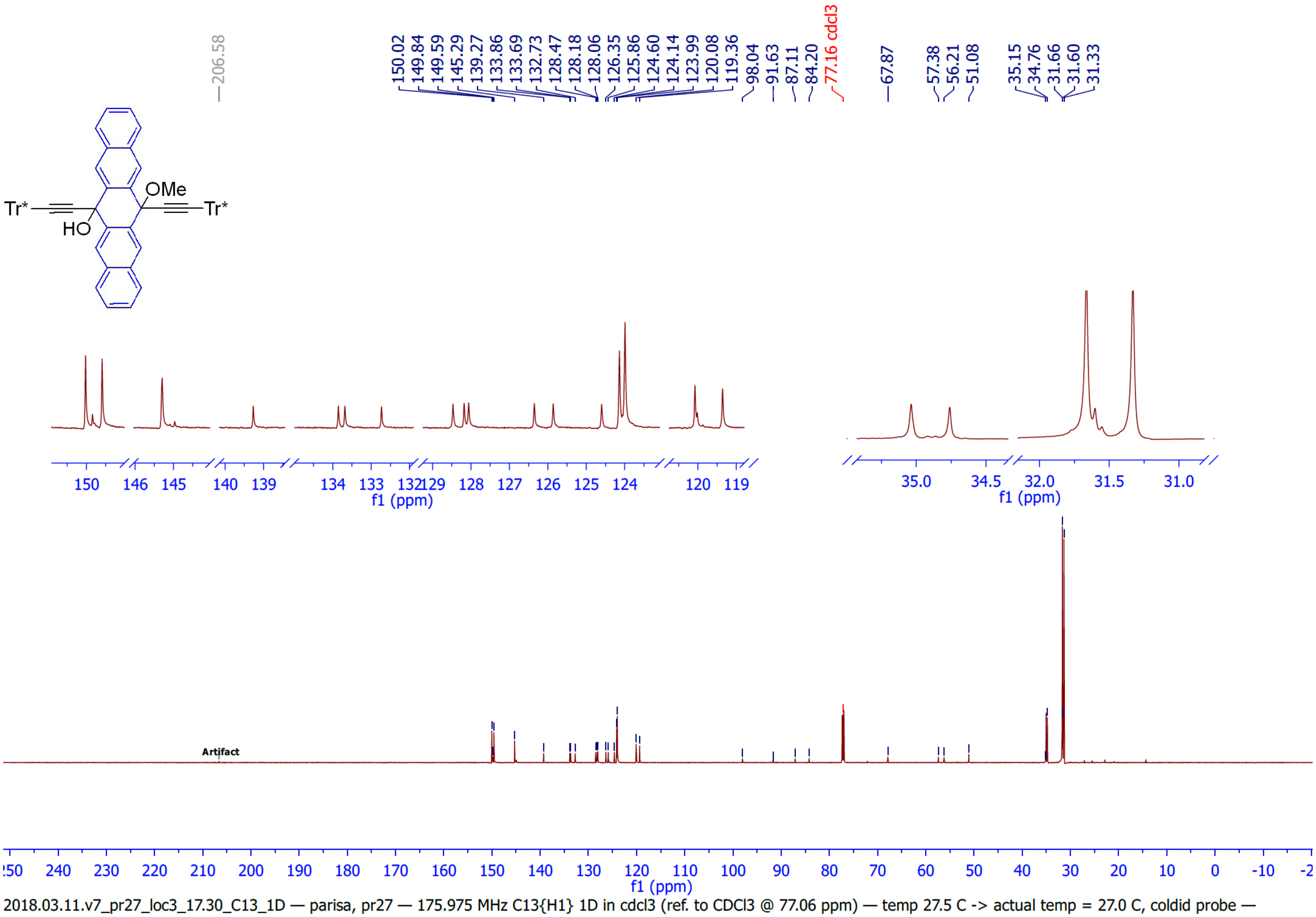

Figure S76. ^{13}C NMR spectrum of compound **5e**, 176 MHz, $CDCl_3$.

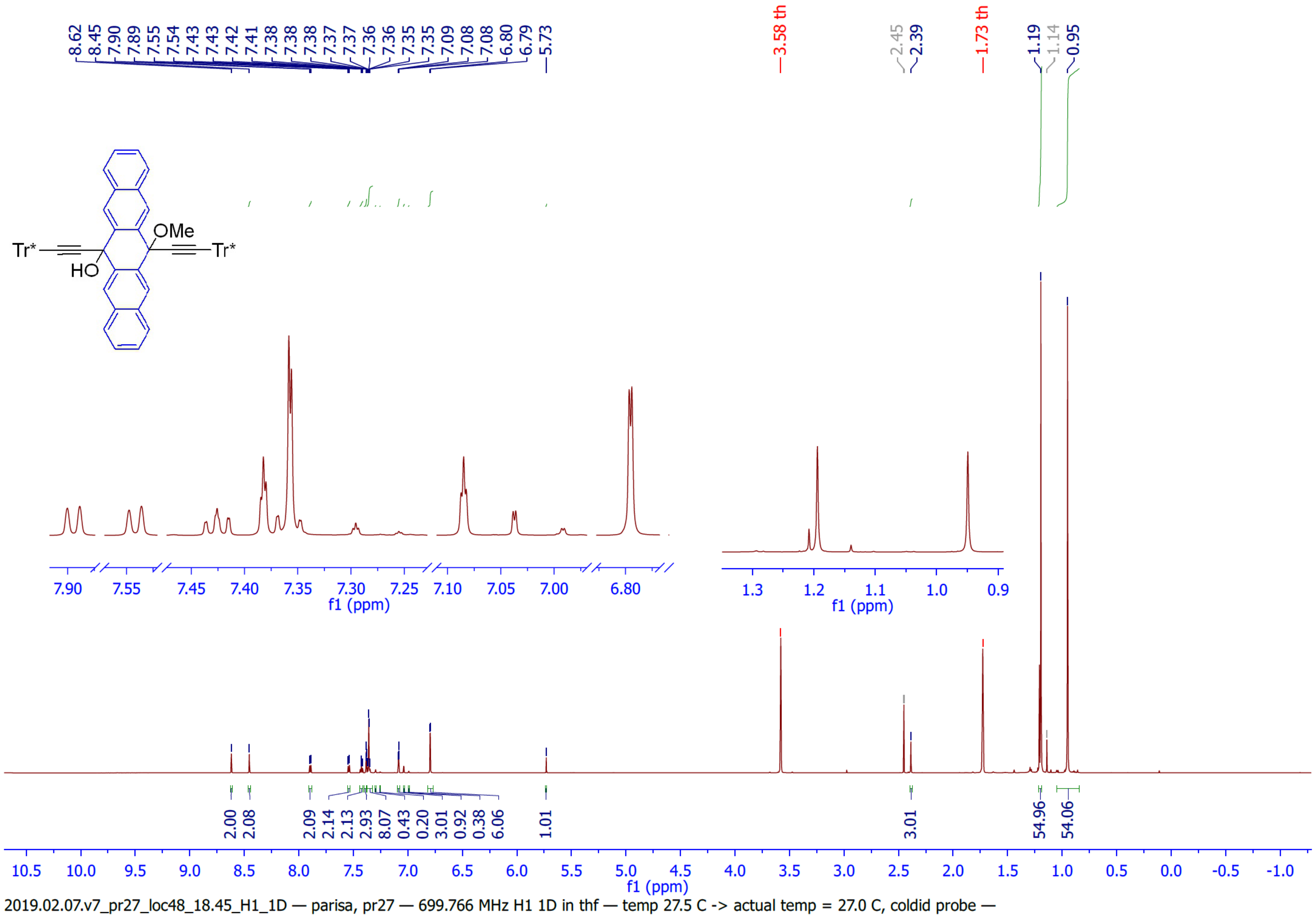

Figure S77. ^{1}H NMR spectrum of compound **5e**, 700 MHz, THF-d_8.

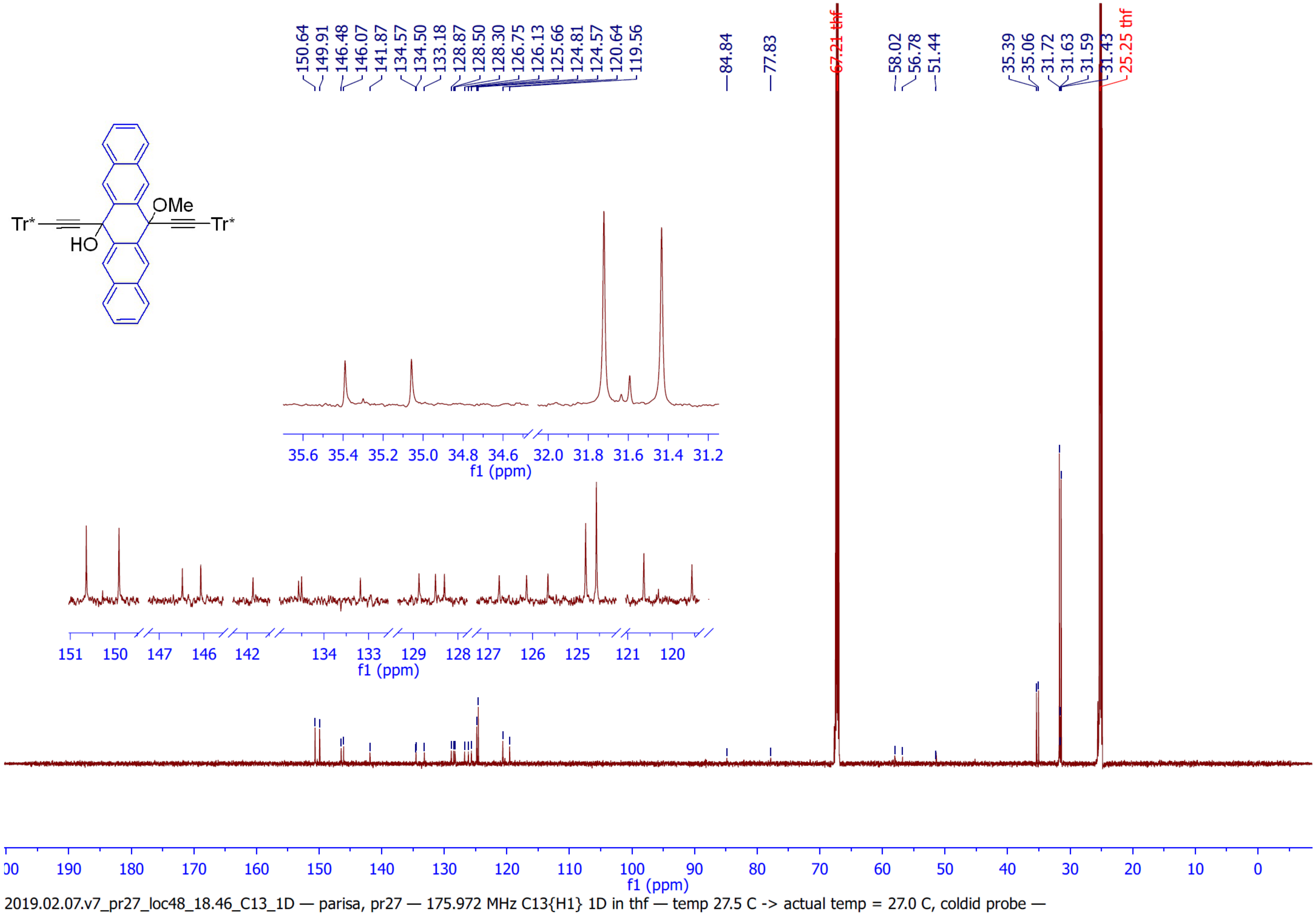

Figure S78. ^{13}C NMR spectrum of compound **5e**, 176 MHz, THF-d_8.

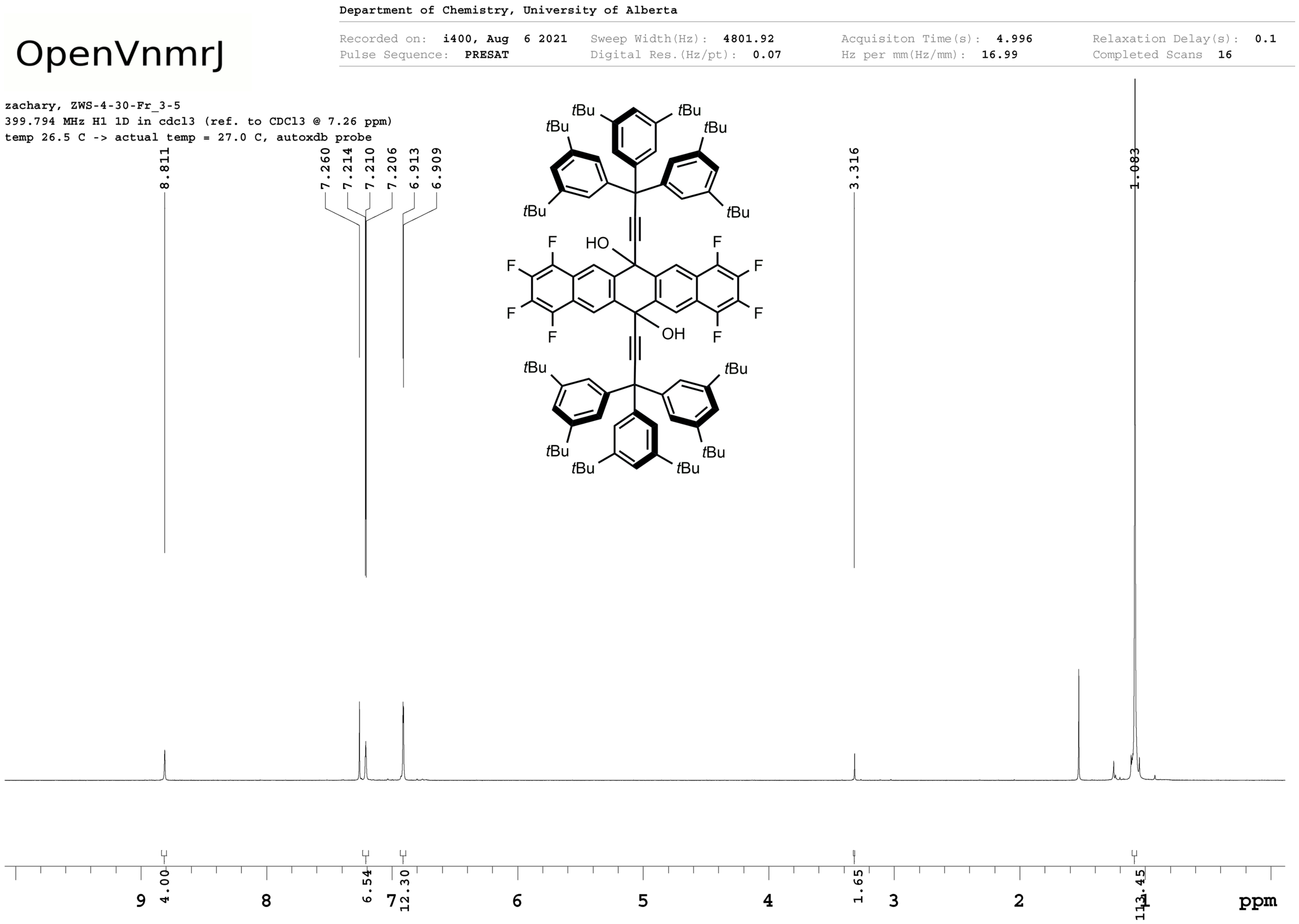

Figure S79. ^{1}H NMR spectrum of compound **5e(F$_8$)**, 400 MHz, $CDCl_3$.

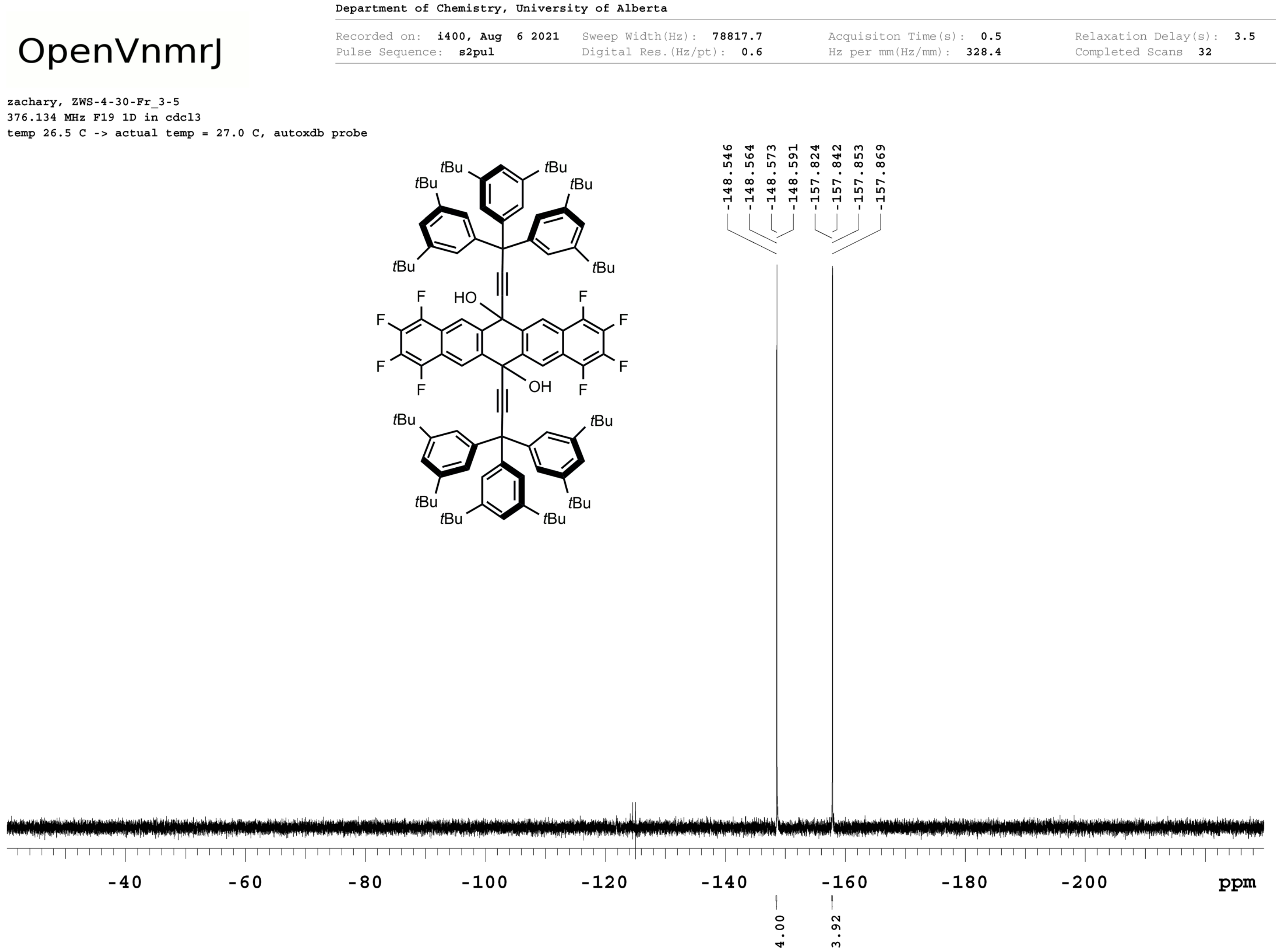

Figure S80. ^{19}F NMR spectrum of compound **5e(F**$_8$**)**, 376 MHz, $CDCl_3$.

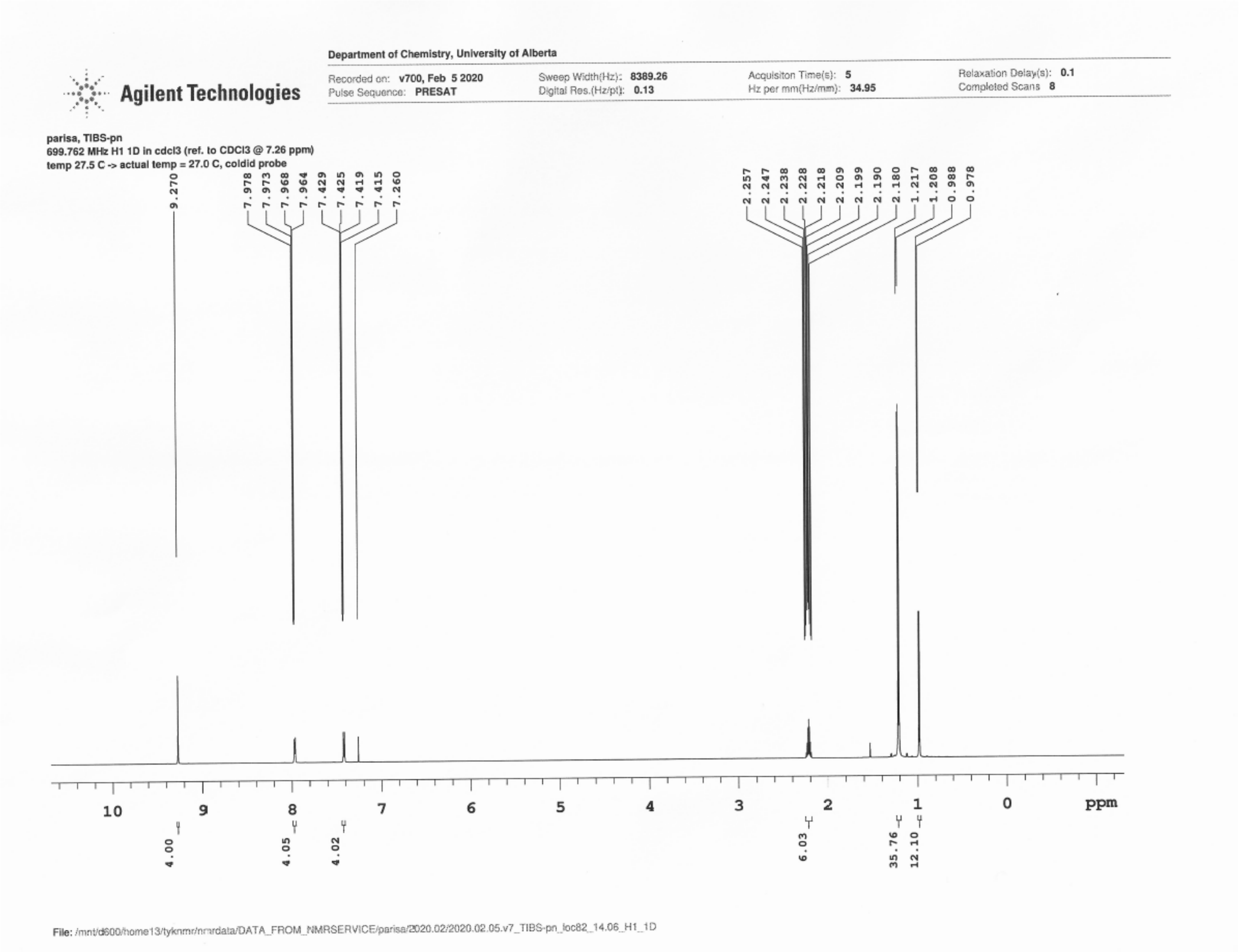

Figure S81. ^1H NMR spectrum of compound **7a**, 700 MHz, $CDCl_3$.

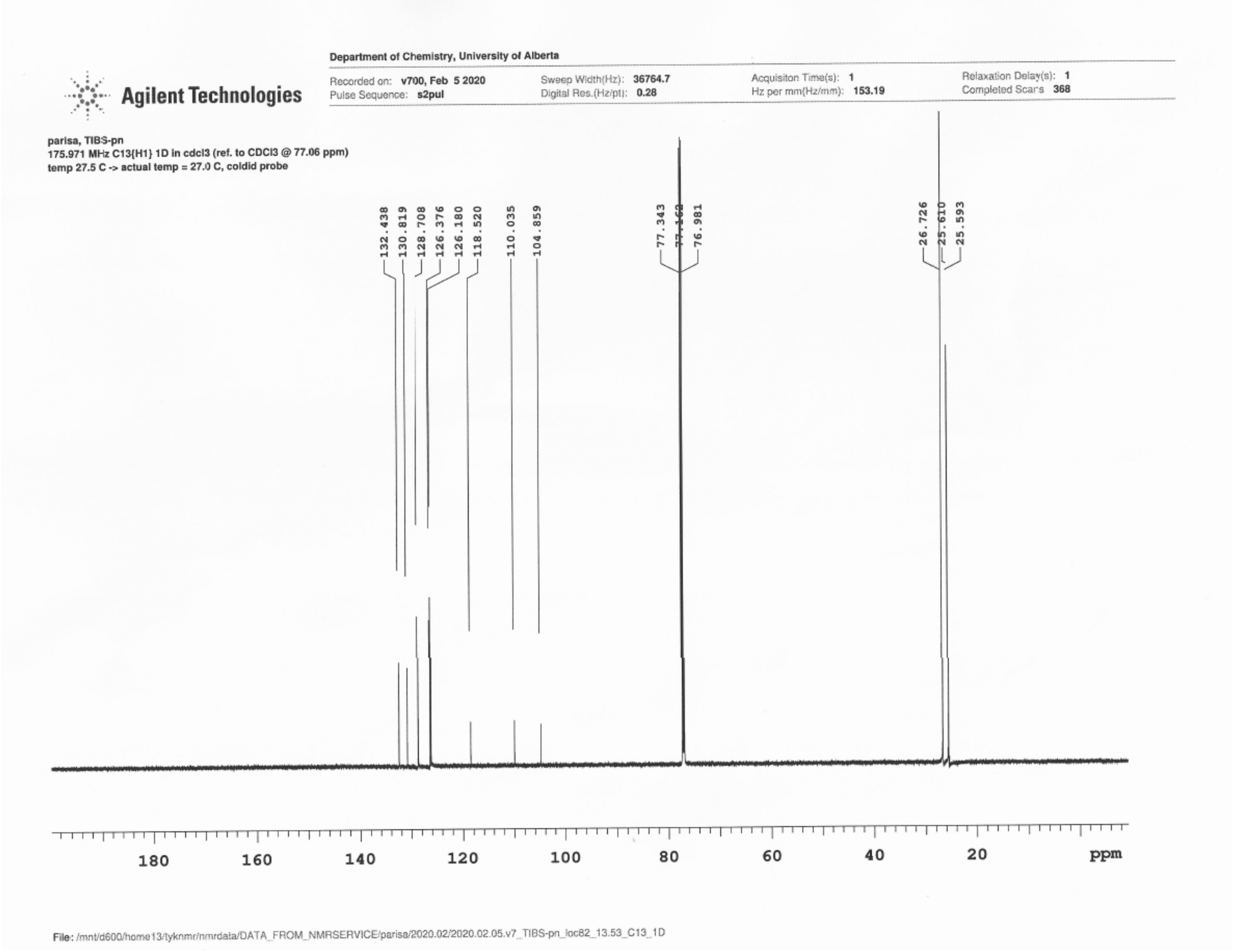

Figure S82. ^{13}C NMR spectrum of compound **7a**, 176 MHz, $CDCl_3$.

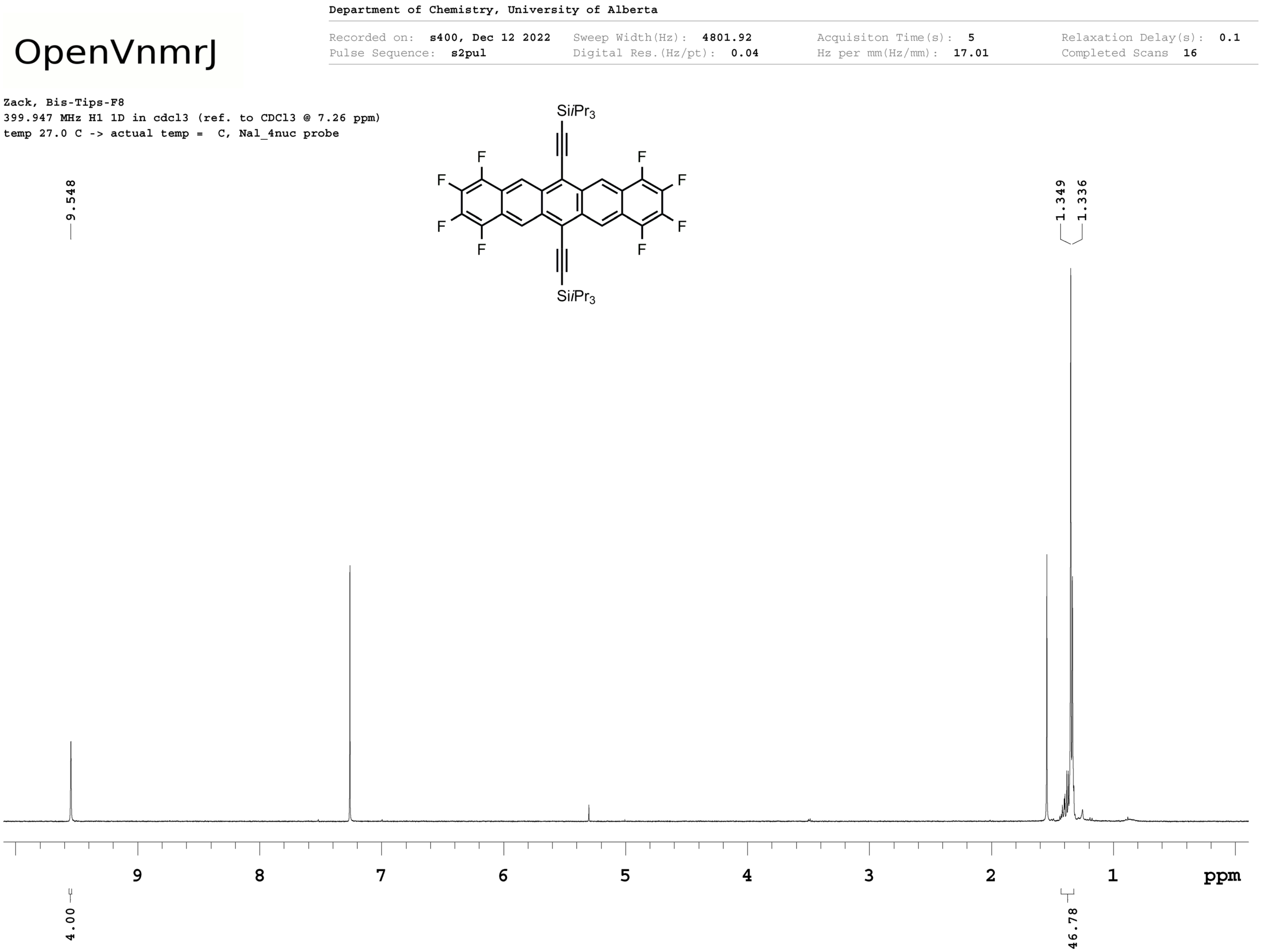

Figure S83. ^{1}H NMR spectrum of compound **7b(F$_8$)**, 400 MHz, $CDCl_3$.

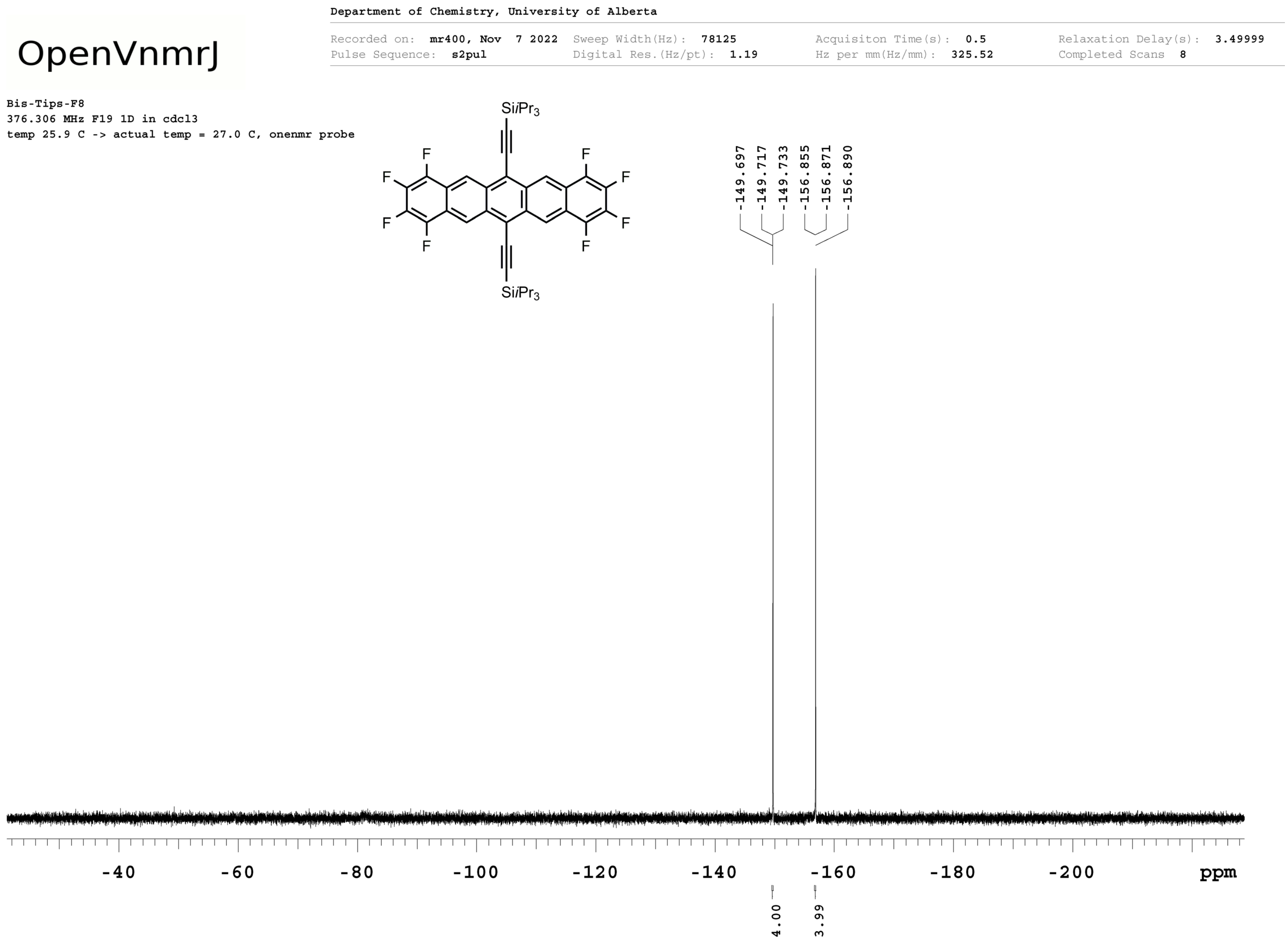

Figure S84. ^{19}F NMR spectrum of compound **7b(F$_8$)**, 376 MHz, $CDCl_3$.

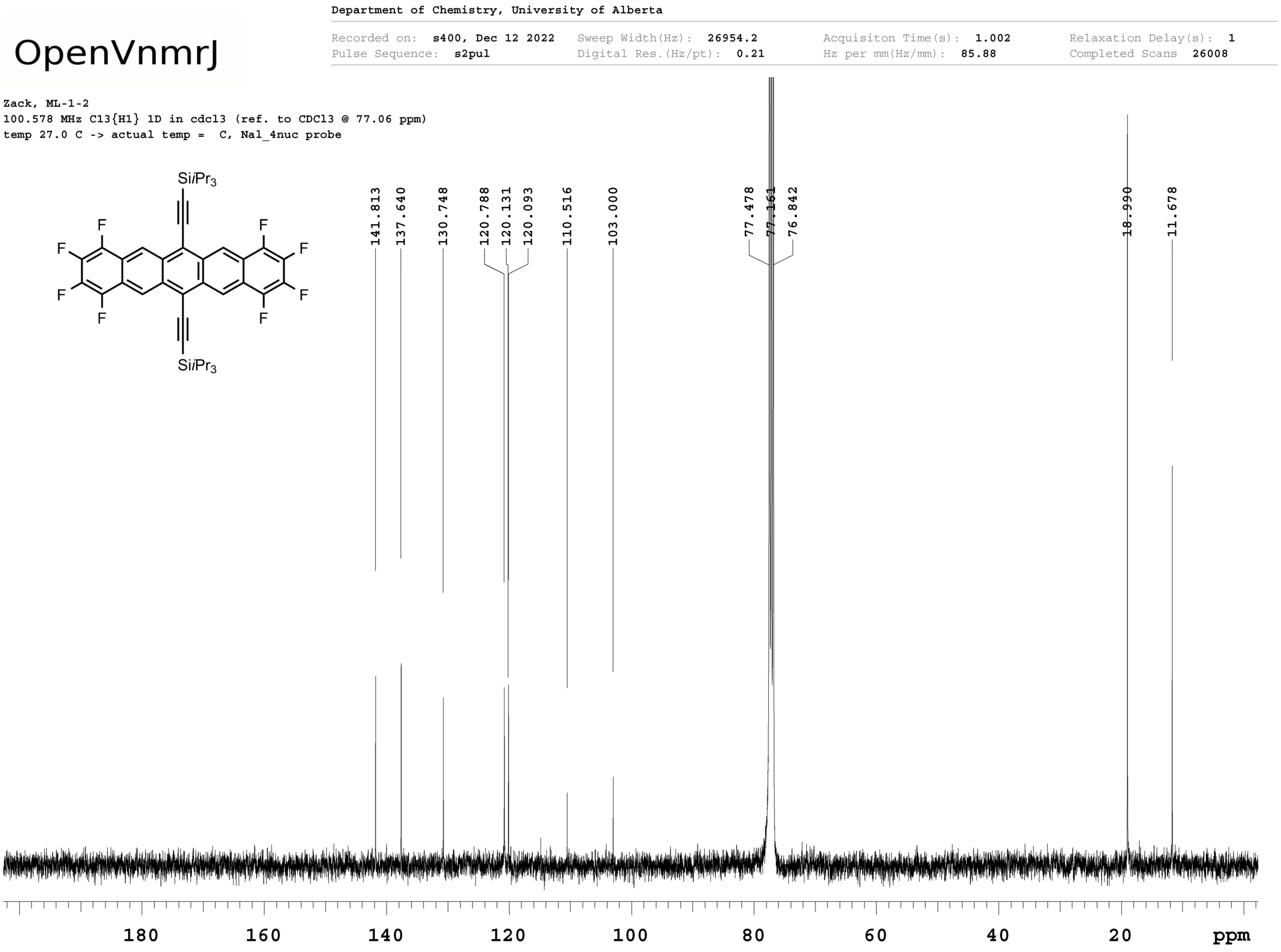

Figure S85. $^{13}C\{^1H\}$ NMR spectrum of compound **7b(F$_8$)**, 100 MHz, $CDCl_3$.

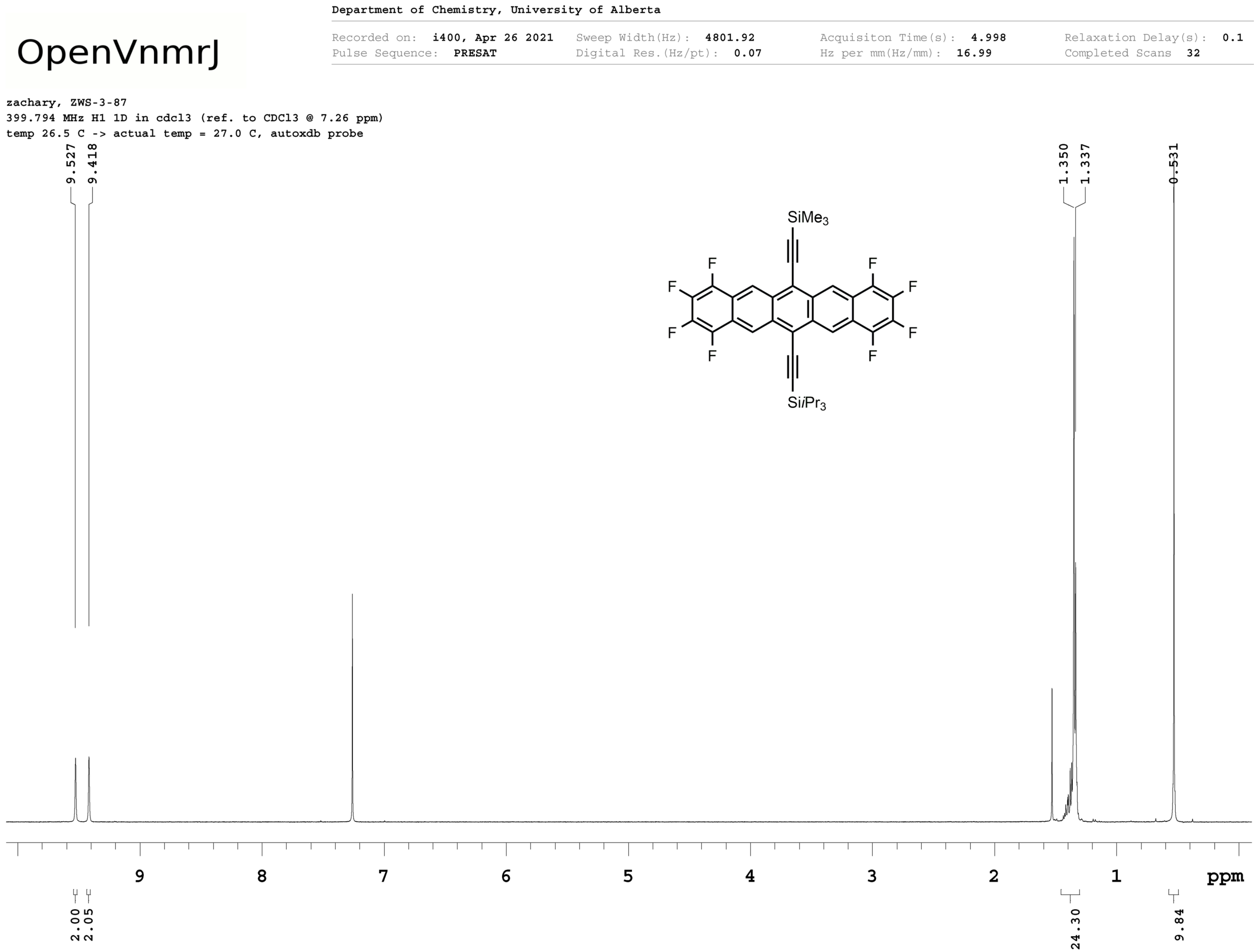

Figure S86. ^{1}H NMR spectrum of compound **7c(F$_8$)**, 400 MHz, CDCl$_3$.

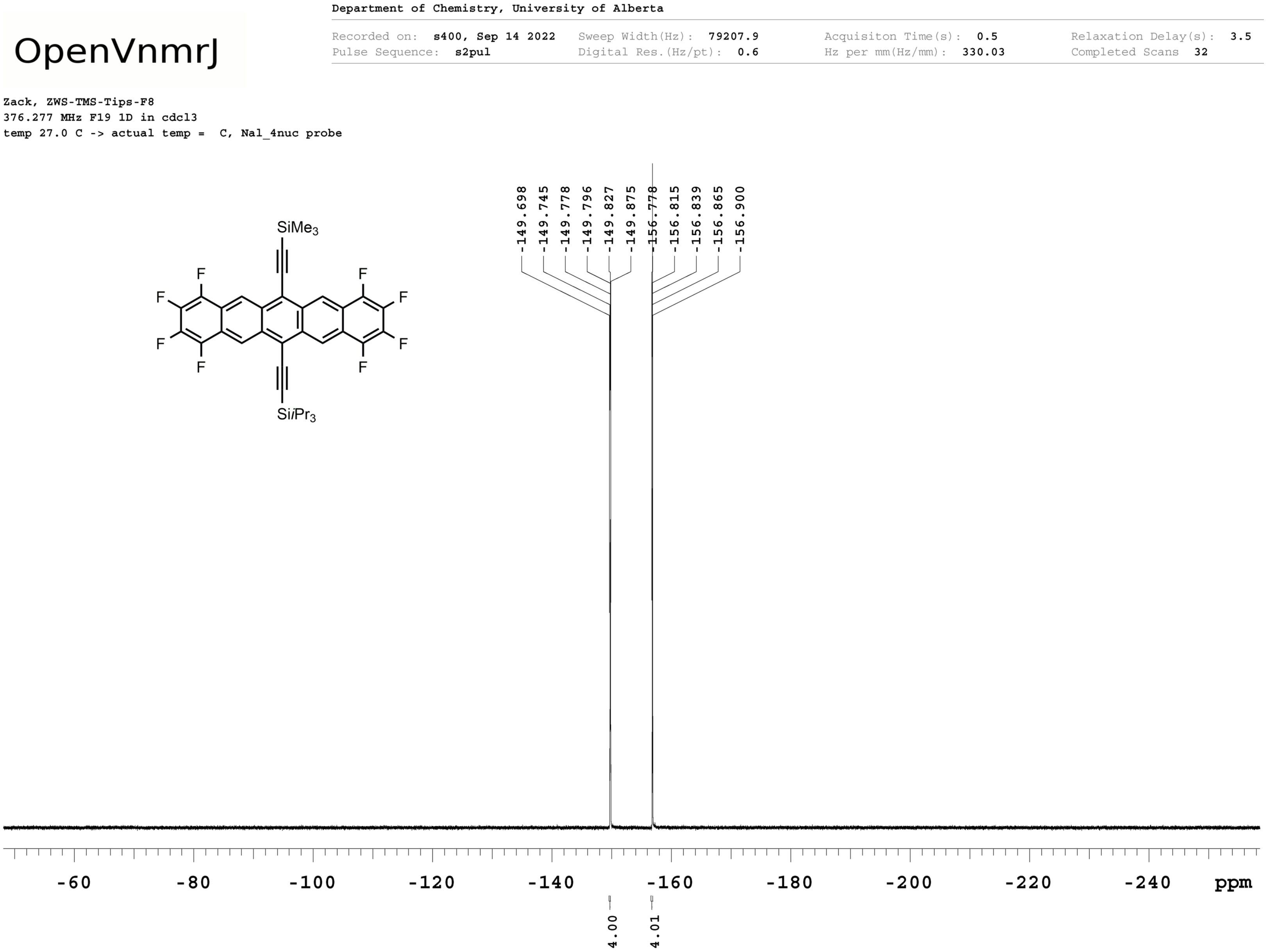

Figure S87. ^{19}F NMR spectrum of compound **7c(F$_8$)**, 376 MHz, $CDCl_3$.

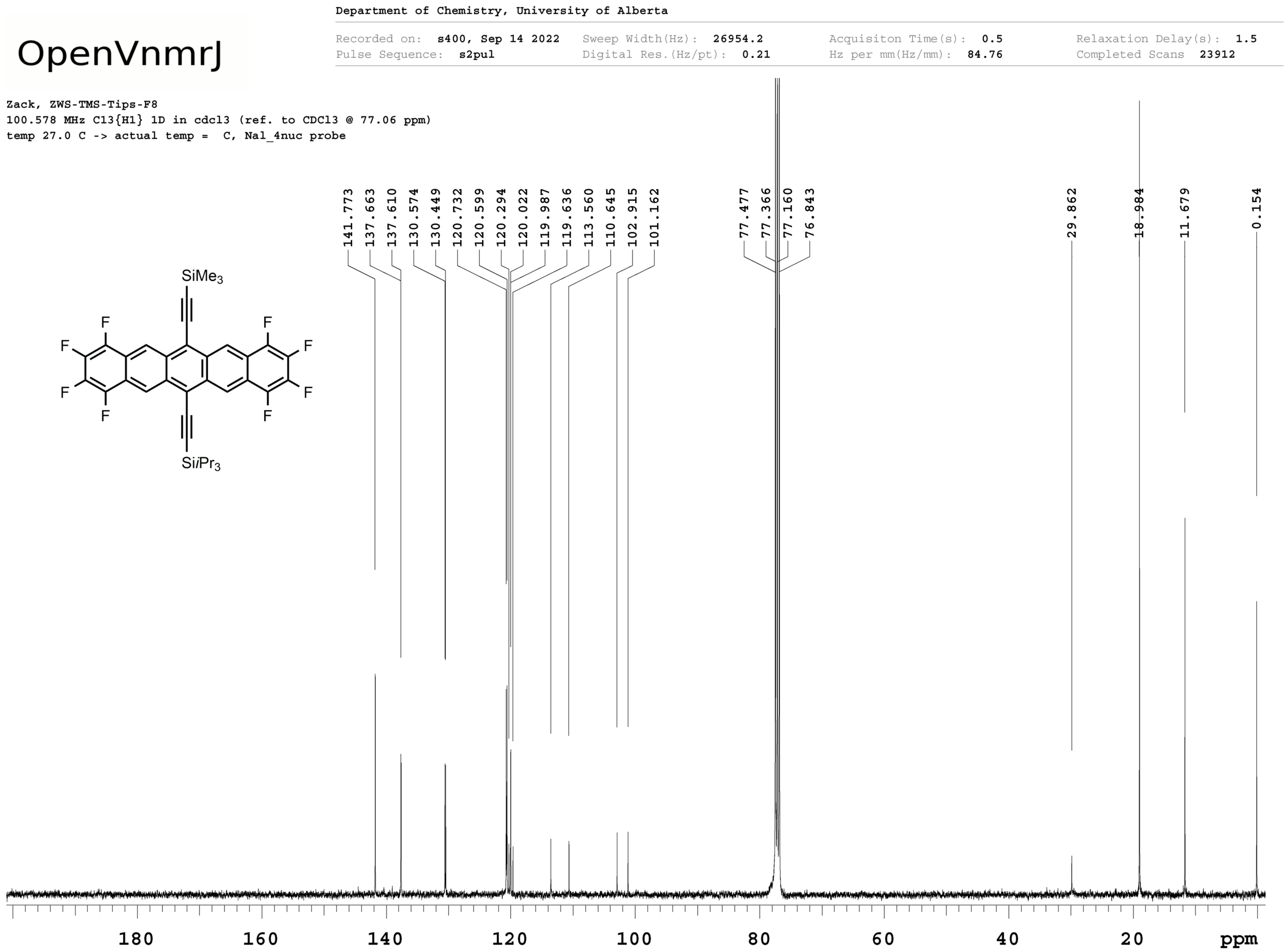

Figure S88. $^{13}C\{^{1}H, ^{19}F\}$ NMR spectrum inset of compound **7c(F$_8$)**, 100 MHz, $CDCl_3$.

Differential Scanning Calorimetry of compounds 2a–e, 2e(F8), 7b(F8), and 7c(F8)

Table S4. Spectral and thermal properties of compounds **2a**–**e** in comparison to compounds **7a**–**e**, **2e(F8)**, **7b(F8)**, and **7c(F8)**.

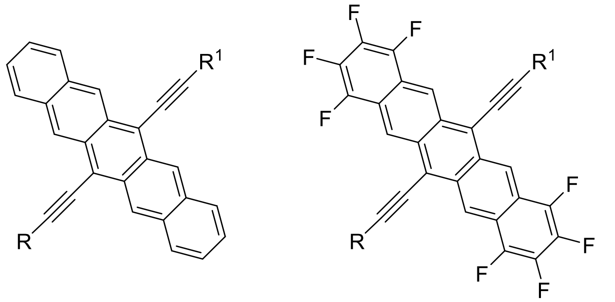

Compd	R¹	R²	DSC (°C)[a] mp	DSC (°C)[a] dp/ onset	DSC (°C)[a] dp/ peak	λmax (nm)[b]
**2a**	Tr*	Si*i*Bu3	263	263[c]	263[c]	644
**2b**	Tr*	Si*i*Pr3	317	317	319	642
**2c**	Tr*	SiMe3	198	204	209	642
**2d**	Tr*	Ph	–	109	135	652
**2e**	Tr*	Tr*	321	321	322	642
**2e(F8)**	Tr*	Tr*	305	–	–	635
**7a**[4]	Si*i*Bu3	Si*i*Bu3	213	213	215	644
**7b**[5]	Si*i*Pr3	Si*i*Pr3	263	263	265	643
**7c**[5]	Si*i*Pr3	SiMe3	158	160	167	642
**7d**[5]	Si*i*Pr3	Ph	–	174	180	652
**7e**[6]	Ph	Ph	–	246	265	660
**7b(F8)**	Si*i*Pr3	Si*i*Pr3	313	313	315	631
**7c(F8)**	Si*i*Pr3	SiMe3	155[d]	289	329[c]	629

[a] Measured under an atmosphere of N_2; mp = melting point; dp = decomposition point, onset, and peak temperatures. [b] Measured in CH_2Cl_2 at rt. [c] Value approximate, clear decomposition point not observed. [d] Undefined endotherm observed; no melt expected near this range based on melting point analysis.[7]

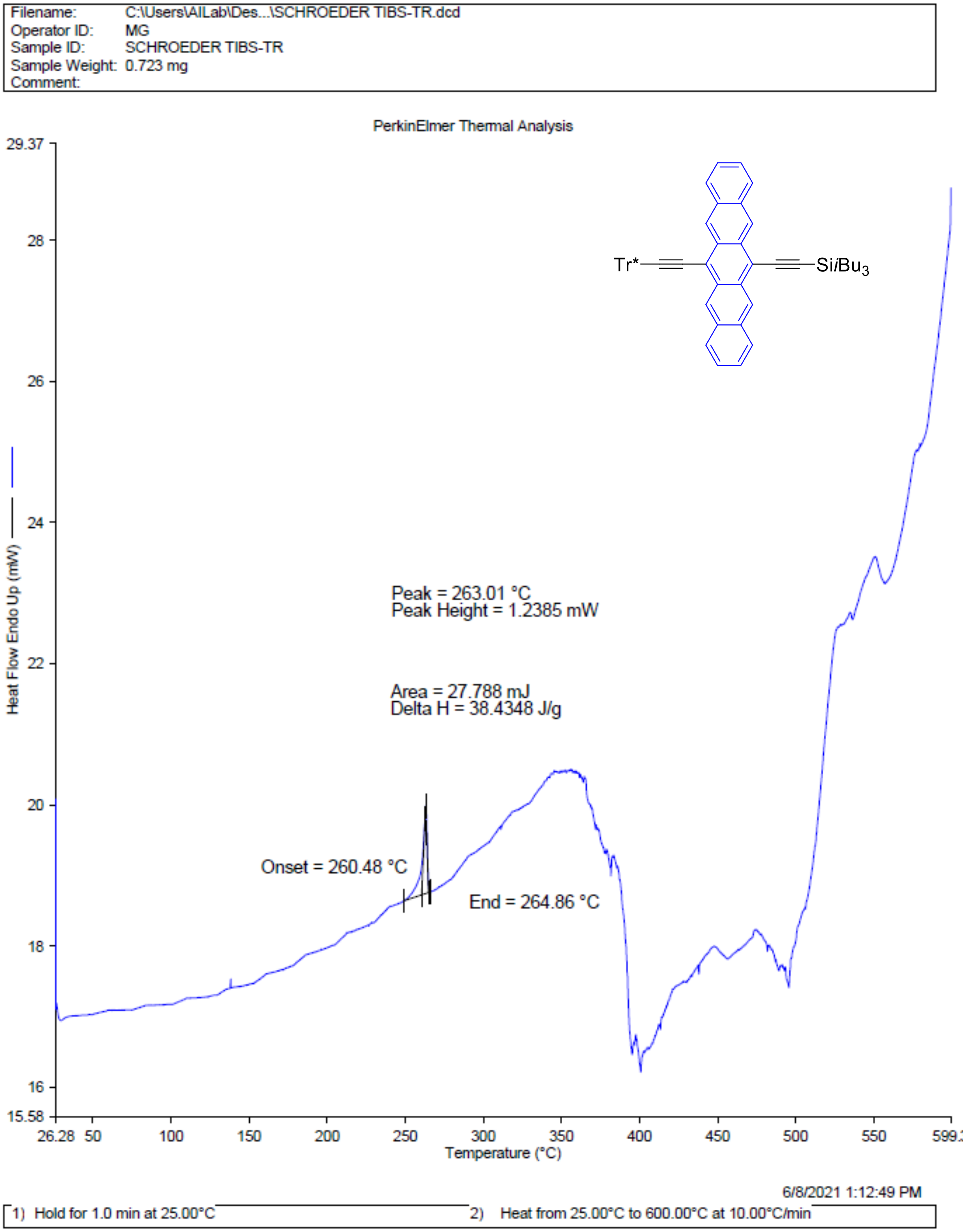

Figure S89. DSC analysis of compound **2a**.

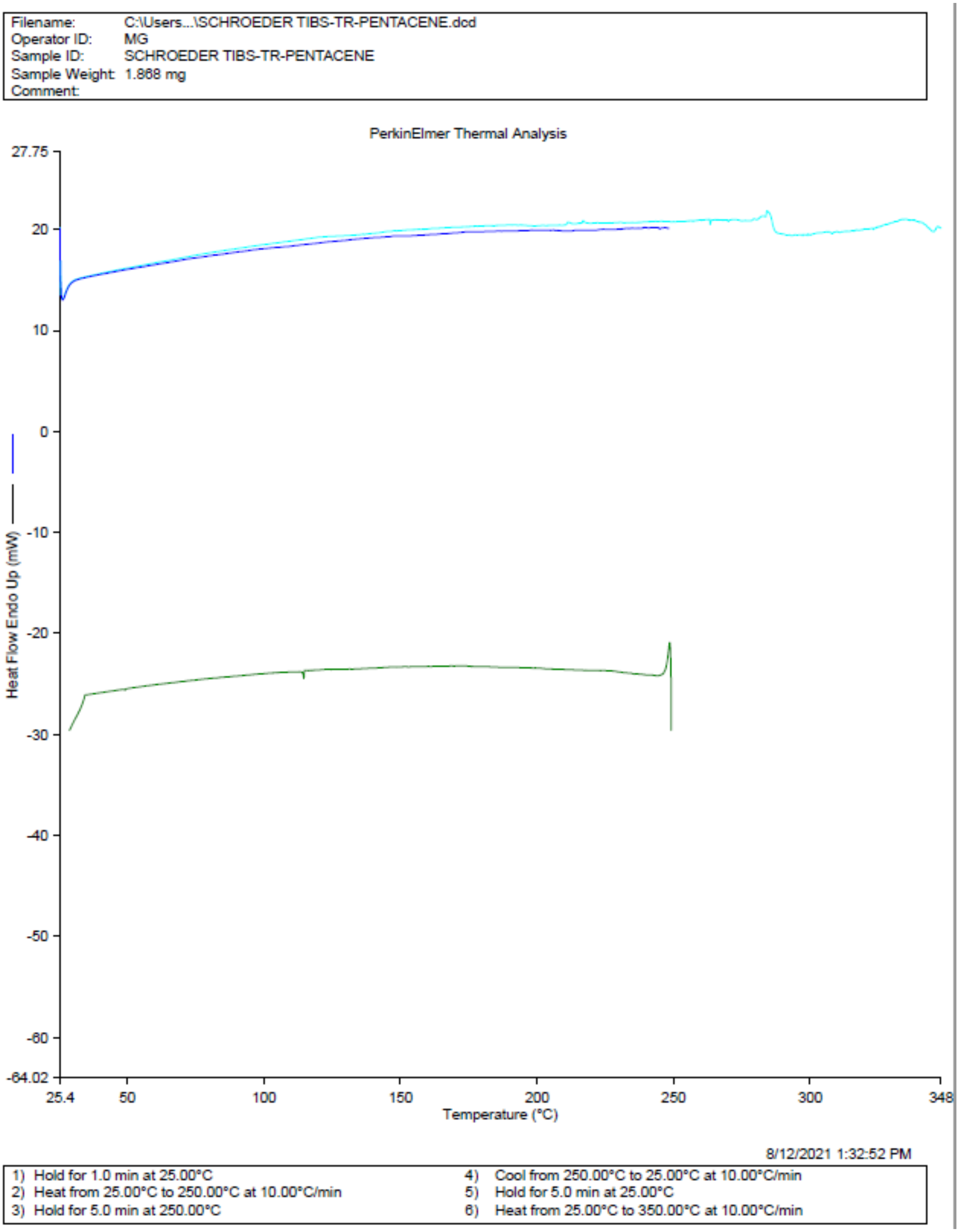

Figure S90. DSC analysis of compound **2a**. Initial heating to melt (green), cooling back to ambient temp. (dark blue), and reheating past melting point (teal).

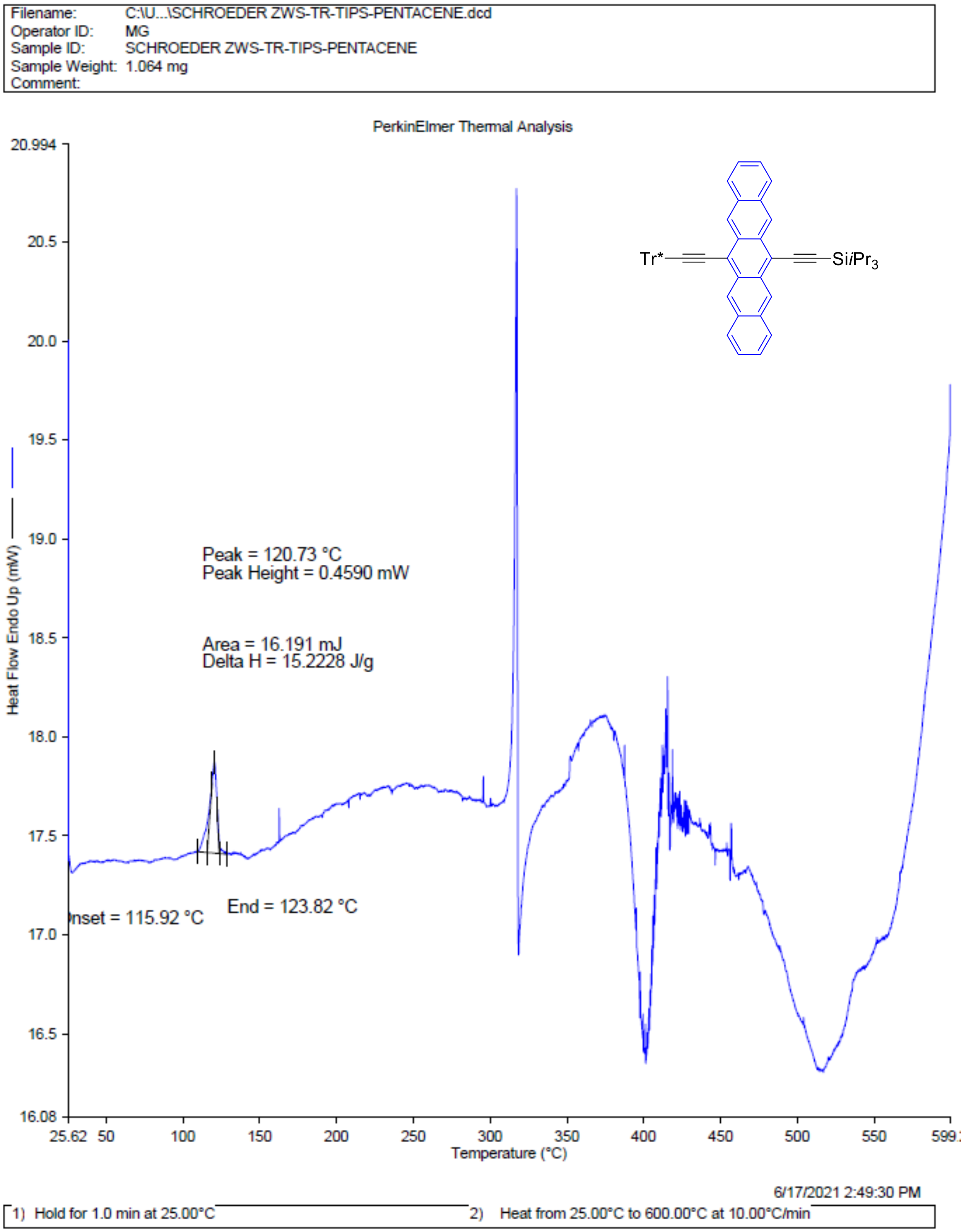

Figure S91. DSC analysis of compound **2b**.

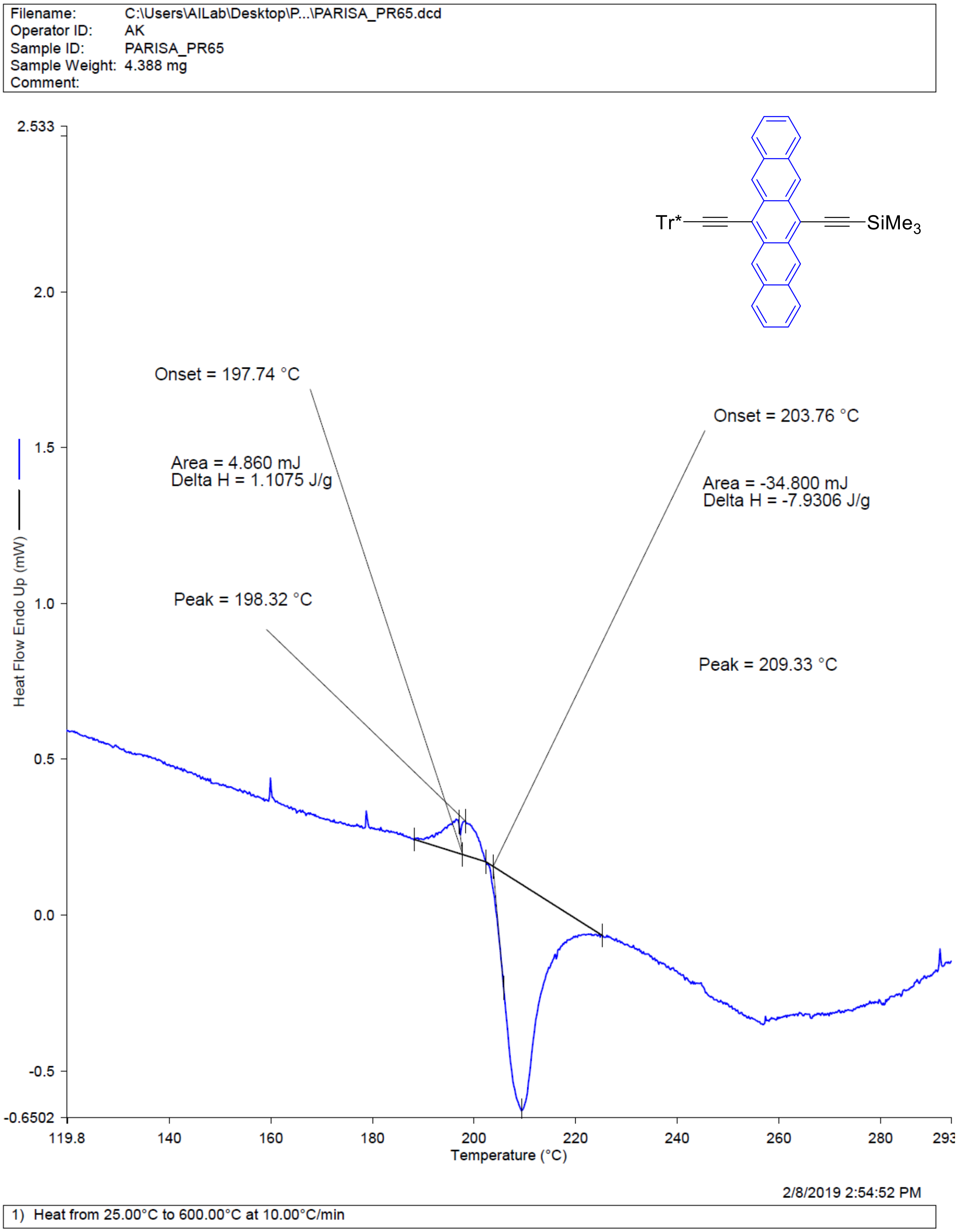

Figure S92. DSC analysis of compound **2c**.

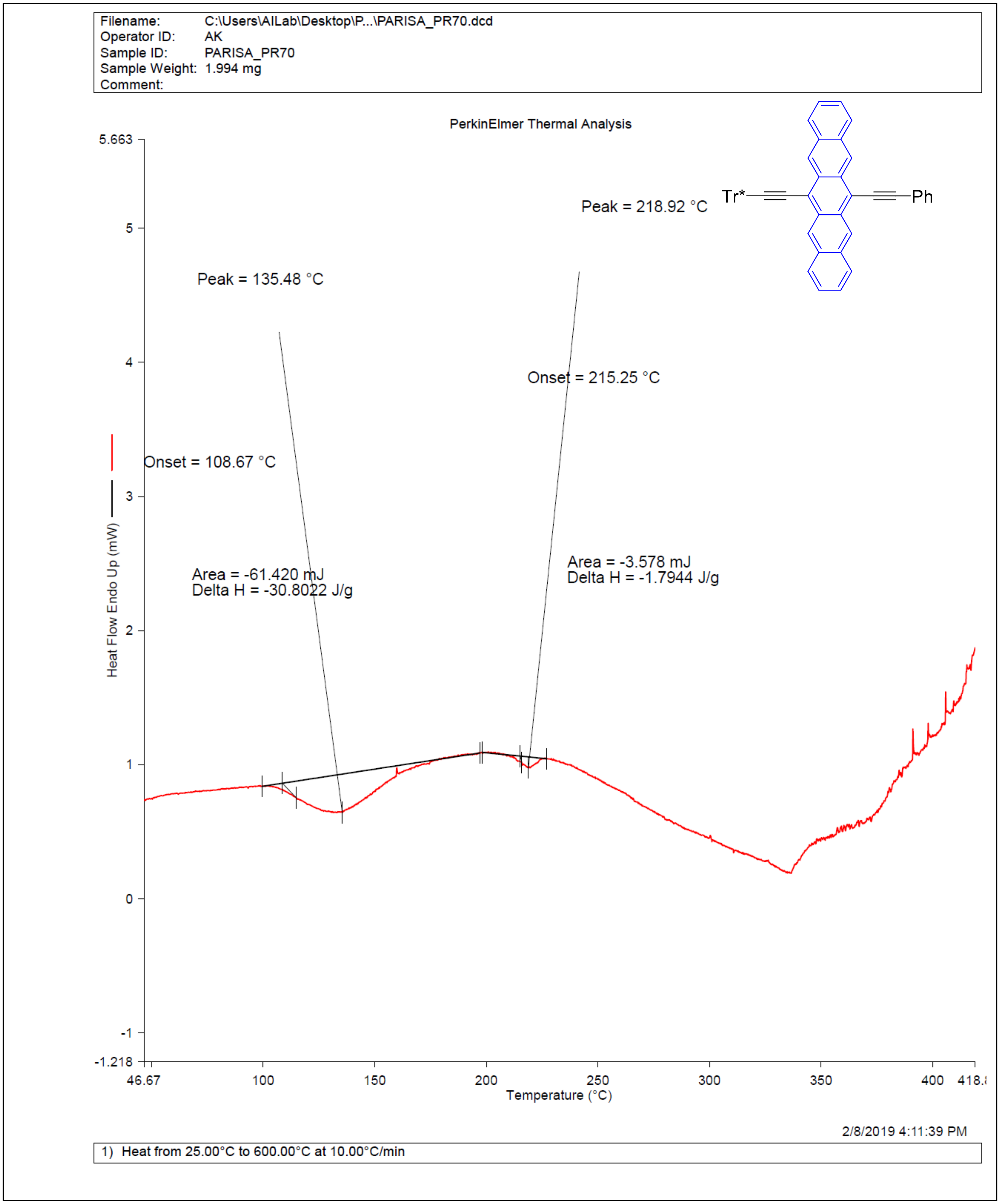

Figure S93. DSC analysis of compound **2d**.

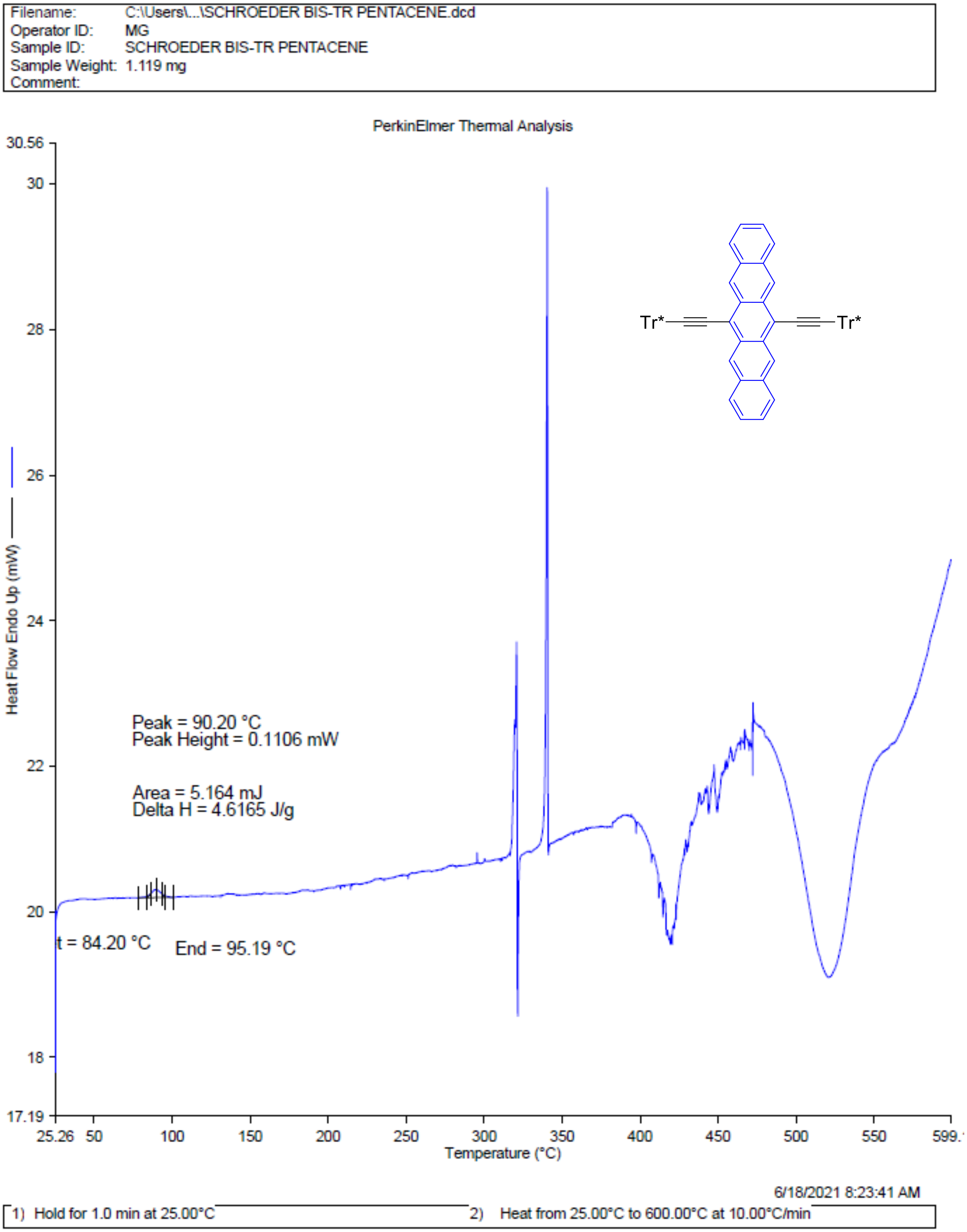

Figure S94. DSC analysis of compound **2e**.

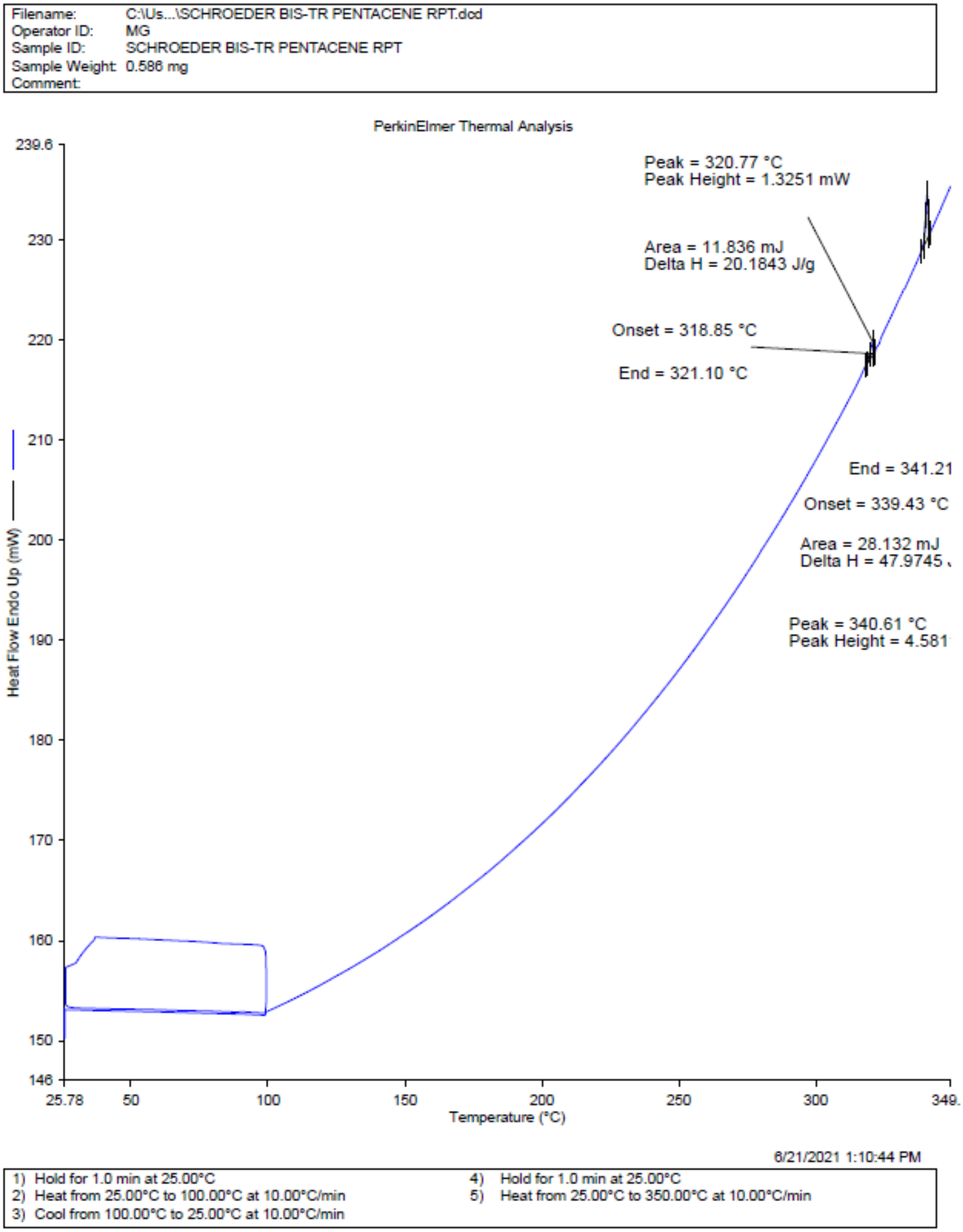

Figure S95. DSC analysis of compound **2e**.

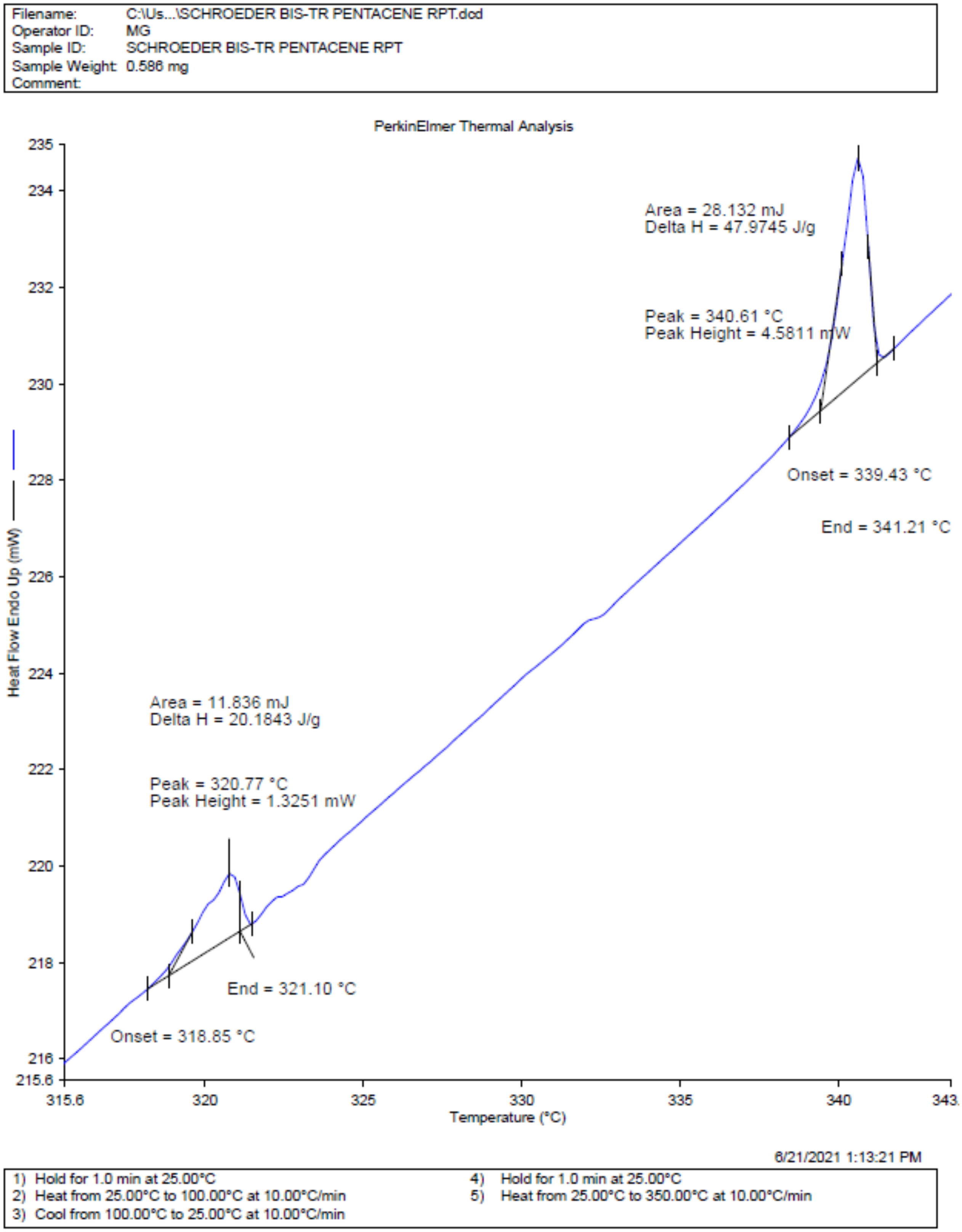

Figure S96. DSC analysis of compound **2e** expansion.

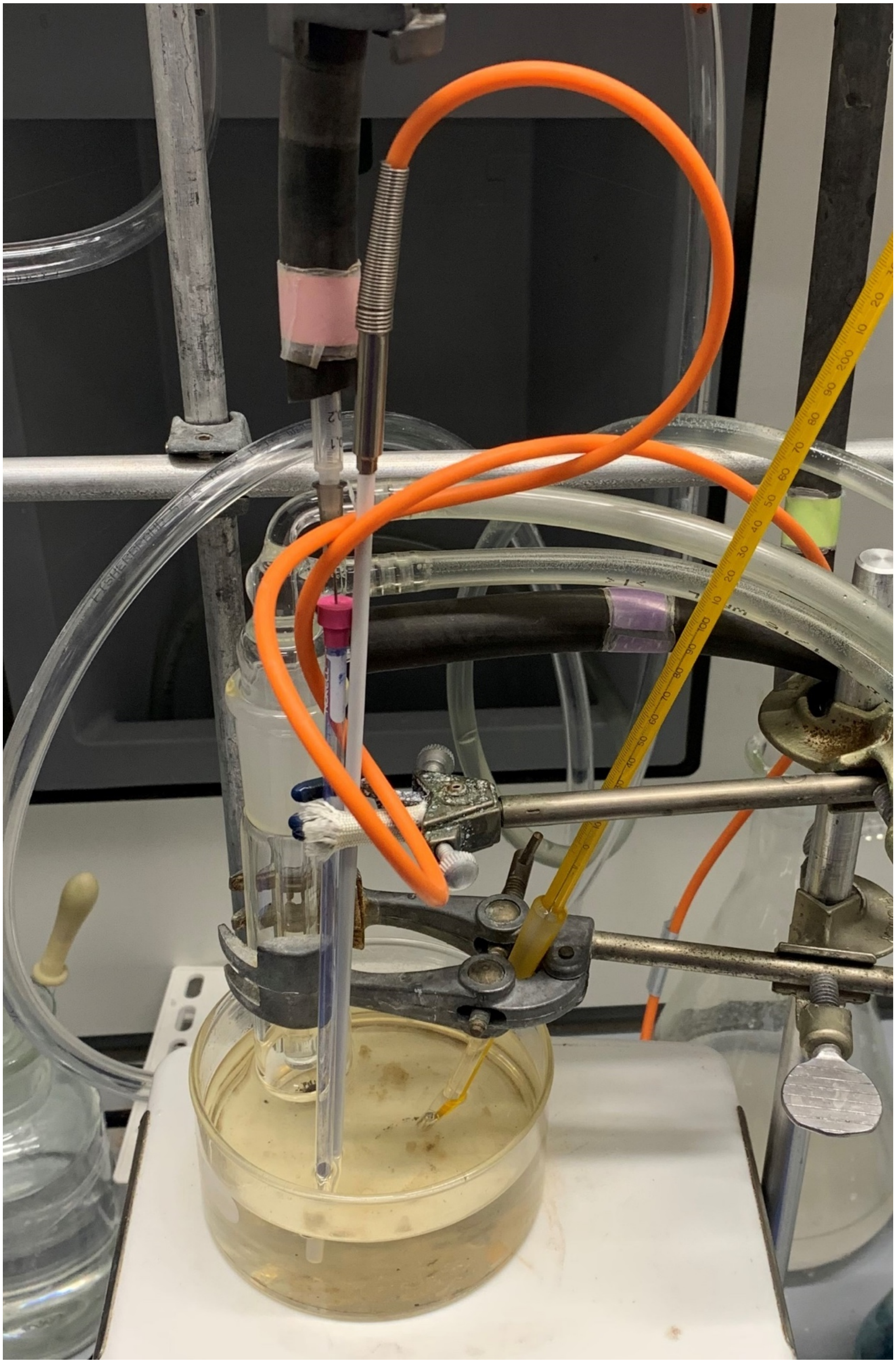

Figure S97. Assessment of the phase for compound **2e** under N_2 at 100 °C.

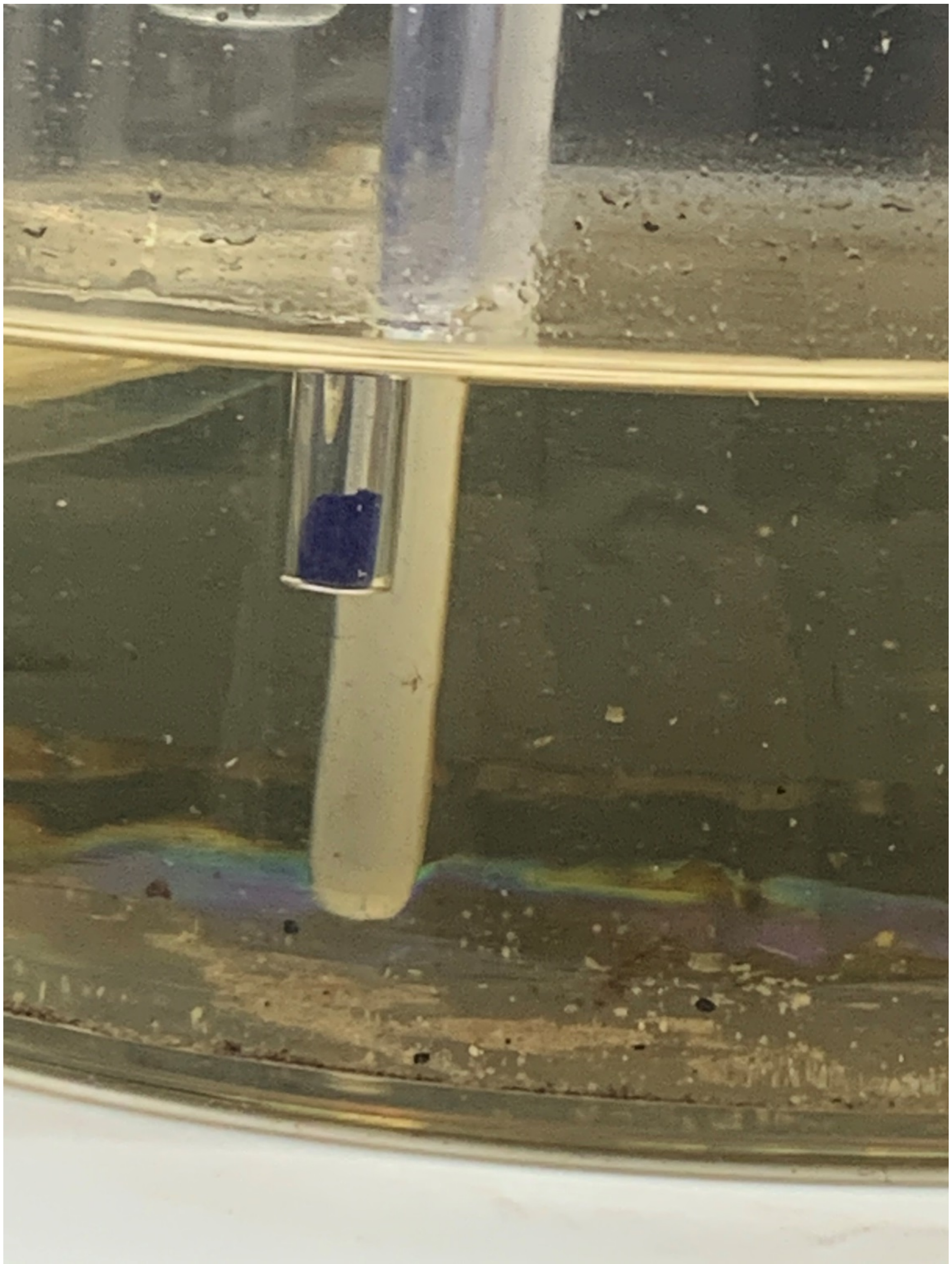

Figure S98. Assessment of the phase for compound **2e** under N_2 at 100 °C expansion.

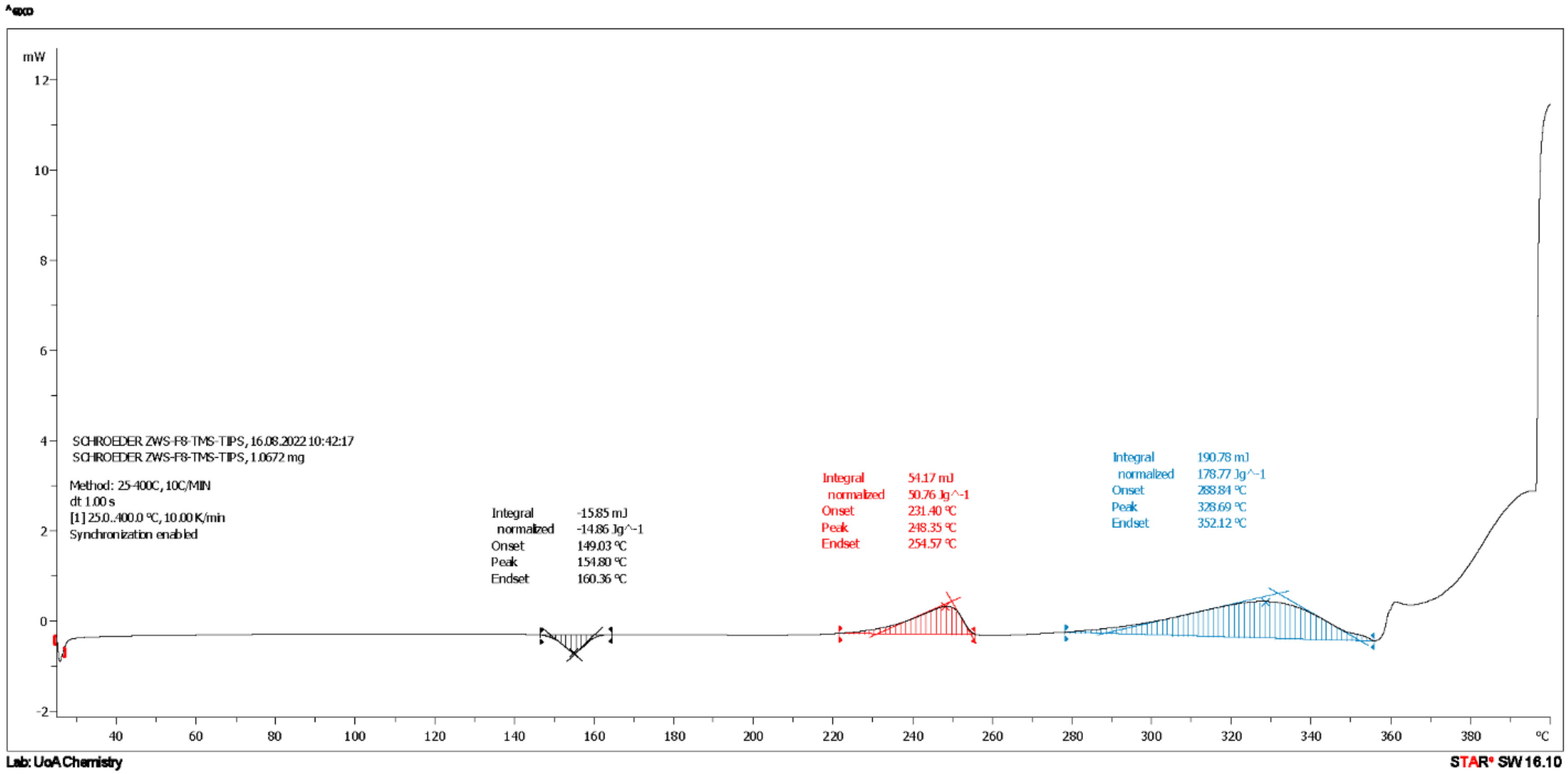

Figure S99. DSC analysis of compound **7c(F8)**.

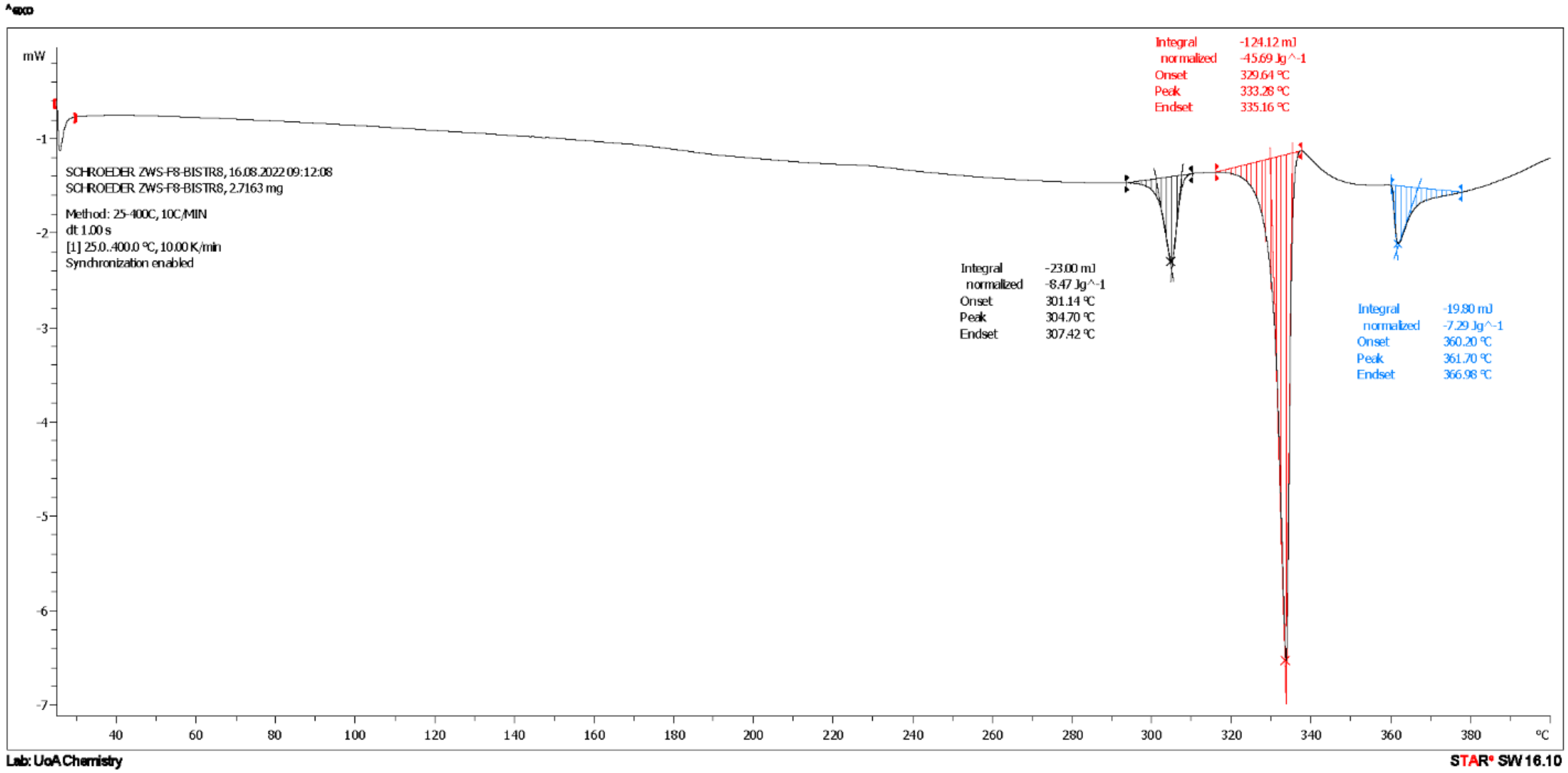

Figure S100. DSC analysis of compound **2e(F8)**.

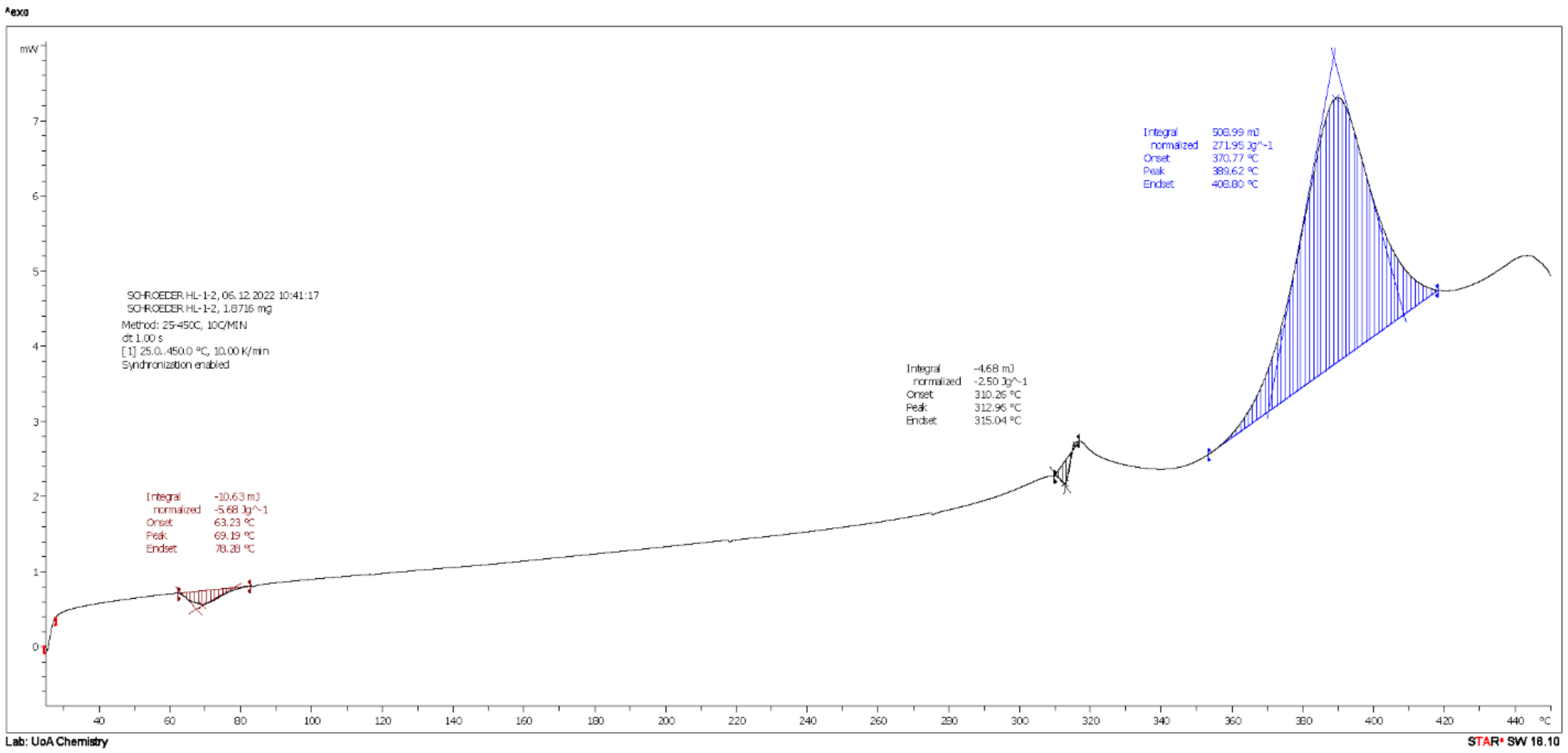

Figure S101. DSC analysis of compound **7b(F8).**

Electrochemical redox potentials

Cyclic voltammetry experiments have been used to probe the stabilizing effects of the trialkylsilyl substituents. A trend in the electrochemical bandgaps (E_g) was obtained for the series **2a**, **2b**, **2e**, and **7a**. A decrease in E_g of ca. 0.07 eV is observed when Tr* substituents are replaced with trialkylsilyl substituents. This observation of a decreasing bandgap is consistent with independent reports from Maliakal[8] and Miller[9].

Table S5. Cyclic voltammetry derived redox potentials of 7a, 2a, and 2e

Compound	Ox_1 (eV)	Red_1 (eV)	E_g
**7a**	0.826	–1.01	1.84
**2a**	0.782	–1.12	1.90
**2e**	0.802	–1.23	1.98

Cyclic voltammetry experiments were run with a platinum disk electrode and Ag/AgCl (silver wire) reference electrode. $TBAPF_6$ (0.1 M) electrolyte was used in CH_2Cl_2 with a substrate concentration of 1 mM and a ferrocene internal reference. The experimental sweep rate was 100 mV/s.

References

[1] J. L. Marshall, F. Arslan, J. A. Januszewski, M. J. Ferguson, R. R. Tykwinski, *Helv. Chim. Acta* **2019**, *102*, e1900001.

[2] Z. W. Schroeder, R. McDonald, M.J. Ferguson, R. R. Tykwinski, D. Lehnherr, *J. Org. Chem*. **2022**, *87*, 16236–16249.

[3] J. B. Sherman, B. Purushothaman, S. R. Parkin, C. Kim, S. Collins, J. Anthony, T. Nguyen, M. L. Chabinyc, *J. Mater. Chem. A* **2015**, *3*, 9989–9998.

[4] D. Lehnherr, A. H. Murray, R. McDonald, R. R. Tykwinski, *Angew. Chem. Int. Ed.* **2010**, *49*, 6190–6194.

[5]D. Lehnherr, R. McDonald, R. R. Tykwinski, *Org. Lett.* **2008**, *10*, 4163–4166.

[6] D. Lehnherr, M. Adam, A. H. Murray, R. McDonald, F. Hampel, R. R. Tykwinski, *Can. J. Chem.* **2017**, *95*, 303–314.

[7] J. Chen, J. Anthony, D. C. Martin, *J. Phys. Chem. B* **2006**, *110*, 16397–16403.

[8] A. Maliakal, K. Raghavachari, H. Katz, E. Chandross, T. Siegrist, *Chem. Mater.* **2004**, *16*, 4980–4986.

[9] I. Kaur, W. Jia, R. P. Kopreski, S. Selvarasah, M. R. Dokmeci, C. Pramanik, N. E. McGruer, G. P. Miller, *J. Am. Chem. Soc*. **2008**, *130*, 16274–16286.
